# NAPRT-mediated deamidated NAD biosynthesis enhances colon tissue resiliency and suppresses tumorigenesis

**DOI:** 10.1038/s41467-026-68998-w

**Published:** 2026-02-10

**Authors:** Xiaoyue Wu, Jason G. Williams, Haoyang Liang, Artiom Gruzdev, Joshua Hartsell, Jack Shpargel, Rabina Mainali, Yi Fang, Ming Ji, Caroline Duval, Xin Xu, Zixin Zhang, Heather Winter, Peter Pediaditakis, Arun R. Pandiri, Marie E. Migaud, Alan K. Jarmusch, Huimin Yu, Xiaojing Liu, Jian-Liang Li, Xiaojiang Xu, Igor Shats, Xiaoling Li

**Affiliations:** 1 Molecular and Cellular Biology Laboratory, National Institute of Environmental Health Sciences, Research Triangle Park, NC 27709 USA; 2 Mass Spectrometry Research and Support Group, National Institute of Environmental Health Sciences, Research Triangle Park, NC 27709 USA; 3 Department of Pathology and Laboratory Medicine, Tulane University School of Medicine, New Orleans, LA 70112 USA; 4 Gene Editing and Mouse Model Core Facility, National Institute of Environmental Health Sciences, Research Triangle Park, NC 27709 USA; 5 Department of Molecular and Structural Biochemistry, North Carolina State University, Raleigh, NC 27695 USA; 6 Epigenetics and Stem Cell Biology Laboratory, National Institute of Environmental Health Sciences, Research Triangle Park, NC 27709 USA; 7 Division of Gastroenterology and Hepatology, Johns Hopkins University School of Medicine, Baltimore, MD 21205 USA; 8 Immunity, Inflammation, and Disease Laboratory, National Institute of Environmental Health Sciences, Research Triangle Park, NC 27709 USA; 9 Molecular Pathology Group, National Institute of Environmental Health Sciences, Research Triangle Park, NC 27709 USA; 10 Experimental Pathology Laboratories, Morrisville, NC 27560 USA; 11 Mitchell Cancer Institute, University of South Alabama, Mobile, AL 36606 USA; 12 Integrative Bioinformatics, National Institute of Environmental Health Sciences, Research Triangle Park, NC 27709 USA

**Keywords:** Tumour-suppressor proteins, Colon cancer, Homeostasis

## Abstract

Nicotinamide adenine dinucleotide (NAD) is synthesized through both amidated salvage and deamidated pathways. Although NAD-producing enzymes are often overexpressed in cancer cells to meet the high metabolic demands of rapid proliferation and are considered oncogenic, we report that physiological levels of nicotinic acid phosphoribosyl transferase (NAPRT), the first enzyme in the Preiss-Handler arm of the deamidated pathways, suppress tumorigenesis. We show that NAPRT is enriched in gut epithelial cells, where it sustains the NAD pool for an efficient response to stress-induced acute NAD depletion. Consequently, NAPRT deficiency impairs the activity of poly-(ADP-ribose) polymerases and DNA repair, sensitizes mice to chemical-induced colitis and tumorigenesis, as well as to age-associated spontaneous tumor development. Moreover, low NAPRT expression correlates with poor prognosis in several human cancer types. Thus, homeostatic levels of deamidated NAD biosynthesis contribute to tumor suppression, and boosting this pathway may offer a strategy for cancer prevention.

## Abstract (web summary)

Nicotinamide adenine dinucleotide (NAD) biosynthetic enzymes have been reported to be oncogenic. Here, the authors show that NAPRT, a key enzyme in the deamidated NAD biosynthesis pathway, functions as a suppressor of colon inflammation and tumorigenesis by maintaining DNA repair capability.

## Introduction

Nicotinamide adenine dinucleotide (NAD), a cofactor for a variety of electron-exchange-dependent biochemical redox reactions, is central in energy metabolism and a molecule essential for life^1^. It also serves as an indispensable co-substrate for the sirtuin protein deacylases, the mono and poly-(ADP-ribose) polymerases (PARPs) involved in DNA repair, and cellular surface cyclic ADP ribose hydrolase CD38, and thereby NAD is important in regulation of genome stability, immune responses, circadian rhythms, and aging^2^.

Mammalian cells have multiple synthetic pathways for NAD, including the de novo pathway from tryptophan, the Preiss-Handler pathway (i.e., deamidated salvage pathway) from nicotinic acid (NA), and the amidated salvage pathway from nicotinamide (NAM), as well as additional pathways using precursors such as nicotinamide riboside (NR) and nicotinamide mononucleotide (NMN)^3,4^ (Supplementary Fig. 1a). Among these pathways, the amidated salvage whereby NAM is converted to NAD by nicotinamide phosphoribosyl transferase (NAMPT), is considered the primary source of NAD in most mammalian cells and tissues^5,6^.

Cellular NAD homeostasis is tightly maintained by its consumption and biosynthesis. Disruption of this balance is associated with various pathological conditions. For instance, reduced cellular NAD levels are observed in various diseases and aging^7^. Consequently, different therapeutic strategies aimed at boosting NAD levels are being actively investigated, including supplementation with NAD precursors such as NAM, NR, and NMN, activation of NAMPT, or inhibition of CD38^8^. On the other hand, actively proliferating cancer cells and active immune cells require high NAD flux, and NAMPT has been shown to be upregulated in many human cancers as well as various inflammatory conditions, such as rheumatoid arthritis and inflammatory bowel disease (IBD)^9–14^. Nicotinic acid phosphoribosyl transferase (NAPRT), the first enzyme in the deamidated salvage pathway, is also reported to have a high frequency of amplification in tumors derived from tissues that normally have high expression of NAPRT^9^. Therefore, inhibitors of the amidated or the deamidated NAD biosynthesis pathways are under development for potential treatment of cancer^9,10,15–18^ and inflammation^12,14^.

Interestingly, although the amidated NAD salvage pathway is considered the main pathway of NAD production in mammals^19^, recent studies uncovered an important role of the deamidated NAD biosynthesis pathway in mediating the host-microbe NAD metabolic interaction^20^. Particularly, following oral administration of amidated precursors such as NAM or NR, gut microbiota can convert these dietary amidated NAD precursors to NA and enable the deamidated NAD biosynthesis^21^. In several organs, including the liver, colon, and kidney, this microbiota-dependent deamidated biosynthesis accounts for the majority of newly synthesized NAD^21^. Moreover, in the absence of dietary consumption of NAD precursors, gut microbiome can convert host-derived NAM in the gut lumen into NA, maintaining host NAD biosynthesis through the deamidated pathway^22^. However, in contrast to the well-studied NAMPT-mediated amidated NAD salvage pathway, the (patho)physiological importance of this microbiota-enabled highly efficient NAD biosynthesis pathway is largely understudied, and the functional difference between these two NAD biosynthesis pathways remains unclear.

In the present study, we investigate the functional impacts of the deamidated NAD biosynthesis pathway on animal (patho)physiology and stress responses and identify an unexpected suppressive role of this pathway in both cancer and inflammation.

## Results

### NAPRT is enriched and functionally important in gut epithelial cells

**Fig. 1:**
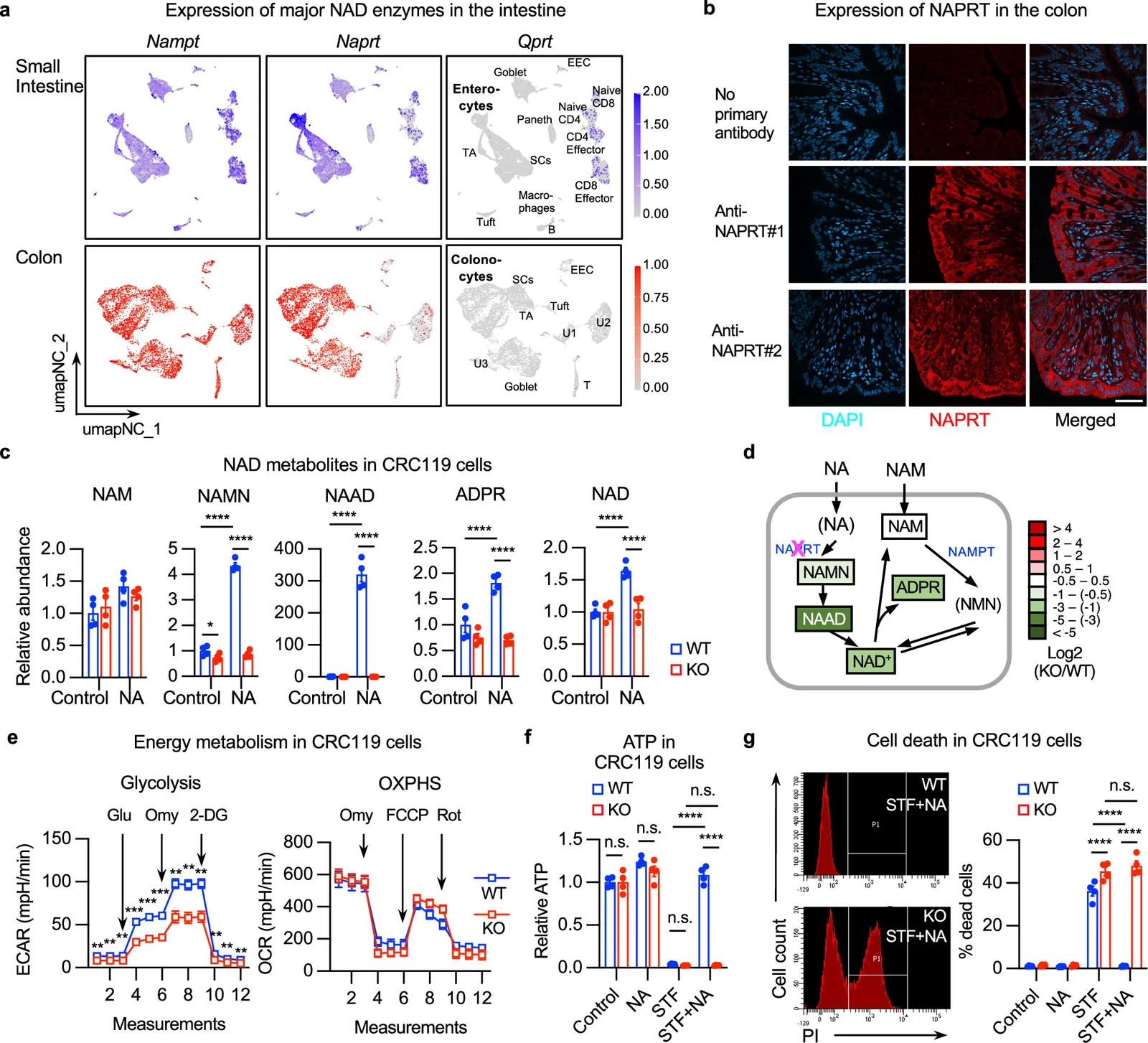
NAPRT deficiency impairs energy metabolism and sensitizes gut epithelial cells to NAMPT inhibition. **a** *Naprt* is highly expressed in enterocytes in the small intestine and colonocytes in the colon. The expression of key NAD biosynthesis enzymes was analyzed using scRNA-seq datasets (*n* = 27,737 total small intestinal single cells and 10,297 total colonic single cells). SCs stem cells, EEC enteroendrocrine cells, TA transit-amplifying cells, U1 undefined cells 1, U2 undefined cells 2, U3, undefined cells 3. **b** NAPRT is highly expressed in colonocytes in the colon. Immunofluorescence staining of WT mouse colon sections with two different anti-NAPRT antibodies. The experiment was independently repeated in 3 different mice with similar results. Bar, 50 μm. **c** Intracellular NAD-related metabolites in WT and NAPRT KO cells cultured in growth medium with or without 100 μM NA for 48 hours. All metabolites were measured by LC-MS (n = 2 clonal cell lines/group in biological duplicates, values are normalized to the WT controls and expressed as mean ± SEM; two-way ANOVA). **d** The log ratios of the relative abundance of metabolites in NAPRT KO vs WT cells cultured in NA-containing medium (in **c**) are represented by a color scale. Metabolites in parentheses were not analyzed in this experiment. **e** Extracellular acidification rate (ECAR) and oxygen consumption (OCR) in WT and NAPRT KO CRC119 cells. Cells cultured with 100 μM NA for 48 hours were analyzed by Seahorse XFe24 Analyzer. Raw readings were normalized to total protein content (n = 10 biological replicates, values are expressed as mean ± SEM; two-tailed unpaired multiple Mann-Whitney tests). Glu, glucose; Omy, oligomycin; 2-DG, 2-deoxy-glucose; FCCP, carbonyl cyanide-4 (trifluoromethoxy)phenylhydrazone; Rot, rotenone. **f** Cellular ATP levels in WT and NAPRT KO CRC119 cells cultured in medium with or without 100 μM NA or 100 nM STF for 48 hours (*n* = 4 biological replicates/group, values are normalized to the corresponding genotype controls and expressed as mean ± SEM; two-way ANOVA). **g** Cell death in WT and NAPRT KO CRC119 cells cultured with or without 20 μM NA or 100 nM STF for 42 hours (*n* = 4 biological replicates/group, values are expressed as mean ± SEM; two-way ANOVA). Source data are provided as a Source Data file.

To explore the potential function of deamidated NAD biosynthesis, we decided to focus on the gut, the direct interface for host-microbe metabolic interaction. Single cell RNA-seq (scRNA-seq) analysis of genes encoding key enzymes of the three major NAD biosynthesis pathways, including *Nampt*, *Naprt*, and quinolinate phosphoribosyl transferase (*Qprt)*, the rate-limiting enzyme in the de novo synthesis pathway, in normal mouse gut revealed that both the small intestine and the colon have very low expression of *Qprt* (Fig. 1a). As a comparison, all three enzymes were highly expressed in the liver, particularly in hepatocytes (Supplementary Fig. 1b, using a publicly available mouse hepatic scRNA-seq dataset^23^). Among two highly expressed NAD biosynthesis enzymes in the gut, *Nampt* was widely expressed in most cells (including immune cells), whereas *Naprt* was highly expressed in gut epithelial cells, particularly in enterocytes in the small intestine and colonocytes in the colon (Fig. 1a). The enrichment of NAPRT in the mouse colonocytes was confirmed by immunofluorescence staining using two independent anti-NAPRT antibodies (Fig. 1b). Similar expression patterns of these enzymes were also observed in human colon based on the scRNA-seq datasets from The Human Protein Atlas (www.proteinatlas.org) (Supplementary Fig. 1c). Enterocytes and colonocytes are the most abundant intestinal epithelial cells responsible for nutrient absorption and transport^24^. They are also in direct contact with gut microbiota in the gut lumen and immune cells inside the gut lamina propria. The enrichment of NAPRT in these gut epithelial cells strongly suggests that NAPRT-mediated deamidated NAD biosynthesis is critical in mediating diet-microbe-host interaction that influences gut physiology and pathology.

To test the direct functional impacts of NAPRT and deamidated NAD biosynthesis in gut epithelial cells, we knocked out NAPRT in a human colon cancer cell line CRC119^25^ using CRISPR/Cas9-mediated gene editing (Supplementary Fig. 1d). NAPRT KO CRC119 cells displayed normal morphology and proliferation rates when cultured in a regular growth medium containing 8 μM NAM but no NA (Supplementary Fig. 1e, f). Supplementation of 100 μM NA, a concentration comparable to the serum NA range after oral supplementation with NAD boosting precursors^22^, markedly induced deamidated NAD intermediates NAMN and NAAD and significantly increased total NAD abundance in WT but not in NAPRT KO CRC119 cells (Fig. 1c, d). Further analysis of extracellular acidification rate (ECAR) and oxygen consumption rate (OCR) revealed that the defective deamidated NAD biosynthesis observed in NAPRT KO CRC119 cells cultured in NA-containing medium impaired their glycolysis but not oxidative phosphorylation (OXPHS) (Fig. 1e), suggesting that NAPRT-mediated deamidated NAD biosynthesis contributes to cytosolic NAD pool. However, this reduced glycolysis in NAPRT KO CRC119 cells cultured in the NA-containing medium was not associated with any reduction in ATP production or increase in cell death unless NAMPT was inhibited by a specific inhibitor STF-118804 (Fig. 1f, g, STF + NA). Therefore, NAPRT deficiency impairs cellular metabolism and increases the sensitivity to NAMPT inhibition-induced NAD depletion and subsequent cell death.

### NAPRT KO cells accumulate DNA damage

**Fig. 2:**
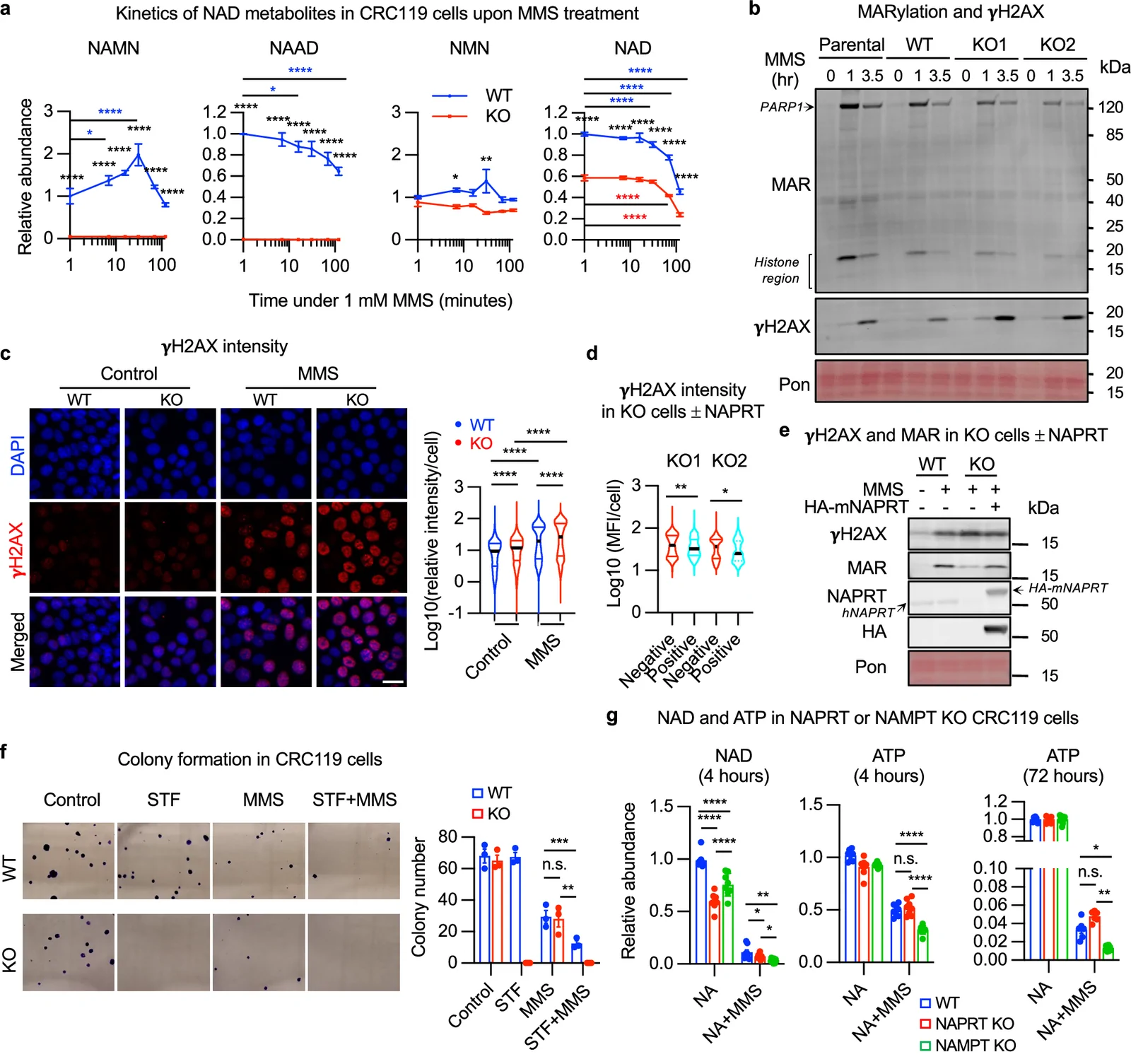
NAPRT deficiency increases cellular sensitivity of cells to chemically induced DNA damage. **a** Kinetics of NAD metabolites in WT and NAPRT KO cells cultured with 100 μM NA for 24 hours then treated with 1 mM MMS for indicated times (*n* = 3 biological replicates/group, values are normalized to WT time 0 and are expressed as mean ± SD; two-way ANOVA). **b** Protein MARylation and γH2AX levels in WT and NAPRT KO cells cultured with 20 μM NA for 16 hours then treated with 1 mM MMS for the indicated times. Similar experiments were repeated independently four times with comparable results. **c** Relative γH2AX immunofluorescence intensity in WT and NAPRT KO cells cultured with 100 μM NA for 48 hours then treated with or without 1 mM MMS for 1 hour (*n* = 2852, 2120, 2189, and 2319 cells, respectively; values are normalized to the WT controls; two-way ANOVA). Bar, 20 μm. **d** Mean γH2AX immunofluorescence intensity in two independent NAPRT KO clones transiently transfected with an NAPRT expressing construct. Cells were cultured with 20 μM NA, then treated with 1 mM MMS for 1 hour. Negative, cells negative for NAPRT; Positive, cells positive for NAPRT (n = 402, 330, 428, and 298 cells, respectively; two-tailed unpaired Mann-Whitney test). **e** γH2AX and histone MARylation levels in WT, NAPRT KO, and NAPRT KO cells stably infected with lentiviruses expressing HA-mNAPRT. Cells were cultured with 1 μM NA, then treated with 1 mM MMS for 4 hours. The experiment was repeated independently three times with similar results. **f** The colony formation ability of WT and NAPRT KO cells treated with MMS (*n* = 3 biological replicates/group, values are expressed as mean ± SEM; two-way ANOVA). **g** NAD and ATP levels in WT, NAPRT KO, and NAMPT KO cells cultured with 20 μM NA then treated with or without 1 mM MMS for 4 or 72 hours (for NAD (4 hours), *n* = 8 biological replicates/group; for ATP (4 hours), *n* = 8 biological replicates/group; for ATP (72 hours), *n* = 6, 6, 6, 6, 15, and 15 biological replicates, respectively; values are expressed as mean ± SEM; two-way ANOVA). Source data are provided as a Source Data file.

DNA damage is a common environmental stress that induces NAD consumption. Upon the induction of DNA damage, the PARP DNA repair enzymes consume large amounts of NAD as a substrate to synthesize poly-(ADP–ribose) (PAR) and mono-ADP-ribose (MAR) at or near DNA damage lesions to initiate DNA repair^26^. DNA-damaging reagents, therefore, are reported to induce nuclear and cellular NAD depletion^27–31^. Indeed, treatment with methyl methanesulfonate (MMS), a DNA alkylating agent that adds methyl groups to DNA preferentially at the N^7^ position of guanine^32^, induced a quick reduction of total cellular NAD in WT CRC119 cells cultured in medium supplemented with NA (Fig. 2a, WT, NAD). This acute reduction of NAD was coupled with a transient increase followed by a reduction of intracellular NAMN but not NMN (Fig. 2a, WT), suggesting that deamidated NAD biosynthesis, particularly NAPRT, was activated in response to MMS treatment. The level of NAAD followed the same trend as NAD (Fig. 2a, WT). Consistent with the observation in Fig. 1c, d, NAPRT KO CRC119 cells cultured in the same NA-containing medium had undetectable levels of the deamidated NAD precursors NAMN and NAAD, and a significantly reduced NAD content compared to WT cells before treatment (Fig. 2a, 0 minute). MMS treatment in these cells also induced an acute reduction of NAD. However, the reduction was slower than that in WT cells (Fig. 2a, KO, NAD). Moreover, WT cells cultured in medium without NA supplementation, thereby possessing only amidated NAD synthesis, had a slow NAD depletion rate comparable to that in NAPRT KO cells (Supplementary Fig. 2a, bottom).

In agreement with their reduced NAD consumption upon DNA damage in NA-containing medium (Fig. 2a and Supplementary Fig. 2a), NAPRT KO cells had reduced induction of protein PARylation, including histone PARylation, after MMS treatment and recovery (Supplementary Fig. 2b, c). Moreover, when cultured in a medium containing 20 μM NA, a dose resulting in the maximal increase of NAD in CRC119 cells (Supplementary Fig. 2d), MMS-induced auto- Mono-ADP-ribosylation (MARylation) of PARP1 (upper band) and MARylation of histones (lower band) were reduced in NAPRT KO cells (Fig. 2b). Histone PARylation and MARylation are important for efficient DNA repair^33,34^. Consistently, the reduction of protein PARylation and MARylation in NAPRT KO cells after MMS treatment was associated with increased DNA damage as assessed by γH2AX immunoblotting (Fig. 2b, γH2AX) and immunofluorescence staining (Fig. 2c and Supplementary Fig. 2e). Importantly, transient re-expression of NAPRT in NAPRT KO cells was able to significantly suppress γH2AX levels upon MMS treatment (Fig. 2d and Supplementary Fig. 2f). Furthermore, when cultured in a medium containing 1 μM NA, which is equivalent to the plasma concentration of NA in mice on a regular chow diet^22^, NAPRT KO CRC119 cells had increased γH2AX levels and decreased histone MARylation following MMS treatment compared to WT cells (Fig. 2e, KO + MMS vs WT + MMS). Stably adding-back HA-tagged mouse NAPRT (HA-mNAPRT) restored MARylation and reduced γH2AX levels (Fig. 2e). Together, our observations indicate that NAPRT deficiency in cells reduces the activation of PARP upon DNA damage, impairing DNA repair and increasing the sensitivity to DNA damage.

As both deamidated and amidated NAD biosynthesis were active in CRC119 cells (Fig. 1), we sought to test the relative importance of these two pathways for cell viability following DNA damage using two different experimental strategies. In the first strategy, we used NAMPT inhibitor STF-118804  (STF) treatment (100 nM STF for 24 hours) in WT cells to inhibit the amidated pathway (WT STF), and NAPRT KO to inactivate the deamidated pathway, and STF treatment in NAPRT KO cells (KO STF) to inactivate both amidated and deamidated pathways. We then challenged these cells with MMS for 1 hour to induce transient DNA damage. Cells were subsequently washed, and long-term cell viability was assessed using colony formation assay. Inhibition of amidated NAD biosynthesis sensitized WT cells to MMS-induced cell death (Fig. 2f, WT STF + MMS vs WT MMS), whereas inactivation of the deamidated synthesis failed to do so (Fig. 2f, KO MMS vs WT MMS). Inactivation of both pathways completely suppressed cell survival (Fig. 2f, KO STF + MMS vs WT MMS). In the second strategy, we generated NAMPT KO CRC119 cells (Supplementary Fig. 2g), and cultured WT, NAPRT KO, and NAMPT KO cells in a medium containing 20 μM NA and 8 μM NAM. We then treated them with or without MMS for 4 hours or 72 hours (Fig. 2g). Despite a significant NAD reduction (Fig. 2g, NA; NAD (4 hours)), either the amidated or the deamidated pathways were sufficient to maintain cellular ATP levels in untreated cells cultured in this full medium (NA; ATP (4 hours)). Treatment with MMS for 4 hours depleted cellular NAD levels by 90% in all cells and markedly reduced cellular ATP levels. Yet compared to WT cells, NAPRT KO cells displayed comparable cellular ATP levels whereas NAMPT KO cells had significantly reduced cellular ATP (NA + MMS; ATP (4 hours)). Similar results were observed after cells were treated with MMS for 72 hours (NA + MMS; ATP (72 hours)). These observations are consistent with our colony formation assay in Fig. 2f, suggesting that NAPRT KO CRC119 cells have a comparable survival ability as the WT cells in response to the genotoxic stress. Therefore, in contrast to NAMPT-mediated amidated NAD salvage, the deamidated pathway is not essential for cell survival in response to genotoxic stress. Taken together, our results indicate that NAPRT-mediated deamidated NAD biosynthesis significantly contributes to DNA repair upon DNA damage. However, despite the accumulation of more severe DNA damage compared to WT cells, a comparable fraction of NAPRT-deficient cells survives the stress.

### NAPRT is important for NAD biosynthesis and stress response in mice

To further investigate the functional importance of deamidated NAD biosynthesis in vivo, particularly in the gut, we generated a mutant mouse model in which a 21-bp fragment encoding 7 amino acids in the catalytic domain of NAPRT was deleted in all cells using the CRISPR/Cas9-mediated gene editing technology (Supplementary Fig. 3a). This deletion did not affect total *Naprt* mRNA levels in multiple tested tissues (Supplementary Fig. 3b) but led to slightly increased electrophoretic mobility and reduced intensity of NAPRT protein bands, particularly in the liver (Supplementary Fig. 3c). These observations suggest that the 7-amino acid deletion in this mutant line destabilizes the NAPRT protein. In mouse embryonic fibroblasts (MEFs) and primary macrophages differentiated from bone marrow of this mouse line, cellular NAD biosynthesis stimulated by NA in the presence of NAMPT inhibitor STF-118804 was completely blocked (Supplementary Fig. 3d), indicating that this mutant line is a true NAPRT null (KO) allele.

**Fig. 3:**
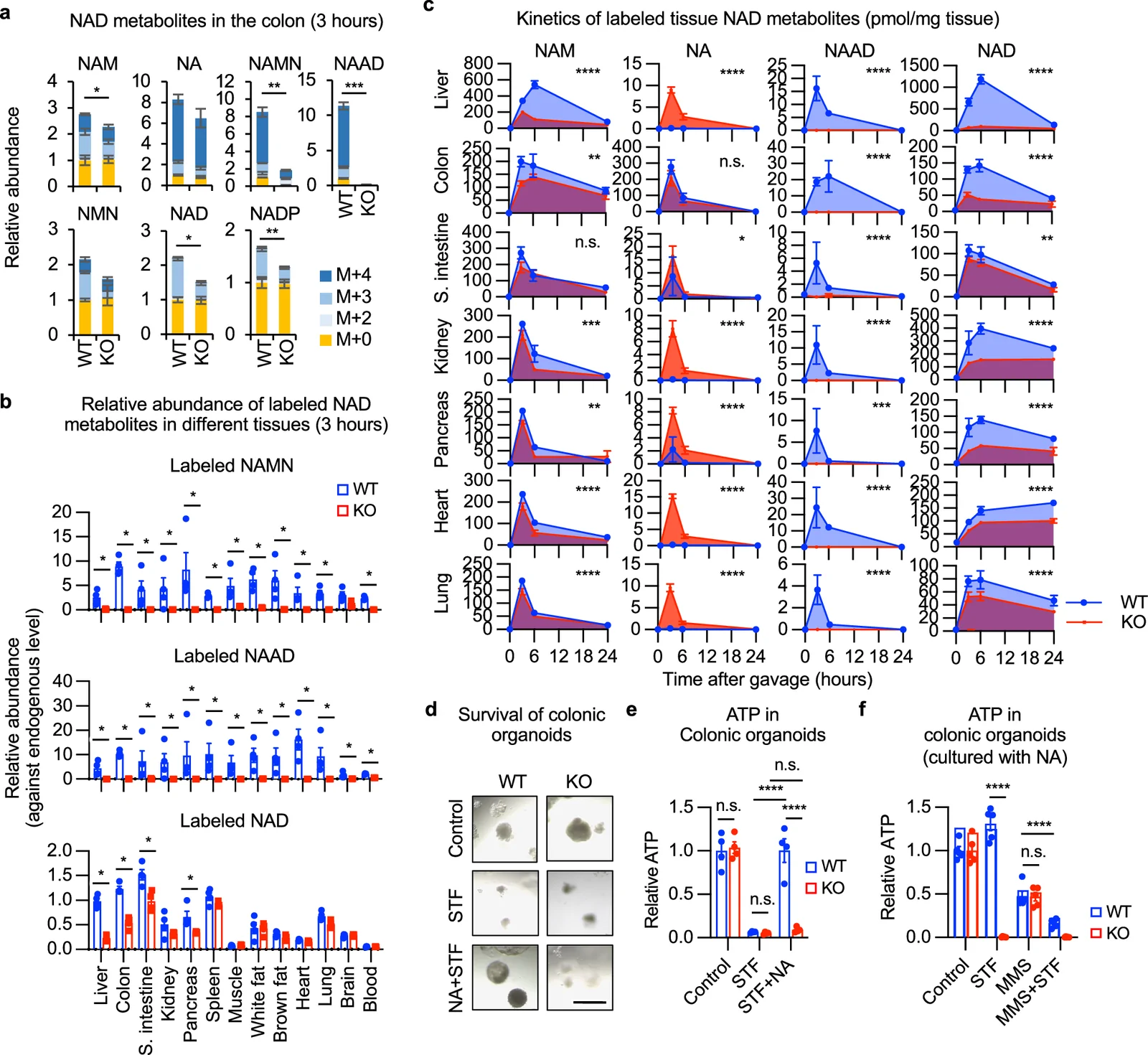
NAPRT-mediated deamidated pathway is important for NAD biosynthesis in vivo and gut epithelial cell stress response in vitro. **a** NAPRT is important for colonic NAD boosting effect of oral NAM supplementation. WT and NAPRT KO mice were treated and analyzed at 3 hours after dosing, as in Supplementary Fig. 1e, and indicated NAD metabolites in the colons were measured by LC-MS (*n* = 4 mice/genotype, values are normalized to the endogenous non-labeled (M + 0) level in WT mice and expressed as mean ± SEM; two-tailed unpaired Student’s *t*- test). **b** Relative abundance of newly synthesized NAD metabolites in WT and NAPRT KO mice. Mice were treated and analyzed as in Supplementary Fig. 1e (n = 4 mice/genotype; values represent the sum of all labeled isotopologs and are normalized to the level of the corresponding non-labeled (M + 0) metabolites in WT mice and expressed as mean ± SEM; two-tailed unpaired Mann-Whitney test). S. Intestine, small intestine. **c** Kinetics of labeled NAD metabolites in WT and NAPRT KO mice. Mice were treated and analyzed as in Supplementary Fig. 1e. Area Under the Curve (AUC) for WT and NAPRT KO mice were compared for each panel (For the 0 and 24 hours timepoints, n = 4 mice/genotype; For the 3 and 6 hours timepoints, n = 4 WT and 5 KO mice; concentrations are expressed as mean ± SEM; two-tailed unpaired Student’s t-test). S. Intestine, small intestine. **d** Representative images of WT and NAPRT KO colonic organoids cultured with or without 100 μM NA or 100 nM STF for 72 hours. Bar, 250 μm. **e** ATP levels in WT and NAPRT KO colonic organoids cultured and treated in **d** (n = 4 biological replicates/group, values are normalized to the corresponding genotype controls and expressed as mean ± SEM; two-way ANOVA). **f** ATP levels in WT and NAPRT KO colonic organoids cultured with 100 μM NA and with or without 100 nM STF and 400 μM MMS for 72 hours. The survival (ATP levels) of organoids was analyzed by 3D CTG assay (*n* = 5 biological replicates/group, values are normalized to the corresponding genotype controls and expressed as mean ± SEM; two-way ANOVA). Source data are provided as a Source Data file.

NAPRT KO mice were born normally and did not display clear phenotypes in unstressed conditions at a young age, which contrasts with the embryonic lethality of mice lacking NAMPT^35^, indicating that deamidated NAD biosynthesis is not essential for development or normal functions of the experimental mice. The steady-state tissue NAD levels in several tested organs of NAPRT KO mice were normal compared to WT control mice (Supplementary Fig. 4a). However, stable isotopic tracing experiments following oral gavage of deuterium-labeled NAM (D4-NAM) (Supplementary Fig. 4b) demonstrated that deletion of NAPRT leads to a dramatic depletion of the deamidated intermediates NAMN and NAAD, along with a decrease in the levels of newly synthesized NAD and nicotinamide adenine dinucleotide phosphate (NADP) in the colon and liver 3 hours after dosing (Fig. 3a and Supplementary Fig. 4c, M + 4/M + 3/M + 2; the M + 3 labeling is the result of rapid turnover of the redox-active hydrogen; the M + 2 labeling is the result of spontaneous hydrogen-deuterium exchange combined with redox turnover^36^). As we and others previously elucidated, the induction of the deamidated NAD precursors NAMN and NAAD in WT mice following supplementation with NAM is a result of gut microbiota-mediated deamidation of NAM to NA^21,22^. Systematic investigation of additional tissues revealed that at the 3-hour time point, NAPRT-mediated deamidated NAD biosynthesis occurs in almost all tested tissues (Fig. 3b, as indicated by production of NAMN and NAAD) and significantly contributes to the levels of newly synthesized NAD in the small intestine and pancreas in addition to the colon and liver (Fig. 3b and Supplementary Fig. 4d, NAD).

Further kinetic analysis of tissue concentrations of NAD metabolites following oral gavage of D4-NAM confirmed that NAPRT-mediated deamidated biosynthesis is a major route of NAD biosynthesis in the liver, colon, kidney and pancreas and significantly contributes to NAD biosynthesis in the small intestine, heart and lung (Fig. 3c). The massive accumulation of NA in all tested NAPRT KO mouse tissues further indicates that NAD synthesis using NA as a substrate is active in all these tissues in WT mice. Moreover, in WT mice, robust NAAD and NAD biosynthesis occurred without significant accumulation of NA, even at the earliest time point examined (except for the colon) (Fig. 3c, NA), indicating that NAPRT-mediated NA assimilation is swift and highly efficient. Colon in WT mice had an extremely high level of NA due to its direct contact with gut microbiota, many of which bear a nicotinamidase (PncA) activity that converts NAM to NA as we previously reported^21^. Interestingly, in NAPRT KO mice, the accumulation of NA in all tested tissues was transient and peaked at or prior to 3 hours (Fig. 3c, NA), possibly due to the catabolism of NA into nicotinuric acid (NUA) in the kidney of these mice (Supplementary Fig. 4e, KO). Of note, NUA was undetectable in WT mice (Supplementary Fig. 4e, WT), suggesting that this catabolite may serve as a potential urinary biomarker for defective deamidated NAD biosynthesis. Recent studies have shown that NA can also be converted to NAR in an NAPRT-dependent manner^37^. NAR then acts as the primary circulating deamidated NAD precursor for certain tissues, such as the kidney^37,38^. Further isotope tracing experiments using D4-labeled NA revealed that in WT mice, oral gavage of D4-NA at 80 mg/Kg elevated plasma NA levels from below the detection limit of our platform (1-2 μM) to ~300 μM, along with a significant increase in plasma NAR levels (from 0.028 μM to 0.202 μM) (Supplementary Fig. 4f, WT). In NAPRT KO mice, the rise of NAR was almost completely abolished despite a higher increase of circulating NA (Supplementary Fig. 4f, KO), confirming the NAPRT-dependent conversion of NA to NAR. Additionally, D4-NA gavage elevated plasma levels of both basal (M + 0) and labeled (M + 3) NAM, peaking between 1-3 hours in WT but not in KO mice (Supplementary Fig. 4f, NAM), suggesting increased NAD consumption following NA-induced NAD boosting in WT mice. Similar results were observed with a higher dose of D4-NA (400 mg/Kg) (Supplementary Fig. 4f, right panels). All together, these results from NAPRT KO mice indicate the key role of the microbiota- and NAPRT-dependent deamidated pathway in mediating the NAD-boosting efficacy of various NAD precursors.

To confirm our observations in CRC119 cells that NAPRT deficiency increases the sensitivity of cells to NAD-depleting stresses, we cultured colonic and small intestinal organoids from WT and NAPRT KO mice then treated them with an NAMPT inhibitor

STF-118804 and/or DNA damaging reagent MMS. Consistent with our findings in CRC119 cell line, primary NAPRT KO colonic and small intestinal organoids were more sensitive to STF-induced ATP depletion and death in the presence of NA compared to WT organoids (Fig. 3d, e, Supplementary Fig. 4g, h). Moreover, we observed that although NAPRT KO colonic organoids cultured in NA-containing medium were sensitive to STF treatment (Fig. 3e, KO STF vs WT STF), they were not more sensitive to MMS induced death than WT colonic organoids (Fig. 3f, KO MMS vs WT MMS). Instead, STF-treated WT colon organoids had reduced survival compared to WT colonic organoids after MMS treatment (Fig. 3f, WT MMS + STF vs WT MMS). These observations support our conclusion from the CRC119 cells that deamidated pathway, although important for tissue NAD biosynthesis, is not essential for gut epithelial cell survival after genotoxic stress.

### NAPRT deficiency sensitizes mice to intestinal DNA damage and inflammation

**Fig. 4:**
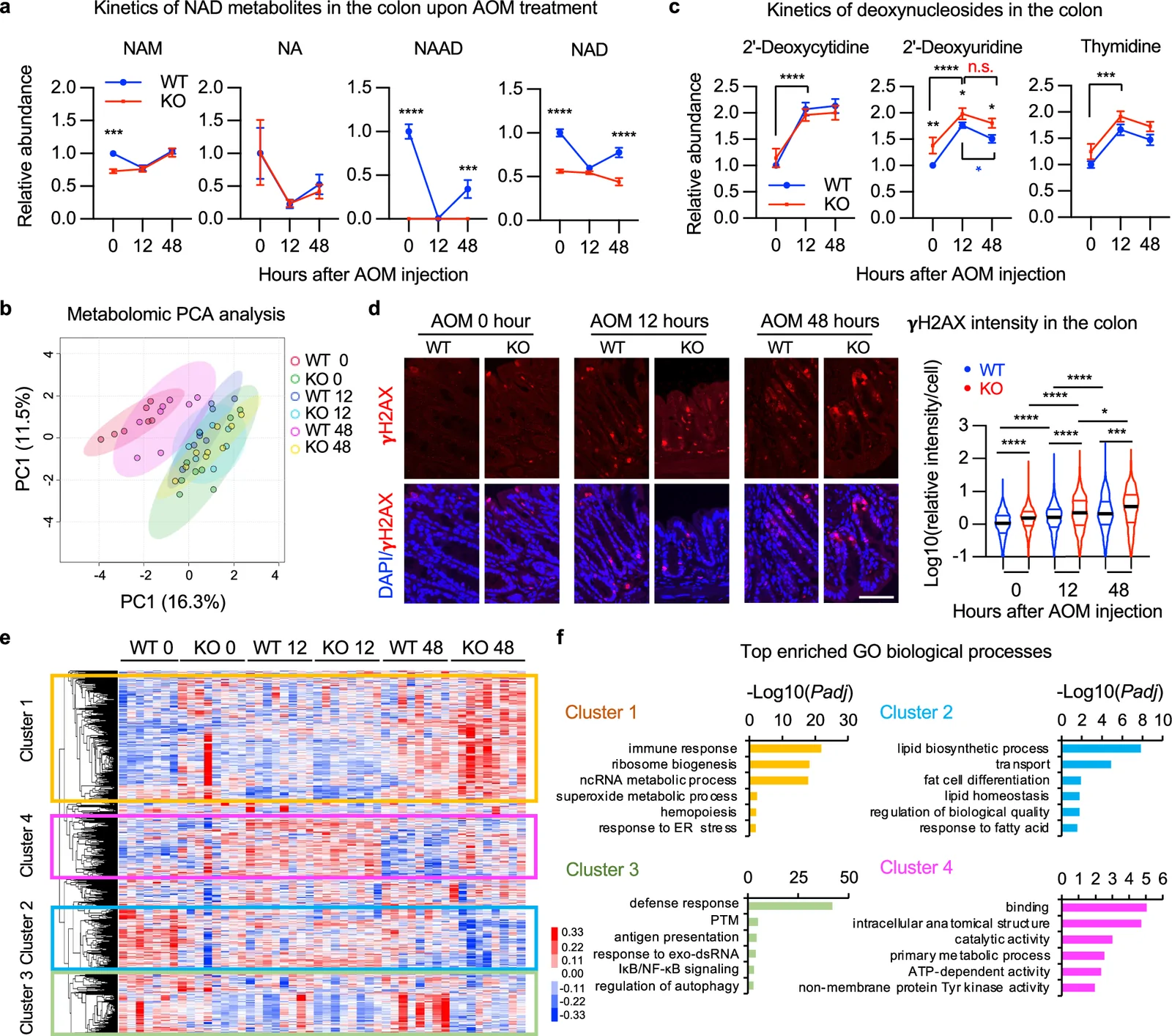
NAPRT KO mice are hypersensitive to chemically induced DNA damage and inflammation. **a**–**c** Metabolic characterization of WT and NAPRT KO mouse colons after AOM treatment. WT and NAPRT KO mice supplemented with 3 g/L NA in the drinking water were i.p. injected with one dose of 10 mg/Kg AOM, colons were then collected 12 or 48 hours post-injection as described in Methods (For 0 hour, *n* = 7 WT and 8 KO mice; For 12 hours, *n* = 8 WT and 8 KO mice; For 48 hours, *n* = 8 WT and 9 KO mice). **a** Kinetics of NAD metabolites in the colon of WT and NAPRT KO mice after AOM treatment. Indicated metabolites were analyzed by targeted LC-MS (values are normalized to the WT time 0 and expressed as mean ± SEM; two-way ANOVA). **b** PCA plot of metabolomic changes in the colon of WT and NAPRT KO mice after injection of AOM for 0, 12, or 48 hours. Colon tissues were analyzed by untargeted metabolomics using LC-MS. All 276 identified metabolites were used for the PCA plot. **c** DNA-related nucleosides in the colon of WT and NAPRT KO mice upon AOM treatment. Colon tissues were analyzed by untargeted metabolomics using LC-MS (values are normalized to the WT time 0 and expressed as mean ± SEM; 2-way ANOVA). **d** Relative γH2AX immunofluorescence intensity in the colon of WT and NAPRT KO mice treated as in (**a**) (*n* = 2200, 2778, 2752, 2819, 3071, and 2040 cells, respectively; values are normalized to the WT time 0; two-way ANOVA). Bar, 50 μm. **e** Heatmap of normalized expression levels of differentially expressed genes (DEGs) between NAPRT KO vs WT mice at different time points after AOM injection are shown as a heatmap. Transcriptomes from colonic tissues of WT and NAPRT KO mice in (**a**) were analyzed by RNA-seq. **f** Top enriched GO pathways in the highlighted clusters in (**e**). Enrichment score represents −log_10_-transformed *p*-values after adjusting for FDR false discovery rate. Source data are provided as a Source Data file.

We next directly assessed the importance of NAPRT-mediated deamidated NAD biosynthesis in DNA damage response of gut epithelium in vivo using WT and NAPRT KO mice. In unstressed conditions, NAPRT KO mice had normal gut morphology and normal expression of intestinal cell marker genes compared to WT mice (Supplementary Fig. 5a, b). We treated WT and NAPRT KO mice with azoxymethane (AOM), a chemical procarcinogen commonly used in experimental models of colon carcinogenesis. When AOM is intraperitoneally injected into rodents, DNA alkylation induced by its metabolites in the colon peaks at 6–12 hours^39^. We found that in the colon of WT mice supplemented with NA in their drinking water, this peak of DNA damage was coupled with an approximately 50% reduction in NAD, which partially recovered at 48 hours (Fig. 4a, NAD, WT). These changes of NAD were associated with a massive depletion followed by a partial restoration of deamidated NAD metabolites NA and NAAD (Fig. 4a, WT). NAPRT KO mice, on the other hand, displayed reduced basal NAD levels and experienced minimal alterations in NAD metabolism after AOM injection within the observed timeframe (Fig. 4a, KO). Additional metabolomic analysis confirmed that NAPRT KO mice had altered metabolism, particularly defective NAD metabolism, in the colon both before and 48 hours after the AOM injection compared to WT mice (Fig. 4b, Supplementary Fig. 5c, and Supplementary Data 1), and NAPRT KO mice had minimal NAD metabolic changes in response to the AOM injection (Supplementary Fig. 5c, red box).

The metabolomic analysis further revealed that AOM treatment significantly induced three deoxyribonucleosides, including 2’-deoxycytidine, 2’-deoxyuridine, and thymidine, in both WT and NAPRT KO colons (Fig. 4c and Supplementary Fig. 5d). Moreover, their induction peaked at 12 hours post-injection when DNA damage was at its highest (Fig. 4c), suggesting that DNA damage-induced DNA turnover and repair may be the sources of these nucleosides^40^. Notably, 2’-deoxyuridine levels were significantly higher in NAPRT KO colons than in WT at all time points and did not show substantial recovery at 48 hours (Fig. 4c, 2’-deoxyuridine), suggesting increased and sustained DNA damage in the absence of NAPRT. Consistently, NAPRT KO colon epithelium had a significantly elevated level of γH2AX compared to those in WT mice both before and after AOM injection (Fig. 4d). Altogether, these results suggest that deamidated NAD biosynthesis is important for efficient DNA repair in the gut.

To better understand the diminished NAD metabolic response of NAPRT KO mice to AOM-induced DNA damage at the molecular level, we carried out genome-wide transcriptomic analysis of the whole colon tissue from WT and NAPRT KO mice at 0, 12, and 48 hours after AOM injection. Despite their normal morphology, colons from NAPRT KO mice had significantly different expression of 2,502 genes compared to those from WT mice before AOM injection (0 h), among which genes mediating immune response were increased while genes involved in type I interferon-mediated signaling pathway were decreased (Supplementary Fig. 6a and Supplementary Data 2). In WT colons, AOM injection suppressed the expression of genes associated with nutrient metabolism and membrane trafficking (Supplementary Fig. 6b, c, clusters 1 and 2) while inducing those involved in immune response, ROS production, cell migration, DNA and RNA catabolism, and stress response (Supplementary Fig. 6b, c, clusters 3–6). In NAPRT KO colons, these transcriptomic changes were more pronounced compared to WT (Supplementary Fig. 6b, c). Comparisons between NAPRT KO vs WT colons at different timepoints after AOM injection further revealed that during the 48-hour timeframe after AOM injection, a cluster of 382 genes enriched in immune response pathways, including numerous immunoglobulin genes, several macrophage markers, and inflammatory chemokines/cytokines, was significantly more induced in the colon of KO mice than that in WT mice at the same time point (Cluster 1 in Fig. 4e, f, and Supplementary Data 3). This result suggests that NAPRT deficiency enhances the recruitment of various immune cells into the colon in response to AOM treatment. Further qPCR analysis showed that many of these inflammatory genes were induced at 12 hours and reduced at 48 hours after the AOM injection in the colon of WT mice, whereas their levels were elevated in the colon of KO mice before the AOM injection and remained high 48 hours after the injection (Supplementary Fig. 6d). Moreover, two clusters of genes involved in metabolic functions and interferon signaling, respectively, were significantly reduced in the colon of KO mice before and after the AOM treatment (Fig. 4e, f, and Supplementary Fig. 6d, Cluster 2 and Cluster 3). Another cluster of genes related to maintenance of cellular structure, protein quality control, and metabolism were induced in both WT and KO at 12 hours and remained elevated in KO but not in WT mice at 48 hours (Fig. 4e, f, Cluster 4). Collectively, the hypersensitivity to AOM-induced DNA damage is associated with an elevated inflammatory response yet reduced metabolic function and interferon signaling in the colon of NAPRT KO mice.

### NAPRT deficiency increases sensitivity to DSS-induced colitis in female mice

**Fig. 5:**
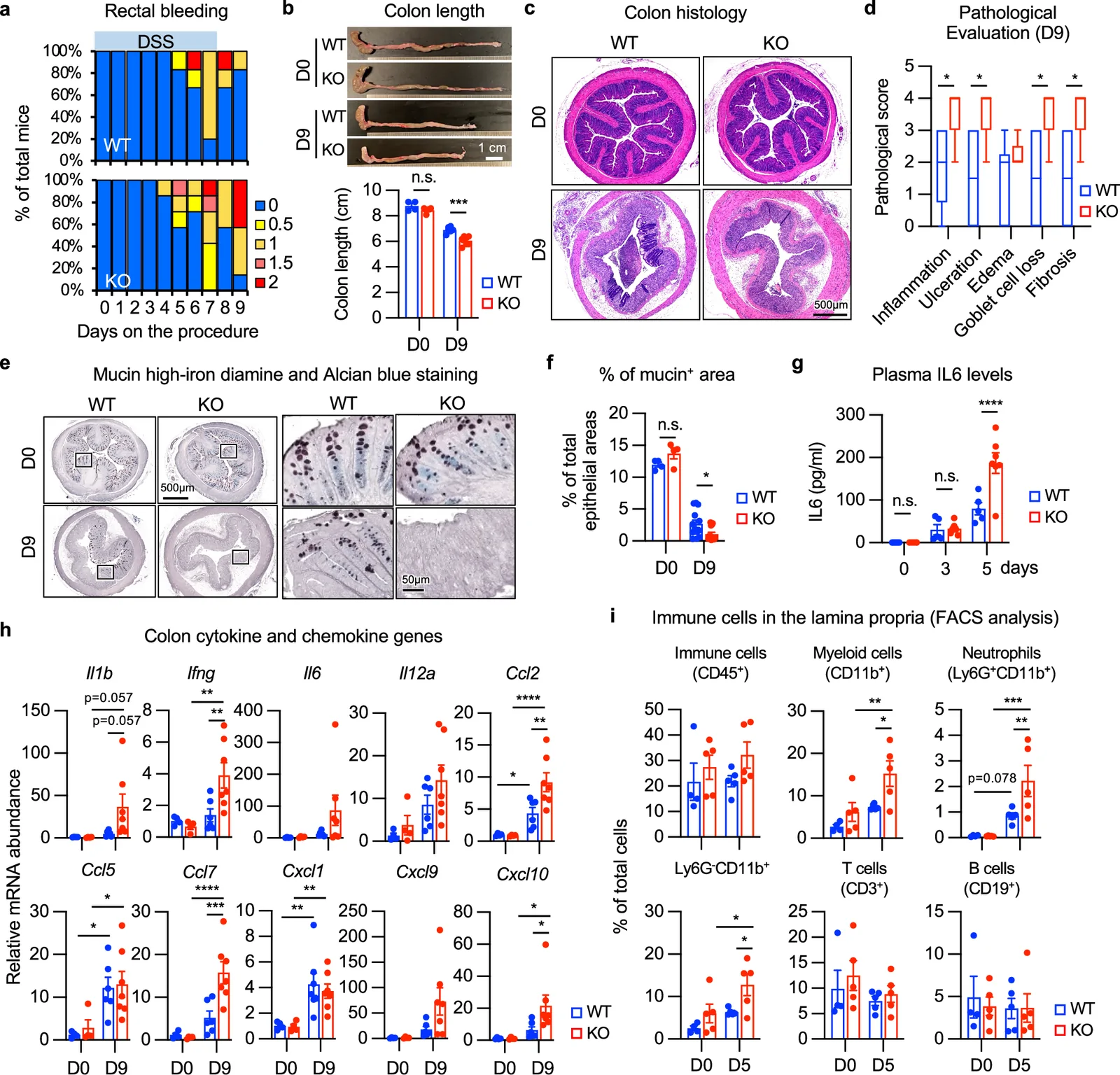
Deamidated NAD biosynthesis is important for the maintenance of gut tissue integrity in an experimental colitis model. **a**–**f** NAPRT KO mice are hypersensitive to DSS-induced colitis. Female mice were treated as described in Supplementary Fig. 7a, Protocol 1. **a** Rectal bleeding scores (*n* = 6 WT and 7 KO mice, D9). **b** Colon length (D0, n = 4 WT and 4 KO; D9, *n* = 6 WT and 7 KO mice; values are expressed as mean ± SEM; two-tailed unpaired Mann-Whitney test). **c** Representative H&E colonic images. **d** Pathological scores. H&E colonic sections from DSS-treated WT and KO mice were evaluated and scored by a board-certified veterinary pathologist (*n* = 6 WT and 5 KO mice, D9; box-and-whisker plot with the box representing the interquartile range (Q1 to Q3), a line inside indicating the median, and whiskers representing the maximum and minimum values; two-tailed unpaired Mann-Whitney test). **e** Representative mucin high-iron diamine and Alcian blue staining colonic images of WT and NAPRT KO mice after DSS treatment and recovery. **f** Percentage of mucin^+^ area in the colon quantified by Fiji (D0, *n* = 4 WT and 4 KO; D9, *n* = 13 WT and 14 KO mice from two independent experiments; values are expressed as mean ± SEM; two-tailed unpaired Mann-Whitney test). **g** Plasma IL-6 levels after 2.5% DSS treatment for 3 or 5 days (D0, *n* = 5 WT and 4 KO; D3, *n* = 5 WT and 7 KO; and D5, *n* = 5 WT and 7 KO; values are expressed as mean ± SEM; two-way ANOVA). **h** mRNA levels of proinflammatory cytokine and chemokine genes in the colon before (D0) and after (D9) DSS treatment (D0, *n* = 4 WT and 4 KO; D9, *n* = 6 WT and 7 KO mice; values are normalized to the regular WT controls and expressed as mean ± SEM; two-way ANOVA). **i** Immune cells in the lamina propria of the colon after 5-day treatment with 2.5% DSS. The abundance of indicated immune cells was analyzed by FACS (D0, *n* = 4 WT and 5 KO; D5, *n* = 5 WT and 5 KO mice; values are expressed as mean ± SEM; two-way ANOVA). Source data are provided as a Source Data file.

To directly test whether NAPRT deficiency may indeed enhance chemically induced inflammation in the gut, we challenged WT and NAPRT KO female mice with dextran sulfate sodium (DSS)-induced colitis, a well-established experimental model of IBD characterized by compromised mucosal barrier function, infiltration of inflammatory immune cells into the lamina propria, epithelial ulceration, and focal crypt damage^41^. In a DSS-induced colitis protocol where mice were first challenged with 2.5% DSS in their drinking water for 7 days and then allowed to recover with regular drinking water for 2 days (Supplementary Fig. 7a, Protocol 1), NAPRT KO mice experienced earlier and more severe rectal bleeding during the 7-day DSS treatment period and reduced recovery during the 2-day regular water recovery period (Fig. 5a). Accordingly, at the end of the procedure, NAPRT KO mice had aggravated colonic shortening (Fig. 5b) and more severe colonic epithelial tissue damage (Fig. 5c, d), including loss of mucin-positive goblet cells (Fig. 5e, f). These elevated measures of the morphological and pathological damage in the colon of NAPRT KO mice were coupled with elevated levels of IL6 cytokine in the plasma and increased induction of proinflammatory cytokine/chemokine genes in the colon tissues (Fig. 5g, h). In another DSS-colitis protocol in which mice were treated with 2.5% DSS together with 3 g/L NAM in drinking water for 7 days without recovery (Supplementary Fig. 7a, Protocol 2), we also observed an increased sensitivity of NAPRT KO mice to DSS-induced rectal bleeding, colonic shortening, and gut epithelial damage (Supplementary Fig. 7b–e). Furthermore, when analyzed at the transcriptomic level, DSS treatment significantly repressed the expression of a cluster of genes involved in epithelial transporter activities in the colon of WT mice and such repression was significantly stronger in the colon of NAPRT KO mice (Supplementary Fig. 7f, g, Cluster 1), suggesting more severe damage of colon epithelial function in NAPRT KO mice. On the other hand, DSS-induced expression of proinflammatory genes was markedly higher in the colon of NAPRT KO mice than in WT mice (Supplementary Fig. 7f, g, Cluster 2). The increased induction of several proinflammatory cytokines and chemokines was further confirmed by qPCR (Supplementary Fig. 7h). Therefore, NAPRT deficiency sensitizes mice to chemically induced tissue damage and inflammation in the gut.

To better understand the potential mechanisms through which NAD depletion leads to increased inflammation in the DSS colitis in NAPRT KO mice, we analyzed the immune cells in the lamina propria of the colon and blood before and after five-day treatment of DSS by FACS analysis. Consistent with the literature reports^41^, the major immune cells responding to DSS-induced colitis were innate immune cells (Fig. 5i and Supplementary Fig. 7i). In WT mice, five-day treatment with DSS increased the abundance of blood myeloid cells, primarily neutrophils (Supplementary Fig. 7i, WT). This increase was coupled with an elevated infiltration of neutrophils into the colonic lamina propria (from 0.07% to 0.86%) (Fig. 5i, WT). Deletion of NAPRT did not significantly affect immune cell abundance in either blood or colonic lamina propria under the basal condition (Fig. 5i and Supplementary Fig. 7i, D0). However, DSS-induced infiltration of neutrophils into the colonic lamina propria was significantly enhanced (from 0.06% to 2.22%) in these mice (Fig. 5i, KO), whereas their blood neutrophil abundance was slightly lower than that of WT mice after DSS treatment (Supplementary Fig. 7i, D5). DSS-treatment also significantly increased infiltrated Ly6G-negative myeloid cells (Fig. 5i, Ly6G^-^CD11b^+^), which include macrophages, monocytes, and dendritic cells. Together with our observations in Fig. 5a–h, these findings suggest that NAPRT deficiency increases the recruitment of myeloid cells, including neutrophils, into colonic lamina propria in response to DSS-induced epithelial damage.

**Fig. 6:**
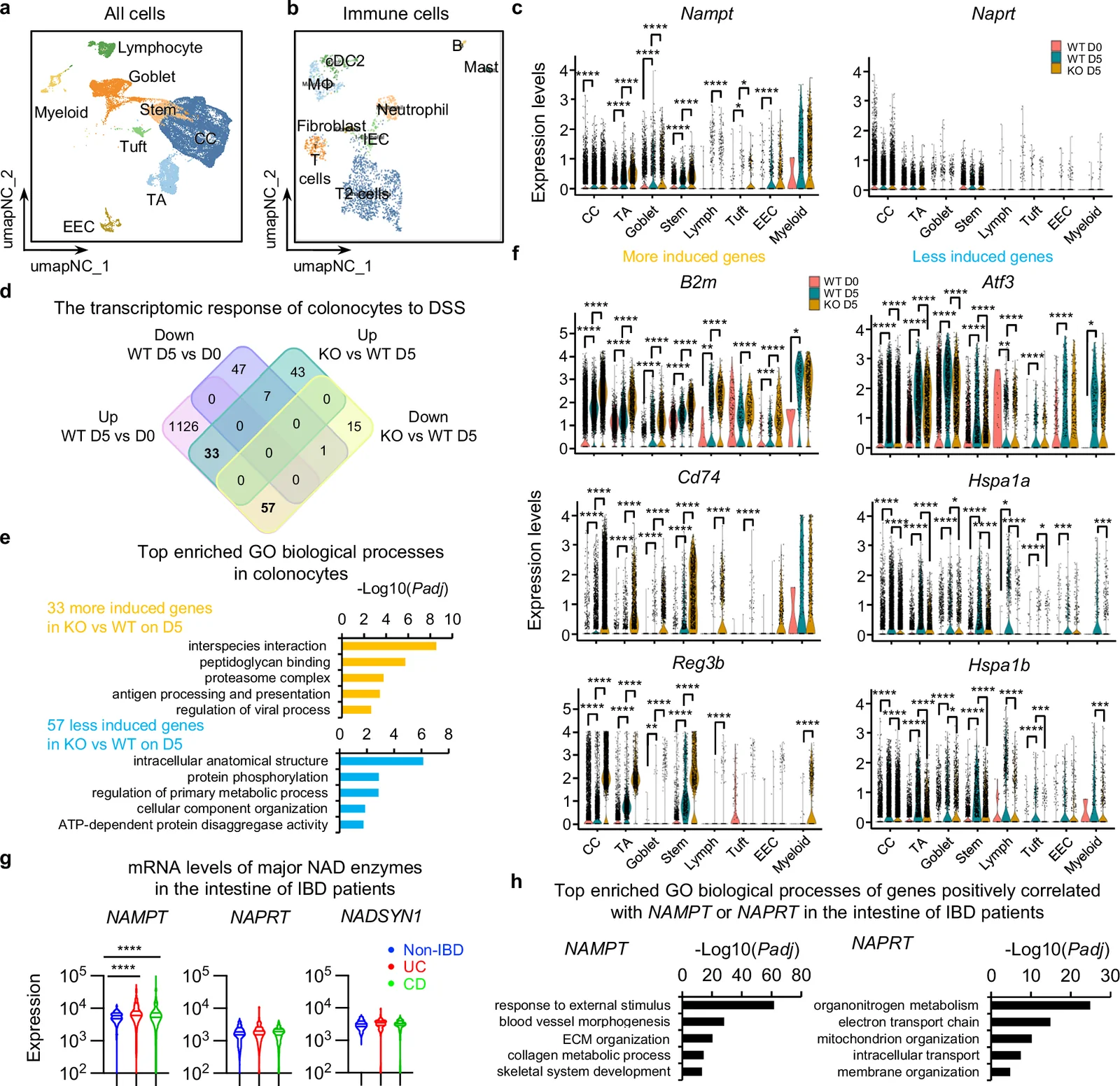
Defective deamidated NAD biosynthesis is associated with increased anti-microbial defense response and reduced maintenance of tissue integrity in colitis. **a** Annotated cell types in the colons of WT and NAPRT KO mice treated with or without DSS. WT and NAPRT KO mice were treated with or without 2.5% DSS for 5 days. Pooled single cells isolated from the colons were analyzed by scRNA-seq analysis as described in Methods (*n* = 3 mice/group, with 30,924 total single cells and 2,377 single immune cells). CC colonocytes, EEC enteroendrocrine cells, TA transit-amplifying cells. **b** Annotated immune cell populations. Two immune cell clusters, lymphocyte and myeloid, were further clustered and annotated. MΦ, macrophages; T2 cells, a subcluster of T cells expressing some exhaustion markers; IEC, intestinal epithelial cells. **c** *Nampt*, but not *Naprt*, is significantly induced by DSS treatment in most cell types (two-sided Wilcoxon test and *p*-values were adjusted using the Holm–Bonferroni correction). **d** Venn-diagram analysis of DSS-induced genes in WT colonocytes (WT D5 vs D0) vs NAPRT deletion altered genes in colonocytes after DSS treatment (KO vs WT D5). **e** Top enriched GO biological processes in 33 more induced genes and 57 less induced genes in KO vs WT colonocytes after DSS treatment. Enrichment score represents −log_10_-transformed *p*-values after adjustment for the FDR false discovery rate. **f** Violin plots of top altered genes in two categories in **e** (two-sided Wilcoxon test and p-values were adjusted using the Holm–Bonferroni correction). **g** The expression of *NAMPT* but not *NAPRT* or *NADSYN* is significantly elevated in the intestine of IBD patients. Intestinal RNA-seq dataset from human IBD patients and non-IBD controls from^42^ were analyzed (Kruskal-Wallis test). **h** The top enriched biological processes of genes positively correlated with *NAMPT* (left) or *NAPRT* (right) in the intestine of human IBD patients. Intestinal RNA-seq datasets from human IBD patients and non-IBD controls from^42^ were analyzed. Enrichment score represents −log_10_-transformed *p*-values after adjustment for the FDR false discovery rate. Source data are provided as a Source Data file.

To further understand how deletion of NAPRT increases the recruitment of myeloid cells upon DSS-induced colitis at the molecular level, we analyzed transcriptomics of all colonic cell types before and after five-day treatment of DSS at the single cell level by scRNA-seq. Based on the individual cell transcriptome and the expression patterns of known marker genes (Supplementary Fig. 8a), the colonic cells could be categorized into 8 functional groups, including 6 epithelial cell groups and 2 immune cell groups (Fig. 6a and Supplementary Data 4). The two immune cell groups could be further subclustered into 9 clusters, including 7 immune cell types and two contaminating non-immune cell types (IECs and fibroblasts) (Fig. 6b, Supplementary Fig. 8b, and Supplementary Data 4). Consistent with the FACS results in Fig. 5i, DSS-treated KO mice had increased abundance of neutrophils in the colon compared to DSS-treated WT mice (Supplementary Data 4, Neutrophils). In contrast, the abundance of colonic T cells, particularly a subcluster of T cells expressing some exhaustion markers (Supplementary Fig. 8b, T2), was markedly reduced in KO mice (Supplementary Data 4, T and T2 cells). Interestingly, in the majority of cell types, the expression of *Nampt* was significantly induced by DSS treatment, whereas the expression of *Naprt* was not significantly affected (Fig. 6c and Supplementary Fig. 8c, WT D5 vs WT D0), suggesting the differential functions of these two NAD biosynthesis pathways in response to DSS-induced colitis. Transcriptomic analysis of colonocytes, the cell type with the highest expression level of *Naprt* (Fig. 6c, CC), showed that DSS treatment induced the expression of genes associated with membrane trafficking, G-protein coupled cell signaling, and adherens junctions, but reduced the expression of genes involved in lipid transport, digestive system, and lipid metabolism (Supplementary Fig. 8d). Venn-diagram analysis further revealed that among the genes induced by DSS in colonocytes of WT mice, deletion of NAPRT further enhanced the expression of genes involved in the defense response, particularly those related to antigen processing and presentation, but suppressed the induction of genes responsible for intracellular structure maintenance and protein quality control (Fig. 6d, e). These key transcriptomic changes were consistently observed across all annotated cell types, including both gut epithelial cells and immune cells (Fig. 6f and Supplementary Fig. 8e), suggesting that the enhanced intestinal inflammation observed in DSS-treated KO mice is associated with increased anti-microbial defense response and reduced maintenance of tissue integrity. Taken together, in agreement with our pathological, biochemical, and qPCR evaluations in Fig. 5a–h, our FACS and scRNA-seq analyses indicate that inactivation of the deamidated NAD biosynthesis in NAPRT-deficient colon leads to reduced maintenance of tissue integrity and elevated anti-microbial response upon DSS challenge, which enhances recruitment of myeloid cells (particularly neutrophils) and induces intestinal inflammation.

To further explore the clinical significance of deamidated NAD biosynthesis in the regulation of gut inflammation and tissue integrity, we analyzed the expression of two key deamidated NAD metabolic enzymes, NAPRT and NADSYN1, in a publicly available RNA-seq dataset from the intestine of human IBD patients and non-IBD controls^42^. Consistent with previous reports^11,43^, the mRNA level of *NAMPT* was significantly elevated in the intestine, including the colon, of both ulcerative colitis (UC) and Crohn’s disease (CD) patients (Fig. 6g and Supplementary Fig. 8f, *NAMPT*), and *NAMPT* expression was strongly correlated with that of genes involved in response to extracellular stimuli, particularly many inflammatory and immune cell genes (*SOCS3*, *CD274*, *LILRA5*, and *S100A9*) (Fig. 6h, *NAMPT*, Supplementary Fig. 8g, and Supplementary Data 5). In contrast, neither *NAPRT* nor *NADSYN1* were significantly altered in the intestine of IBD patients in this study (Fig. 6h and Supplementary Fig. 8f). Moreover, the mRNA level of *NAPRT* was positively correlated with that of genes involved in metabolism, mitochondrial function, and nutrient transport regardless of the patient IBD status (Fig. 6h, *NAPRT*, Supplementary Fig. 8h, and Supplementary Data 5). Collectively, our findings indicate that NAPRT is important for metabolism and overall functions of gut epithelial cells in both mice and humans.

### NAPRT deficiency promotes tumorigenesis

**Fig. 7:**
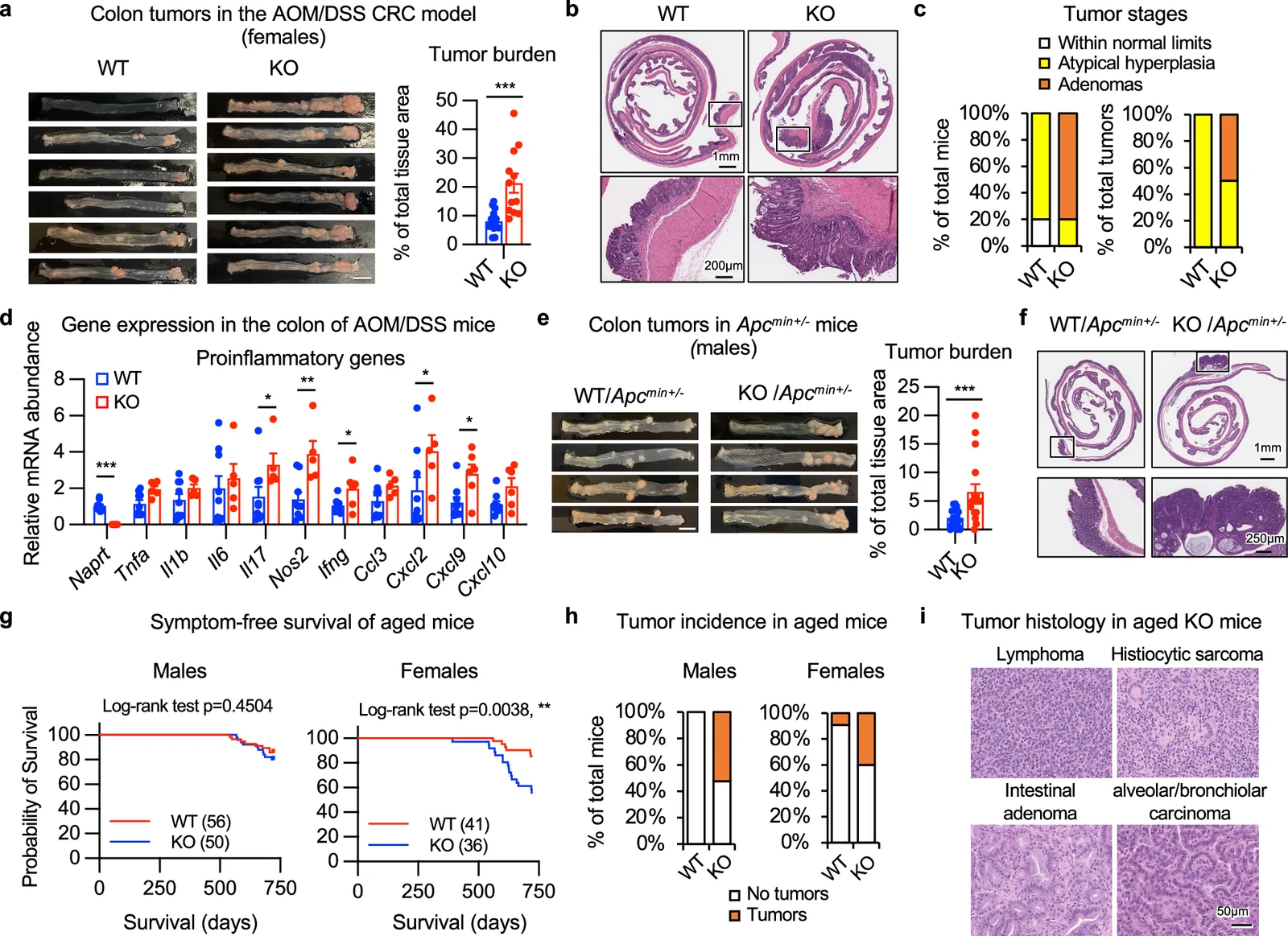
NAPRT deficiency promotes tumorigenesis in mice. **a** Tumor burden in WT and NAPRT KO female mice in an AOM/DSS CRC model. Colonic tumor burden was quantified in Fiji (*n* = 14 WT and 12 KO mice, values are expressed as mean ± SEM; two-tailed unpaired Mann-Whitney test). **b** Representative H&E colonic images from WT and NAPRT KO mice at the end of the AOM/DSS CRC procedure. **c** Stages of colorectal tumors developed in WT and NAPRT KO mice in the AOM/DSS CRC model. H&E colonic sections were evaluated by a board-certified veterinary pathologist. Left, percentage of mice bearing colorectal tumors at different stages (*n* = 5 WT and 5 KO mice). Right, percentage of colorectal tumors at different stages (*n* = 6 tumors from WT mice and 12 tumors from KO mice). **d** mRNA levels of proinflammatory genes in the colon of AOM/DSS-treated mice analyzed by qPCR (*n* = 9 WT and 5 KO mice, values are expressed as mean ± SEM and normalized to those of the Lamin A gene; multiple unpaired Mann-Whitney tests). **e** Colon tumor formation in WT and NAPRT KO male mice in the *Apc*^*min+/-*^ background analyzed at the age of 16 weeks. Colonic tumor burden was quantified in Fiji (*n* = 20 WT males, 17 KO males, values are expressed as mean ± SEM; two-tailed unpaired Mann-Whitney test). **f** Representative H&E colonic images of WT and NAPRT KO mice on the *Apc*^*min+/-*^ background at the age of 16 weeks. **g** Symptom-free survival of aged male and female WT and NAPRT KO mice (*n* = 56 WT and 50 KO males; 41 WT and 36 KO females at the beginning of the experiment). **h** Spontaneous tumor incidence in various tissues of WT and NAPRT KO mice analyzed at the ages of 22–24 months (*n* = 12 WT and 19 KO males; 11 WT and 5 KO females). **i** Representative histological images of tumors from aged KO mice. All tumors from aged male mice are shown in Supplementary Fig. 9g, and H&E slides of all tumors were evaluated by a board-certified pathologist. Source data are provided as a Source Data file.

The increased sensitivity of NAPRT KO mice to chemically induced DNA damage and inflammation in the colon raises the possibility that they may have an enhanced propensity to tumorigenesis in this tissue. To test this possibility, we challenged WT and NAPRT KO female mice with an AOM/DSS-induced inflammation-associated colorectal cancer (CRC) model, in which mice were first injected with 10 mg/Kg AOM to induce DNA damage and then treated with 2 % DSS in drinking water for 7-8 days to induce inflammation and tissue damage (Supplementary Fig. 9a). In support of our hypothesis, when analyzed at the end of this procedure (D90 after AOM injection and around 11 weeks after DSS treatment), NAPRT KO mice developed more and bigger colorectal tumors, thereby significantly increasing tumor burden in the colon (Fig. 7a). Further pathological evaluation indicated that colorectal tumors developed in NAPRT KO mice were at the more advanced adenoma stage compared with those in WT mice (Fig. 7b and Supplementary Fig. 9b). More specifically, 80% NAPRT KO mice had colon tumors at the adenoma stage whereas WT mice had none (Fig. 7c, left). Moreover, while all tumors developed in WT mice were at a less advanced atypical hyperplasia stage, half of colorectal tumors in NAPRT KO mice were adenomas (Fig. 7c, right). Furthermore, the colon tissue from NAPRT KO mice displayed increased expression of multiple proinflammatory genes and immune cell markers at the end of the AOM/DSS CRC procedure (Fig. 7d and Supplementary Fig. 9c).

To confirm the increased susceptibility of NAPRT KO mice to intestinal tumorigenesis in an additional model, we bred WT and NAPRT KO mice into the *Apc*^*min+/-*^ background, a genetic intestinal tumor model that develops spontaneous intestinal adenomas driven by dysregulated WNT/β-catenin pathway^44,45^. When analyzed at the age of 16 weeks, NAPRT KO male but not female mice had increased colon tumor burden on this *Apc*^*min+/-*^ background (Fig. 7e, f, and Supplementary Fig. 9d, e). Both male and female mice displayed expected defects in deamidated NAD biosynthesis (Supplementary Fig. 9f). Finally, monitoring the mortality of WT and NAPRT KO male and female mice during the first 24 months of age showed that female but not male NAPRT KO mice had significantly reduced symptom-free survival compared with age-matched WT females (Fig. 7g). Among the 16 KO females removed/lost from the aging cohort, five were removed due to visible large abdominal mass or rectal prolapse, whereas none of the six removed/lost WT females displayed these tumor-related symptoms, suggesting that NAPRT KO mice may have increased risk of developing spontaneous tumors. Indeed, detailed analysis of two independent cohorts of 22 to 24-month-old male and female mice revealed that 10 out of 19 male and 2 out of 5 female aged NAPRT KO mice developed spontaneous tumors in various organs, including gut, gut-associated tissues, liver, and lung (Fig. 7h, i, and Supplementary Fig. 9g). In contrast, none of the 12 male and only one out of 11 female aged WT mice carried a tumor (Fig. 7h). Further histopathological evaluation by a board-certified pathologist indicated that the majority of tumors developed in aged NAPRT KO mice were lymphomas and sarcomas (Supplementary Fig. 9g), suggesting that the tumor suppressor function of NAPRT is not limited to epithelial cancers.

**Fig. 8:**
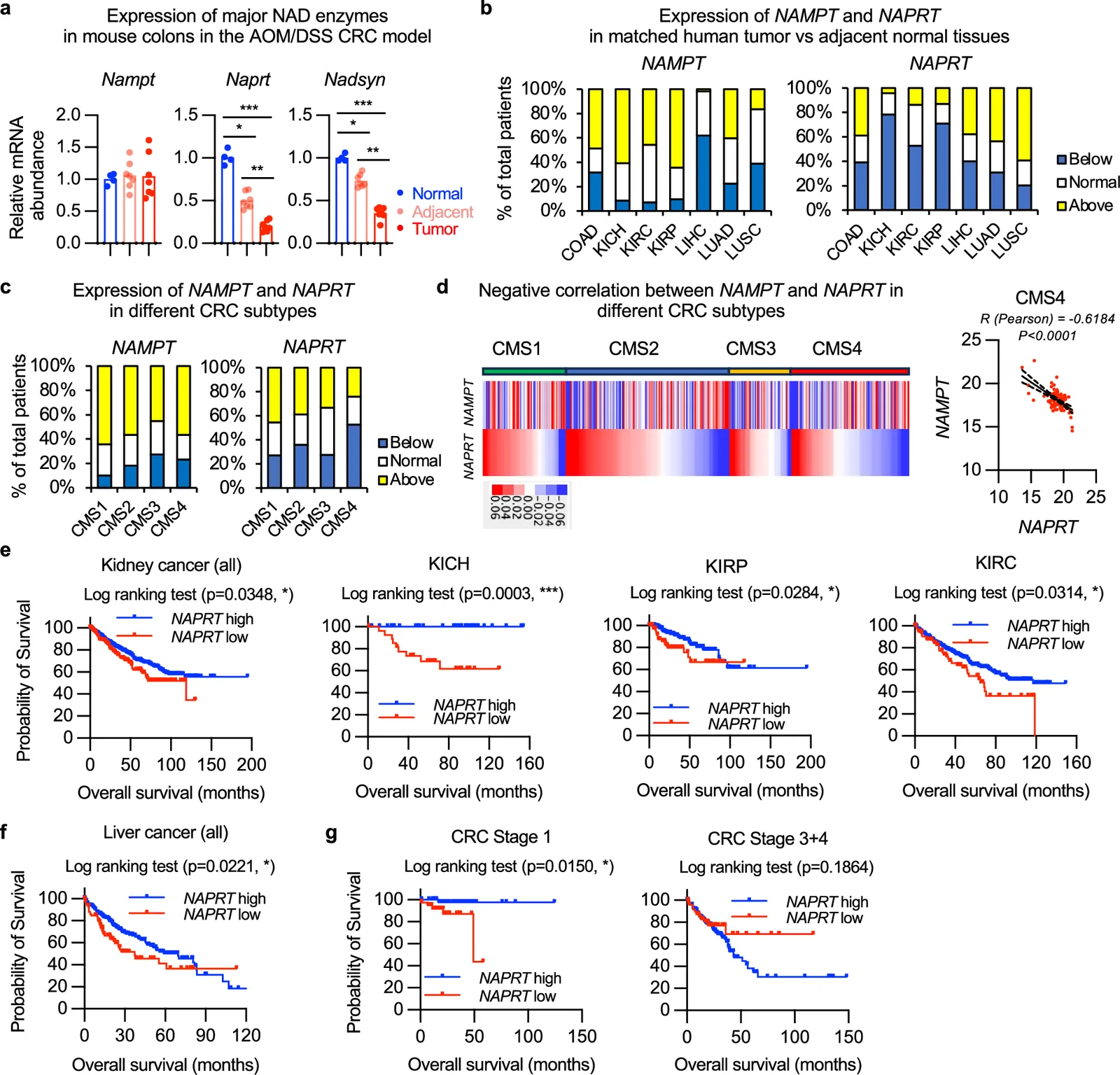
Low *NAPRT* is associated with increased susceptibility and poor prognosis in several human cancer types. **a** The expression of *Naprt* and *Nadsyn* but not *Nampt* is significantly reduced in the AOM/DSS-induced CRC tumors (Tumor) and tumor-adjacent normal colon tissues (Adjacent) compared to normal colon tissues from untreated WT mice (Normal). mRNA levels of indicated genes were analyzed by qPCR (n = 4 normal, 7 Adjacent, and 7 Tumor from individual mice, Kruskal-Wallis test). **b** A significant fraction of human cancer patients has reduced expression of *NAPRT*. mRNA levels of *NAMPT* and *NAPRT* in different human cancer types were stratified into normal, above normal, and below normal based on their expression in matched normal tissue samples as described in Methods (*n* = 41, 23, 72, 31, 50, 62, and 49 pairs for COAD, KICH, KIRC, KIRP, LIHC, LUAD, and LUSC respectively). **c** The expression of *NAMPT* and *NAPRT* in different subtypes of human colorectal cancer (CRC) patients (*n* = 70 CMS1, 136 CMS2, 51 CMS3, and 99 CMS4). CMS subtypes were characterized in ref. ^46^. CMS1 (microsatellite instability immune, 14%), hypermutated, microsatellite unstable and strong immune activation; CMS2 (canonical, 37%), epithelial, marked WNT and MYC signaling activation; CMS3 (metabolic, 13%), epithelial and evident metabolic dysregulation; CMS4 (mesenchymal, 23%), prominent transforming growth factor β activation, stromal invasion and angiogenesis. **d** Negative correlation between *NAMPT* and *NAPRT* in different subtypes of CRC (*n* = 70 CMS1, 136 CMS2, 51 CMS3, and 99 CMS4). **e**–**g** Low expression of *NAPRT* is associated with poor prognosis in human kidney cancers, liver cancer, and Stage 1 CRC. TCGA datasets from Cbioportal (https://www.cbioportal.org/) were analyzed (*n* = 661 *NAPRT* high and 192 *NAPRT* low in all kidney cancer; *n* = 37 *NAPRT* high and 28 *NAPRT* low in KICH; *n* = 199 *NAPRT* high and 83 *NAPRT* low in KIRP; *n* = 432 *NAPRT* high and 76 *NAPRT* low in KIRC; *n* = 275 *NAPRT* high and 90 *NAPRT* low in all liver cancer; *n* = 63 *NAPRT* high and 40 *NAPRT* low in CRC stage 1; *n* = 196 *NAPRT* high and 50 *NAPRT* low in CRC stage 3 + 4). Source data are provided as a Source Data file.

*NAPRT* is found to be frequently amplified and/or overexpressed in various human cancer types, and its knockdown slowed the growth of xenograft tumors^9,17^, suggesting a tumor-promoting role of this gene. However, our observations in NAPRT KO mice suggest that although high levels of NAD may promote tumor progression, maintaining an endogenous level of the deamidated NAD biosynthesis is critical for efficient DNA repair and subsequent suppression of tumorigenesis. In support of this hypothesis, AOM/DSS-induced tumorigenesis in WT mice was coupled with reduced expression of key deamidated NAD biosynthesis genes (Fig. 8a). Compared to normal colon tissues from age- and gender-matched untreated mice, tumor adjacent colon tissues from AOM/DSS-treated WT mice had significantly reduced expression of both *Naprt* and *Nadsyn* but not *Nampt* (Fig. 8a, Adjacent vs Normal). The mRNA levels of these two key deamidated NAD biosynthesis enzymes were further reduced in AOM/DSS-induced tumors (Fig. 8a, Tumor vs Adjacent). Moreover, analysis of TCGA datasets showed that the mRNA levels of *NAPRT* are reduced in a significant proportion of tumors from seven analyzed human cancer types, including colon adenocarcinoma (COAD), all three types of kidney cancers, kidney chromophobe (KICH), kidney renal clear cell carcinoma (KIRC), kidney renal papillary cell carcinoma (KIRP), liver hepatocellular carcinoma (LIHC), as well as two types of lung cancers, lung adenocarcinoma (LUAD) and lung squamous cell carcinoma (LUSC) (Fig. 8b). Similar results were observed when all primary tumor samples with expression values were analyzed (Supplementary Data 6).

To gain deeper insight into the role of NAMPT and NAPRT in colonic tumorigenesis, we stratified the colorectal cancer (CRC) data in Fig. 8b based on CMS subtypes^46^. As shown in Fig. 8c, all four CRC subtypes had more tumors with an elevated expression of *NAMPT* than those with low expression of this gene. Notably, CMS1, the subtype characterized by increased immune infiltration and strong activation of immune evasion pathways^46^, had the highest proportion of *NAMPT*-high tumors. On the other hand, CMS4, the subtype that shows upregulation of epithelial mesenchymal transition (EMT) and activation of transforming growth factor β (TGF β)^46^, had the highest proportion of *NAPRT*-low tumors (Fig. 8c, *NAPRT*). These findings were consistent with our observations that NAMPT was enriched in immune cells whereas NAPRT was predominantly expressed in epithelial cells (Figs. 1a, b, 6c, and Supplementary Figs. 2b, c, 8c). Intriguingly, the expression levels of *NAMPT* and *NAPRT* were negatively correlated in CRC, especially in the CMS4 subtype that displays worse overall and relapse-free survival compared to other subtypes^46^ (Fig. 8d), highlighting the subtype-specific roles of these two NAD biosynthesis pathways in intestinal tumorigenesis. In colorectal polyps, alterations in the expression of *NAMPT* and *NAPRT* also showed distinct patterns. Notably, the mRNA levels of *NAMPT* were significantly reduced in colorectal polyps compared to normal colonic tissues, whereas the mRNA levels of *NAPRT* remained largely unchanged (Supplementary Fig. 9h).

Further analysis of TCGA PanCancer Altas revealed that low expression of *NAPRT* in liver and kidney cancers, particularly in KICH, is significantly associated with reduced patient survival (Fig. 8e, f). Interestingly, the impact of *NAPRT* expression on the prognosis of CRC was stage dependent. Stage 1, but not stage 3 and 4, CRC patients with low *NAPRT* levels displayed worse prognosis compared to stage-matched patients with high *NAPRT* expression (Fig. 8g). Collectively, our findings indicate that NAPRT-mediated deamidated NAD biosynthesis is important for normal tissue integrity and tumor suppression in response to environmental stress.

## Discussion

The evolutionarily ancient deamidated NAD salvage from NA is the only pathway to recycle NAM produced by NAD-consuming enzymes in the majority of lower organisms^47^. In vertebrates, the loss of a nicotinamidase activity that converts NAM to NA is accompanied by the evolvement of a shorter NAMPT-mediated amidated NAD salvage pathway, allowing them to recycle NAM to NAD independently of the deamidated pathway. As a result, the deamidated NAD biosynthesis is considered a minor pathway in mammalian cells. In the present study, by employing several intestinal disease models in a newly generated NAPRT mutant mouse strain, together with extensive in vitro and in vivo mechanistic analyses and clinical database mining, we show that deamidated NAD biosynthesis is functionally important for the gut epithelial stress response and whole-body tumor suppression. We provide evidence that in the gut, this metabolic pathway is selectively active in the gut epithelium, where it functions to maintain a suitable NAD pool to support efficient DNA repair, improve tissue recovery, and suppress tumorigenesis.

Both amidated NAD salvage and deamidated NAD biosynthesis are active in a wide array of tissues in mammals (Fig. 3b)^2,48,49^. Interestingly, several lines of evidence in the present study suggest that these two branches of NAD biosynthesis have distinct functions even in the same tissue. For instance, our scRNA-seq analysis showed that in both small intestine and colon, *NAMPT* and *NAPRT* have distinct cell-type specific expression patterns, with *NAMPT* ubiquitously expressed in all cell types, particularly in gut immune cells, whereas *NAPRT* is relatively selectively enriched in the epithelial compartments (Fig. 1a and Supplementary Fig. 1b). Consistently, in the setting of IBD, *NAMPT* is significantly induced in the intestine along with genes involved in inflammatory and immune activation, whereas *NAPRT* is positively correlated with genes important for metabolism and epithelial functions independent of the patient IBD status (Fig. 6). Moreover, in CRC, *NAMPT* and *NAPRT* expression levels were negatively correlated (Fig. 8d). Specifically, CMS1, the CRC subtype marked by increased immune infiltration (CMS1), had the highest proportion of *NAMPT*-high tumors, while CMS4, the CRC subtype associated with enhanced EMT, contained the highest proportion of *NAPRT*-low tumors (Fig. 8c). Therefore, it is likely that NAMPT-mediated amidated NAD salvage is important to fuel proinflammatory response mainly in immune cells, while NAPRT-mediated deamidated NAD biosynthesis is critical for cell-autonomous maintenance of gut epithelial metabolism and overall functions. Furthermore, in the setting of genotoxic stress, the deamidated NAD biosynthesis was important to maintain a high NAD pool for more efficient PARP-mediated DNA repair but was less critical for cell survival compared to the amidated synthesis (Fig. 2). Consequently, NAPRT-deficient gut epithelial cells carrying DNA mutations were more likely to survive the genotoxic stress than those with defective NAMPT activity. Finally, our data indicate that a physiological level of NAPRT is tumor-suppressing in mice (Fig. 7) and is associated with favorable prognosis in several human cancer types (Fig. 8). NAMPT, on the other hand, is generally an oncogene. Future investigation of the mechanisms underlying the functional differences of these two NAD salvage pathways will be of great value.

An intriguing implication of the present study is that the impact of NAPRT and deamidated NAD biosynthesis on cancer development is U-shaped. On the one hand, NAPRT is amplified and/or overexpressed in many human cancer types^9,17^. Particularly, it has been previously shown that cancers arising from tissues with a high expression of NAPRT have a high frequency of NAPRT amplification with a subset of tumors completely and irreversibly dependent on NAPRT for survival^9^. Inhibition of the activity of NAPRT, therefore, has been proposed as a strategy for anti-cancer treatment for established cancer^9,17,50^. On the other hand, by using a newly developed genetic NAPRT KO model, we showed in this study that maintaining a normal endogenous level of *NAPRT* activity, especially in tissues with normally high expression of this gene, is important for suppression of tumor initiation (Fig. 6). Given our finding that NAPRT inactivation led to higher levels of DNA damage without a corresponding increase in the elimination of damaged cells in both colon cancer cells and normal colon organoids (Figs. 2 and 3), it is possible that the tumor-promoting effect of NAPRT inactivation is at least partially explained by defective DNA repair and an increased mutational burden during the early stages of tumor initiation. In addition, the anti-inflammatory activity of NAPRT (Fig. 5) may also constrain tumor growth, which is frequently fueled by chronic inflammation^51^. In fact, our observations that NAPRT deficiency increased tumor initiation in mice, together with previous reports on the anti-tumor growth effects of NAPRT inhibition in established cancer cell lines and xenografts reveal an intriguing stage-specific role of NAD in cancer: suppressing tumor initiation at early stages while supporting tumor growth and progression at later stages. These findings could offer valuable guidance for the development of future NAD-based strategies in cancer prevention and treatment. Our results further suggest that, depending on the expression level of NAPRT and the stage of a given tumor, both NAPRT inhibitors and activators or substrates could be used for cancer therapy or prevention once tailored to the individual patient.

The importance of NAD in supporting DNA repair and maintaining genome stability has been well-established in the literature (reviewed in refs. ^52,53^). Consistently, pathological conditions that lead to NAD depletion, such as aging, high-fat diet feeding, and oncogenic gene expression, have been reported to increase DNA damage and promote tumorigenesis through multiple NAD-dependent mechanisms, including PARP inactivation, sirtuin inhibition, and reduced dNTP synthesis. For instance, a seminal study by Tummala et al. showed that in hepatocytes, suppression of the de novo NAD biosynthesis pathway by expression of an oncogene unconventional prefoldin RPB5 interactor (URI) leads to reduced cellular NAD levels and increased DNA damage at early stages of tumorigenesis possibly by inhibition of PARP^54^. Restoration of hepatic NAD pool with NR supplementation prevents DNA damage and tumor formation^54^. In a following-up study, Gomes et al. reported that URI-induced inhibition of de novo NAD biosynthesis and subsequent DNA damage also trigger hepatic T cell infiltration and increase IL-17A production, which further promotes non-alcoholic steatohepatitis and hepatocellular carcinoma^55^. These defects could also be abolished by dietary supplementation of NR^55^. In the present study, by utilizing a genetic NAPRT KO model that enabled us to investigate the role of the Preiss-Handler arm of the deamidated NAD biosynthesis during tumor initiation, we demonstrated that in the gut epithelium, deficiency of this pathway impairs PARP activity and compromises DNA repair, increasing susceptibility to inflammation and tumorigenesis. Therefore, endogenous NAPRT is a tumor suppressor rather than a tumor promoter. Future studies employing tissue-specific NAPRT knockout models are needed to further elucidate the role of NAPRT in different cell types and at different stages along the carcinogenesis process.

In summary, our study establishes NAPRT-mediated deamidated NAD biosynthesis as a key factor in maintaining NAD homeostasis, supporting DNA repair, and inhibiting inflammation in gut epithelial cells. Our findings further highlight the importance of this environment-sensitive pathway in suppression of tumorigenesis. Enhancing this pathway through NAPRT activation, dietary supplementation and/or probiotics could be a promising approach to preserve or improve gut health and protect against various human cancers.

## Methods

### Ethics statement

All animal experiments in this study were approved by the NIEHS/NIH Animal Care and Use Committee under the Animal Study Proposal # 07-03 and 2014-0016, MCBL.

For DSS-colitis and AOM/DSS CRC model, any mouse with three of the clinical signs in Category A or any one clinical sign in category B was euthanized: Category A: ruffled hair coat, hunched posture, lethargy, weight loss of 20%, rectal prolapse greater than 2 mm. Category B: weight loss of greater than or equal to 25% that does not improve or stabilize in 24 h of close observation, inability to move about the cage, inability to right itself, labored breathing, pale extremities, bloating, rectal prolapse greater than 5 mm, rectal bleeding score of 3.

For *Apc*^*min+/-*^ intestinal tumor model, mice displaying clinical signs of 10% loss of body weight compared to non *Apc*^*min+/-*^ littermates, hunched posture, rough coat, slower activity compared to cage mates, pinched face, rectal protrusion, rectal bleeding were evaluated by the veterinary staff for euthanasia.

For mice in aging study, any mouse that displays 20% weight loss with hunched posture and rough coat, or any of the following: labored breathing, incoordination, seizures, lethargy, or becoming prostrate, was euthanized.

### Cells

The CRC119 human colon cancer cell line derived from a metastatic female CRC patient was acquired as a generous contribution from Dr. David Hsu at Duke University^25^. These cells were cultured in RPMI 1640 medium supplemented with 10% fetal bovine serum (FBS) and 1% penicillin and streptomycin and were incubated at 37 °C and 5% CO2.

MEFs were isolated from E13.5 embryos and maintained in Dulbecco’s modified Eagle’s medium (DMEM) supplemented with 10% FBS and 1% penicillin and streptomycin.

Bone marrow-derived macrophages were isolated from femurs of 8-week-old female mice and grown in DMEM supplemented with 10% fetal calf serum, 20% L929 conditioned medium and 1% penicillin and streptomycin.

### NAPRT deletion and ectopic expression, and NAMPT deletion in CRC119 cells

To generate NAPRT or NAMPT-deficient CRC119 cells using CRISPR/Cas9-mediated gene editing, specific guide RNAs (gRNAs) targeting NAPRT or NAMPT were designed. For NAPRT deletion, the forward and reverse sequences of the gRNA were 5′-CACCG ACAAAGGCTGCCCGCTCGCC-3′ and 5′-AAACGGCGAGCGGGCAGCCTTTGTC-3′, respectively. For NAMPT deletion, the forward and reverse sequences of the gRNA were 5′-CACCGGGCCACTGTGATTGGATACC-3′ and 5′-AAACGGTATCCAATCACAGTGGCCC-3′, respectively. The gRNAs were cloned into a lentiCRISPRv2 vector via BsmBI restriction enzyme sites and subsequently transfected into CRC119 cells. After two days selection with 2 μg/ml puromycin, single colonies were isolated. To identify NAPRT KO clones, each colony was split in half: one half was expanded, while the other was seeded in a 96-well plate and cultured for 2 days in a medium supplemented with 100 nM STF-118804 and 100 μM NA. Clones that failed to survive in this selection medium were selected for further validation of NAPRT inactivation. Similar strategy was used to identify NAMPT KO clones, but using regular growth medium for selection and NA-containing medium for maintenance. Cellular viability was determined by CellTiter-Glo® kit (Promega). The efficiency of NAPRT or NAMPT depletion was validated by immunoblotting analysis.

For transient NAPRT add-back in NAPRT-deficient CRC119 cells, cells were transfected with pcDNA3-HA-hNAPRT.

For stable NAPRT add-back, pLV-mCherry:T2A:Puro-EFHA-HA-mNAPRT was custom built by Vector Builder. NAPRT-expressing and control lentiviruses were packaged in 293T cells and used to infect CRC119 NAPRT-deficient cells followed by puromycin selection.

### Experimental animals

All mice at the NIEHS facility were housed in individualized ventilated cages (Techniplast, Exton, PA) with a combination of autoclaved nesting material (Nestlet, Ancare Corp., Bellmore, NY, and Crink-l’Nest, The Andersons, Inc., Maumee, OH) and housed on hardwood bedding (Sani-chips, PJ Murphy, Montville, NJ). Mice were maintained on a 12:12-h light:dark cycle at 22±0.5 °C and relative humidity of 40% to 60%. Mice were provided ad libitum autoclaved plant-based chow diet supplemented with 83 mg/Kg of NA (NIH-31, cat# 7017, Harlan Laboratories, Madison, WI) and deionized water treated by reverse osmosis. Mice were negative for mouse hepatitis virus, Sendai virus, pneumonia virus of mice, mouse parvovirus 1 and 2, epizootic diarrhea of infant mice, mouse norovirus, *Mycoplasma pulmonis, Helicobacter* spp., and endo- and ectoparasites upon receipt, and no pathogens were detected in sentinel mice during this study.

The experimental mice were acclimatized for a week before being randomly assigned to experimental groups. Clinical health assessments were performed twice weekly during the experiments.

### Generation of whole-body NAPRT KO (*Naprt*^*-/-*^*)*, *Naprt*^*+/+*^*Apc*^*min/+*^ and *Naprt*^*-/-*^*Apc*^*min/+*^ mice

All mice utilized in the study were on the C57BL/6 J background. The global NAPRT knockout (KO) mice (*Naprt*^*-/-*^) were generated by CRISPR-cas9 technology by the Gene Editing and Mouse Model Core Facility at National Institute of Environmental Health Sciences (NIEHS). To generate the NAPRT mutant line, single cell C57BL/6 J embryos were microinjected with 100 ng/μl Cas9 protein (New England BioLabs EnGen Spy Cas9 NLS - M0646M) and 125 ng/μl sgRNA targeting exon 9 ORF at GGCAGTGAGGTGAATGTCATNGG[PAM] (synthesized by IDTDNA). Injected single cell embryos were surgically transferred to SWISS pseudo-pregnant mice. F0 founders were screened at weaning using primers that amplify the entire target region (Fwd: GACCATGGACCTATTTCAGGGG; Rev: AGGTCCAGTAGCAAAGACCCTAT). Several independent founders with a NHEJ alleles of interest were crossed to C57BL/6 J wild-type mice, and their F1 offspring were screened by genomic DNA sequencing to confirm transmission of the mutant NAPRT alleles. Among several different alleles generated, a catalytic inactive allele containing a 21 bp NHEJ deletion, which resulted in the in-frame loss of coding sequence of 376-IGIGTSV-382, disrupting the catalytic site in exon 9 (Supplementary Fig. 3a, d(CAT or d(21)), was selected for future studies. The d(21) mouse colony was subsequently genotyped by Transnetyx (Cordova, TN) using primer probe assays specific for the wild-type allele and d(21) catalytically-inactive allele. All experimental mice from the d(21) allele were further backcrossed into the C57BL/6 J background for at least 4 generations.

*Naprt*^*+/+*^*Apc*^*min/+*^ and *Naprt*^*-/-*^*Apc*^*min/+*^ mice were generated by crossing wild-type (WT) and NAPRT KO mice with *Apc*^*min/+*^ mice (C57BL/6J-ApcMin/J, Jax: 002020, both sexes) respectively. Age- and gender-matched mice were used for all experiments. Both males and females were analyzed in all experiments, and sex-specific findings were observed and reported.

### Isotopic tracing

For D4-NAM isotopic tracing experiment, male WT and NAPRT KO mice, aged between eight to thirteen weeks, were orally administered either 80 mg/kg of nicotinamide labeled with four deuterium atoms on the pyridine ring (D4-NAM) or received a PBS gavage in the control group. To prevent any bias, gavages were staggered to keep the time between gavage and sacrifice constant and were administered alternately between the WT and NAPRT KO mice. The mice were euthanized at 0, 3, 6, or 24 hours after gavage administration, and small intestines and colons were flushed and flash-frozen in liquid nitrogen along with blood, liver, pancreas, kidneys, spleen, heart, lungs, quadriceps muscles, brain, white and brown adipose tissues. These samples were then preserved at −80 degrees until further processing. Each group receiving a specific treatment consisted of 4-5 mice.

For D4-NA isotopic tracing experiment, male WT and NAPRT KO mice, aged between eight to thirteen weeks, were orally administered with either 80 or 400 mg/kg of nicotinic acid labeled with four deuterium atoms on the pyridine ring (D4-NA) or received a PBS gavage in the control group. To prevent any bias, gavages were staggered to keep the time between gavage and sacrifice constant and were administered alternately between the WT and NAPRT KO mice. The mice were euthanized at 1, 3, 6 hours after gavage of 80 mg/kg D4-NA or 3 hr after 400 mg/kg D4-NA administration, and plasma was isolated by centrifugation of systemic blood at 2000g for 10 minutes and kept at −80 degrees until further processing.

### AOM-induced DNA damage and DSS-induced colitis

For the AOM-induced DNA damage model, 9 to 16-week-old female WT and NAPRT KO mice were supplemented with 3 g/L NA in the drinking water. Twenty-four hours later, they were intraperitoneally (i.p.) injected with one dose of 10 mg/Kg AOM with a continuous supply of the NA water for the rest of the experiment. Colon tissues were collected either 12 hours or 48 hours post-injection.

For the DSS-induced colitis model, 8 to 12-week-old female WT and NAPRT KO mice were treated with 2.5% DSS in drinking water for 7 days, then recovered with regular drinking water for 2 days (protocol 1). Alternatively, mice were treated with 2.5% DSS in drinking water containing 3 g/L NAM for 7 days without the recovery period (protocol 2) Throughout these experiments, the body weight, rectal bleeding, and diarrhea of experimental mice were monitored daily, and the severity of rectal bleeding was scored based on a score system as previously described^41^.

### AOM/DSS, *Apc*^*min/+*^, and spontaneous aging tumorigenesis models

For the chronic inflammation-related colon cancer model (AOM/DSS-colon cancer model), 9 to 16-week-old WT and NAPRT KO female mice were initially i.p. injected with 10 mg/kg AOM. One week later, they were treated with 2% DSS in drinking water for 8 days. Tumors were analyzed 13 weeks after the AOM injection. Colonic tumor burden was quantified by determining the ratio between the area occupied by tumors and the total area of the colon using ImageJ 2.14.0 (Fiji) software.

For a genetic intestinal tumor model, *Naprt*^*+/+*^*Apc*^*min/+*^ and *Naprt*^*-/-*^*Apc*^*min/+*^ mice were utilized. Small intestines and colons were collected when the mice were 16 weeks old. Tumors were analyzed and tumor burden was quantified by ImageJ 2.14.0 (Fiji) software. The different tumor burdens between *Naprt*^*+/+*^*Apc*^*min/+*^ and *Naprt*^*-/-*^*Apc*^*min/+*^ were only observed in males.

For the spontaneous aging model, male and female WT and NAPRT KO mice were allowed to age to 24 months, dissected and examined for tumors in different organs. All tumors were collected, fixed in formalin and paraffin embedded, and hematoxylin and eosin sections were examined.

### Immunofluorescence staining

Colonic tissues from WT and NAPRT KO mice treated with AOM were formalin fixed, embedded in paraffin, and sectioned into slides. The slides were then subjected to a series of steps: deparaffinization in xylene and rehydration through graded ethanol. Antigen retrieval was performed using citrate-based unmasking solution in a microwave for 20 min at 10% power. The Squenza Slide Rack and Coverplate System was used for staining the slides with a mouse anti-rabbit phospho-histone H2AX (γH2AX, Cell Signaling Technology, 9718) antibody or anti-NAPRT antibodies (NAPRT #1, Proteintech, 13549-1-AP), NAPRT #2 (Abcam, ab211529) overnight at 4 °C followed by goat anti-rabbit or anti-mouse IgG H&L (Alexa Fluor® 594) for one hour at room temperature. Nuclei were stained with 5 μg/ml DAPI for 5 minutes, followed by mounting with Prolong Diamond Antifade. Images from five different fields of view per condition were acquired with the confocal Zeiss LSM 880 or 980 microscope. Total nuclear fluorescent intensity from the γH2AX channel from each cell was quantified using a custom ImageJ 2.14.0 (Fiji) macro.

CRC119 cells seeded on coverslips in the 12-well plate were fixed with cold methanol for 10 minutes and permeabilized with 0.5% Triton X-100 in PBS at room temperature for 10 minutes. Coverslips were incubated with anti-γH2AX (1:200) for 1 hour at room temperature. After washing with PBS for 4-5 times, goat anti-rabbit IgG H&L (Alexa Fluor® 594) was applied for 1 hour at room temperature. Nuclei were stained with 5 μg/ml DAPI for 5 minutes, followed by mounting the samples using Prolong Diamond Antifade. Imaging and analysis were performed as described for colonic tissues.

For co-staining of HA-NAPRT and γH2AX, cells were grown in 8-chamber slides (Thermo Fisher), fixed with 4% formaldehyde for 10 minutes at room temperature, permeabilized with 0.5% Triton X-100 in PBS for 10 minutes, and stained with a mixture of anti-HA (Santa Cruz Biotechnology, sc-7392) and anti-γH2AX antibodies, followed by goat anti-mouse IgG H&L (Alexa Fluor® 594) and goat anti-rabbit IgG H&L (Alexa Fluor® 488) antibodies, respectively. Nuclei were stained with 5 ug/ml DAPI. Images from five different fields of view per condition were acquired with the confocal Zeiss LSM 880 microscope. Total nuclear fluorescent intensity from both channels in each cell was quantified using a custom ImageJ 2.14.0 (Fiji) macro.

### Histopathological evaluation

For the colon tissues from mice treated with DSS, 0.5 cm distal colon was collected and fixed in formalin. After paraffin embedding, sectioned slides were stained with hematoxylin and eosin (H&E), and evaluated by a certified pathologist. The evaluation included identification and quantification of inflammation, ulceration, edema, goblet cell loss, and granulation tissue/fibrosis.

For the colon tissues from mice treated with AOM/DSS, *Naprt*^*+/+*^*Apc*^*min/+*^ and *Naprt*^*-/-*^*Apc*^*min/+*^ mice, Swiss roll sections of the colon were prepared, stained with H&E, and assessed by a board-certified pathologist. The evaluation comprised identifying tumors, assessing atypical hyperplasia and adenomas. Detailed descriptions of lesions and their morphological diagnoses were provided. Atypical hyperplasia exhibited distinctive features including crowded epithelial cells lining crypts with retained polarity and some dysplasia. Adenomas, on the other hand, displayed proliferations of branching tubules or finger-like projections in the lamina propria, often presenting various degrees of dysplasia characterized by disrupted cell layering and irregular nuclear features. Carcinoma was identified as an invasive proliferation of epithelial cells invading the underlying lamina propria and submucosa.

For spontaneous tumors in aged mice, H&E-stained tumor slides were evaluated by a board-certified pathologist.

### RNA isolation, RT-PCR and RNA-seq

Total RNA was isolated using TRIzol™ Plus RNA Purification Kit (Thermo Fisher Scientific) from frozen colon tissue after pulverization in liquid nitrogen with mortar and pestle. Total RNA from cultured cells was extracted using the RNeasy kit (Qiagen). For RT-PCR analysis, RNA was reverse transcribed using High-Capacity cDNA Reverse Transcription kit (Thermo Fisher Scientific). Real-time PCR assays were conducted on a CFX96 or CFX Opus 384 real-time PCR instruments (Bio-Rad) utilizing iQ SYBR Green SuperMix (Bio-Rad). Primer sequences can be found in Supplementary Data 7.

To conduct RNA-seq analysis, libraries were prepared using the TruSeq Stranded Total RNA kit (Illumina, San Diego, CA) with Ribo-Zero technology. The indexed samples were sequenced on the Nova-seq 6000 (Illumina) employing a 75-bp single-end protocol as per the manufacturer’s guidelines. Subsequently, reads (ranging from 40 to 80 million reads per sample) were aligned to mm10 reference genome separately using the STAR aligner (version 2.6)^56^. Gene quantification based on GENCODE annotation release (GRCh37, p13) was conducted utilizing Subread featureCounts (version 1.4.6)^57^. Principal Component Analysis (PCA) aided in detecting any potential sample outliers. Comparisons between the vehicle and treatment samples were conducted to detect differentially expressed genes. To adjust the false discovery rate (FDR), the Benjamini and Hochberg method was employed. A gene was deemed significantly differentially expressed if the FDR-adjusted p-value for differential expression was below 0.05. The heatmaps were generated utilizing Partek Genomic Suite (Version 6.6, Partek, St. Louis, Missouri).

The RNA-seq data are available in the Gene Expression Omnibus repository at the National Center for Biotechnology Information (RNA-seq dataset of the AOM experiment: GSE271834; RNA-seq dataset of the DSS-colitis experiment: GSE271250).

### Pathway enrichment analysis

All pathway enrichment analysis were preformed using generated differential gene lists in https://biit.cs.ut.ee/gprofiler/gost.

### Western blotting

Cellular pellets were lysed in RIPA buffer supplemented with Complete Mini protease inhibitor cocktail (Roche Diagnostics). Subsequently, the proteins were separated on 4–20% gradient SDS-PAGE gel (BioRad), followed by their transfer onto PVDF membranes. Primary antibodies targeting specific proteins-NAPRT #1 (Proteintech, 13549-1-AP), NAPRT #2 (Abcam, ab211529), PAR (Sigma, MABE1016; Sigma, AM80), PARP (Cell Signaling Technology, 9542), MAR (Bio-Rad, HCA355), γH2AX (Cell Signaling Technology, 9718), HA (Santa Cruz Biotechnology, sc-7392) and Actin (Sigma, MAB1501) were used followed by incubation with fluorescently labeled secondary antibodies and detection on the Odyssey (LI-COR) or ChemiDoc (Bio-Rad) imaging systems.

### Mucin high-iron diamine and Alcian blue staining

Thin sections of formalin-fixed paraffin-embedded tissue (8 μm) were deparaffinized in xylene for five minutes and then sequentially passed through various alcohol concentrations (100, 90, 75, 50, and 0 percent) for five minutes each. These sections were subjected to treatment with a high-iron-diamine (HID) solution for 16 hours at Room Temperature. The HID solution composition comprised 120 mg of NN-dimethyl-meta-phenylenediamine-dihydrochloride and 20 mg of NN-dimethyl-paraphenylenediamine-dihydrochloride dissolved in distilled water up to 50 ml. To this solution, 1.4 ml of freshly prepared 60% (w/v) ferric chloride solution was added (all from Sigma). Following treatment, sections were rinsed multiple times in tap water and counterstained using 1% Alcian blue in 3% acetic acid (pH 2.5) for 20 minutes. After rinsing with water, the sections were mounted. Sulfated mucins were distinguished by displaying a brown/black color, while sialomucins exhibited a blue coloration. Mucin positive area in the colon were quantified by Fiji.

### IL-6 ELISA

Measurement of IL-6 protein levels in plasma of DSS-treated mice was performed using IL-6 Mouse ELISA Kit (Thermo Fisher Scientific, KMC0061).

### Colony formation assay

Pools of six single cell clones of WT or NAPRT KO CRC119 cells were seeded at 500 cells/well in 6-well plates in triplicates. 24 hours later, medium was replaced to fresh growth medium with 100 μM NA and/or 100 nM STF-118804 for 24 hours. Then, medium was replaced to fresh growth medium with corresponding NA/STF with or without 1 mM MMS for 1 hour. Subsequently, cells were washed twice with PBS and medium was replaced to fresh growth medium with 100 μM NA.

Colonies were stained with Crystal Violet 7 days later and visible colonies were counted.

### Primary mouse organoids

Small intestinal and colon organoids were isolated from 6-8 weeks-old WT or NAPRT KO female mice according to IntestiCult™ protocol (StemCell). Organoids were grown in 3D in 25 μl Matrigel (Sigma) domes per well of 48-well plates for maintenance and in 6 μl Matrigel per well of 96-well plates for assays. Small intestinal organoids were grown in IntestiCult™ Organoid Growth Medium (Mouse) (StemCell). Colon organoids were grown in DMEM/F12 (ThermoFisher Scientific) supplemented with 20% R-Spondin conditioned medium, 10% Noggin-conditioned medium, 50 ng/ml human EGF (R&D Systems), 0.15 nM Wnt3a-Surrogate (ThermoFisher Scientific), 1:500 B27-Vit A (ThermoFisher Scientific), 1 mM N-acetylcysteine (Sigma), and 100 ug/ml Primocin (ThermoFisher Scientific). R-Spondin and Noggin-expressing cells for the production of the conditioned media were obtained from Johns Hopkins University.

### Real time metabolic (Seahorse) analysis

Cells were seeded at 6 × 10^4^ cells per well on XF24 cell plates with 100 μM NA in 250 μL RPMI 1640 medium supplemented with 10% FBS and 1% penicillin and streptomycin. 48 hours later, for glycolysis stress test, medium in each well was replaced with 500 μL XF RPMI assay medium (pH 7.4) supplemented with 2 mM glutamine, 100 μM NA and cells were incubated for two hours at 37 °C without CO2 before running the assay. The Seahorse measurements on Seahorse XFe24 Analyzer (Seahorse Biosciences) included sequential injections of glucose (10 mM), oligomycin (1 μM), and 2-deoxy glucose (2-DG, 50 mM).

For mitochondrial stress test, medium in each well was replaced with 500 μL XF RPMI assay medium (pH 7.4) supplemented with 100 μM NA, 2 mM glutamine, 10 mM glucose, 1 mM pyruvate and cells were incubated for two hours at 37 °C without CO2 before running the assay. The Seahorse measurements on Seahorse XFe24 Analyzer (Seahorse Biosciences) included sequential injections of oligomycin (1.5 μM), FCCP (1 μM), and rotenone (0.5 μM). Following the Seahorse assay, assay medium was carefully removed without disturbing the cells, and protein concentration was determined using BCA assay (ThermoFisher Scientific). The raw ECAR and OCR measurements were normalized to the total protein content.

### scRNA-seq analysis of colonic epithelial cells

#### Isolation of colonic epithelial cells

Mouse colons were freshly dissected. Following the removal of the mesentery/fat and connective tissue, colons from three mice per group were combined, flushed with ice-cold CMF-PBS, longitudinally opened, and rinsed with ice-cold CMF-PBS. The colons were then cut into 0.5 to 1 centimeter pieces and washed once in rinse buffer containing 5.6 mM Na2HPO4 x2H2O, 8.0 mM KH2PO4, 96.2 mM NaCl, 1.6 mM KCl, 43.4 mM Sucrose, 54.9 mM D-sorbitol. Then, the colon pieces were resuspended in isolation buffer (2 mM EDTA and 0.5 mM DTT in rinse buffer) and incubated on a rotation platform for 20 min at room temperature. Subsequently, supernatants were removed, and the remaining tissue was resuspended in 10 ml ice-cold rinse buffer and pipetted 25 times up and down with a 25 ml pipette. Following the settling of the large tissue pieces, the supernatants containing crypts were transferred to a new tube. After additional pipetting 25 times up and down with a 25 ml pipette and settling of the tissue pieces, the supernatants were centrifuged at 300 g, 4 °C for 3 min, and the pellets were resuspended in 10 ml room-temperature single-cell-isolation-buffer (1 mg/ml DNase I, 5 mM MgCl2, 20 μM Y27 in TrypLE Express) and incubated on 100 RPM at 37 °C for 20 min. Then, the cell solution was passed through the 40 μm cell strainers. Digestion was stopped by the addition of 35 ml ice-cold CMF-PBS, and centrifugation at 500 g, 4 °C for 4 min. The cell pellets were resuspended in ice-cold PBS with 2% FBS.

#### scRNA-seq library preparation and sequencing

Cell counts and viability assessments were performed using fluorescent AO/PI (Logos Biosystems, F23001) staining via a Luna-FX7 cell counter (Logos Biosystems, L70001). Subsequently, approximately 10,000 viable cells exhibiting over 50% viability were utilized and loaded into the Single Cell Chip for the creation of single-cell emulsion in the Chromium Controller by 10x Genomics (120263) employing the Chromium Single Cell 3’ Library & Gel Bead Kit v3.1 (10x Genomics, 1000268). Following the manufacturer’s guidelines (10x Genomics, 1000268), the process encompassed reverse transcription of mRNA and subsequent cDNA amplification. The amplified cDNA underwent fragmentation to generate NGS libraries, which were sequenced by the NIEHS Epigenomics and DNA Sequencing Core Laboratory.

#### scRNA-seq data processing

Initial processing of raw reads involved the utilization of the Cell Ranger Single-Cell Software Suite (version 6.0.1, 10x Genomics Inc., CA). The demultiplexed FASTQ files, composed of paired-end reads (Read 1: 30 bp, Read 2: 100 bp), were generated using the CellRanger mkfastq command. Primary data analyses, including alignment, filtering, barcode counting, and quantification of unique molecular identifiers (UMIs) to determine gene transcript counts per cell, were conducted using CellRanger count command referencing the genome “refdata-gex-mm10-2020-A”. This process resulted in the creation of a gene-barcode matrix.

The raw gene expression matrices produced per sample by CellRanger were imported into R (version 4.3.1) and transformed into a Seurat object using the Seurat R package (version 4.3.2)^58^. The function decontX from the R package “celda” was employed to eliminate RNA contamination^59^. Removal of deceased cells, doublets, and low-quality cells was performed systematically. This involved evaluating the total UMIs, total genes, and the percentage of UMIs originating from the mitochondrial genome for each cell. Cells with over 10% UMIs derived from the mitochondrial genome were excluded. Additionally, cells falling outside the upper and lower bounds—calculated as the mean plus or minus two standard deviations (SD) for both total UMIs and genes—were further eliminated based on this criterion.

#### Single-cell gene expression quantification and major cell type classification

The cleaned cells underwent normalization of gene expression, identification of highly variable genes (HVGs), and scaling using the Seurat SCTransform function. Normalization involved adjusting gene expression data to a standardized total cellular read count (set at 10,000 per cell), while the top 2000 highly variable genes were selected as features for subsequent dimensionality reduction and clustering. Additionally, Cell-Cycle scores were computed using the Seurat CellCycleScoring function, utilizing specific cell-cycle-related gene pairs to classify cells into G1, S, and G2/M phases. Any cell cycle-related effects were addressed through regression using the SCTransform function.

Principal Component Analysis (PCA) was conducted using the Seurat RunPCA function, and the top 40 principal components (PCs) were chosen for further analysis. Subsequently, the Seurat FindNeighbors function established a Shared Nearest Neighbor (SNN) Graph, followed by the application of the RunUMAP function to visualize the selected significant PCs. Cell clustering was accomplished using the FindClusters function, with a parameter of “resolution = 1”.

Marker genes for distinct clusters or cell types were identified using the Seurat FindMarkers function, which compared the gene expression values of cells within a particular cluster against those from other clusters. Cell clusters were annotated using two approaches: canonical marker genes were utilized to assign known biological cell types, while predicted marker genes helped confirm annotated cell types and name clusters lacking canonical marker genes.

The access number of scRNA-seq dataset generated from colon tissues is GSE271836.

For analysis of small intestinal scRNAseq, a dataset GSE261216 from a separate unpublished study was used.

### Enzymatic assays for NAD and ATP

Cells or organoids were seeded in clear-bottom white plates obtained from Corning (#3610). The determination of total NAD using enzymatic methods was conducted utilizing the NAD/NADH-Glo™ kit sourced from Promega. Additionally, CellTiter-Glo® or CellTiter-Glo® 3D from Promega were employed for determination of ATP levels and served as a surrogate measure for number of viable cells.

### Sample preparation for LC-MS

Cells were cultured in 6-well plates and subjected to various treatments. Metabolites were extracted following a brief saline wash. This extraction process involved scraping the cells on dry ice into a solution comprising 80% methanol and 20% water. Subsequently, the plates were incubated at −80 degrees for 15 minutes, and the resulting extracts were transferred to microcentrifuge tubes. Samples were incubated at 4 degrees with 800 rpm mixing followed by centrifugation at 14,000 rpm for 10 minutes. Supernatants were collected and dried using a vacuum concentrator at room temperature. The resultant dry pellets were reconstituted in 30 μl of sample solvent (a mixture of water, methanol, and acetonitrile in a 2:1:1 ratio, v/v), and 3 μl was subjected to further analysis by liquid chromatography-mass spectrometry (LC-MS). The final results were normalized to the total protein content.

In the case of tissue analysis, frozen tissues were pulverized using a mortar in the presence of liquid nitrogen and maintained on dry ice. Approximately 10-20 mg of tissue powder was accurately weighed and subjected to extraction using cold 80% methanol and 20% water, following the same procedure as described for the cells. LC-MS signals for different metabolites were normalized to tissue weight.

### Targeted LC-MS methods for NAD pathway metabolites

The methods for sample preparation and relative and absolute LC-MS measurements of unlabeled and isotopically labeled NAD pathway metabolites NAD, NAAD, NA, NAM, NAMN, NMN were as described under Targeted Analyses of Compounds from the NAD Pathway (NIEHS LC-MS) in^21^. Similarly, NADP and ADPR measurements were made using the identical C18-reversed-phase method described previously^21^ with the additional narrow MS1 channels for NADP (MS acquisition range of m/z 735 – m/z 760 with an EIC drawn for the precursor m/z 744.083 and retention time of 2.3 minutes) and ADPR (MS acquisition range of m/z 555 – m/z 580 with an EIC drawn for the precursor m/z 560.080 and a retention time of 2.0 minutes). The representative spectral peaks of these targeted measurements are provided in Supplementary Data 8.

Relative quantification of nicotinuric acid (NUA) (Supplementary Data 9) was performed separately using an Agilent 1200 Series HPLC coupled to a Thermo Q Exactive Plus mass spectrometer. An autosampler was used to inject the samples onto an Agilent Zorbax 300SB-C18 reversed-phase column (2.1 × 150 mm, 5-micron) using a 1.0 µL injection volume for each. Solvent A consisted of 10 mM ammonium acetate in H2O + 0.1% formic acid, and Solvent B consisted of methanol + 0.1% formic Acid. A flow rate of 50 µL per minute was used for the first 6 minutes of the run at 2.0% B solvent, followed by a gradient from 2.0–95.0% B from 6 to 20 minutes along with an increased flow rate of 150 µl per minute. The solvent was held at 95.0% B from 20 to 30 min and returned to 2.0% B for the remainder of the run. A HESI (heated electrospray ionization) source was used with positive polarity, a capillary temperature of 320 °C, the source voltage of 3.2 kV, S-lens RF level of 60, and a sheath gas flow rate of 16.0. One full scan from 100–900 m/z was performed at 17,500 resolution, followed by targeted MS2 scans at 35,000 resolution with an isolation width of 2.0 m/z. Normalized CID collision energy was set to 25. The total run time was 38 min. A blank was run in-between each sample to minimize and monitor for carryover.

### Targeted LC-MS methods for NAD pathway metabolites in mouse plasma

5 µL mouse plasma was mixed with 5 µL ^13^C yeast extract, followed by the addition of 40 µL ice-cold methanol. The mixture was vortexed for 1 min, kept on ice for 10 min with occasional vortexing, and then centrifuged for 10 min at 20,000 *g* at 4 °C. The supernatant was transferred to an LC vial, and 2 µL was injected.

A Vanquish Horizon UHPLC system equipped with ACQUITY UPLC BEH Amide column (1 mm×100 mm, 1.7 μm, Waters) was used for LC separation, coupled to Exploris 480 mass spectrometer (Thermo Fisher Scientific). LC mobile phase A was water with 5 mM ammonium acetate (pH 6.8), and mobile phase B was 100% Acetonitrile. The LC gradient was as follows: 0-1.5 min, 85% B; 5.5 min, 35% B; 10.5 min, 35% B; 10.6 min, 10% B; 12.5 min, 10% B; 13.5-20 min, 85% B. The flow rate was 40 µL/min, except at 13.5 min when it was increased to 70 µL/min to accelerate column re-equilibration. The column temperature was maintained at 25 °C.

For all metabolites, the following MS parameters were used: spray voltage, 3000 V; sheath gas, 18; auxiliary gas, 5; vaporizer temperature, 80 °C; ion transfer tube temperature, 300 °C; and S-lens, 30%. For Nicotinic acid (NA) and Nicotinamide (NAM) analysis, full-scan acquisition in positive mode was performed over an m/z range of 100–400 with a mass resolution of 120,000. Extracted ion chromatograms corresponding to m/z values of metabolites with 0, 3, or 4 deuterium incorporations (M + 0, M + 3, and M + 4, respectively) were used for quantification. ^13^C-labeled NAM from ^13^C yeast extract was used as an internal standard for both NA and NAM due to structural and retention time similarity. To determine the concentration of ^13^C-labeled NAM in the yeast extract and to correct for differential ion responses between NA and NAM, standards of NA and NAM (0.16, 0.8, 4, and 20 ng/µL) were spiked into 5 µL ^13^C yeast extract, followed by methanol extraction and LC–MS analysis. Peak areas of extracted ions corresponding to NA, NAM, and ^13^C-labeled NAM were used to determine internal standard concentration and ion response factors. For nicotinic acid riboside (NAR) analysis, due to interference in the D4-NAR (M + 4) channel at MS1 level, characteristic fragment ions (m/z 124.0392, 127.0581, 128.0643, and 130.0594) from MS2 spectra of NAR, D3-NAR, D4-NAR, and ^13^C-NAR precursors were used for quantification. MS2 data acquisition was performed with the following parameters: isolation window, 0.8 m/z; HCD collision energy, 30%; resolution, 30,000; and a targeted mass list of m/z 256.0816, 259.1004, 260.1067, and 267.1185. To determine the concentration of ^13^C-labeled NAR in the yeast extract, standards of NAR (0.16 and 0.8 ng/µL) were spiked into 5 µL ^13^C yeast extract, followed by methanol extraction and LC–MS analysis. Skyline was used for MS2-based quantification, while the FrameSeed function in SIEVE was applied for MS1-based quantification. LC-MS data and representative spectral peaks from this measurement are provided in Supplementary Data 10.

### Untargeted Metabolomics

#### Pre-Analytical Materials and Methods

##### Sample Preparation

Pre-extracted, dried colon samples were removed from storage at −80 °C, allowed to warm to room temperature, and resuspended via the addition of a range of 200 µL−334 µL of water-acetonitrile 98%:2% *v/v*, normalized based on extract weights (ranging from 10.0 mg to 16.7 mg). Resuspended extracts were briefly vortexed (3 s) and then centrifuged for 10 minutes at 14000 rcf at 4 °C (Eppendorf Centrifuge 5425 R). 100 µL of supernatant of the resuspended extract, the soluble fraction of the extract, was transferred to 2 mL autosampler vial (12 mm×32 mm height vial, 12 mm screw cap, and PTFE/silicone septa, Agilent) containing a glass microvolume insert (Agilent). The pellet (insoluble extract) was discarded in accordance with chemical and biological safety procedures. 30 µL of supernatant from each sample was pooled into a 2 mL autosampler vial for a quality control standard.

### Analytical Materials and Methods

#### Untargeted Metabolomics: Vanquish – Tribrid Fusion

##### Instrumentation

Samples were analyzed using an ultra-high performance liquid chromatograph (Vanquish^TM^ Horizon UHPLC, Thermo Scientific) coupled to a high-resolution mass spectrometer (Orbitrap Fusion^TM^ Tribrid, Thermo Scientific). EASY-Max NG^TM^ was used as the ionization source, operated in the heated-electrospray ionization (H-ESI) configuration. The source parameters in positive ionization mode were as follows: spray voltage of +4000 V, sheath gas of 50 arbitrary units (arb), auxiliary gas of 10 arb, sweep gas of 1 arb, ion transfer tube at 325 °C, vaporizer at 350 °C. The source parameters used in negative ionization mode were identical, with the exception of the spray voltage of −3000 V. Prior to measurement, the mass spectrometer was calibrated using FlexMix (Thermo Scientific) following manufacture directions. EASY-IC™ (Thermo Scientific) was used during data collection; this is a secondary reagent ion source, yielding fluoranthene ions that are used as a lock mass to improve *m/z* accuracy by correcting for mass errors that result from variation in *m/z* measurement (e.g., scan-to-scan variation) and environmental changes (*e.g*. ambient temperature).

Chromatographic separation was carried out on a Kinetex F5 analytical column (2.1 inner diameter, 100 mm length, 100 Å, 2.6 µm particle size, Phenomenex) with corresponding guard cartridge. The column was maintained at 30 °C during separation. The Vanquish solvent pre-heater was maintained at 30 °C. Gradient elution was performed after an initial period of isocratic elution using water with 0.1% acetic acid *v/v* (A) and acetonitrile with 0.1% acetic acid *v/v* (B). Separation was performed as follows: 0% B from 0 − 2.0 min, 0% to 100% B from 2.0 to 10.5 min, 100% B from 10.5 to 12.0 min, 100% to 0% B from 12.0 to 13.0 min, 0% B from 13.0 to 20.0 min. The flow rate was 500 µL min^-1^. A 10 µL static mixer was used. The post column flow path consisted of a Viper^TM^ connection (0.1 mm ID, 550 mm length, Thermo Scientific) from the column to a six-port value and PEEK tubing from the six-port value to the ionization source.

##### Data Acquisition: Liquid chromatography – mass spectrometry (LC-MS)

LC-MS data were collected from individual samples, system blanks, and a pooled quality control. System blanks consist of the same solvent used to resolubilize samples without biological material and are used to evaluate the chemical background of the results from the analysis system; system blanks were analyzed after every seven samples in the data acquisition order. The pooled quality control (QC) was generated by combining 30 µL aliquots from every sample in the data set into one pooled sample. Samples and system blanks were each injected at 4 µL; the QC pool was injected 9 times at different volumes (3 injections each at 2 µL, 4 µL, and 6 µL). MS data were collected with an anticipated LC peak width of 8 s and a default charge of 1. MS data were acquired at 120,000 resolution from *m/z* 100-1000 with an RF lens of 60% and a maximum injection time of 50 ms.

##### Data Acquisition: Liquid chromatography – tandem mass spectrometry (LC-MS/MS)

Prior to acquiring LC-MS data for samples, liquid chromatography – tandem mass spectrometry (LC-MS/MS) data were acquired using the AcquireX (Thermo Scientific) deep scan methodology. The acquisition order for AcquireX was the following: system blank (n = 1 injection) for exclusion list generation, QC (n = 1 injection) for inclusion list generation, and n = 7 injections of QC. MS and MS/MS data were collected with an anticipated LC peak width of 8 s and a default charge of 1. MS data were acquired at 120,000 resolution from *m/z* 100-1000 with an RF lens of 60% and maximum injection time of 50 ms. MS/MS data were acquired at 30,000 resolution using an isolation width of 1.5 (*m/z*), stepped assisted higher-energy collision induced dissociation was used with energy steps of 20, 35, and 60 normalized collision energy, and a maximum injection time of 54 ms. The inclusion list was generated and updated via AcquireX with a low and high mass tolerance of 5 parts-per-million (ppm) mass error. An intensity filter was applied with an intensity threshold of 2.0 ×. Dynamic exclusion was used with the following parameters: exclude after *n* = 3 times; if occurs within 15 s; exclusion duration of 6 s; a low mass tolerance of 5 ppm mass error; a high mass tolerance of 5 ppm mass error; and excluding isotopes.

### Data processing

#### Processing of raw data – feature finding

Data files (.raw) were processed with Compound Discoverer 3.3.0.550 (ThermoFisher Scientific) to identify unique molecular features and, where possible, annotate them with chemical names. Features with distinct measured accurate mass, unique retention time, and MS/MS data were tabulated after removal of isotope peaks, blank contaminants, and noise artifacts from the data (workflow and parameters displayed in Supplementary Data 11). The table contained feature descriptors (*e.g. m/z* and retention time), annotation information (e.g. MS/MS database match), and peak area. Features were then processed using R via Jupyter Notebook. Processing steps included formatting of the table output from Compound Discoverer, comparison of *m/z* and retention time of annotated features versus an in-house generated list of *m/z* and retention time based on authentic chemical standards, assessment of signal response in pooled QC samples, assessment of signal variance in pooled QC samples versus samples (i.e. dispersion ratio), and multi- and univariate statistics.

### Data Quality and Data Filtering

#### Signal response evaluation

We evaluated the signal response of the pooled sample with the intention of evaluating the fundamental principle of LC-MS/MS that if more of a given feature is present, a corresponding increase in the signal should be obtained. To do this, the pooled QC sample was injected and analyzed at three volumes in technical triplicate: 2 µL, 4 µL, and 6 µL, where 4 µL is the amount of material injected during the untargeted metabolomics assay in the present experiment. For any given feature, the peak area of the feature (*i.e*. the integration of a signal of unique *m/z* over a specific retention time) in the 4 µL sample is assumed to represent the mean value of that feature in the dataset; the peak area of that feature in the 2 µL sample and 6 µL sample should therefore represent 50% and 150% of the mean value, respectively. The metric used to evaluate the signal response was the coefficient of determination (R ^2^, evaluates fit to a linear model). For every feature in the dataset, a value of these metrics was calculated to evaluate the signal response for that feature over the QC range. Any feature for which the value does not meet the filtering parameter is filtered out of the dataset, ensuring that only features displaying positive correlations within the R

^2^ = 0.7 parameter are retained for further evaluation and interpretation.

#### Assessment of Dispersion Ratio

The pooled QC was utilized to calculate the dispersion ratio for the sample set. The dispersion ratio is a metric for describing the measurement precision of a detected metabolite; the metric focuses on statistical dispersion of the pooled QC samples in relation to the dispersion of the biological test samples. If the distribution of both the biological test sample measurements and the QC random error are Gaussian, then the dispersion ratio (D-ratio) equal to 50 is the ratio of the sample standard deviation for the pooled QC samples to the sample standard deviation for the biological test samples. (If the data distribution is not Gaussian, then the raw data must be mathematically transformed.) D-ratio is a measurement of technical variance: a D-ratio of 0% means that the technical variance is zero (a “perfect” measurement), and all observed variances can therefore be attributed to a non-measurement (putatively biological) cause, whereas a D-ratio of 100% indicates that all variances can be attributed to the measurement.

#### Annotation

Features were annotated based on MS/MS spectral matching, in alignment with the Metabolomics Standards Initiative guidelines (Level 2). Prior to annotation, the data acquisition strategy AcquireX (Thermo Fisher Scientific) was used to improve coverage compared to similar data acquisition strategies (i.e., data-dependent acquisition). The public, commercial, and in-house MS/MS spectral libraries NIST2020, GNPS (accessed 04-01-2022), and mzCloud (offline, endogenous metabolites) were used to evaluate MS/MS spectral matches, as well as an in-house MS/MS spectral library acquired from authentic chemical standards purchased and analyzed by the MCF. The MS/MS spectral matching was performed in Compound Discoverer (Thermo Fisher Scientific).

During processing in the Jupyter Notebook, each feature was assigned an MSI level of confidence based on the measured data (without manual review). MSI Level 2 features have an MS/MS match to either a database entry or an in-house library reference match. MSI Level 4 and Level 5 features have either an MS/MS match but no database match, or no MS/MS match. MSI Level 2 features were promoted to MSI Level 1 after matching in-house *m/z* and retention time lists acquired on the same analytical platform using identical analytical conditions. The MSI Level 1 annotations are referred to as identified.

Abundance matrices of 277 annotated metabolites from positive and negative modes were combined (Supplementary Data 1) and used to generate PCA plot in MetaboAnalyst 6.0 without any filtering or normalization. For heatmap generation, 50 top differential annotated metabolites were used.

### Flow cytometry analysis

Blood samples were collected in EDTA-coated tubes. Red blood cells were lysed with ACK lysis buffer at room temperature for 10 min. The collected lymphocytes were incubated with anti-mouse CD16/32 at room temperature for 10 min to block the IgG Fc receptors. Expression of surface markers was detected by simultaneously staining with the following antibodies (eFluor 450 anti-mouse CD45, AF 700 anti-mouse CD4, FITC anti-mouse CD3, PE-Cy7 anti-mouse MHCII, BV711 anti-mouse CD8, PerCP-Cy™5.5 anti-mouse CD11b, APC anti-mouse CD19, BV510 anti-mouse CD11c, PE anti-mouse Ly6G, PE-Cyanine5 anti-mouse F4/80, APC/Cyanine7 anti-mouse Ly6C, PE-Dazzle 594 anti-mouse CD115) on ice for 30 min followed by wash and flow cytometry.

For flow cytometry analysis of colonic immune cells, colons were flushed with ice-cold PBS (Mg²⁺/Ca²⁺-free) and surrounding fat and mesentery were removed. Colons were then opened longitudinally and transferred to a 50 mL Falcon tube containing 20 mL ice-cold PBS. The tissue was shaken vigorously five times in fresh PBS until the intestines appeared clean and pale (white/pink). Colons were cut into 0.5–1 cm pieces and incubated in 10 mL of pre-digestion buffer (Mg²⁺/Ca²⁺-free HBSS with 2% FBS, 10 mM HEPES, 2 mM EDTA, and 1 mM DTT) at 37 °C in a shaking incubator (250 rpm) for 15 minutes followed by three washes with cold PBS. Tissue was digested in 10 mL RPMI-1640 containing 5% FBS, 10 mM HEPES, 0.6 mg/mL Collagenase IV, 0.4 mg/mL Collagenase I, 1 mg/mL Dispase II, and 50 µg/mL DNase I at 37 °C with shaking at 250 rpm for 15 minutes. After vortexing for 10 seconds, cells were filtered through a 70 µm strainer and collected in RPMI-1640 with 10% FBS and 10 mM HEPES on ice. The cell suspension was centrifuged at 500 × g for 5 minutes. Before flow cytometry, cells were diluted to 10⁶ cells per 100 μL and incubated with a blocking cocktail containing anti-mouse CD16/CD32 to prevent non-specific binding. Following blocking, cells were stained for surface markers described above for 10 minutes at room temperature.

Flow cytometric analysis was performed on BD LSRFortessa instrument (BD Biosciences) and analyzed using FACSDiva (BD Biosciences) software. All antibodies are listed in Supplementary Data 12. The FACS gating strategy is shown in Supplementary Fig. 10.

### Analysis of publicly available expression and clinical data

To analyze the expression of NAD metabolic enzymes in IBD samples, RNA-seq dataset GSE111889 was utilized^42^.

To analyze the expression of *NAMPT* and *NAPRT* in different human solid tumors in comparison with their respective normal tissues, TCGA data were downloaded from the UCSC Xena web portal. The expression values of *NAMPT* and *NAPRT* are HTSeq FPKM-UQ normalized values. Normal tissue samples taken from tissue adjacent to the tumor were used as normal tissue controls, and only a limited number of normal samples have been sequenced. Primary tumors matching adjacent normal tissues (some normal tissues might have two or more matched tumor samples) as well as all primary tumor samples with expression values were used in separate analyses. Based on the expression values in normal tissue samples, the expression of *NAMPT* or *NAPRT* in tumors were stratified into three categories: *Below*, in which tumor values were below the 25th percentile of the corresponding normal tissue data; *Normal*, in which tumor values were within the 25th to 75th percentile of the corresponding normal data; and *Above*, in which tumor values were above the 75th percentile of the corresponding normal data. Similar results were obtained in matched primary tumors and all primary tumors (Supplementary Data 6).

To analyze the expression of *NAMPT* and *NAPRT* in colonic polyps in comparison with normal colonic tissues, single-cell RNA-seq datasets from Becker et al^60^. and Chen et al^61^. were downloaded from public resources. Chen’s dataset was obtained from the CELLxGENE collection (HTAN VUMC: a48f5033-3438-4550-8574-cdff3263fdfd) and further processed using the Scanpy Python package. Becker’s dataset was downloaded from GEO (GSE201348) and processed using the Seurat R package. For both single-cell datasets, mean-based, sample-level pseudobulk counts were aggregated from the raw cell counts and then normalized to CPM (counts per million). The CPM expression values for the genes of interest were retrieved and compared between polyp and normal samples.

For association of NAPRT expression and cancer patients’ survival, RNAseq expression and clinical data for TCGA PanCancer datasets of colon, liver and kidney cancers were downloaded from https://www.cbioportal.org/^62,63^. Survival analysis was performed in GraphPad Prism v 9.5.1 using cutoffs of NAPRT expression that resulted in best separation of NAPRT-low and NAPRT-high samples for each dataset.

### Statistics and reproducibility

Values are presented as mean with either standard deviation (SD) or standard error of mean (SEM) from a minimum of three independent experiments or biological replicates, unless specified differently in the figure legend. To evaluate significant differences between the means, two-tailed, unpaired, non-parametric Mann-Whitney test was employed when sample size ≥4, while two-tailed, unpaired, Student’s t-test was utilized when sample size <4^64^. Statistical significance was set at p < 0.05. Differences between the means with more than two comparison groups were analyzed by either Kruskal-Wallis test or two-way ANOVA with correction for multiple comparisons by controlling the false discovery rate and adjusted p-values (q-values) were reported. For all figures, *p (or q)<0.05, **p (or q)<0.01, *** p (or q)<0.001, **** p (or q)<0.0001, n.s., not significant. The exact p values are included in the Source Data file. Data analyses were performed using Prism Software 10.0 (GraphPad) or Microsoft Office Excel (Version 16.79.1). No methods were applied to ascertain whether the data met the assumptions of the statistical approach (e.g., normal distribution test).

For animal studies, the sample size in each independent experiment was determined based on our prior experience and mouse availability. The combined sample size for the DSS-colitis study was estimated to achieve a 2-fold difference of colitis phenotypes (tissue damage scores or rectal bleeding scores) with 80% of power. The sample size for other phenotypes (metabolic changes or tumor burden) was estimated to achieve 30% difference with 80% of power. For gene expression analysis, the sample size was estimated to achieve a 2-fold difference in gene expression level with 80% of power. In vitro cell culture experiments were independently performed at least three times and similar results were observed. Each independent experiment was performed with at least three biological replicates; no explicit calculations were done to determine the sample size.

### Reporting summary

Further information on research design is available in the Nature Portfolio Reporting Summary linked to this article.

## Supplementary information

Supplementary Information

Description of Additional Supplementary Files

Supplementary Data 1

Supplementary Data 2

Supplementary Data 3

Supplementary Data 4

Supplementary Data 5

Supplementary Data 6

Supplementary Data 7

Supplementary Data 8

Supplementary Data 9

Supplementary Data 10

Supplementary Data 11

Supplementary Data 12

Reporting Summary

Transparent Peer Review file

## Source data

Source data

## Data Availability

The RNA-seq and scRNA-seq datasets of this study have been deposited to Gene Expression Omnibus under the following accession numbers: RNA-seq dataset of the AOM experiment: GSE271834 [https://www.ncbi.nlm.nih.gov/geo/query/acc.cgi?acc= GSE271834]RNA-seq dataset of the DSS-colitis experiment: GSE271250 [https://www.ncbi.nlm.nih.gov/geo/query/acc.cgi?acc= GSE271250]. scRNA-seq datasets of colon tissues: GSE271836 [https://www.ncbi.nlm.nih.gov/geo/query/acc.cgi?acc= GSE271836] scRNA-seq datasets of small intestinal tissues: GSE261216 [https://www.ncbi.nlm.nih.gov/geo/query/acc.cgi?acc= GSE261216], Metabolomics data of this study have been deposited to massive.ucsd.edu with the accession number MSV000100103, 10.25345/C56M33H4M. The detailed data of 277 identified or annotated metabolites are provided in Supplementary Data 1. LC-MS data for targeted measurement of mouse tissue NAD metabolites have been deposited to massive.ucsd.edu with the accession number MSV000100196, 10.25345/C5639KJ8W. The representative spectral peaks are provided in Supplementary Data 8. LC-MS parameters and representative spectral peaks from targeted measurement of NUA in mouse kidney are provided in Supplementary Data 9. The LC-MS raw data files for NAD metabolites in mouse plasma have been uploaded to metabolomicsworkbench.org under tracking ID 6868 (NA and NAM) and 6875 (NAR), 10.21228/M8BN9Z. The LC-MS data and representative spectral peaks are provided in Supplementary Data 10. Public datasets used in this study are: GSE111889. GSE201348. HTAN VUMC [https://cellxgene.cziscience.com/collections/a48f5033-3438-4550-8574-cdff3263fdfd]. TCGA PanCancer datasets: https://www.cbioportal.org/. Source data containing the exact p-values for all statistical tests are provided with this paper.

## Code availability

Scripts used for scRNA-seq analyses in this study are available with no restriction at github: https:github.com/JohnXu24/NAPRTKO_scRNA. Published software packages and pipelines are used for RNA-seq and scRNA-seq analyses. Detailed description of these data analyses is included in Methods.
