# Supplementary material for: NAPRT-mediated deamidated NAD biosynthesis enhances colon tissue resiliency and suppresses tumorigenesis
Source: Nat Commun. 2026 Feb 10;17:8651. doi: 10.1038/s41467-026-68998-w (PMC13490428; doi:10.1038/s41467-026-68998-w)
Supplement: Supplementary file 1 [file 41467_2026_68998_MOESM15_ESM.pdf]

nature portfolio

Corresponding author(s): Xiaoling Li

Last updated by author(s): Dec 6, 2025

Reporting Summary

Nature Portfolio wishes to improve the reproducibility of the work that we publish. This form provides structure for consistency and transparency in reporting. For further information on Nature Portfolio policies, see our Editorial Policies and the Editorial Policy Checklist.

Statistics

For all statistical analyses, confirm that the following items are present in the figure legend, table legend, main text, or Methods section.

n/a	Confirmed	
☐	☒	The exact sample size (*n*) for each experimental group/condition, given as a discrete number and unit of measurement
☐	☒	A statement on whether measurements were taken from distinct samples or whether the same sample was measured repeatedly
☐	☒	The statistical test(s) used AND whether they are one- or two-sided<br>*Only common tests should be described solely by name; describe more complex techniques in the Methods section.*
☒	☐	A description of all covariates tested
☐	☒	A description of any assumptions or corrections, such as tests of normality and adjustment for multiple comparisons
☐	☒	A full description of the statistical parameters including central tendency (e.g. means) or other basic estimates (e.g. regression coefficient) AND variation (e.g. standard deviation) or associated estimates of uncertainty (e.g. confidence intervals)
☐	☒	For null hypothesis testing, the test statistic (e.g. *F*, *t*, *r*) with confidence intervals, effect sizes, degrees of freedom and *P* value noted<br>*Give P values as exact values whenever suitable.*
☒	☐	For Bayesian analysis, information on the choice of priors and Markov chain Monte Carlo settings
☒	☐	For hierarchical and complex designs, identification of the appropriate level for tests and full reporting of outcomes
☒	☐	Estimates of effect sizes (e.g. Cohen's *d*, Pearson's *r*), indicating how they were calculated

Our web collection on statistics for biologists contains articles on many of the points above.

Software and code

Policy information about availability of computer code

Data collection

Flow cytometry: BD LSRFortessa instrument (BD Biosciences)
Luminescence measurements: LUMIstar OPTIMA
Immunofluorescence: Zeiss LSM 980, Zeiss LSM 880
Real-time PCR: CFX96 or CFX Opus 384 real-time PCR instruments (Bio-Rad)
Immunoblotting: LICOR Odyssey imaging system
Seahorse: Seahorse XFe24 Analyzer
RNA-seq: NOVAseq (Illumina)
scRNA-seq: 10x Genomics
Targeted LC-MS: Q Exactive Plus mass spectrometer (QE-MS, Thermo Scientific) interfaced with a Vanquish (Thermo Fisher) UHPLC system
Targeted LC-MS (NCSU platform): A Vanquish Horizon UHPLC system equipped with ACQUITY UPLC BEH Amide column (1 mm x 100 mm, 1.7 µm, Waters) coupled to Exploris 480 mass spectrometer (Thermo Fisher Scientific)
Untargeted metabolomics: ultra-high performance liquid chromatograph (VanquishTM Horizon UHPLC, Thermo Scientific) coupled to a high-resolution mass spectrometer (Orbitrap FusionTM Tribrid, Thermo Scientific)

Data analysis

Published software packages and pipelines for RNA-seq analyses: STAR (Version 2.6) with Gencode vM23 annotation- Reads alignment; featureCount (subread, Version 1.4.6)- quantification; Bioconductor package DESeq2- DEG idenfication; Ingenuity Pathway Analysis (IPA, QIAGEN, USA; Content version 60467501) and GSEA software (Version 4.1)- Pathway enrichment analysis. Detailed description of the RNA-seq data analyses is included in Methods.
Scripts used for scRNA-seq analysis in this study are available with no restrictions at github: https:github.com/JohnXu24/NAPRTKO_scRNA. There are no restrictions to access the codes. Published software packages and pipelines for scRNA-seq analysis include CellRanger 6.0.0 -

preprocession the sequencing data; R version 4.0.2 - R environment for data analysis; Seurat_4.0.3 - R Data Class Environment used throughout analyses, SNN graph clustering implementation; celda_1.4.7- Remove ambient RNA; uwot_0.1.8- Creation of UMAP embeddings in R; harmony_l.0 - Batch correction for single cell data; MAST_1.16.0 - Used for calling marker gene and differential expression genes; pheatmap_1.0.12 - Data visualization; reticulate_1.20 - used for runing Python implementations within R; clusterProfiler_3.18.1 - preranked GSEA analysis of silenced or depressed genes; SingleCellExperiment_1.12.0- R Data Class Environment used throughout analyses; https://biit.cs.ut.ee/gprofiler/gost- Pathway enrichment analysis. Detailed description of the scRNA-seq data analyses is included in Methods.
Image quantification: ImageJ (Fiji).
Seahorse: Wave software
Real-time PCR: Bio-Rad CFX Manager
Flow cytometry: FACSDiva (BD Biosciences)
Targeted LC-MS: the Qual Browser function in the Xcalibur software suite (Thermo Scientific)
Targeted LC-MS (NCSU platform): Skyline was used for MS2-based quantification, while the FrameSeed function in SIEVE was applied for MS1-based quantification.
Untargeted metabolomics: Data files (.raw) were processed with Compound Discoverer 3.3.0.550 (ThermoFisher Scientific)
All other routine data analysis: Microsoft Excel and GraphPad Prism

For manuscripts utilizing custom algorithms or software that are central to the research but not yet described in published literature, software must be made available to editors and reviewers. We strongly encourage code deposition in a community repository (e.g. GitHub). See the Nature Portfolio guidelines for submitting code & software for further information.

Data

Policy information about availability of data

All manuscripts must include a data availability statement. This statement should provide the following information, where applicable:

- Accession codes, unique identifiers, or web links for publicly available datasets
- A description of any restrictions on data availability
- For clinical datasets or third party data, please ensure that the statement adheres to our policy

The RNA-seq and scRNA-seq datasets of this study have been deposited to Gene Expression Omnibus under the following accession numbers:
RNA-seq dataset of the AOM experiment:
GSE271834 [https://www.ncbi.nlm.nih.gov/geo/query/acc.cgi?acc= GSE271834]
RNA-seq dataset of the DSS-colitis experiment:
GSE271250 [https://www.ncbi.nlm.nih.gov/geo/query/acc.cgi?acc= GSE271250]
scRNA-seq datasets of colonic tissues:
GSE271836 [https://www.ncbi.nlm.nih.gov/geo/query/acc.cgi?acc= GSE271836]
scRNA-seq datasets of small intestinal tissues:
GSE261216 [https://www.ncbi.nlm.nih.gov/geo/query/acc.cgi?acc= GSE261216]

Metabolomics data of this study have been deposited to massive.ucsd.edu with the accession number MSV000100103, https://doi.org/doi:10.25345/C56M33H4M. The detailed data of 277 identified or annotated metabolites are provided in Supplementary Data 1.

The LC-MS parameters and representative spectral peaks from targeted measurement of NUA in mouse kidney are provided in Supplementary Data 9.

The LC-MS raw data files for NAD metabolites in mouse plasma have been uploaded to metabolomicsworkbench.org under tracking ID 6868 (NA and NAM) and 6875 (NAR), http://dx.doi.org/10.21228/M8BN9Z. The LC-MS data and representative spectral peaks are provided in Supplementary Data 10.

Public datasets used in this study are:
GSE111889 [https://www.ncbi.nlm.nih.gov/geo/query/acc.cgi?acc=GSE111889]
GSE201348 [https://www.ncbi.nlm.nih.gov/geo/query/acc.cgi?acc=GSE201348]
HTAN VUMC [https://cellxgene.cziscience.com/collections/a48f5033-3438-4550-8574-cdff3263fdfd]
TCGA PanCancer datasets: https://www.cbioportal.org/

SSource Data containing the exact p-values for all statistical tests are provided with this paper.

Research involving human participants, their data, or biological material

Policy information about studies with human participants or human data. See also policy information about sex, gender (identity/presentation), and sexual orientation and race, ethnicity and racism.

Reporting on sex and gender	*Use the terms sex (biological attribute) and gender (shaped by social and cultural circumstances) carefully in order to avoid confusing both terms. Indicate if findings apply to only one sex or gender; describe whether sex and gender were considered in study design; whether sex and/or gender was determined based on self-reporting or assigned and methods used.*<br>*Provide in the source data disaggregated sex and gender data, where this information has been collected, and if consent has been obtained for sharing of individual-level data; provide overall numbers in this Reporting Summary. Please state if this information has not been collected.*<br>*Report sex- and gender-based analyses where performed, justify reasons for lack of sex- and gender-based analysis.*
Reporting on race, ethnicity, or other socially relevant groupings	*Please specify the socially constructed or socially relevant categorization variable(s) used in your manuscript and explain why they were used. Please note that such variables should not be used as proxies for other socially constructed/relevant variables (for example, race or ethnicity should not be used as a proxy for socioeconomic status).*<br>*Provide clear definitions of the relevant terms used, how they were provided (by the participants/respondents, the*

	researchers, or third parties), and the method(s) used to classify people into the different categories (e.g. self-report, census or administrative data, social media data, etc.)<br>*Please provide details about how you controlled for confounding variables in your analyses.*
Population characteristics	*Describe the covariate-relevant population characteristics of the human research participants (e.g. age, genotypic information, past and current diagnosis and treatment categories). If you filled out the behavioural & social sciences study design questions and have nothing to add here, write "See above."*
Recruitment	*Describe how participants were recruited. Outline any potential self-selection bias or other biases that may be present and how these are likely to impact results.*
Ethics oversight	*Identify the organization(s) that approved the study protocol.*

Note that full information on the approval of the study protocol must also be provided in the manuscript.

Field-specific reporting

Please select the one below that is the best fit for your research. If you are not sure, read the appropriate sections before making your selection.

[x] Life sciences [ ] Behavioural & social sciences [ ] Ecological, evolutionary & environmental sciences

For a reference copy of the document with all sections, see nature.com/documents/nr-reporting-summary-flat.pdf

Life sciences study design

All studies must disclose on these points even when the disclosure is negative.

Sample size	For animal studies, the sample size in each independent experiment was determined based on our prior experience and mouse availability. The combined sample size for DSS-colitis study was estimated to achieve 2-fold difference of colitis phenotypes (tissue damage scores or rectal bleeding scores) with 80% of power. The sample size for other phenotypes (metabolic changes or tumor burden) was estimated to achieve 30% difference with 80% of power. For gene expression analysis, the sample size was estimated to achieve 2-fold difference of gene expression level with 80% of power. In vitro cell culture experiments were independently performed at least three times and similar results were observed. Each independent experiment was performed with at least three biological replicates, no explicit calculations were done to determine the sample size.
Data exclusions	For all in vivo experiments, outlier samples that fall below Q1 − 3.0 IQR or above Q3 + 3.0 IQR were removed. For Seahorse analysis, all negative values (due to the instability of the equipment) were also removed.
Replication	For NAD metabolite quantification in mice, 3-5 mice/group were used. For AOM-induced DNA damage experiment, 7-9 mice/group were used; for AOM-DSS CRC model, 12-14 mice/group were used. For tumor number/burden studies in Apcmin+/- mice, 17-20 mice/group were used. For DSS-colitis, 6-8 mice/group were used. For aging tumor study, 12 WT and 19 KO males and 11 WT and 5 KO females were used. For symptom-free survival study, 56 WT and 50 KO males and 41 WT and 36 KO females were used. For scRNA-seq experiment, single cell suspension from 3 mice/group was combined for each sample. Each in vitro cell culture experiment was performed with at least three biological replicates. All attempts at replication were successful.
Randomization	No randomization methods were utilized for this study. Mice in all animal experiments were randomized to experimental groups.
Blinding	For in vitro experiments, blinding was not performed because these assays were designed and conducted by a single investigator. For animal studies, administration of experimental compounds was performed blinded to genotypes. For the analysis of -omics data, the bioinformaticians were blinded to sample allocation in the initial analyses and sample processing steps. They were informed about the sample labels to enable final supervised comparisons. The LC-MS analysis was blinded to sample allocation. Pathological evaluation was performed by a single board-certified pathologist and was not blinded.

Reporting for specific materials, systems and methods

We require information from authors about some types of materials, experimental systems and methods used in many studies. Here, indicate whether each material, system or method listed is relevant to your study. If you are not sure if a list item applies to your research, read the appropriate section before selecting a response.

Materials & experimental systems

n/a	Involved in the study	
[ ]	[x]	Antibodies
[ ]	[x]	Eukaryotic cell lines
[x]	[ ]	Palaeontology and archaeology
[ ]	[x]	Animals and other organisms
[x]	[ ]	Clinical data
[x]	[ ]	Dual use research of concern
[x]	[ ]	Plants

Methods

n/a	Involved in the study	
[x]	[ ]	ChIP-seq
[ ]	[x]	Flow cytometry
[x]	[ ]	MRI-based neuroimaging

Antibodies

Antibodies used

Rabbit polyclonal anti-NAPRT1 (NAPRT#1) Proteintech cat# 13549-1-AP N/A 00011458 1:1000 For WB; 1:200 For IF
Mouse monoclonal anti-NAPRT1 (NAPRT#2) Abcam cat# ab211529 CL0665 GR3403091-10 1:1000-1:3000 For WB; 1:200 For IF
Rabbit anti-Phospho-Histone H2A.X (Ser139) Cell Signaling Technology cat# 9718 20E3 21 1:500
Anti-pan-ADP-ribose binding reagent Sigma-Aldrich cat# MABE1016 N/A 3921912 1:1000
Mouse monoclonal anti-PAR (10H) Sigma-Aldrich cat# AM80 10H 4244186 1:1000
Mouse monoclonal anti-Actin Sigma-Aldrich cat# MAB1501 C4 4158903 1:10000
Rabbit anti-PARP Cell Signaling Technology cat# 9542 N/A 15 1:1000
Mono-ADP-Ribose antibody | AbD33205 BIO-RAD cat# HCA355 AbD33205 162189 1:1000
Anti-Mouse CD16/CD32 (Mouse BD Fc Block™) BD Biosciences cat# 553142 2.4G2 3101344 1:50
CD45 Monoclonal Antibody (30-F11) Invitrogen cat# 48-0451-82 30-F11 2640501 1:400
BD OptiBuild™ BV510 Hamster Anti-Mouse CD11c BD Biosciences cat# 744178 N418 4211113 1:400
Brilliant Violet 711™ anti-mouse CD8a Antibody BioLegend cat# 100759 53-6.7 B424666 1:200
FITC anti-mouse CD3 Antibody BioLegend cat# 100204 17A2 B415732 1:100
PerCP/Cyanine5.5 anti-mouse/human CD11b Antibody BioLegend cat# 101227 M1/70 B353754 1:400
PE/Cyanine7 anti-mouse I-A/I-E Antibody BioLegend cat# 107629 M5/114.15.2 B410855 1:1500
Ly-6G Monoclonal Antibody (1A8-Ly6g), PE, eBioscience™ Invitrogen cat# 12-9668-82 1A8-Ly6g 2896812 1:1500
PE/Dazzle™ 594 anti-mouse CD115 (CSF-1R) Antibody BioLegend cat# 135527 AFS98 B409633 1:800
F4/80 Monoclonal Antibody (BM8), PE-Cyanine5, eBioscience™ Invitrogen cat# 15-4801-82 BM8 2608838 1:400
BD Pharmingen™ APC Rat Anti-Mouse CD19 BD Biosciences cat# 550992 1D3 4080467 1:1500
CD4 Monoclonal Antibody (RM4-5), Alexa Fluor™ 700, eBioscience™, Invitrogen™ Invitrogen cat# 56-0042-82 RM4-5 2763974 1:800
APC/Cyanine7 anti-mouse Ly-6C Antibody BioLegend cat# 128025 HK1.4 B417977 1:1500
anti-Nampt (Visfatin/PBEF), mAb (OMNI379) AdipoGen Life Sciences cat# AG-20A-0034-C050 OMNI379 A25931406F 1:2000

Validation

NAPRT and NAMPT antibodies were validated by manufacturers (see the links below), and further validated using their respective KO cell lines/mice in this study (Supplementary Figure 1d, Supplementary Figure 2g, and Supplementary Figure 3c).

All other routinely used antibodies for FACS, immunoblotting, and immunofluorescence staining were all validated by manufacturers. Here are links to the respective manufacturer websites:
Rabbit polyclonal anti-NAPRT1 (NAPRT#1) https://www.ptglab.com/products/NAPRT1-Antibody-13549-1-AP.htm?srsltid=AfmBOoq28_VmsvEzZVVYa_CEZ77m4T9rCVjeVgkiEPKmAOq9483YXDdb
Mouse monoclonal anti-NAPRT1 (NAPRT#2) https://www.abcam.com/en-us/products/primary-antibodies/naprt1-antibody-cl0665-ab211529
Rabbit anti-Phospho-Histone H2A.X (Ser139) https://www.cellsignal.com/products/primary-antibodies/phospho-histone-h2a-x-ser139-20e3-rabbit-monoclonal-antibody/9718?utm_term&utm_campaign=SO-Products-PrimaryAntibodies&utm_source=adwords&utm_medium=ppc&hsa_acc=8625036580&hsa_cam=21865491299&hsa_grp=167649643577&hsa_ad=730824565110&hsa_src=g&hsa_tgt=dsa-2373170399499&hsa_kw&hsa_mt&hsa_net=adwords&hsa_ver=3&gad_source=1&gad_campaignid=21865491299&gbraid=0AAAAAD_viZJopqDkYStSOrKV6STR03qpm&gclid=Cj0KCQiArt_JBhCTARIsADQZaykJFwFTZn47Mw5y7I2q-m9QuCqxpgXfNyuKDqn-Gkz5-xeCL2vrNrUaArLkEALw_wcB
Anti-pan-ADP-ribose binding reagent https://www.sigmaaldrich.com/US/en/product/mm/mabe1016?srsltid=AfmBOoqZw62Si1cVXLs5iX7Vpd718DBAwPnGX_T2GTFX-DAxqaA5de2J
Mouse monoclonal anti-PAR (10H) https://www.antibodyregistry.org/AB_2155072
Mouse monoclonal anti-Actin https://www.sigmaaldrich.com/US/en/product/mm/mab1501?srsltid=AfmBOophNIrD6wWdwHySu7rxCDKFplxso6v9gcWSenE3MOMUJpXOIkCQ
Rabbit anti-PARP https://www.cellsignal.com/products/primary-antibodies/parp-antibody/9542?srsltid=AfmBOoontZfhRircb8hqtS-RMMQfnQ9MksTqpFQvWafIN9hg8rVuZeAs
Mono-ADP-Ribose antibody | AbD33205 https://www.bio-rad-antibodies.com/monoclonal/human-mono-adp-ribose-antibody-abd33205-hca355.html?f=purified
Anti-Mouse CD16/CD32 (Mouse BD Fc Block™) https://www.bdbiosciences.com/en-at/products/reagents/western-blotting-and-molecular-reagents/purified-rat-anti-mouse-cd16-cd32-mouse-bd-fc-block.553142?tab=product_details
CD45 Monoclonal Antibody (30-F11) https://www.thermofisher.com/antibody/product/CD45-Antibody-clone-30-F11-Monoclonal/48-0451-82
BD OptiBuild™ BV510 Hamster Anti-Mouse CD11c https://www.bdbiosciences.com/en-us/products/reagents/flow-cytometry-reagents/research-reagents/single-color-antibodies-ruo/bv510-hamster-anti-mouse-cd11c.744178?tab=antibody_details
Brilliant Violet 711™ anti-mouse CD8a Antibody https://www.biolegend.com/en-ie/products/brilliant-violet-711-anti-mouse-cd8a-antibody-7926?GroupID=BLG279
FITC anti-mouse CD3 Antibody https://www.biolegend.com/de-de/products/fitc-anti-mouse-cd3-antibody-45?GroupID=BLG6732
PerCP/Cyanine5.5 anti-mouse/human CD11b Antibody https://www.biolegend.com/en-ie/sean-tuckers-tests/percp-cyanine5-5-anti-mouse-human-cd11b-antibody-4257?GroupID=BLG10552
PE/Cyanine7 anti-mouse I-A/I-E Antibody https://www.biolegend.com/de-at/clone-search/pe-cyanine7-anti-mouse-i-a-i-e-antibody-6136?GroupID=BLG11931
Ly-6G Monoclonal Antibody (1A8-Ly6g), PE, eBioscience™ https://www.thermofisher.com/antibody/product/Ly-6G-Antibody-clone-1A8-Ly6g-Monoclonal/12-9668-82
PE/Dazzle™ 594 anti-mouse CD115 (CSF-1R) Antibody https://www.biolegend.com/ja-jp/products/pe-dazzle-594-anti-mouse-cd115-csf-1r-antibody-12484
F4/80 Monoclonal Antibody (BM8), PE-Cyanine5, eBioscience™ https://www.thermofisher.com/antibody/product/F4-80-Antibody-clone-BM8-Monoclonal/15-4801-82
BD Pharmingen™ APC Rat Anti-Mouse CD19 https://www.bdbiosciences.com/en-us/products/reagents/flow-cytometry-reagents/

research-reagents/single-color-antibodies-ruo/apc-rat-anti-mouse-cd19.550992?tab=product_details
CD4 Monoclonal Antibody (RM4-5), Alexa Fluor™ 700, eBioscience™, Invitrogen™ https://www.thermofisher.com/antibody/product/CD4-Antibody-clone-RM4-5-Monoclonal/56-0042-82
APC/Cyanine7 anti-mouse Ly-6C Antibody https://www.biolegend.com/en-ie/products/apc-cyanine7-anti-mouse-ly-6c-antibody-6758?GroupID=BLG5853
anti-Nampt (Visfatin/PBEF), mAb (OMNI379) https://www.fishersci.com/shop/products/nampt-mab-50ug-1/502052821

Eukaryotic cell lines

Policy information about cell lines and Sex and Gender in Research

Cell line source(s)	CRC119 human female colon cancer cell line, a gift from Dr. David Hsu at Duke University
Authentication	CRC119 cells were derived from a female CRC patient at Duke University. They are not authenticated using fingerprinting.
Mycoplasma contamination	Cells were tested negative for the mycoplasma contamination by the Quality Assurance Laboratory of the National Institute of Environmental Health Sciences.
Commonly misidentified lines (See ICLAC register)	No commonly misidentified lines were used.

Animals and other research organisms

Policy information about studies involving animals; ARRIVE guidelines recommended for reporting animal research, and Sex and Gender in Research

Laboratory animals	Mouse, NAPRT KO and WT control mice on the C57BL/6J background, generated by NIEHS Mouse Model core facility, males and females, adults (starting from 6-8 weeks old).<br>Mouse, WT/Apcmin+/- and NAPRT KO/Apcmin+/-, generated by breeding WT and NAPRT KO mice with Apcmin+/- from the Jackson Laboratory (C57BL/6J-ApcMin/J, Jax strain #: 002020), males and females, adults (staring from 6-8 weeks old).<br>Apcmin+/- breeders (Jax: 002020), males, 6-8 weeks old.
Wild animals	The study did not involve wild animals
Reporting on sex	Both males and females were analyzed in experiments, and sex-specific findings were observed and reported.
Field-collected samples	The study did not involve samples collected from the field.
Ethics oversight	All experiments involving animals were approved by the Institutional Animal Care and Use Committee of the National Institute of Environmental Health Sciences.

Note that full information on the approval of the study protocol must also be provided in the manuscript.

Plants

Seed stocks	*Report on the source of all seed stocks or other plant material used. If applicable, state the seed stock centre and catalogue number. If plant specimens were collected from the field, describe the collection location, date and sampling procedures.*
Novel plant genotypes	*Describe the methods by which all novel plant genotypes were produced. This includes those generated by transgenic approaches, gene editing, chemical/radiation-based mutagenesis and hybridization. For transgenic lines, describe the transformation method, the number of independent lines analyzed and the generation upon which experiments were performed. For gene-edited lines, describe the editor used, the endogenous sequence targeted for editing, the targeting guide RNA sequence (if applicable) and how the editor was applied.*
Authentication	*Describe any authentication procedures for each seed stock used or novel genotype generated. Describe any experiments used to assess the effect of a mutation and, where applicable, how potential secondary effects (e.g. second site T-DNA insertions, mosiacism, off-target gene editing) were examined.*

Flow Cytometry

Plots

Confirm that:

- [x] The axis labels state the marker and fluorochrome used (e.g. CD4-FITC).
- [x] The axis scales are clearly visible. Include numbers along axes only for bottom left plot of group (a 'group' is an analysis of identical markers).
- [x] All plots are contour plots with outliers or pseudocolor plots.
- [x] A numerical value for number of cells or percentage (with statistics) is provided.

Methodology

Sample preparation	Blood samples were collected with EDTA coated tubes. Red blood cells were lysed with ACK lysis buffer at room temperature for 10 min. The collected lymphocytes were incubated with anti-mouse CD16/32 at room temperature for 10 min to block the IgG Fc receptors. Expression of surface markers was detected by simultaneously staining with the following antibodies (eFluor 450 anti-mouse CD45, APC-Cy7 anti-mouse CD4, FITC anti-mouse CD4, FITC anti-mouse CD3, PE-Cy7 anti-mouse CD3, FITC anti-mouse CD8, PerCP-Cy™5.5 anti-mouse CD8, anti-mouse CD11b, APC anti-mouse CD19, BV510 anti-mouse CD11c, PE anti-mouse Ly-6G, PE-Cyanine5 anti-mouse F4/80, APC/Cyanine7 anti-mouse Ly-6C) and LIVE/DEAD fixable Aqua dead cell stain kit (ThermoFisher, Cat. # L34957) at room temperature for 15 min followed by flow cytometry.<br><br>For flow cytometry analysis of colonic immune cells, the colon was flushed with ice-cold PBS (Mg^{2+}/Ca^{2+}-free) and remove surrounding fat and mesentery. Then opened longitudinally and transferred to a 50 mL Falcon tube containing 20 mL ice-cold PBS. The tissue was shaken vigorously five times in fresh PBS until the intestines appeared clean and pale (white/pink) Cut the colon into 0.5–1 cm pieces and incubated in 10 mL of pre-digestion buffer (Mg^{2+}/Ca^{2+}-free HBSS with 2% FBS, 10 mM HEPES, 2 mM EDTA, and 1 mM DTT) at 37°C in a shaking incubator (250 rpm) for 15 minutes then wash three times with cold PBS. To digest the remaining tissue, it was incubated in 10 mL RPMI-1640 containing 5% FBS, 10 mM HEPES, 0.6 mg/mL Collagenase IV, 0.4 mg/mL Collagenase I, 1 mg/mL Dispase II, and 50 µg/mL DNase I. This digestion was performed at 37°C with shaking at 250 rpm for 15 minutes. After vortexing for 10 seconds, cells were filtered through a 70 µm strainer and collected in RPMI-1640 with 10% FBS and 10 mM HEPES on ice. The cell suspension was centrifuged at 500 × g for 5 minutes. Before flow cytometry, cells were diluted to 10^6 cells per 100 µL and incubated with a blocking cocktail containing anti-mouse CD16/CD32 to prevent non-specific binding. Following blocking, cells were stained for surface markers 10 minutes at room temperature.
Instrument	Flow cytometric analysis was performed on BD LSRFortessa instrument (BD Biosciences).
Software	FACSDiva (BD Biosciences).
Cell population abundance	Initial forward scatter versus side-scatter gates were carefully adjusted by backgating on live CD45+ populations to include all cells and exclude debris. Stringent exclusion of dead cells and doublets was performed before gating for immune cells (CD45 +) .
Gating strategy	Myeloid cells were gated by live CD45+CD11b+ and subsetted into neutrophils (Ly6G+), Ly6G- cells, dendritic cells (CD11c +MHCII+). Ly6G- cells were further subsetted into macrophages (Ly6ClowF4/80+) and monocytes (Ly6ChighCD115+). T cells were gated by live CD45+CD11b-CD3+ and subsetted into CD4 T (CD4+) cells or CD8 T (CD8+) cells. B cells were gated by live CD45+CD11b-CD19+ cells.

☒ Tick this box to confirm that a figure exemplifying the gating strategy is provided in the Supplementary Information.
