# Supplementary material for: NAPRT-mediated deamidated NAD biosynthesis enhances colon tissue resiliency and suppresses tumorigenesis
Source: Nat Commun. 2026 Feb 10;17:8651. doi: 10.1038/s41467-026-68998-w (PMC13490428; doi:10.1038/s41467-026-68998-w)
Supplement: Supplementary file 2 [file 41467_2026_68998_MOESM16_ESM.pdf]

nature portfolio

Peer Review File

NAPRT-mediated deamidated NAD biosynthesis enhances colon tissue resiliency and suppresses tumorigenesis

Corresponding Author: Dr Xiaoling Li

This file contains all reviewer reports in order by version, followed by all author rebuttals in order by version.

Version 0:

Reviewer comments:

Reviewer #1

(Remarks to the Author)
In this paper, Wu X et al. have revealed that Naprt deficiency promotes two major processes in colon tumorigenesis: tumor initiation (DNA damage) and promotion (inflammation). They propose that the increased DNA damage in the Naprt-KO mice is likely due to a reduced PARP activity associated with lower NAD levels in epithelial cells. Given these results, a phenotype that Naprt deficiency leads to an increased incidence of colon tumors is convincing. These are novel and interesting findings. However, there are two major concerns regarding this paper that will require many additional efforts, as outlined below;

The first concern relates to the inverse correlation between Naprt expression and prognosis in several cancers (Fig 6J-L). As noted above, data regarding increased tumor formation in Naprt-KO mice is convincing. However, this does not imply that tumors with Naprt deficiency exhibit higher malignancy or more therapeutic resistance. After diagnosis, the prognosis of cancer patients typically worsens with increasing malignancy and/or treatment resistance of tumor cells. Importantly, tumor cells with DNA repair defects often show higher sensitivity to chemotherapy and radiation therapy, which can extend patient survival. Thus, even if Naprt-low cancers show worse prognoses compared to Naprt-high cancers, it does not seem reasonable to attribute this to an impaired DNA repair ability associated with Naprt deficiency.
Another major issue is that this study overlooks recent important findings on systemic NA metabolism. In brief, NA absorbed from the intestine is actively converted to nicotinic acid riboside (NAR) NAPRT-dependently, then released into the circulation (Nomura, Nat Commun ’23. PMID: 38092728). Their paper reports that (1) the blood concentration of NA is much lower than that of NAR, and (2) Naprt deficiency significantly decreases the blood NAR levels in mice. These findings are critical to interpret the data presented in this paper. For example, in the culture experiments of this study, the authors added NA at a concentration of 100 µM, which is likely supraphysiological by a factor of 1000 or more in most organs. Direct fueling form the dietary NA or NA converted from Nam by gut microbiota may result in higher NA concentrations in the colon epithelium compared to the circulation. However, there is little direct evidence that NAD synthesis using NA as a substrate is active in other organs. Instead, it is questionable whether the proposed mechanism in this paper applies beyond the intestinal epithelium. Given the NA to NAR conversion, many of author’s claim in this paper remain speculative.

Besides the two major issues mentioned above, the following points require significant attention:
Figs 1-3: The authors added NA at a final concentration of 100 µM in many experiments in vitro. A theoretical basis for this concentration setting must be provided. Additionally, It would be better to present results with lower concentrations of NA for comparison.
Fig 2: The differences in PARylation and DNA damage between the parental and NAPRT-KO cells are relatively small. Therefore, add-back experiments should be conducted. Alternatively, experiments with multiple KO clones should be performed. Additionally, gH2AX levels should be examined by WB for a clearer comparison.
Fig 3: The NAD levels in various organs of Naprt-KO mice under steady-state conditions (without Nam load) should be reported.
Fig 4: The reason for administering additional NA through drinking water, given that NA is already provided in the dietary chow, should be clarified. Panels C-E are underwhelming, and most of them could be moved to supplementary items.
Fig 6G: The experiment appears to be very important, but the method section lacks essential details. Were comparisons made using littermates? Were events (e.g., mortality) monitored up to the time of inspection at 24 months? These are critical details for interpreting the data. Moreover, the results should be presented separately for male and female mice.

The following statements appear overly speculative or exaggerated:
Line 160-1: "suggesting that NAD pool maintained by … … DNA repair."
Line 185: "in gut epithelial cells"
Line 186-8: "despite accumulation of more severe DNA … … the surviving cells."
Line 213: "NAPRT-mediated deamidated NAD … … all tested tissues."
Line 218-20: "deamidated biosynthesis is a major route of … … and lung."
Line 296: "NAPRT deficiency is associated with an increased sensitivity to IBD."
Line 377: "NAPRT is believed to be oncogenic."
Line 394-5: "These results indicate that … … in both mice and humans."

The reasoning behind the statements in lines 381-8 and lines 401-3 is difficult to follow.

Reviewer #2

(Remarks to the Author)
The study by Wu et al. explores how the Preiss-Handler pathway in NAD biosynthesis, mediated by NAPRT, impacts NAD metabolism, DNA repair, and tumorigenesis in the gut. Key findings highlight the role of NAPRT in sustaining NAD levels across tissues such as the liver, colon, and pancreas, and the consequences of NAPRT deficiency on NAD synthesis and tissue vulnerability to NAD-depleting stress.

In NAPRT KO mice, NAD synthesis is significantly compromised under DNA damage conditions, like those induced by AOM treatment, leading to inadequate metabolic and DNA repair responses, heightened inflammation, and increased gut cell damage. This dysfunction predisposes NAPRT KO mice to more severe colitis and tumorigenesis, resulting in a higher tumor burden, advanced adenomas, and upregulated inflammatory genes, underscoring the critical function of NAPRT-mediated NAD synthesis in safeguarding gut tissue from inflammation and cancer progression.

This is a very well written paper, that provides important insights into the role of the Preiss-Handler pathway in NAD biosynthesis, particularly in the context of NAPRT and its impact on DNA repair and tumorigenesis. However, it lacks novelty in certain aspects, as highlighted in major concerns, particularly point 5. The pioneering study on the role of NAD+ in tumorigenesis by Tummala et al. (2014) as well as the controversial work by Lee et al. (2022) should be cited, discussed, and integrated into the context of the manuscript, supported by experimental evidence. Additionally, several key issues outlined below need to be addressed before this manuscript is considered suitable for publication.Once these issues are addressed, this manuscript could make a valuable contribution to the field.

Major concerns

1. The connection between NAD+ depletion and DNA damage is well-established, as shown in pivotal studies like Tummala et al. (2014) and Gomes et al. (2016), whose papers have not been cited but should. However, in their study, authors should mechanistically provide more insights into how NAPRT KO leads to DNA damage. This could result from decreased PARP activity, inhibition of Sirtuins, reduced dNTP synthesis, or perturbation in alkylation routes, all pathways where NAD+ plays a crucial role. To clarify this, the authors should measure the activities of PARP, Sirtuins, and dNTP synthesis in the NAPRT KO context. These assays would provide insight into the specific mechanisms by which NAD+ depletion leads to genotoxic stress and tumorigenesis in their model. Furthermore, performing rescue experiments, such as supplementing precursors to restore NAD+ levels or enhancing activity in the affected pathways, would significantly strengthen the study's conclusions regarding the role of NAD+ metabolism in DNA damage and cancer progression. Rescue experiments with dNTPs, or using PARP inhibitors are also requested.

2. A key concern is that while NAMPT depletion results in cell death, NAPRT KO does not induce such effects, in particular because the medium is supplemented with NAM. Likewise, NAPRT KO mice develop normally without obvious phenotypes, whereas NAMPT KO mice are embryonically lethal. The authors conclude that the deamidated NAD biosynthesis pathway is non-essential for survival, development, or normal cellular function. However, the underlying reason for this disparity remains unclear. Are NAD+ levels significantly more reduced to be completely abolished in NAMPT KO compared to NAPRT KO, thereby leading to extensive DNA damage, cell death, and catastrophic outcomes in NAMPT KO but not in NAPRT KO? Could it be that NAD+ depletion is less severe in NAPRT KO, allowing partial compensation through NAD+ synthesis from tryptophan catabolism? Notably QPRT is expressed in human colons and therefore this pathway might still be active despite the enzymes are not highly expressed in mice. Resolving these questions is crucial, and the study would benefit from comparative NAD+ quantification between NAMPT and NAPRT KO models, along with an analysis of compensatory metabolic pathways.

3. The authors claim that their scRNA-seq analysis reveals distinct cell-type expression patterns for NAMPT and NAPRT in the small intestine and colon, with NAMPT being ubiquitously expressed across all cell types, particularly in gut immune cells, while NAPRT is relatively enriched in the epithelial compartments (Figure 1A and S1B). However, this is not entirely accurate, as NAPRT also appears to be expressed in immune cells. Thus, a complete NAPRT knockout could indeed affect NAD+ levels in immune cells, potentially impacting their functional role in reducing tumorigenesis. A conditional knockout mouse model would have been a more suitable approach to target specific cell types for this study. By restricting the NAPRT knockout to particular tissues or cell types, the authors could better assess the cell-type-specific effects of NAD+ depletion on tumorigenesis. Alternatively, the authors could utilize a xenograft or patient-derived xenograft model (that will give some clinicl relevance to the study), comparing NAPRT knockout with NAMPT knockout, to assess the tumor-inducing potential in

immunodeficient mice.

4. In line with the previous comment, the authors should provide a more comprehensive explanation of their results addressing inflammation, in particular when using the DSS-induced colitis models. Reduction of NAD+ levels in their Naprt KO model induces DNA damage and this is well-known to be linked to tumour formation, especially in epithelial cells, but the connection between the reduction of NAD+ levels with inflammation is not clear. Why the reduction of NAD+ levels leads to more inflammation? Which cells contribute to this enhanced sensitivity in their KO mice? Which are the cellular pathways important for this process? Perhaps an immune cell characterization in the presence of DSS in NAPRT KO mice should be provided via scRNA seq of isolated CD45+ cells. The conditional KO mouse model would have solved these questions. We do understand that this is not possible at this stage, but authors could explored alternative experiments such as PDX or/and xenografts studies in immunodeficient mice to circumvent this.

5. A rescue experiment using nicotinamide riboside (NR) both in vitro (Figure 1) and in vivo to counteract the effects of NAPRT depletion on tumorigenesis would be highly valuable. For tumorigenesis, NR should be implemented in prevention and in therapy once tumors are formed to better understand the pro- or anti-tumorigenic effects of NAPRT KO. This experiment could provide direct evidence for the potential therapeutic role of NR in restoring NAD+ levels and mitigating the negative effects of NAD+ depletion on DNA repair, inflammation, and tumorigenesis. Including such data would significantly strengthen the manuscript by demonstrating the feasibility of NR as a potential intervention for restoring NAD+ homeostasis and reducing cancer progression in the context of NAPRT deficiency.

6. While this study introduces the first NAPRT KO mouse model to explore the role of the Preiss-Handler pathway in tumorigenesis, the concept of NAD+ depletion in DNA damage and cancer is not novel. The authors overlook the foundational work by Tummala et al., published in Cancer Cell in 2014, which first demonstrated the significance of NAD+ depletion in tumorigenesis. Additionally, the study by Lee et al., published in Cancer Discovery in 2022, has been ignored. In their work, the authors showed that APC-deficient colorectal cancer models were susceptible to TDO2 depletion or pharmacological inhibition, leading to NAD+ depletion, impaired cancer cell proliferation, and enhanced antitumor immune responses. These important studies should be cited, discussed, and integrated into the context of the current manuscript through experimental evidence. One possible explanation to the study of Lee et al. is that since APC deletion drives cell proliferation, cells might rely on increased NAD+ from de novo synthesis to initiate DNA synthesis and repair pathways. Without the support of the NAPRT-NAD+ arm, APC-deleted cells may not survive. Therefore, it is crucial to measure NAD+ levels under different conditions in order to determine how the depletion of NAPRT affects cellular NAD+ metabolism, especially in the context of APC deletion. This would help clarify whether NAPRT-mediated NAD+ synthesis is essential for the survival and function of APC-deleted cells and further elucidate the role of NAD+ in DNA repair and cell survival under genotoxic stress.

7. Figure 2D is difficult to understand, and the data interpretation lacks clarity. The authors claim that STF treatment sensitizes cells to MMS-induced cell death by comparing WT MMS+STF to WT MMS alone. However, when discussing the inactivation of deamidated synthesis in KO cells, the authors compare KO MMS to WT MMS instead, which seems inconsistent. According to Figure 2C, KO cells appear to have more DNA damage; therefore, in Figure 2D, MMS treatment alone would be expected to result in fewer colonies in KO cells compared to WT, but this outcome is not shown. Furthermore, if the comparison in KO cells were done similarly to WT cells—that is, MMS+STF vs. MMS alone—a similar observation (fewer colonies with STF treatment) would likely emerge. The precise methodology of this experiment, particularly the comparisons drawn between groups, needs more explanation.

Minor points

1. Some data described in the text is not shown in the figures (e.g. Figure 1 is missing to show cell morphology and proliferation rates).

2. Some panels of the figures are not even mentioned in the text (e.g. Figure 2A). All data shown in the figures must be discussed.

3. Figure legends contain explanations already present in the results section. They should be more descriptive and summarized.

4. Full uncut immunoblots should not be added in the supplementary figures (Fig. S1D, Fig. S2B).

5. In lines 141-142, the authors claim that '' NAPRT deficiency impairs cellular metabolism and increases the sensitivity of gut epithelial cells to NAMPT inhibition induced NAD depletion and subsequence cell death''. However, the authors should provide an experiment that shows that cell death occurs such as Brdu incorporation or stainings for apoptotic markers.

6. The authors should consider changing the title of the manuscript since it does not align with the findings shown.

7. Figure 3D, quantify the number of organoids

8. The authors explain the observed phenotypes due to the accumulation of DNA damage due to NAD+ depletion. Therefore, DNA damage measures are key to support their conclusions. In this regard, authors rely on yH2AX staining quantified by intensity/cell. The use of additional DNA markers for DNA damage, beyond yH2AX, should be also included to

further corroborate their results. In addition, measure of DNA damage in their NARPT KO aged mice is also requested.

9. The authors should show the activation of DNA repair pathways in gut epithelial cells upon DNA damage.

10. Microbiome or diet are the source of NA is their study? The relevance of the microbiome could be explored via antibiotics treatment to get insights into its role.

Reviewer #3

(Remarks to the Author)
The manuscript by Xiaoyue and colleagues "NAPRT-mediated deamidated NAD biosynthesis enhances tissue resiliency and suppresses tumorigenesis," aims to investigate the tumour suppressing roles of the deamidated arm of NAD biosynthesis. To achieve that, they constructed NAPRT gene knock-out models and assess their effects using both in vitro (CRC119 cell lines and colonic organoids) and in vivo (mouse) systems. Here they found that removal of NAPRT gene altered metabolism such as glycolysis, increased DNA damage, impaired PARP activity, heightened sensitivity to inflammatory stress, and susceptibility to tumorigenesis in mouse models. Furthermore, reduced NAPRT expression correlates with poor prognosis in stage 1, not stage 3 and 4, CRC patients.

The manuscript is well-written, and the analyses are thorough and comprehensive. In my view, the findings presented in this manuscript are valuable and provide some insights into the mechanisms underlying colorectal cancer (CRC) development. Additionally, these insight may also extend to understanding precancerous conditions, such as polyps, offering a foundation for further research in this area.

However, I have a few comments that I hope the authors will address to strengthen the manuscript further:
1. The authors focus their study on gut, but occasionally deviate and discuss NAPRT expression in other organs without clear purpose or sufficient discussion, which may confuse the reader. For example, it is not clear why the author show NAD metabolites in liver instead of colon in figure 3A?
2. For NAPRT/NAPRT expression in humans (Fig. 1A or Fig. 6I), it would be helpful to stratify the data based on previously published transcriptomic subtypes, such as CMS (https://doi.org/10.1038/nm.3967), IMF (https://doi.org/10.1038/s41588-022-01100-4), or MSI/MSS, to provide a more detailed understanding.
3. It would also be interesting to investigate whether there is any dysregulation of NAPRT in the polyps. The authors could consider utilizing previously published polyp datasets, such as those from Chen et al. (Cell, Volume 184, Issue 26, 6262 - 6280.e26) or Becker et al. (Nat Genet 54, 985–995 (2022)), to explore this further.
4. The mechanism behind the increase in NA following NAM feeding in the mouse model is unclear. The authors suggest that gut bacteria may convert dietary NAM to NA, but a similar increase in NA was also observed in other organs (Fig. 3C). Further explanation are needed.
5. It is unclear why NA was used as the substrate in the in vitro experiments, while NAM was used in the in vivo studies. The authors may consider using NAM as the substrate in the in vitro system as well, which could also help address my previous question (point 4).
6. Figure 4A, why NA level was decreased in KO mice after AOM treatment?
7. Figure 4C:
a. Why was the group highlighted in the black box below are not discussed? This group appears to include genes that are highly expressed in both WT and KO at 12 hours but downregulated in WT at 48 hours.
b. It would also be interesting to compare genes within the group across different time points (e.g., WT0 vs. WT12 vs. WT48) and then examine which pathways are altered in the KO
8. Figure 5H: The author write in line 330-333 that NAMPT, NAPRT and NADSYN1 are differentially altered in IBD. However, figure 5H clearly shown only NAMPT are differentially altered. This results actually contradict their conclusion that "NAPRT deficiency is associate with increased sensitivity to IBD". Better explanation and support are needed.
a. The author may also be interested in checking the NAPRT expression in colon instead of intestine (https://doi.org/10.1038/s41586-019-0992-y).
9. Figure 6I: The authors state in lines 387-388 that "TCGA datasets showed that the mRNA levels of NAPRT are reduced in a significant proportion of tumors," which is inaccurate. Figure 6I shows that, in COAD, only 40% of patients show a reduction in NAPRT, while another 40% actually show an increase. This discrepancy is even more pronounced in the LUAD and LUSC datasets, where a larger proportion of patients exhibit increased NAPRT expression. This contradicts the authors' claim that reduced deamidated NAD biosynthesis is associated with tumorigenesis in both mice and humans (lines 394-395). A better explanation and additional support for this discrepancy are needed.
10. The figure legends could be improved. Ex: it is unclear what is M+2, M+3, and M+4 in figure 3A and what is 0.5,1,1.5, and 2 in figure 5A means.

Version 1:

Reviewer comments:

Reviewer #1

(Remarks to the Author)
This manuscript is a revised version of a paper I previously reviewed. In this revision, several issues have been addressed

and improved. However, I believe there remain critical concerns, particularly regarding aspects of systemic nicotinic acid (NA) metabolism, that require further revision.

Major points

1. In my previous review, I pointed out that there is a Nat Commun paper (Nomura M, PMID: 38092728) demonstrating that NA is converted to NAR in a NAPRT-dependent manner. During this revision period, an additional study reported NA-to-NAR conversion in mice (Song WS, Cell Metab '25, PMID: 40315855). Thus, two independent groups have now shown that NA can be converted to NAR. Nevertheless, the present manuscript does not mention or consider this conversion pathway at all. I believe this omission constitutes a significant limitation.

2. The authors state that the NA concentration used in the in vitro experiments (100 uM) is comparable to the peak serum concentration following NAD booster administration, as reported in the previous study (Chellappa K, Cell Metab '22, PMID: 36476934). While this is factually correct, the study also reported that steady-state levels of NA (in the absence of NR administration) are very low. Omitting this point could lead readers to overestimate physiological NA concentrations.

3. To allow for a more accurate and reliable interpretation of the metabolic data presented in this study, I strongly recommend that the authors include, at a minimum, plasma or serum (not whole blood) concentrations of NAD-related metabolites in WT and Naprt-KO mice, both at both at baseline (prior to NA administration) and at multiple time points following NA administration. Blood sampling should preferably be performed from the systemic circulation, rather than the portal vein, to better reflect whole-body metabolism. In this context, blood collection from the right ventricle is considered the most appropriate.

Minor points.

4. In Figures 1 and 2, the in vitro experiments were conducted using only one colorectal cancer cell line, CRC119. The authors should be more cautious in generalizing the results to normal "gut epithelial cells" as a whole. In addition, more information about CRC119 should be provided in the Methods section; at present, even the species is not indicated.

5. In Figure 2D, the results of the add-back experiment are not clear. Such experiments should be performed using a stable expression system, rather than transient transfection.

Reviewer #2

(Remarks to the Author)

At the outset, I was indeed very positive about this manuscript, under the condition that my initial comments were thoroughly addressed. While the paper remains a very well-written and insightful contribution, providing important advances on the role of the Preiss–Handler pathway in NAD biosynthesis, particularly the function of NAPRT in DNA repair and tumorigenesis, it is concerning that some of my key points appear to have been overlooked.

Several other key issues outlined below remain unaddressed. I strongly recommend that these be carefully revised and clarified before the manuscript can be considered suitable for publication. If adequately addressed, this work could indeed represent a valuable contribution to the field.

Point 1

In particular, as highlighted in my Major Concern, the pioneering study by Tummala et al. (2014) on the role of NAD^+ in tumorigenesis, along with the more recent and controversial work by Lee et al. (2022), should be not only cited—which the authors have done—but also properly discussed and integrated into the conceptual framework of the manuscript. These citations currently lack critical engagement.

I respectfully disagree with the response provided to Comment 1. The authors argue that their study is mechanistically distinct from the work of Tummala et al. and Gomes et al., both of which demonstrated that NAD^+ depletion leads to DNA damage, likely through PARP inactivation or reduced dNTP synthesis. While the authors emphasize that their study centers on NAPRT and its role in DNA repair, they also conclude that NAPRT knockout cells, which have compromised NAD^+ levels, exhibit defective DNA repair and accumulate DNA damage upon MMS treatment.

In my view, the underlying message of these studies remains fundamentally aligned. Therefore, a proper and critical discussion of these prior findings is necessary. Without it, the current manuscript falls short in demonstrating sufficient novelty.

Mechanistic insights into how NAPRT deficiency leads to DNA damage remain insufficiently resolved. Although the authors now demonstrate reduced PARylation and MARylation in NAPRT KO cells, this evidence essentially confirms the role of PARP in their phenotype, thereby aligning their study more closely with previously published findings. Similarly, while SIRT1's role remains unclear in their system, and dNTP synthesis appears largely unaffected. As established in seminal studies such as Tummala et al. (2014) and Gomes et al. (2016), NAD^+ depletion can lead to DNA damage through several converging pathways, including PARP inactivation, Sirtuin inhibition, and reduced dNTP synthesis, all of which are potentially relevant in this context. These studies should be properly cited and integrated into the manuscript's conceptual framework.

Therefore, I respectfully maintain that the manuscript would benefit from a more rigorous and integrative discussion acknowledging how their findings intersect with existing knowledge and litteratire. The notion that NAPRT-derived NAD^+ is essential for DNA repair in gut epithelial cells is interesting; however, the message conveyed converges with previously published observations about NAD^+ depletion and genome instability. This convergence must be transparently acknowledged and discussed to better contextualize the contribution of this work and to address concerns around novelty.

It is unclear why the authors have chosen to omit citation and discussion of previous pioneering studies that have significantly shaped the understanding of NAD^+ metabolism and its role in DNA damage and tumorigenesis.

Point 2

The authors fairly replied to this point: Orally administered NR and NMN are largely converted by gut microbiota into nicotinic acid (NA), which then enters the Preiss-Handler pathway requiring NAPRT for NAD^+ synthesis. In NAPRT knockout (KO) models, this pathway is disrupted, preventing NAD^+ restoration from NR/NMN, as seen in both in vitro (e.g., CRC119 cells) and in vivo studies.
Despite this, NR and NMN remain widely used because, in organisms with functional NAPRT and normal gut microbiota, they effectively boost NAD^+ levels and provide physiological benefits. Thus, the efficacy of NR/NMN depends on the presence of NAPRT and highlights the critical role of the Preiss-Handler pathway in their metabolism.

Yet, dietary levels of tryptophan may be sufficient to compensate for NAPRT deficiency in many tissues. This has not been tested. A rescue experiment can still be important.

Point 3

This is justified by the authors stating, "We agree that conditional NAPRT KO in different cell types would be more informative. However, as the reviewer noted here, such studies are beyond the scope of the current revision."
This is inaccurate; the reviewer never claimed it was beyond the scope but explicitly requested this experiment. Nevertheless, the xenograft studies presented do not fully support the paper's conclusions, and conditional KO mouse models would be important to address this gap.

Other important points

I regret to note that γH2AX, the standard marker for DNA damage, has not been successfully demonstrated in this study, despite its widespread and well-established use in numerous publications. It is unclear why evidence of DNA damage is not provided or visible here.

Reviewer #3

(Remarks to the Author)
The authors have addressed all my concern

Version 2:

Reviewer comments:

Reviewer #1

(Remarks to the Author)
I appreciate that in this revision the authors have addressed the concerns I previously raised. However, there remains one important issue that should be resolved before publication. I apologize for not noticing it earlier, but I believe it is critical for the validity of the study.

This concerns the mutant mouse line referred to as "Naprt-KO" in the manuscript. Typically, genome-editing–based gene disruption involves a frameshift mutation, which leads to nonsense-mediated mRNA decay and thus a marked reduction in transcript levels. However, as the authors noted, the allele in this mouse carries an in-frame deletion of seven amino acid residues (Fig. S3A), which would not normally cause a severe decrease in mRNA levels as reported in Fig. S3B.

Although several functional studies in the paper suggest a loss of Naprt activity, it remains essential to confirm that Naprt protein is indeed absent in this mutant line. This could be demonstrated, for example, by Western blotting of tissue lysates. Such verification would clarify whether the allele truly represents a null mutation.

I am aware that the deletion may have disrupted an exon splicing enhancer (ESE), potentially leading to cryptic splicing;

however, this possibility further underscores the need to verify the actual loss of Naprt protein.

Reviewer #2

(Remarks to the Author)
Although I was initially very critical of certain experiments and aspects of the discussion, the authors have addressed all my concerns. I am satisfied with their responses and the revisions provided.

One minor point is that the authors should discuss the limitation of the study arising from the use of xenograft models, rather than the use of conditional NAPRT knockout in different cell types.

With this minor suggestion, I recommend that this paper be accepted for publication.

Open Access This Peer Review File is licensed under a Creative Commons Attribution 4.0 International License, which permits use, sharing, adaptation, distribution and reproduction in any medium or format, as long as you give appropriate credit to the original author(s) and the source, provide a link to the Creative Commons license, and indicate if changes were made.
In cases where reviewers are anonymous, credit should be given to 'Anonymous Referee' and the source.
The images or other third party material in this Peer Review File are included in the article's Creative Commons license, unless indicated otherwise in a credit line to the material. If material is not included in the article's Creative Commons license and your intended use is not permitted by statutory regulation or exceeds the permitted use, you will need to obtain permission directly from the copyright holder.
To view a copy of this license, visit https://creativecommons.org/licenses/by/4.0/

REVIEWER COMMENTS

Reviewer #1 (Remarks to the Author):

In this paper, Wu X et al. have revealed that Naprt deficiency promotes two major processes in colon tumorigenesis: tumor initiation (DNA damage) and promotion (inflammation). They propose that the increased DNA damage in the Naprt-KO mice is likely due to a reduced PARP activity associated with lower NAD levels in epithelial cells. Given these results, a phenotype that Naprt deficiency leads to an increased incidence of colon tumors is convincing. These are novel and interesting findings. However, there are two major concerns regarding this paper that will require many additional efforts, as outlined below;

The first concern relates to the inverse correlation between Naprt expression and prognosis in several cancers (Fig 6J-L). As noted above, data regarding increased tumor formation in Naprt-KO mice is convincing. However, this does not imply that tumors with Naprt deficiency exhibit higher malignancy or more therapeutic resistance. After diagnosis, the prognosis of cancer patients typically worsens with increasing malignancy and/or treatment resistance of tumor cells. Importantly, tumor cells with DNA repair defects often show higher sensitivity to chemotherapy and radiation therapy, which can extend patient survival. Thus, even if Naprt-low cancers show worse prognoses compared to Naprt-high cancers, it does not seem reasonable to attribute this to an impaired DNA repair ability associated with Naprt deficiency.

Thanks for this insightful comment. We agree with the reviewer that we don't have a clear mechanistic understanding why cancer patients with low NAPRT-low tumors have worse prognosis than patients with NAPRT-high tumors. To address the reviewer's concern, we deleted the sentence "This observation in CRC supports the notion that NAPRT-mediated deamidated NAD biosynthesis is important for DNA repair, thereby suppressing tumor initiation" from the last paragraph of the Results section. We also modified the Discussion section and toned down the link of NAPRT and DNA repair in human patients.

Another major issue is that this study overlooks recent important findings on systemic NA metabolism. In brief, NA absorbed from the intestine is actively converted to nicotinic acid riboside (NAR) NAPRT-dependently, then released into the circulation (Nomura, Nat Commun '23. PMID: 38092728). Their paper reports that (1) the blood concentration of NA is much lower than that of NAR, and (2) Naprt deficiency significantly decreases the blood NAR levels in mice. These findings are critical to interpret the data presented in this paper. For example, in the culture experiments of this study, the authors added NA at a concentration of 100 µM, which is likely supraphysiological by a factor of 1000 or more in most organs. Direct fueling form the dietary NA or NA converted from Nam by gut microbiota may result in higher NA concentrations in the colon epithelium compared to the circulation. However, there is little direct evidence that NAD synthesis using NA as a substrate is active in other organs. Instead, it is questionable whether the proposed mechanism in this paper applies beyond the intestinal epithelium. Given the NA to NAR conversion, many of author's claim in this paper remain speculative.

If we understood the argument here correctly, the reviewer believed that based on the referenced

study, NAR but NA is the major circulating deamidated NAD precursor after dietary supplementation of NA/NAM. Therefore, the reduced NAD synthesis we observed in organs other than the colon epithelium in NAPRT KO mice was due to reduced circulating NAR released from the colon, rather than deletion of NAPRT in these organs. Thereby, the reviewer concluded that "there is little direct evidence that NAD synthesis using NA as a substrate is active in other organs".

This argument would hold true if only the data from the referenced paper were considered. However, our findings from the current study, along with those from a previous study, contradict this interpretation. First, our finding that NA was massively accumulated in all tested NAPRT KO mouse tissues (Figure 3C, S3F, and S3G) provided direct evidence that NAD synthesis using NA as a substrate is active in all these tissues. Moreover, the observation that robust NAAD and NAD biosynthesis in WT mice occurred without significant accumulation of NA, even at the earliest time point examined (except for the colon) (Figure 3C, NA), indicating that NAPRT-mediated NA assimilation is swift and highly efficient. Second, in our prior study [1], we analyzed systemic NAM/NR/NA/NAR metabolism. As shown in Figure 6D and Figure S5G of that paper, following oral NAM gavage, NAMN was the most abundant deamidated NAD precursor in both portal blood (12 pmol/mg) and inferior vena cava blood (8 pmol/mg), compared to 1.5 pmol/mg NA and 0.3 pmol/mg NR in inferior vena cava blood (Figure R1). Thus, in our experimental system, NAR is not the major circulating deamidated precursor. Yet despite the presence of various circulating deamidated precursors, NA accumulated in KO tissues beyond the intestinal epithelium, strongly indicating that NA serves as a main source for deamidated NAD biosynthesis in these tissues.

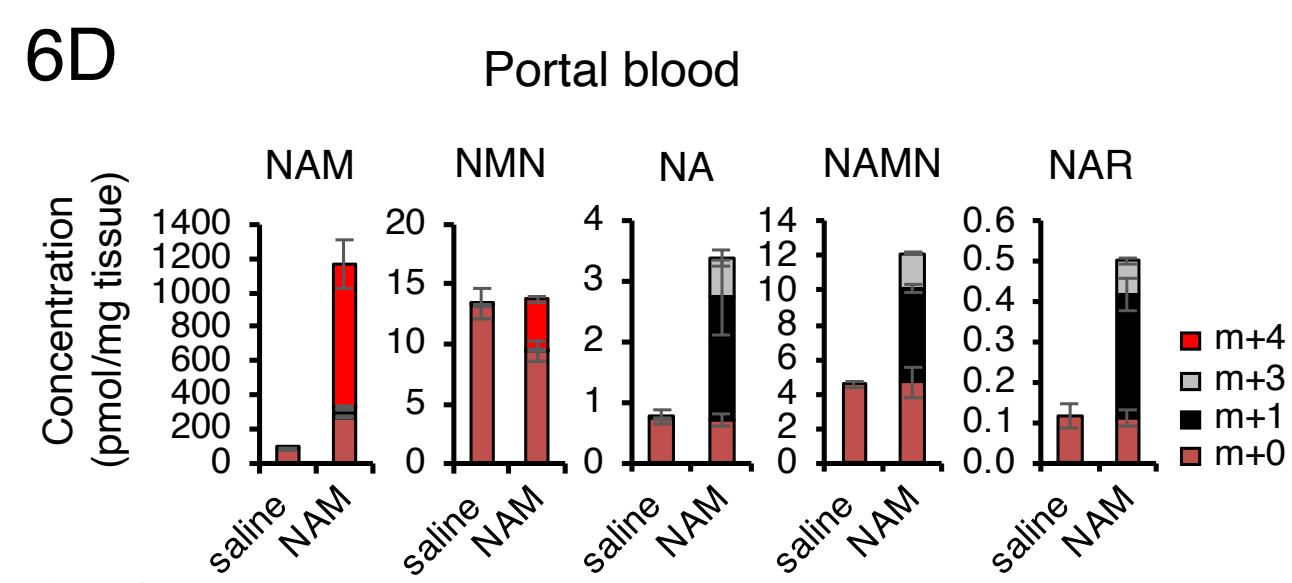

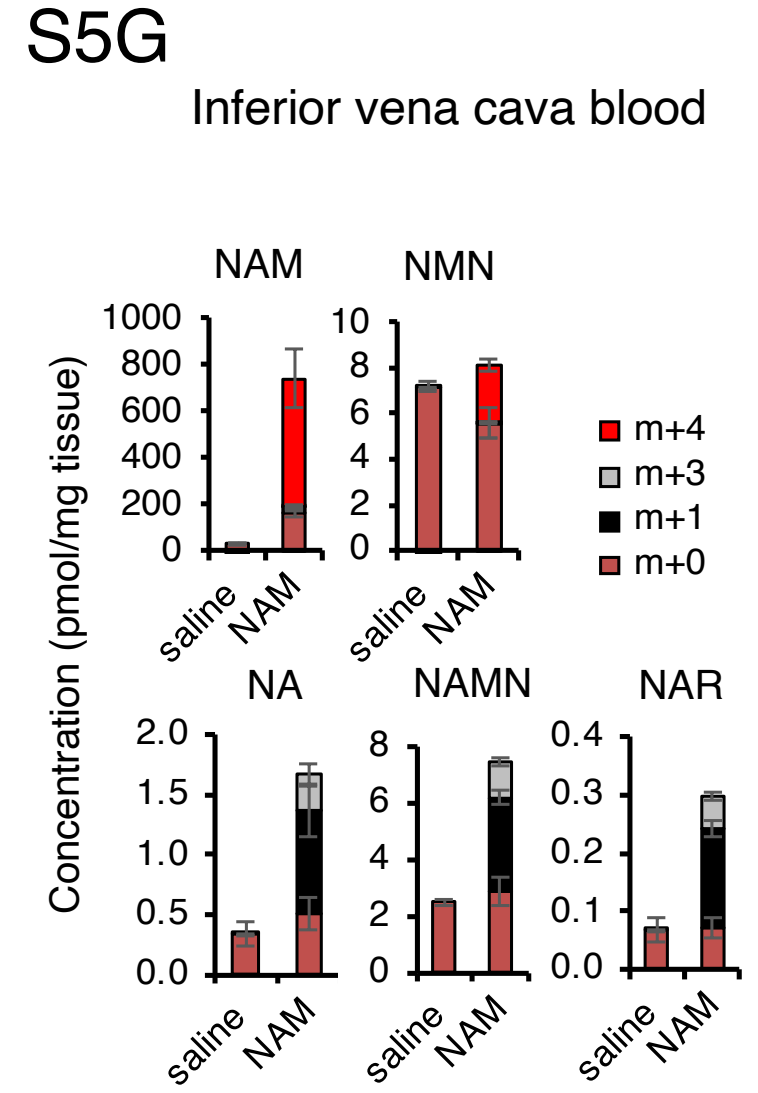

Figure R1. NAMN is the major deamidated NAM precursor in portal and systemic blood (data from Figure 6D and S5G in Shats et al., Cell Metabolism, 2020). Regular mice were gavaged with 80 mg/kg of 18O,15N,13C-NAM and the concentration of unlabeled (m+0) and labeled NAD pathway metabolites synthesized via the deamidated (m+3 and m+1) and amidated (m+4) pathways in blood collected from the portal vein and inferior vena cava were measured by LC-MS 3 hours after gavage as described in Methods (n=3 mice/group, values are expressed as mean ±SEM).

For the criticism on the concentration of NA we used in the cell culture system, please see our response below.

Besides the two major issues mentioned above, the following points require significant attention:

Figs 1-3: The authors added NA at a final concentration of 100 μM in many experiments in vitro.

A theoretical basis for this concentration setting must be provided. Additionally, It would be better to present results with lower concentrations of NA for comparison.

Indeed, the concentration of NA in the serum of mice on regular chow diet is around 1 µM NA [1]. However, it has been reported that this concentration goes up to 50-100 µM 8 hours after oral gavage with 500 mg/kg NR [2]. Please note that we and others have shown that NR is degraded in the gut to NAM, which is then deamidated by the gut microbiota to NA [1, 2]. Moreover, we have previously showed that in the colon, the NA concentration reaches 400 µM after gavage with 80 mg/kg NAM [1]. Thus, 100 µM of NA in our culture system is well within the physiological range after supplementation with NAD boosting supplements.

To further address the reviewer's concern, we performed a dose-response experiment, which revealed a biphasic response of NAD levels to increasing NA concentrations with 12.5-25 µM NA resulting in maximal increase in cellular NAD (Figure S2D).

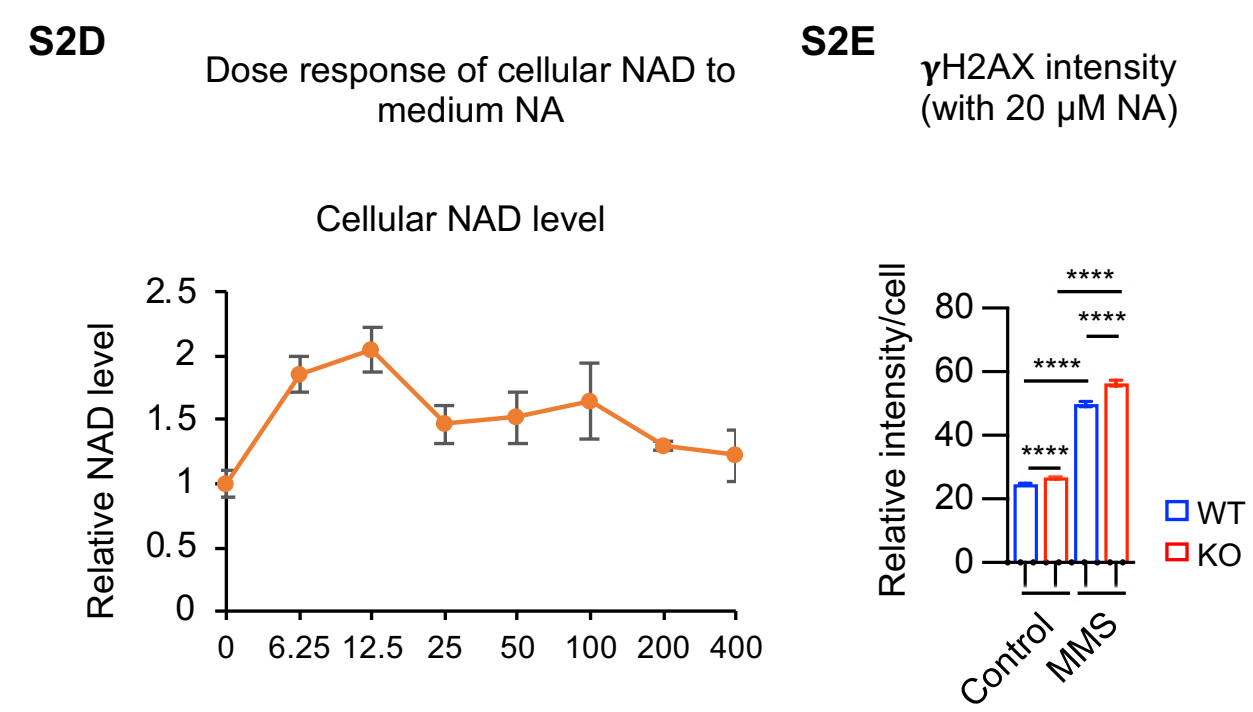

We then repeated the DNA damage experiment shown in Figure 2C, but now using 20 µM instead of 100 uM NA. We confirmed that NAPRT KO cells also have higher basal and MMS-induced DNA damage in the presence of this lower NA concentration (Figure S2E and new Figure 2B, see below).

Fig 2: The differences in PARylation and DNA damage between the parental and NAPRT-KO cells are relatively small. Therefore, add-back experiments should be conducted.

As suggested by the reviewer, we performed add-back experiments by transiently overexpressing NAPRT in two independent NAPRT KO clones. Cells were then treated with MMS for 1 hour and co-stained for NAPRT and γH2AX. As shown in Figure S2F and 2D, in both clones DNA damage was significantly lower in NAPRT-positive than in NAPRT-negative cells, confirming the genuine role of NAPRT in DNA repair.

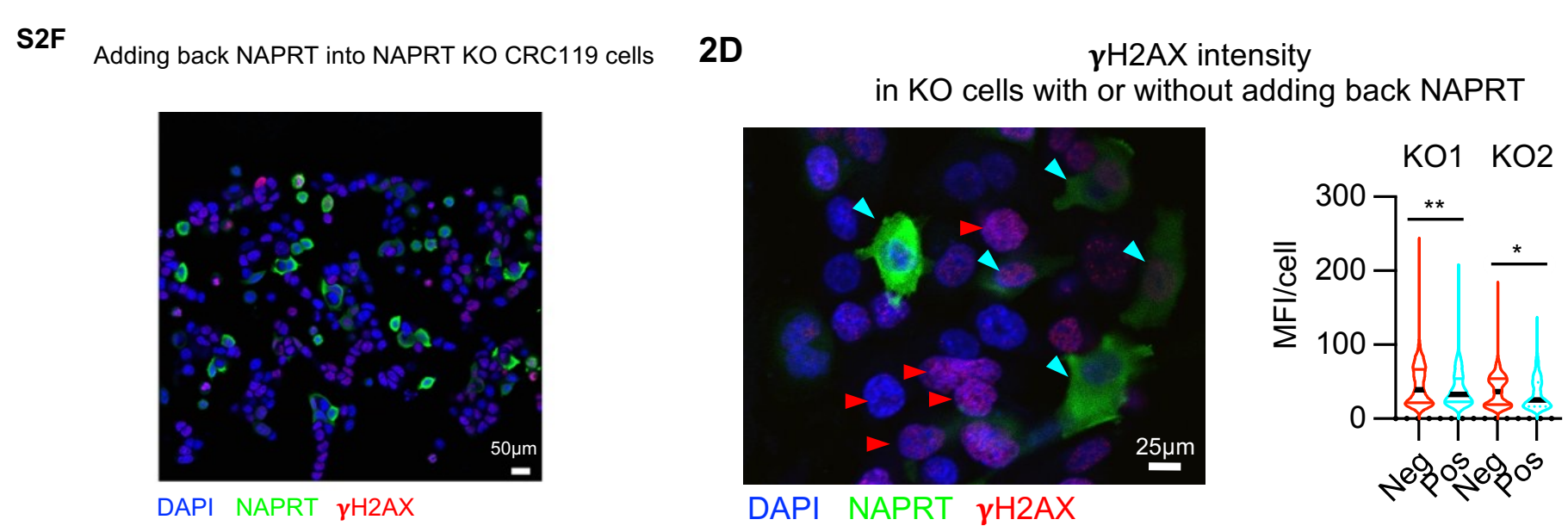

Alternatively, experiments with multiple KO clones should be performed. Additionally, gH2AX levels should be examined by WB for a clearer comparison.

The WT and NAPRT KO cells we used in the original Figure 2 are mixture of two independent clones each. We apologize that we forgot to mention this in the Methods section. Additionally, the PARylation results in Figure S2B are from two independent clones.

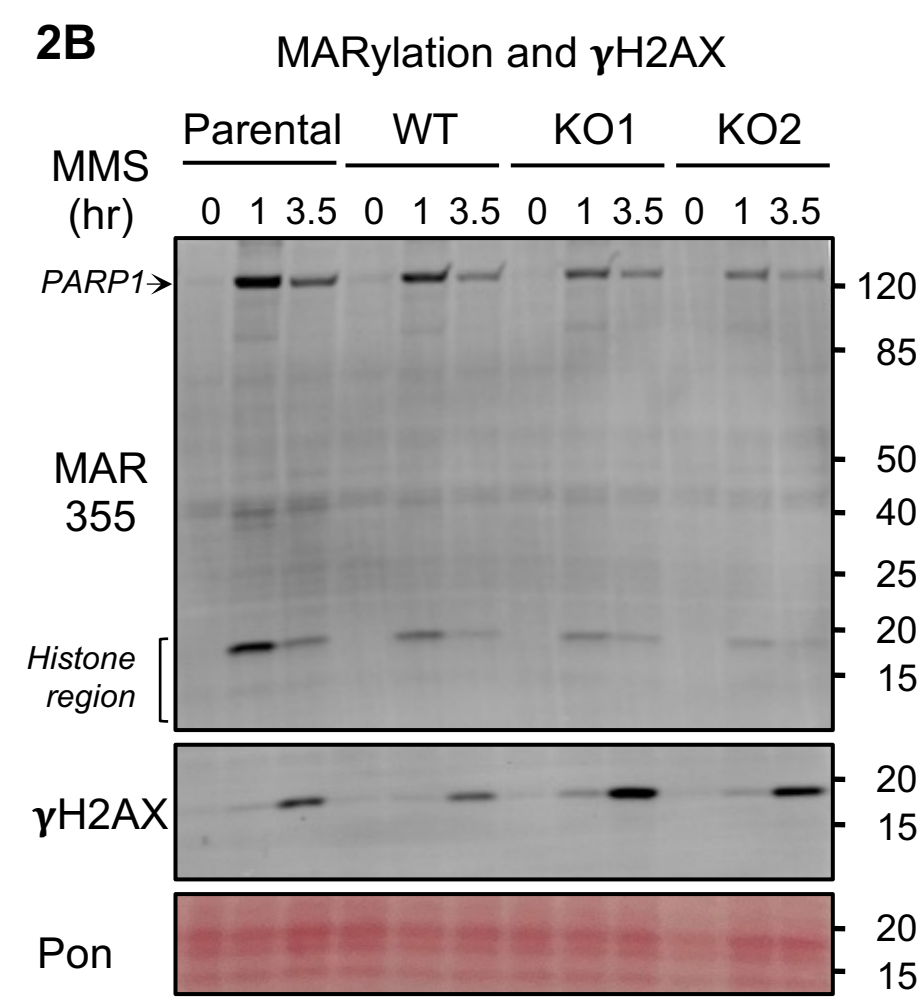

To further address the reviewer's concern, we optimized the timeframe of MMS treatment and analyzed Mono-ADP-ribosylation (MARylation) and γH2AX by western blotting in two independent KO clones separately. Cells were supplemented with 20 µM NA to address reviewer's request to repeat some experiments with a lower NA concentration. Similar to our results on PARylation (Figure S2B and S2C), auto MARylation of PARP1 (upper band) and MARylation of histones (lower band), which was recently shown to be important for efficient DNA repair [3], were less induced in both KO clones (Figure 2B), demonstrating reduced induction of PARP activity. The results further confirmed that NAPRT KO cells have increased γH2AX after MMS treatment in both KO clones, providing an additional piece of clear evidence that NAPRT KO cells have defective DNA repair thereby accumulating DNA damage after MMS treatment.

Please note that in the revision, the new MARylation and γH2AX results are shown in new Figure 2B, and the original PARylation data are moved into supplementary figures as Figure S2C.

Fig 3: The NAD levels in various organs of Naprt-KO mice under steady-state conditions (without Nam load) should be reported.

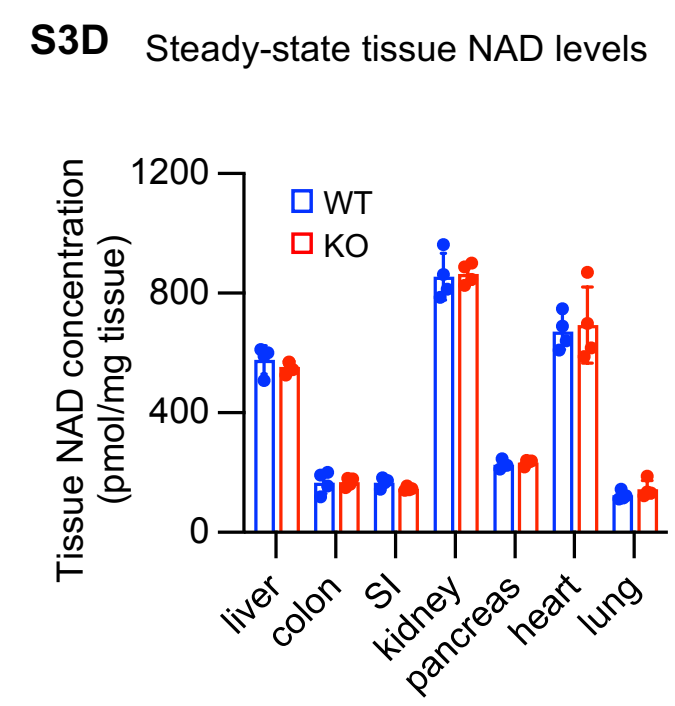

We added a new Figure S3D showing that without dietary supplementation of NAD precursors, the steady-state tissue NAD levels were normal in all tested tissues of NAPRT KO mice. As stated in our response to the next point, we believe this is due to compensation from amidated and/or *de novo* pathways.

Fig 4: The reason for administering additional NA through drinking water, given that NA is already provided in the dietary chow, should be clarified.

The reason why we supplemented experimental mice with NA in this experiment is because the chow diet (NIH-31) in our animal facility contains a very low level of NA (0.0083%) but a high level of Trp (0.20%), the precursor for *de novo* NAD biosynthesis. Given that hepatocytes, which have high expression of *Qprt* (Figure S1B), are the main producers of circulating NAM from NAD (>95% of total circulating NAM) and that circulating NAM serves as the primary NAD precursor for most tissues *in vivo* [4], it is likely that hepatic *de novo* NAD biosynthesis from Trp compensated for the defective deamidated NAD synthesis in NAPRT KO mice fed the chow diet. Additionally, although the expression of *Qprt* in mouse intestinal epithelial cells is very low

under normal conditions (Figure 1A), we were concerned that it might be activated by increased NAD consumption upon AOM-induced DNA damage, potentially masking the deficiency caused by NAPRT deletion. We therefore decided to supplement NA to increase the dependence of experimental mice on the deamidated pathway.

Panels C-E are underwhelming, and most of them could be moved to supplementary items.

We agree with the reviewer, therefore moved Figure 4E into Figure S5D.

Fig 6G: The experiment appears to be very important, but the method section lacks essential details. Were comparisons made using littermates? Were events (e.g., mortality) monitored up to the time of inspection at 24 months? These are critical details for interpreting the data.

The WT and NAPRT KO mice were originally littermates. After being backcrossed into the C57BL/6J background for four generations, they were bred separately within each genotype to produce sufficient experimental animals within the available space for this study.

For two cohorts of mice analyzed in this specific experiment, their health status and mortality were regularly monitored by the institute veterinary medicine staff during the two-year period of the study. But unfortunately, due to the renovation of the animal facility during this period, they were transferred multiple times between rooms, and we were not able to locate their health status and mortality records. The only data we have for these two cohorts is that at the time of inspection at 24 months, 6 out of 17 WT and 12 out 17 KO female mice were removed/lost, and 11 out 23 WT and 8 out of 27 KO male mice were removed/lost due to health issues/mortality. This result suggests that female NAPRT KO mice may have increased mortality. Indeed, analyzing another two ongoing independent aging cohorts confirmed that female but not male NAPRT KO mice had significantly reduced symptom-free survival compared with age-matched WT females (new Figure 7G). Among those females removed/lost from the aging cohort, 5 out 16 KO were due to visible large abdominal mass or rectal prolapse, whereas none of 6 removed/lost WT displayed these tumor-related symptoms.

7G

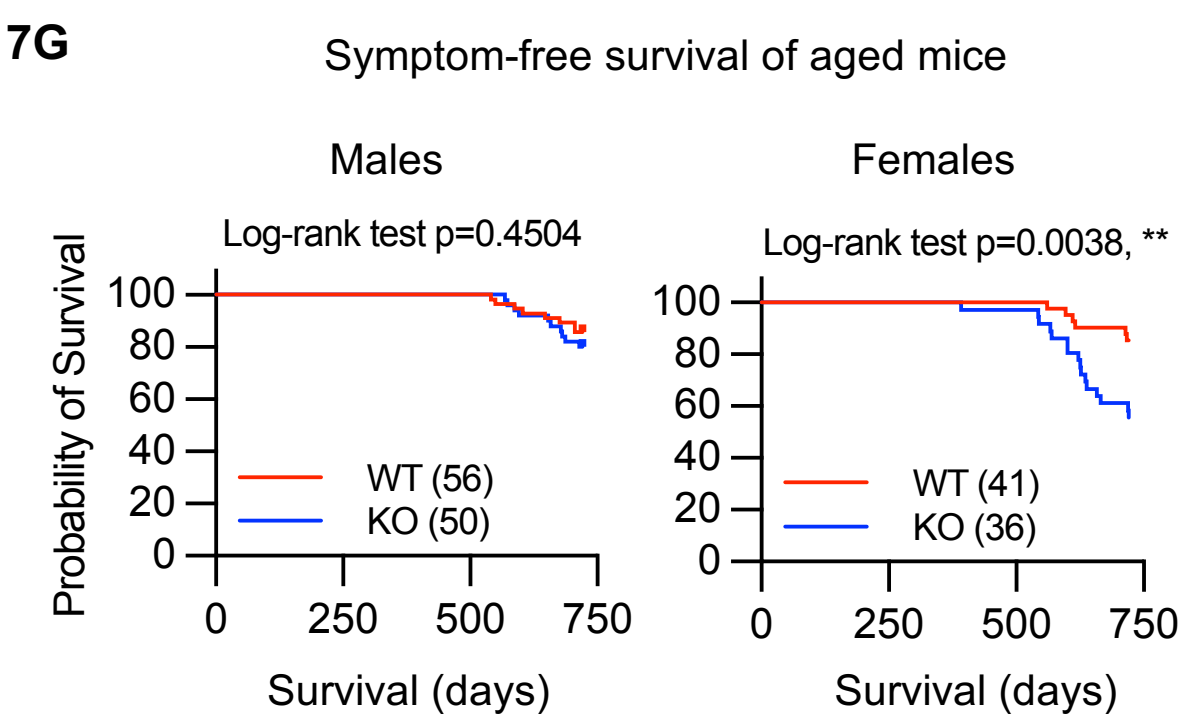

Moreover, the results should be presented separately for male and female mice.

Thanks for the suggestion. We re-graphed the original tumor incidence data and presented male and female mice separately and showed them in new Figure 7H.

The following statements appear overly speculative or exaggerated:

Line 160-1: “suggesting that NAD pool maintained by … … DNA repair.”

This statement was deleted in the revision.

Line 185: “in gut epithelial cells”

Deleted in the revision.

Line 186-8: “despite accumulation of more severe DNA … … the surviving cells.”

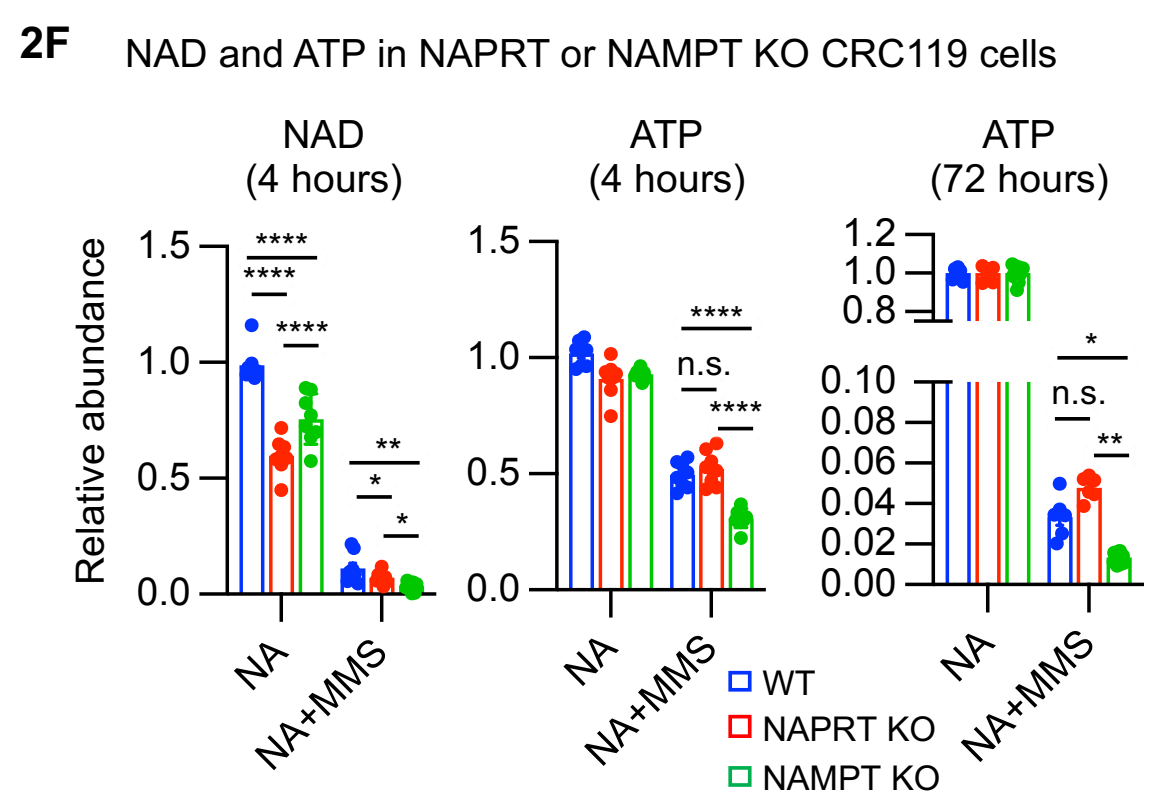

We hope that our new data will convince the reviewer that DNA damage is indeed more severe in NAPRT KO cells. We also now provide additional evidence that a “comparable fraction of NAPRT-deficient gut epithelial cells survives the stress” using Cell-titer GLO assay after 72 hours of continuous MMS treatment (Figure 2F, ATP (72 hours)). We believe that we were careful enough at this early point in the paper to qualify our hypothesis that increased DNA damage coupled with equal cell survival may be “**potentially** leading to increased mutagenesis and tumorigenesis within the surviving cells.” We use this hypothesis as a setup for the following cancer-related experiments and analyses. We are glad that the Reviewer found our results on increased tumorigenesis in NAPRT KO mice in 3 different models (AOM-DSS, APC, spontaneous aging) convincing. Since we do not provide direct evidence on increased mutagenesis, we deleted “mutagenesis” from this sentence.

Line 213: “NAPRT-mediated deamidated NAD … … all tested tissues.”

Please see our response to the second major concern. This is an accurate statement.

Line 218-20: “deamidated biosynthesis is a major route of … … and lung.”

Please see our response to the second major concern. This is an accurate statement.

Line 296: “NAPRT deficiency is associated with an increased sensitivity to IBD.”

We changed the title of this section to “NAPRT deficiency is associated with an increased sensitivity to DSS-induced colitis.”

Line 377: “NAPRT is believed to be oncogenic.”

This statement was changed into “*NAPRT* is found to be frequently amplified and/or overexpressed in various human cancer types and its knockdown slowed growth of xenograft tumors, suggesting tumor-promoting role of this gene [5, 6].”

Line 394-5: “These results indicate that … … in both mice and humans.”

This statement was deleted in the revision.

The reasoning behind the statements in lines 381-8 and lines 401-3 is difficult to follow.

We modified the statement in lines 381-8 into “In support of this hypothesis, AOM/DSS-induced tumorigenesis in WT mice was coupled with reduced expression of key deamidated NAD biosynthesis genes (Figure 8A). Compared to normal colon tissues from age- and gender-matched untreated mice, tumor adjacent colon tissues from AOM/DSS treated WT mice had significantly reduced expression of both *Naprt* and *Nadsyn* but not *Nampt* (Figure 8A, Adjacent vs Normal). The mRNA levels of these two key deamidated NAD biosynthesis enzymes were further reduced in AOM/DSS-induced tumors (Figure 8A, Tumor vs Adjacent).”

The statement in lines 401-3 was deleted.

Reviewer #2 (Remarks to the Author):

The study by Wu et al. explores how the Preiss-Handler pathway in NAD biosynthesis, mediated by NAPRT, impacts NAD metabolism, DNA repair, and tumorigenesis in the gut. Key findings highlight the role of NAPRT in sustaining NAD levels across tissues such as the liver, colon, and pancreas, and the consequences of NAPRT deficiency on NAD synthesis and tissue vulnerability to NAD-depleting stress.

In NAPRT KO mice, NAD synthesis is significantly compromised under DNA damage conditions, like those induced by AOM treatment, leading to inadequate metabolic and DNA repair responses, heightened inflammation, and increased gut cell damage. This dysfunction predisposes NAPRT KO mice to more severe colitis and tumorigenesis, resulting in a higher tumor burden, advanced adenomas, and upregulated inflammatory genes, underscoring the critical function of NAPRT-mediated NAD synthesis in safeguarding gut tissue from inflammation and cancer progression.

This is a very well written paper, that provides important insights into the role of the Preiss-Handler pathway in NAD biosynthesis, particularly in the context of NAPRT and its impact on DNA repair and tumorigenesis. However, it lacks novelty in certain aspects, as highlighted in major concerns, particularly point 5. The pioneering study on the role of NAD+ in tumorigenesis by Tummala et al. (2014) as well as the controversial work by Lee et al. (2022) should be cited, discussed, and integrated into the context of the manuscript, supported by experimental evidence. Additionally, several key issues outlined below need to be addressed before this manuscript is considered suitable for publication. Once these issues are addressed, this manuscript could make a valuable contribution to the field.

Major concerns

1. The connection between NAD+ depletion and DNA damage is well-established, as shown in pivotal studies like Tummala et al. (2014) and Gomes et al. (2016), whose papers have not been cited but should. However, in their study, authors should mechanistically provide more insights into how NAPRT KO leads to DNA damage. This could result from decreased PARP activity, inhibition of Sirtuins, reduced dNTP synthesis, or perturbation in alkylation routes, all pathways where NAD+ plays a crucial role. To clarify this, the authors should measure the activities of PARP, Sirtuins, and dNTP synthesis in the NAPRT KO context. These assays would provide insight into the specific mechanisms by which NAD+ depletion leads to genotoxic stress and tumorigenesis in their model.

Indeed, a wealth of literature has already established the connection between DNA damage and NAD depletion [7-10] and this connection was reviewed in [11, 12]. However, our study is not about this previously established link. Instead, our focus is on the sources of NAD for DNA repair in gut epithelial cells. Our observations showed that NAPRT-mediated deamidated NAD biosynthesis, which was previously considered a minor alternative pathway, is a major source of NAD for DNA repair in the gut epithelial cells. We did not cite Tummala et al. in the original manuscript because our study is focused on the deamidated NAD biosynthesis in the gut while Tummalal et al. [13] is about de novo NAD biosynthesis in the liver. We cited this study, as well as Gomes et al. [14], together with above literature in the revision.

In terms of the molecular mechanisms on how defective deamidated NAD biosynthesis leads to increased DNA damage, we have already demonstrated that the activity of PARP is decreased in NAPRT KO cells (previously Figure 2B and S2B, now Figure S2B and S2C) using protein PARylation as a functional outcome of in vivo PARP activity. To further address the reviewer's concern, we optimized the timeframe of MMS treatment and analyzed Mono-ADP-ribosylation (MARylation) and γH2AX by western blotting in two independent KO clones. Similar to our results on PARylation (Figure S2B and S2C), auto MARylation of PARP1 (upper band) and of histones (lower band), which was recently shown to be important for efficient DNA repair [3], were reduced in both KO clones (new Figure 2B), demonstrating a reduced PARP activity. The results further confirmed that NAPRT KO cells have increased γH2AX after MMS treatment in both KO clones, providing an additional piece of evidence that NAPRT KO cells have defective DNA repair thereby accumulating DNA damage after MMS treatment.

2B

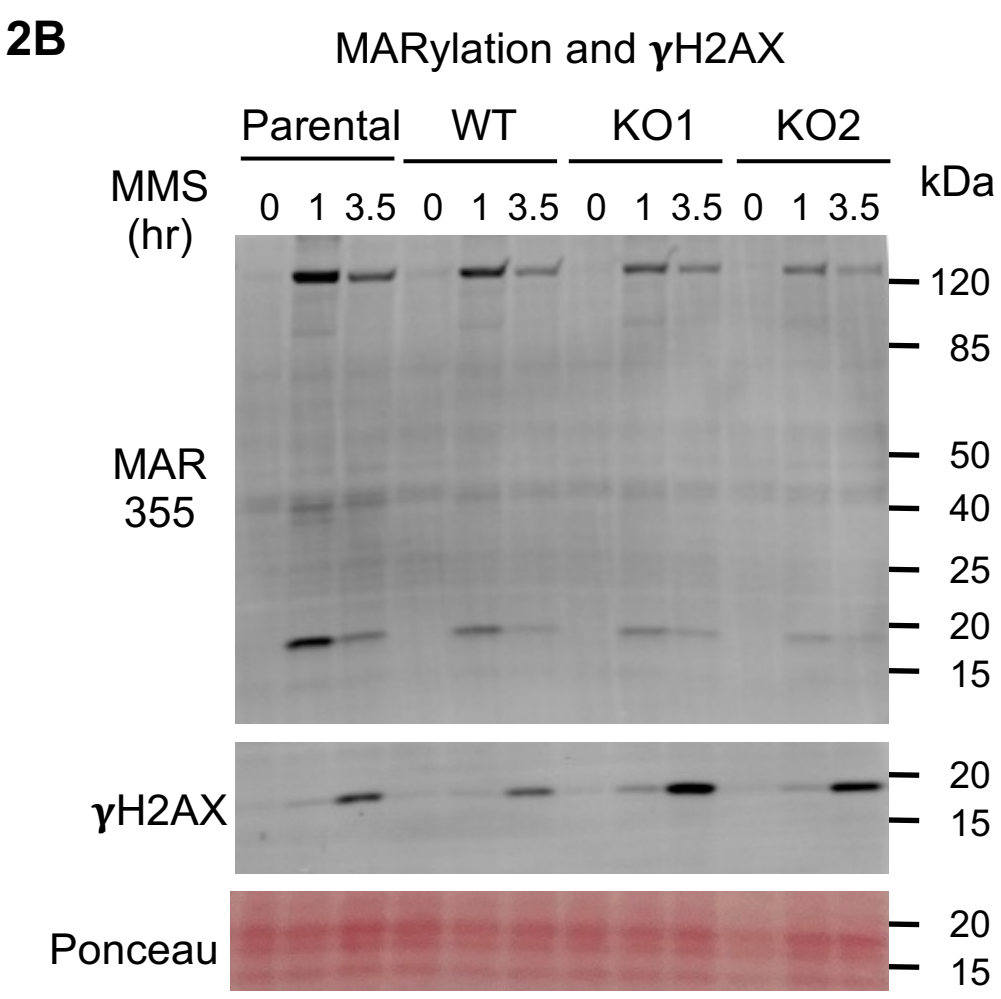

As requested by the reviewer, we tried to assess the activity of SIRT1 in WT and NAPRT KO CRC119 cells. SIRT1 is the most NAD-sensitive member of the sirtuin family [15] and plays a key role in DNA repair and genome stability [16]. Initially, we attempted to evaluate the acetylation status of a known SIRT1 deacetylation target p53, which is involved in the DNA damage

response, but were unable to detect any signal on Western blot possibly due to mutant or null p53 in CRC119 cells. As an alternative approach, we examined whether NAPRT deficiency alters cellular sensitivity to SIRT1 inhibition using EX527, a SIRT1-specific inhibitor. Unexpectedly, treatment with EX527 *reduced* γH2AX levels in WT CRC119 cells and NAPRT KO CRC119 cells were slightly less responsive to this inhibition (Figure R2). This observation suggests that SIRT1 may not play a direct role in DNA repair following the MMS-induced DNA damage in this cell line.

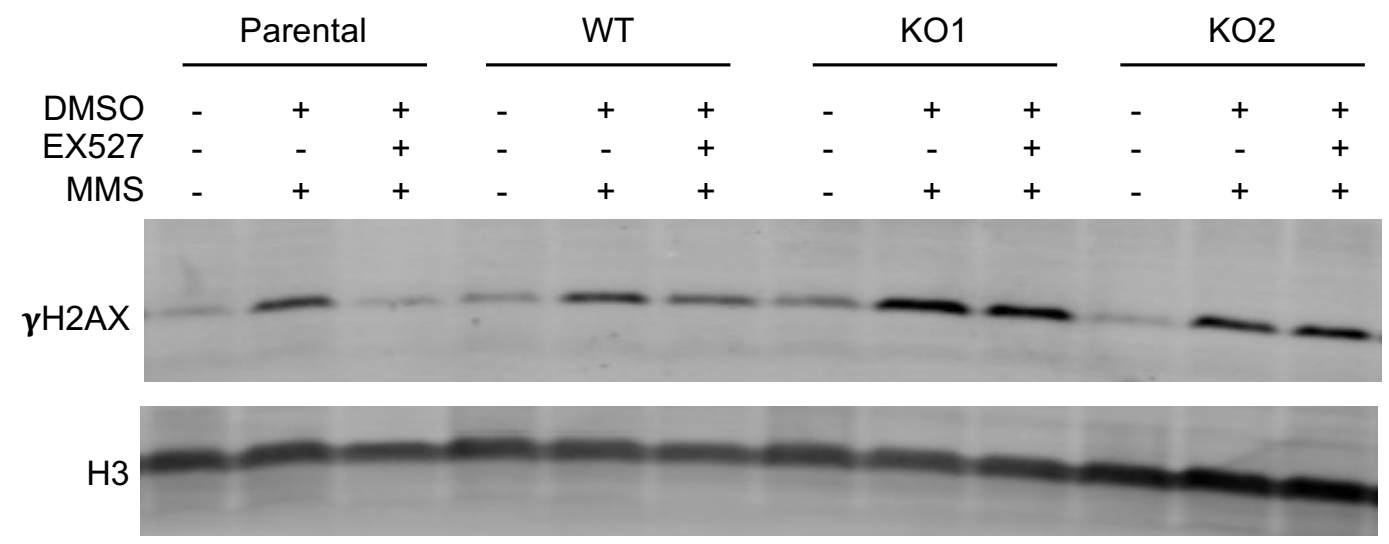

Figure R2. The impact of SIRT1 inhibition on DNA damage in WT and NAPRT KO CRC119 cells. WT and NAPRT KO CRC119 cells were cultured in full medium containing 8 μM NAM and 20 μM NA for 20 hours. They were then treated with or without 1 mM MMS together with 0.1% DMSO vehicle control or 10 μM EX527 for additional 4 hours.

Regarding dNTP synthesis, we have performed a metabolomic analysis of colonic tissues from WT and NAPRT KO mice at 0, 12, and 48 hours after i.p. injection of AOM (Figure 4B and S4C, and Table S1). We analyzed 15 dNTP-related metabolites that were identified or annotated in this dataset (Figure S4D). The mice in this experiment were given with 3 g/L NA in drinking water for 24 hours before AOM injection. As a result, the colonic tissues from the NAPRT KO mice displayed about 50% reduction of NAD levels compared to those from WT mice at time 0 (Figure 4A and S4C). However, despite this marked reduction of NAD, only 2'-deoxyuridine (out of 15 metabolites) was significantly altered in the KO colons (Figure S4D, KO 0 vs WT 0), suggesting that defective deamidated NAD biosynthesis in NAPRT KO colons does not result in major changes of dNTP biosynthesis.

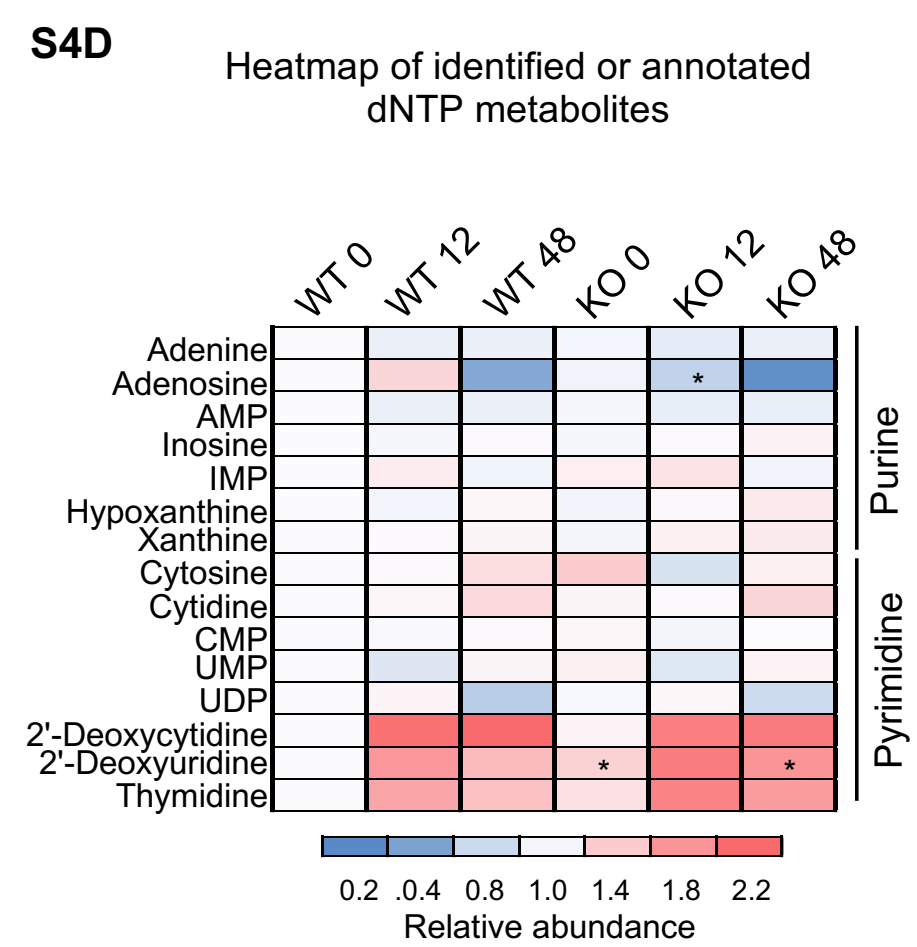

Interestingly, AOM treatment significantly induced three deoxyribonucleosides, including 2'-deoxycytidine, 2'-deoxyuridine, and thymidine, in both WT and NAPRT KO colons (Figure 4C). Moreover, their induction peaked at 12 hours after AOM injection when DNA damage was at the maximum (Figure 4C), suggesting that DNA damaged-induced DNA turnover and repair may be the sources of these nucleosides [17]. Particularly, KO colons exhibited significantly elevated levels of 2'-deoxyuridine compared to WT at all tested time points and did not show significant recovery at 48 hours (Figure 4C, 2'-deoxyuridine), suggestive of increased DNA damage in these mice. This finding aligns with our observations in Figure 4D.

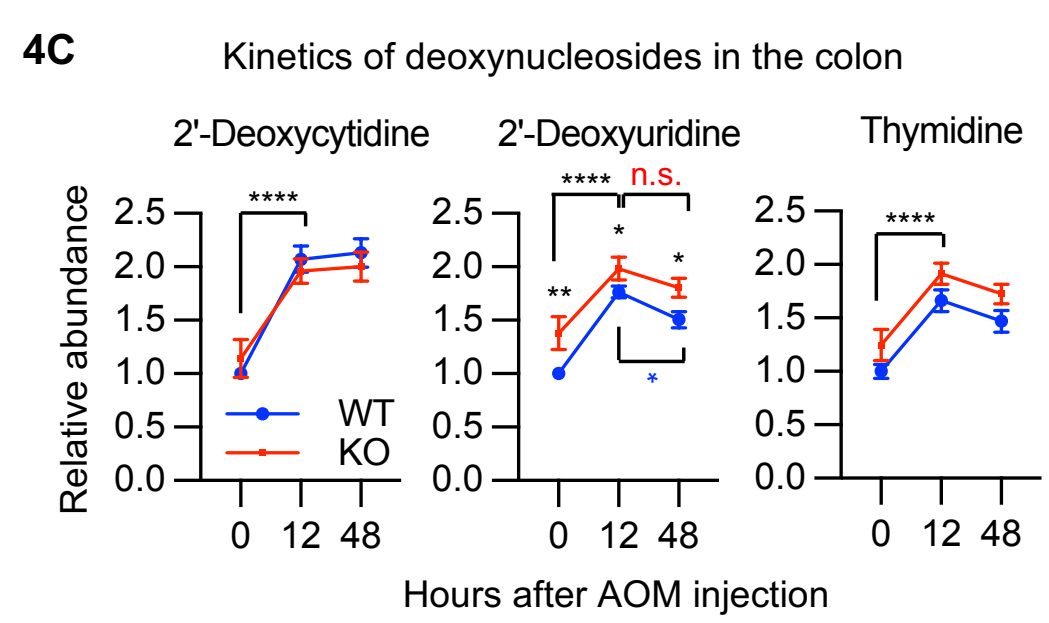

Furthermore, performing rescue experiments, such as supplementing precursors to restore NAD+ levels or enhancing activity in the affected pathways, would significantly strengthen the study's conclusions regarding the role of NAD+ metabolism in DNA damage and cancer progression. Rescue experiments with dNTPs, or using PARP inhibitors are also requested.

A rescue experiment using an alternative NAD precursor independent of the NAPRT-mediated deamidated NAD biosynthesis is a great idea, which had also been one of our original plans. However, we were not able to obtain a suitable NAD precursor for this type of rescue experiment. First, we and others have already reported that in vivo, orally administered NR is largely degraded in the gut to NAM, which is then deamidated by the gut microbiota to NA, boosting tissue NAD biosynthesis primarily via NAPRT-mediated deamidated NAD biosynthesis [1, 2]. Orally administered NMN has also recently been shown to be deamidated by gut microbial and host deamidases, incorporating into NAD through the deamidated pathway [18]. In fact, even after IV delivery, the direct contribution of intact NR or NMN to the liver NAD is minimal in mice [4]. Therefore, NR and NMN are unlikely to alleviate NAD deficiency in NAPRT KO mice. Secondly, when tested in vitro, NR was not able to effectively boost NAD levels at physiologically relevant concentrations in our CRC119 cells (Figure R3).

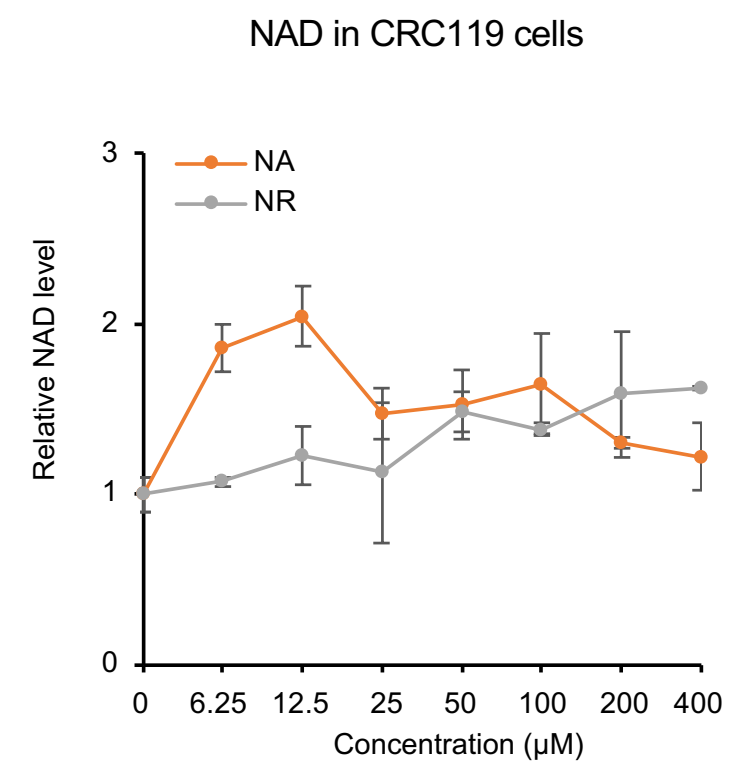

Figure R3. NA, but not NR, increases cellular NAD levels in CRC119 cells. CRC119 cells were cultured with indicated concentration of NAD precursors for 22 hours in regular RPMI medium. Cellular total NAD levels were measured by the NAD-GLO assay kit.

In terms of rescue experiments with dNTPs, as outlined in our response above, we did not observe significant alterations of their levels in NAPRT KO colons (Figure S4D).

The rationale of rescue experiments using PARP inhibitors in the context of DNA damage and cancer progression is not clear to us, as we have already shown that DNA-damage induced activation of PARP is *reduced* in NAPRT KO cells (Figure 2B, S2B, and S2C). Further treatment with PARP inhibitors will only lead to increased DNA damage. Nevertheless, we performed the experiments using PARP inhibitor Olaparib as the reviewer requested, and analyzed cellular NAD levels and cell survival (Figure R4). Inhibition of PARP activity rescued MMS-induced NAD depletion in both WT and NAPRT KO CRC119 cells

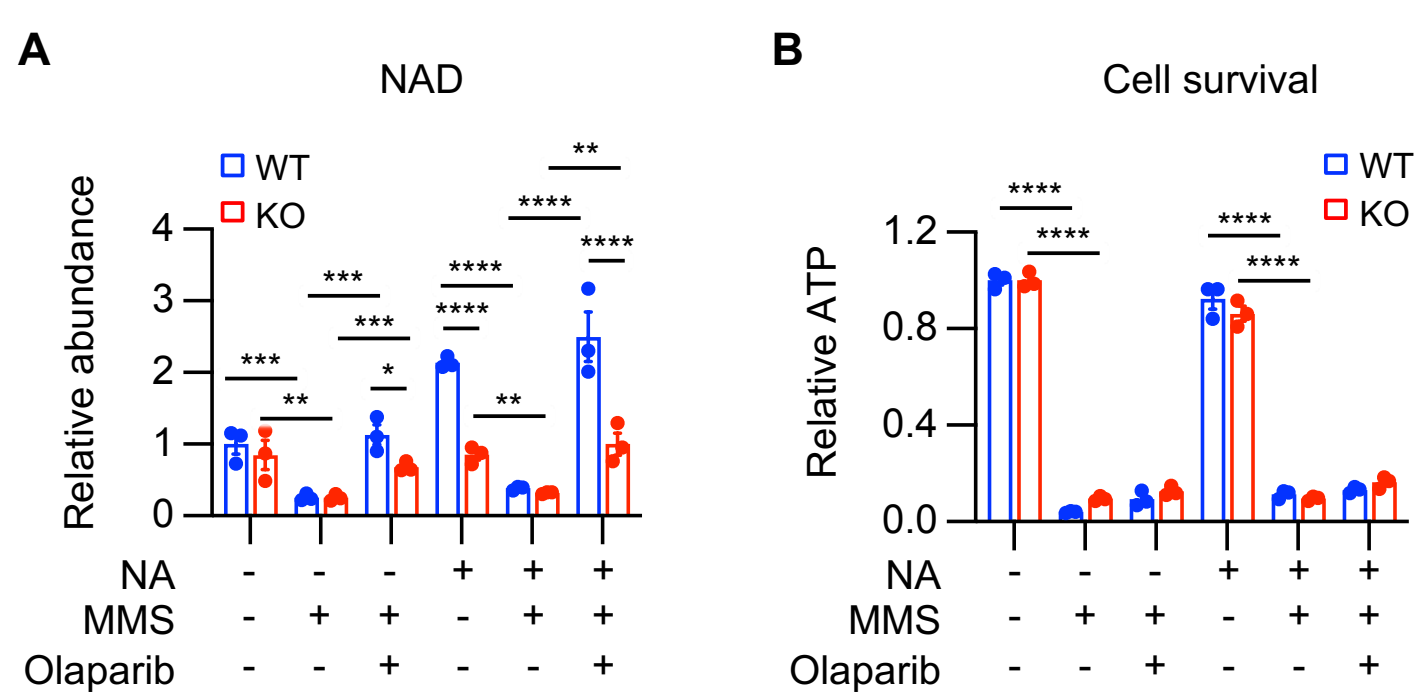

Figure R4. PARP inhibitor Olaparib rescues DNA damage-induced NAD depletion but not cell survival in CRC119 cells. WT and NAPRT KO CRC119 cells were cultured in regular medium containing 8 µM NAM or full medium containing 8 µM NAM and 20 µM NA. (A) WT and NAPRT KO CRC119 cells were treated with 1 mM MMS with or without 10 µM olaparib for 5 hours and their cellular NAD levels were measured by the NAD-GLO assay kit (n=3 replicates/group, values are normalized to the first WT sample; 2-way ANOVA, *q<0.05, **q<0.01, ***q<0.001, ****q<0.0001). (B) WT and NAPRT KO CRC119 cells were treated with 1 mM MMS with or without 10 µM olaparib for 4 hours. They were then washed and cultured in fresh regular medium or full medium for additional 72 hours. ATP levels in the surviving cells were measured (n=3 replicates/group, values are normalized to the first WT sample; 2-way ANOVA, ****q<0.0001).

(Figure R4A), demonstrating that PARP1 is the main consumer of NAD during DNA repair in this system. However, this recuse of NAD did not lead to increased cell survival (Figure R4B), suggesting that MMS-induced cell death is not caused by NAD depletion.

2. A key concern is that while NAMPT depletion results in cell death, NAPRT KO does not induce such effects, in particular because the medium is supplemented with NAM. Likewise, NAPRT KO mice develop normally without obvious phenotypes, whereas NAMPT KO mice are embryonically lethal. The authors conclude that the deamidated NAD biosynthesis pathway is non-essential for survival, development, or normal cellular function. However, the underlying reason for this disparity remains unclear. Are NAD+ levels significantly more reduced to be completely abolished in NAMPT KO compared to NAPRT KO, thereby leading to extensive DNA damage, cell death, and catastrophic outcomes in NAMPT KO but not in NAPRT KO? Could it be that NAD+ depletion is less severe in NAPRT KO, allowing partial compensation through NAD+ synthesis from tryptophan catabolism? Notably QPRT is expressed in human colons and therefore this pathway might still be active despite the enzymes are not highly expressed in mice. Resolving these questions is crucial, and the study would benefit from comparative NAD+ quantification between NAMPT and NAPRT KO models, along with an analysis of compensatory metabolic pathways.

Good questions. NAD has a half-life from 15 minutes to 15 hours in different cells and tissues due to homeostatic activities of various NAD-consuming enzymes (e.g. PARP, SIRT, CD38, SARM, etc.) [4, 19]. Therefore, rapid and efficient biosynthesis is essential to maintain NAD homeostasis and cell survival, especially in cells and tissues with high NAD turnover. Theoretically, with a sufficient supply of the correspondent substrates (NAM, NA, or tryptophan) and adequate expression levels of the corresponding biosynthetic enzymes (NAMPT; NAPRT, NADSYN; IDO, TDO, QPRT, NADSYN), the activity of a single pathway (amidated, deamidated, or *de novo*, respectively) could be sufficient to maintain levels of NAD necessary for cell viability. However, based on the literature reports and our own observations, we believe there are at least two reasons why global NAMPT KO in mice is lethal, whereas mice with NAPRT KO are apparently normal in unstressed conditions at young age. First, NAMPT is expressed in all cells to recycle NAM released by NAD-consuming enzymes back into NAD. Since its activity is also subject to feedback inhibition by NAD [20], this enzyme is essential in maintenance of a homeostatic level of NAD in all cells. In contrast, the expression pattern of NAPRT (as well as various enzymes of the *de novo* pathway) is more tissue specific and relatively enriched in nutrition-rich organs. Thus, in NAMPT KO, the deamidated and *de novo* pathways cannot rescue NAD levels at least in some tissues. Secondly, even in tissues that have high deamidated and/or *de novo* biosynthesis activities, the dietary levels of NA and/or tryptophan (0.0083% and 0.20%, respectively in the NIH-31 chow diet) may not be sufficient to compensate for NAMPT deficiency. For instance, in hepatocytes where all three major NAD biosynthesis pathways are highly active (Figure S1B), specific deletion of NAMPT still leads to a 40-50% reduction of total tissue NAD [21, 22]. This suggests that the combined hepatic deamidated and *de novo* NAD biosynthesis are not able to match the rate of hepatic NAD consumption in the absence of NAMPT. In NAPRT KO mice, on the other hand, NAM remains abundant in all tissues due to its continuous supply from NAD degradation within the tissues, with NAMPT recycling this NAM back into NAD. Moreover, the relatively high level of dietary tryptophan (0.20% in NIH-31 chow diet) could compensate for the defective deamidated NAD

synthesis in their liver and some other tissues (e.g. kidney) via *de novo* NAD biosynthesis. The elevated hepatic *de novo* NAD biosynthesis could further mask the deficiency caused by NAPRT deletion in other tissues, as hepatocytes are the main producers of circulating NAM (>95% of total circulating NAM), which serves as the primary NAD precursor for most tissues *in vivo* [4]. Consequently, NAPRT KO mice display no defects in basal NAD levels in all tested tissues (Figure S3D).

Per the request of the reviewer, we directly compared the relative contribution of the amidated and the deamidated pathways to cellular NAD in gut epithelial cells in vitro by generating NAMPT KO CRC119 cells using the crispr/cas9 gene editing technology (Figure S2G). We cultured WT, NAPRT KO, and NAMPT KO cells in regular medium (containing 8 μM NAM) supplemented with or without 20 μM NA, then measured their cellular NAD and ATP levels (Figure R5).

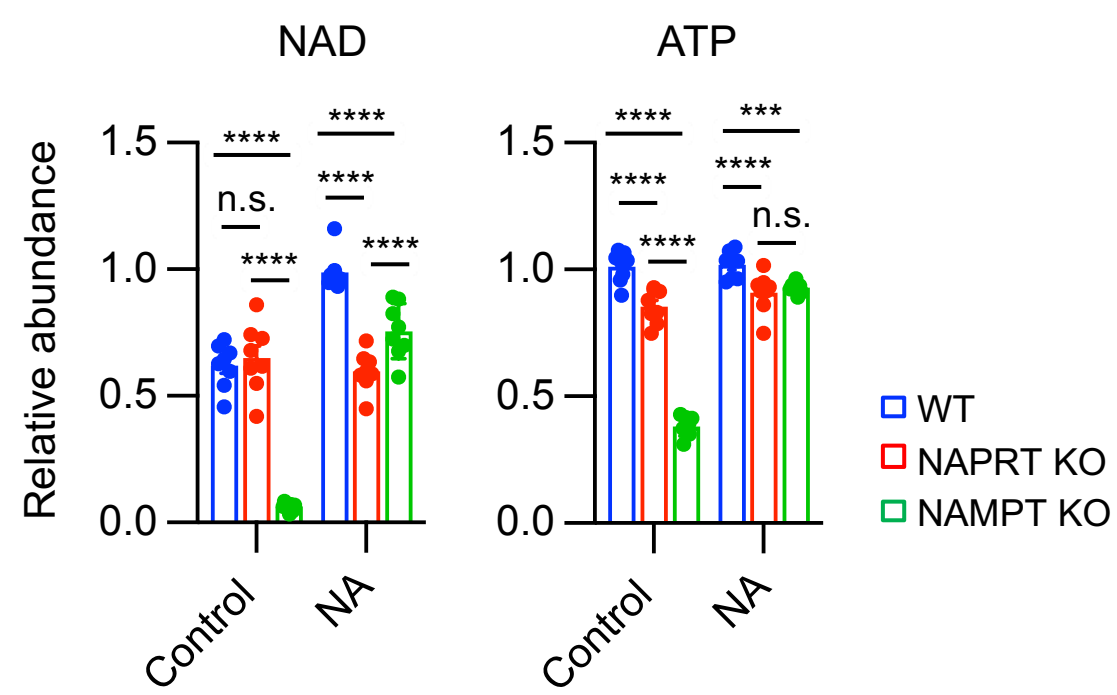

Figure R5. The interaction between deamidated and amidated NAD biosynthesis pathway in CRC119 cells. WT, NAPRT KO, and NAMPT KO CRC119 cells were cultured in regular medium containing 8 μM NAM and with or without 20 μM NA. Their cellular NAD and ATP levels were measured (n=8 replicates/group, values are normalized to the WT NA; 2-way ANOVA, *q<0.05, **q<0.01, ****q<0.0001, n.s., not significant).

When cultured for 24 hours in full medium containing 20 μM NA and 8 μM NAM that enables the activity of both amidated and deamidated pathways (Figure R5, NA), NAPRT KO cells had 40% reduction of their NAD levels, whereas NAMPT KO cells reduced NAD by 25% compared with WT cells, suggesting that when the amidated pathway is inactive, the deamidated pathway is able to compensate more to NAD synthesis than amidated pathway does when the deamidated pathway is inactive in these cells (75% vs 60%). When both pathways were inhibited (Figure R5, NAMPT KO cells, Control), NAD was depleted by 94%, resulting in about 60% depletion of cellular ATP. Thus, in our CRC119 cells, either the amidated or the deamidated pathways are sufficient to maintain cellular ATP levels despite significant NAD reduction. On the other hand, the contribution of the *de novo* pathway appears to be minimal even though the culture medium contains 25 μM tryptophan.

3. The authors claim that their scRNA-seq analysis reveals distinct cell-type expression patterns for NAMPT and NAPRT in the small intestine and colon, with NAMPT being ubiquitously expressed across all cell types, particularly in gut immune cells, while NAPRT is relatively enriched in the epithelial compartments (Figure 1A and S1B). However, this is not entirely accurate, as NAPRT also appears to be expressed in immune cells. Thus, a complete NAPRT knockout could indeed affect NAD+ levels in immune cells, potentially impacting their functional role in reducing tumorigenesis. A conditional knockout mouse model would have been a more suitable approach to target specific cell types for this study. By restricting the NAPRT knockout to particular tissues or cell types, the authors could better assess the cell-type-specific effects of NAD+ depletion on tumorigenesis. Alternatively, the authors could utilize a xenograft or patient-derived xenograft model (that will give some clinical relevance to the

study), comparing NAPRT knockout with NAMPT knockout, to assess the tumor-inducing potential in immunodeficient mice.

The reviewer is right that NAPRT is only relatively enriched in the epithelial compartments, as some immune cells (e.g. CD8 T cells) still express NAPRT. We agree that conditional NAPRT KO in different cell types would be more informative. However, as the reviewer noted here, such studies are beyond the scope of the current revision. The xenograft model also has its drawbacks as it necessitates the use of immunocompromised mice and thus is unsuitable for studying the potential function of NAPRT in immune cells. In addition, we propose defective DNA repair as an important mechanism through which NAPRT KO in epithelial cells enhances tumorigenesis. We believe that this defect is particularly important at the early cancer initiation stages, as it increased the rate of oncogenic mutations. Since the xenograft experiments use established cancer cells that have already accumulated all the mutations needed for malignant growth, further inhibition of DNA repair by NAPRT KO is not expected to further enhance their growth rate. In fact, it can instead decrease the growth rate of xenograft tumors due to excessive DNA damage and reduced metabolic fitness. Such NAPRT knockdown xenograft experiments have already been reported in the literature [6].

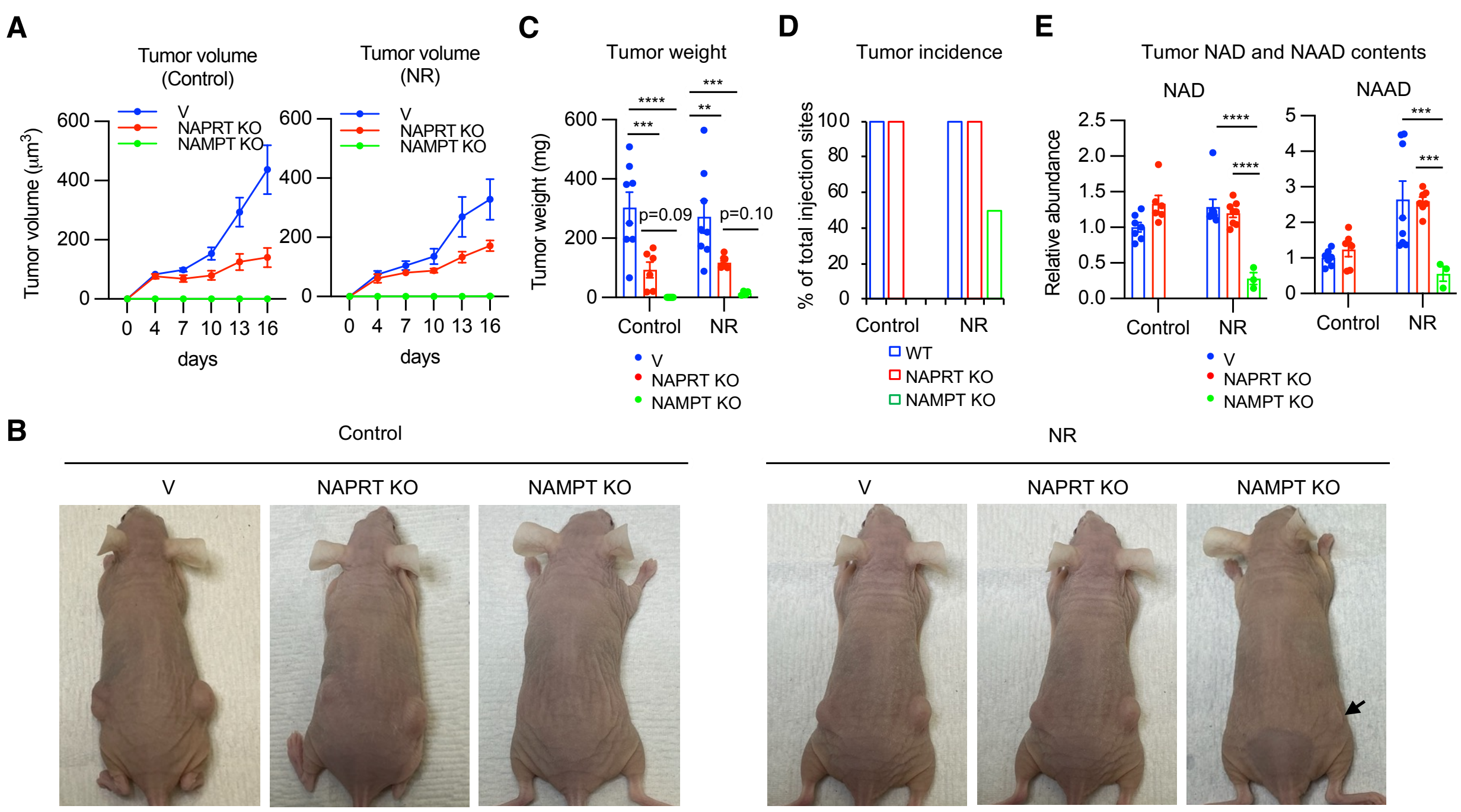

Figure R6. Both amidated and deamidated NAD biosynthesis pathways promote the growth of CRC119 tumors in a xenograft model. Vector control (V), NAPRT KO, and NAMPT KO CRC119 cells (two independent clones for each genotype) were subcutaneously inoculated into nude mice supplemented with or without 3 g/L NR in drinking water. The tumor growth (A), tumor appearance (B), final tumor weights (C), tumor incidence (D), and intratumoral NAD and NAAD contents (E) were analyzed (n=4 mice and 8 injection sites/group, values are expressed as mean ±SEM; 2-way ANOVA, **p<0.01, ***p<0.001; ****p<0.0001).

Keeping these potential drawbacks in mind, per the reviewer's request for this point and point #5, we performed a xenograft experiment using Control CRC119 cells, as well as NAPRT KO and NAMPT KO CRC119 cells generated by the Crispr/Cas9 technology. These cells were then subcutaneously inoculated into nude mice supplemented with or without 3 g/L NR in their drinking water, and tumor growth was monitored over the next 16 days. In support of the notion

that NAD is critical for proliferation and survival of established cancer cells, deletion of NAPRT significantly reduced the growth of xenograft CRC119 tumors, and deletion of NAMPT completely abolished their growth (Figure R6A-6C, control). Supplementation of NR in drinking water failed to significantly affect the growth of tumors from control (V) or NAPRT KO cells (Figure R6A-6C, NR), yet it did partially rescue the growth of NAMPT KO tumors (Figure R6D). Collectively, these results demonstrate that the amidated NAMPT-dependent pathway is crucial for the survival of CRC119 cells in vivo, while the deamidated NAPRT-dependent pathway contributes to tumor growth but is not essential. Notably, LC-MS analysis did not detect any intact NR in the tumors and the partial rescue of tumor growth from NAMPT KO cells was likely mediated by degradation of NR to NAM and deamidation to NA by the gut microbiota [1, 2]. Elevation of tumor NAAD by NR supplementation supports this idea, although the fact that NAAD was equally elevated in NAPRT KO tumors suggests that it was not produced by the tumor cells but rather was imported from circulation or produced by stromal cells (Figure R6E, NAAD).

Here we would like to emphasize that our study is the first to demonstrate that NAPRT can play tumor suppressive rather than tumor promoting function. We believe that this novel conclusion stems from our use of the new genetic NAPRT KO model, which allowed us to study the effects of NAPRT deficiency on the tumor initiation as opposed to the artificial xenograft systems based on established cancer cell lines.

4. In line with the previous comment, the authors should provide a more comprehensive explanation of their results addressing inflammation, in particular when using the DSS-induced colitis models. Reduction of NAD+ levels in their Naprt KO model induces DNA damage and this is well-known to be linked to tumour formation, especially in epithelial cells, but the connection between the reduction of NAD+ levels with inflammation is not clear. Why the reduction of NAD+ levels leads to more inflammation? Which cells contribute to this enhanced sensitivity in their KO mice? Which are the cellular pathways important for this process? Perhaps an immune cell characterization in the presence of DSS in NAPRT KO mice should be provided via scRNA seq of isolated CD45+ cells. The conditional KO mouse model would have solved these questions. We do understand that this is not possible at this stage, but authors could explored alternative experiments such as PDX or/and xenografts studies in immunodeficient mice to circumvent this.

DSS-induced colitis in mice is one of the well-established models to study inflammatory bowel diseases (IBD) [23]. While exactly how DSS, a water-soluble negatively charged sulfated polysaccharide, induces intestinal inflammation is still unclear, the literature suggests that the process begins with DSS damages the epithelial monolayer lining of the colon. This damage allows proinflammatory intestinal contents (such as bacteria and their products) to leak into the underlying tissue, triggering the recruitment of immune cells. However, unlike in human disease, T and B cells are not required for the development of colitis in this experimental model. Therefore, the acute DSS colitis model is particularly useful when studying the contribution of the innate immune system to the development of intestinal inflammation [23].

In terms of how NAPRT deletion leads to increased intestinal inflammation in the DSS-colitis model, our results in Figure 5A-5F demonstrated that NAPRT KO mice experienced increased

colonic epithelial damage after DSS-treatment. Together with the increased expression of various chemokine genes in the KO colon (Figure 5H), these observations suggest that NAD reduction in these mice increases the sensitivity to DSS-induced tissue damage, which in turn enhances the recruitment of immune cells and elevates intestinal inflammation.

5I

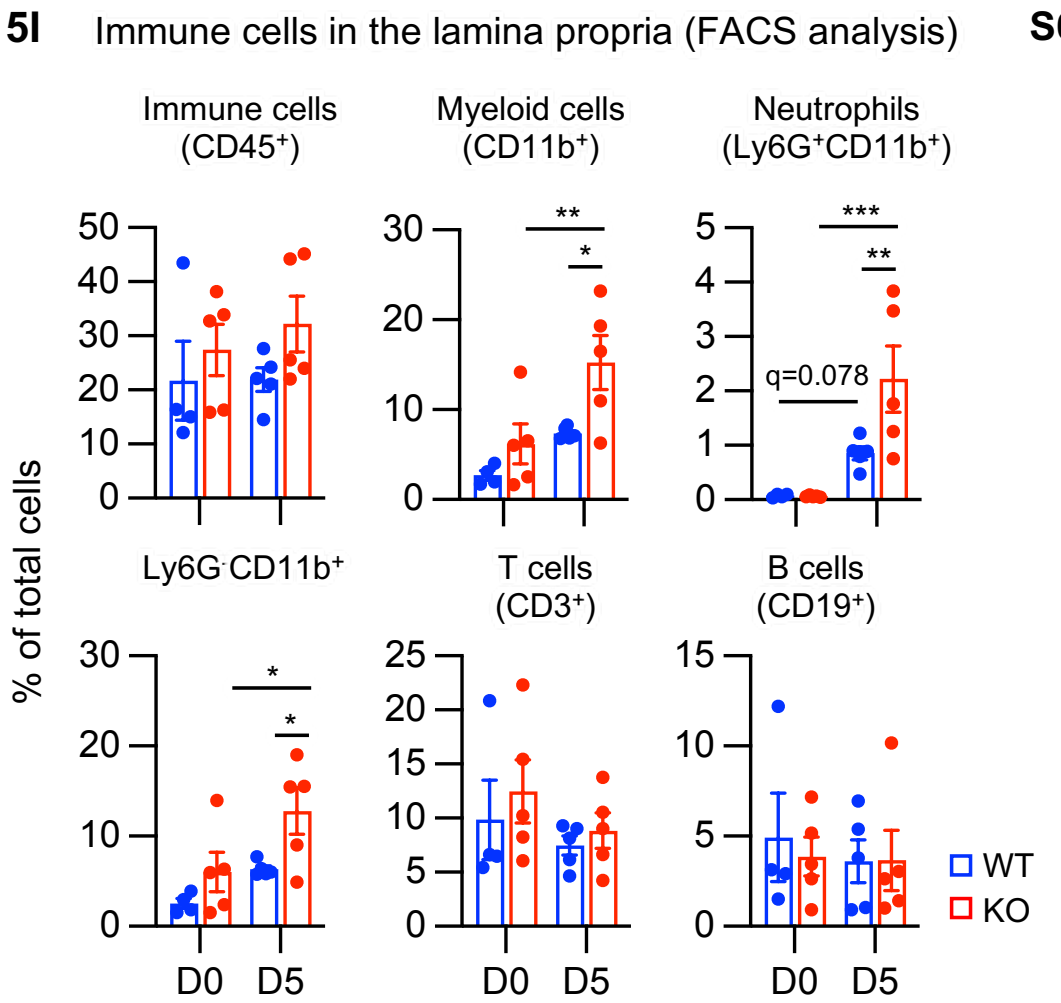

S6I

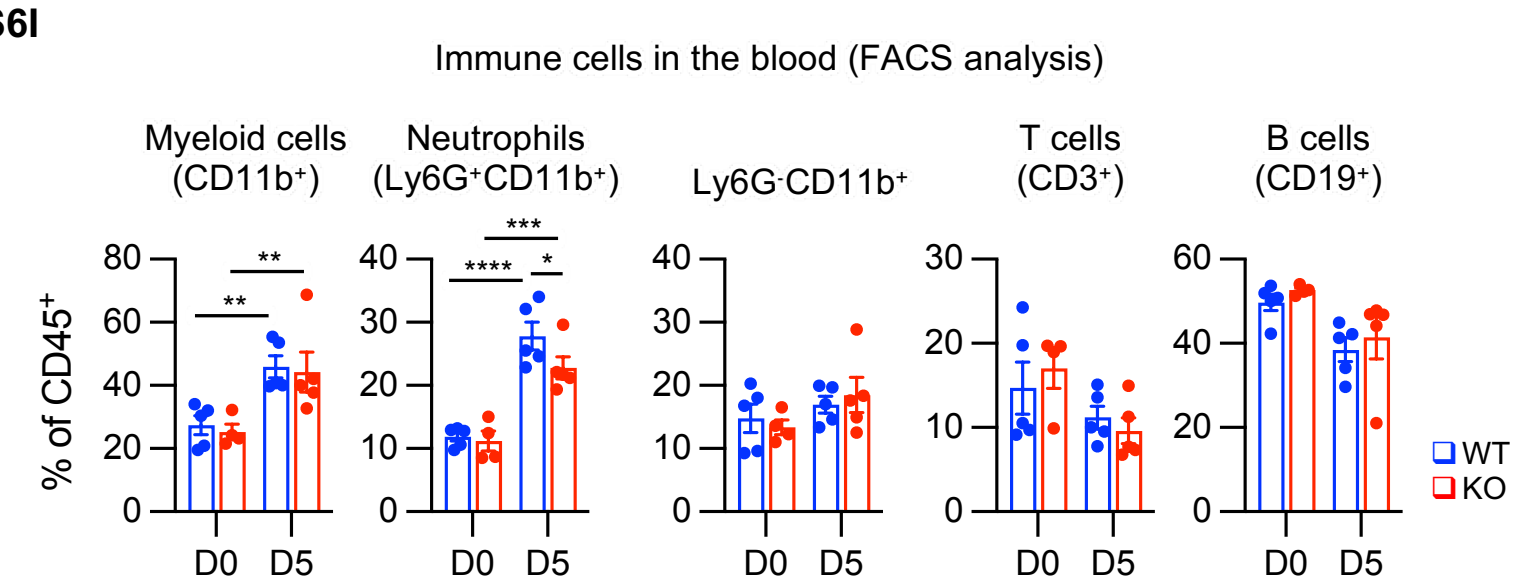

To further address the reviewer's concern on potential mechanisms through which NAD depletion leads to increased inflammation in the DSS colitis, we performed two types of analyses. We first analyzed the populations of immune cells in the lamina propria of the colon and blood before and after five-day treatment of DSS by FACS analysis. Consistent with the literature reports [23], our results showed that the major immune cells responding to DSS-induced colitis are innate immune cells (new Figure 5I and S6I). In WT mice, five-day treatment with DSS increased the abundance of blood myeloid cells, primarily neutrophils (Figure S6I, WT). This increase was coupled with an elevated infiltration of neutrophils into the colonic lamina propria (from 0.07% to 0.86%) (Figure 5I, WT). Deletion of NAPRT did not significantly affect immune cell abundance in either blood or colonic lamina propria under the basal condition (Figure 5I and S6I, D0). However, DSS-induced infiltration of neutrophils into the colonic lamina propria was significantly enhanced (from 0.06% to 2.22%) in these mice (Figure 5I, KO), although their blood neutrophil abundance was slightly lower than that of WT mice after DSS treatment (Figure S6I, D5). DSS-treatment also significantly increased infiltrated Ly6G negative myeloid cells (Figure 5I, Ly6G$^-$CD11b$^+$), which include macrophages, monocytes, and dendritic cells. Together with our observations in Figure 5A-5H, these observations suggest that NAPRT deficiency increases the recruitment of myeloid cells, including neutrophils, into colonic lamina propria in response to DSS-induced epithelial damage.

To better understand how deletion of NAPRT increases the recruitment of myeloid cells upon DSS-induced colitis at the molecular level, we analyzed transcriptomics of all colonic cell types before and after five-day treatment of DSS at the single cell level by scRNA-seq (the KO D0 sample failed to pass the quality control and was therefore removed from the final analysis). Based on the individual cell transcriptome and the expression patterns of known marker genes (Figure S7A), the colonic cells could be categorized into 8 functional groups, including 6 epithelial cell groups and 2 immune cell groups (Figure 6A and Table S4). The two immune cell

groups could be further subclustered into 9 clusters, including 7 immune cell types and two contaminating non-immune cell types (IEC and fibroblasts) (Figure 6B and S7B, Table S4). Consistent with the FACS results in Figure 5I, DSS-treated KO mice had increased abundance of neutrophils in the colon compared to DSS-treated WT mice (Table S4, Neutrophils). In contrast, the abundance of their colonic T cells, particularly a subcluster of T cells expressing some exhausted markers (Figure S7B, T2), were markedly reduced (Table S4, T and T2 cells). Interestingly, in the majority of cell types, the expression of *Nampt* was significantly induced by DSS treatment, whereas the expression of *Naprt* was not significantly affected (Figure 6C and S7C, WT D5 vs WT D0), suggesting the differential functions of these two NAD biosynthesis pathways in response to DSS-induced colitis. Transcriptomic analysis of colonocytes, the cell type with the highest expression level of *Naprt* (Figure 6C, CC), showed that DSS treatment induced the expression of genes associated with membrane trafficking, G-protein coupled cell signaling, and adherens junctions, while reducing the expression of genes involved in lipid transport, digestive system, and lipid metabolism (Figure S7D). Venn-diagram analysis further revealed that among the genes induced by DSS in colonocytes of WT mice, deletion of NAPRT further enhanced the expression of genes involved in the defense response, particularly those related to antigen processing and presentation, but suppressed the induction of genes responsible for intracellular structure maintenance and protein quality control (Figure 6D and 6E). These key transcriptomic changes were consistently observed across all annotated cell types, including both gut epithelial cells and immune cells (Figure 6F and S7E), suggesting that the enhanced intestinal inflammation observed in DSS-treated KO mice is associated with increased anti-microbial defense response and reduced maintenance of tissue integrity.

Taken together, in agreement with our pathological, biochemical, and qPCR evaluations in Figure 5A-5H, our FACS and scRNA-seq analyses indicate that inactivation of the deamidated NAD biosynthesis in NAPRT-deficient colon leads to reduced maintenance of tissue integrity and elevated anti-microbial response upon DSS challenge, which enhance recruitment of myeloid cells (particularly neutrophils) and induce intestinal inflammation.

5. A rescue experiment using nicotinamide riboside (NR) both in vitro (Figure 1) and in vivo to counteract the effects of NAPRT depletion on tumorigenesis would be highly valuable. For tumorigenesis, NR should be implemented in prevention and in therapy once tumors are formed to better understand the pro- or anti-tumorigenic effects of NAPRT KO. This experiment could provide direct evidence for the potential therapeutic role of NR in restoring NAD+ levels and mitigating the negative effects of NAD+ depletion on DNA repair, inflammation, and tumorigenesis. Including such data would significantly strengthen the manuscript by demonstrating the feasibility of NR as a potential intervention for restoring NAD+ homeostasis and reducing cancer progression in the context of NAPRT deficiency.

Again, a rescue experiment using an alternative NAD precursor independently of the NAPRT-mediated deamidated NAD biosynthesis is a great idea. However, as stated in our response to point #1, we were not able to obtain a suitable NAD precursor for this type of rescue experiment. Particularly, NR is not the right choice as orally administered NR largely boosts tissue NAD biosynthesis via NAPRT-mediated deamidated NAD biosynthesis [1, 2], and is therefore unlikely alleviate NAD deficiency in NAPRT KO mice. NR was also unable to effectively boost NAD levels at physiologically relevant concentrations in vitro in our CRC119 cells (Figure R4).

On the other hand, dietary supplementation of NR has been shown to protect against liver tumor formation in mice with intact NAPRT by increasing hepatic NAD concentrations and preventing DNA damage and inflammation [13, 14]. Given that dietary NR relies heavily on NAPRT-mediated deamidated NAD biosynthesis to increase tissue NAD levels [1, 2], these observations are consistent with our findings in this study. They suggest that boosting deamidated NAD biosynthesis through diet helps prevent tumor formation in tissues by enhancing DNA repair.

To further test the therapeutic potential of dietary NAD boosting supplements in cancer, as the reviewer suggested here, we performed a xenograft CRC model with or without supplementation of NR in drinking water (Figure R6). However, NR failed to increase the NAD content in either WT or NAPRT KO tumors (Figure R6E, NAD). We also did not observe any significant inhibition of tumor growth (Figure R6A-R6C) or significant reduction of tumor incidence (Figure R6D). Instead, we found that deletion of NAPRT significantly reduced the growth of xenograft CRC119 tumors, and deletion of NAMPT completely abolished their growth (Figure R6A-R6C, control). Moreover, NR supplementation increased the incidence of NAMPT KO tumors that still had intact NAPRT-mediated deamidated NAD biosynthesis (Figure R6D). Together, our observations support the notion that in established late-stage cancer cells, NAD is critical for their proliferation and survival.

We agree with the reviewer that evaluation of NAD-boosting supplements for cancer prevention in different cancer types is a promising idea, especially given the clinical use of NAM for melanoma prevention[24]. However, such experiments would require long experiments beyond the time frame of this revision. We plan to perform these studies in the future and report the results in a separate publication.

6. While this study introduces the first NAPRT KO mouse model to explore the role of the Preiss-Handler pathway in tumorigenesis, the concept of NAD+ depletion in DNA damage and cancer is not novel. The authors overlook the foundational work by Tummala et al., published in Cancer Cell in 2014, which first demonstrated the significance of NAD+ depletion in tumorigenesis. Additionally, the study by Lee et al., published in Cancer Discovery in 2022, has been ignored. In their work, the authors showed that APC-deficient colorectal cancer models were susceptible to TDO2 depletion or pharmacological inhibition, leading to NAD+ depletion, impaired cancer cell proliferation, and enhanced antitumor immune responses. These important studies should be cited, discussed, and integrated into the context of the current manuscript through experimental evidence. One possible explanation to the study of Lee et al. is that since APC deletion drives cell proliferation, cells might rely on increased NAD+ from de novo synthesis to initiate DNA synthesis and repair pathways. Without the support of the NAPRT-NAD+ arm, APC-deleted cells may not survive. Therefore, it is crucial to measure NAD+ levels under different conditions in order to determine how the depletion of NAPRT affects cellular NAD+ metabolism, especially in the context of APC deletion. This would help clarify whether NAPRT-mediated NAD+ synthesis is essential for the survival and function of APC-deleted cells and further elucidate the role of NAD+ in DNA repair and cell survival under genotoxic stress.

Yes, as stated in our response to #1, a wealth of literature has already established the connection between DNA damage and NAD depletion [7-10] and reviewed in [11, 12]. However, the focus of our current study is on the sources of NAD for DNA repair in gut epithelial cells instead of this previously established link. Our data showed that by providing a major source of NAD for DNA repair in the gut epithelial cells, NAPRT-mediated deamidated NAD biosynthesis is tumor-suppressing. As discussed in our manuscript, this finding is unexpected and novel.

Again, as mentioned in our response to #1, we did not cite Tummala et al. [13] in the original manuscript because our study is focused on the deamidated NAD biosynthesis in the gut while Tummala et al. investigated de novo NAD biosynthesis in the liver. We cited this study, as well as Gomes et al. [14], together with above literature in the revision.

Per the suggestion of the reviewer, we also carefully read the study by Lee et al. published in Cancer Discovery in 2022 [25]. However, we failed to see any data related to the link between TDO2 inhibition and NAD depletion in APC-deficient CRC the reviewer mentioned here. Instead, the authors provided evidence that increase of TDO2 in APC-deficient CRC enhances cancer cell proliferation and impairs antitumor immune response through aryl hydrocarbon receptor (AhR) signaling, as Kyn is a known agonist for AhR.

S8E

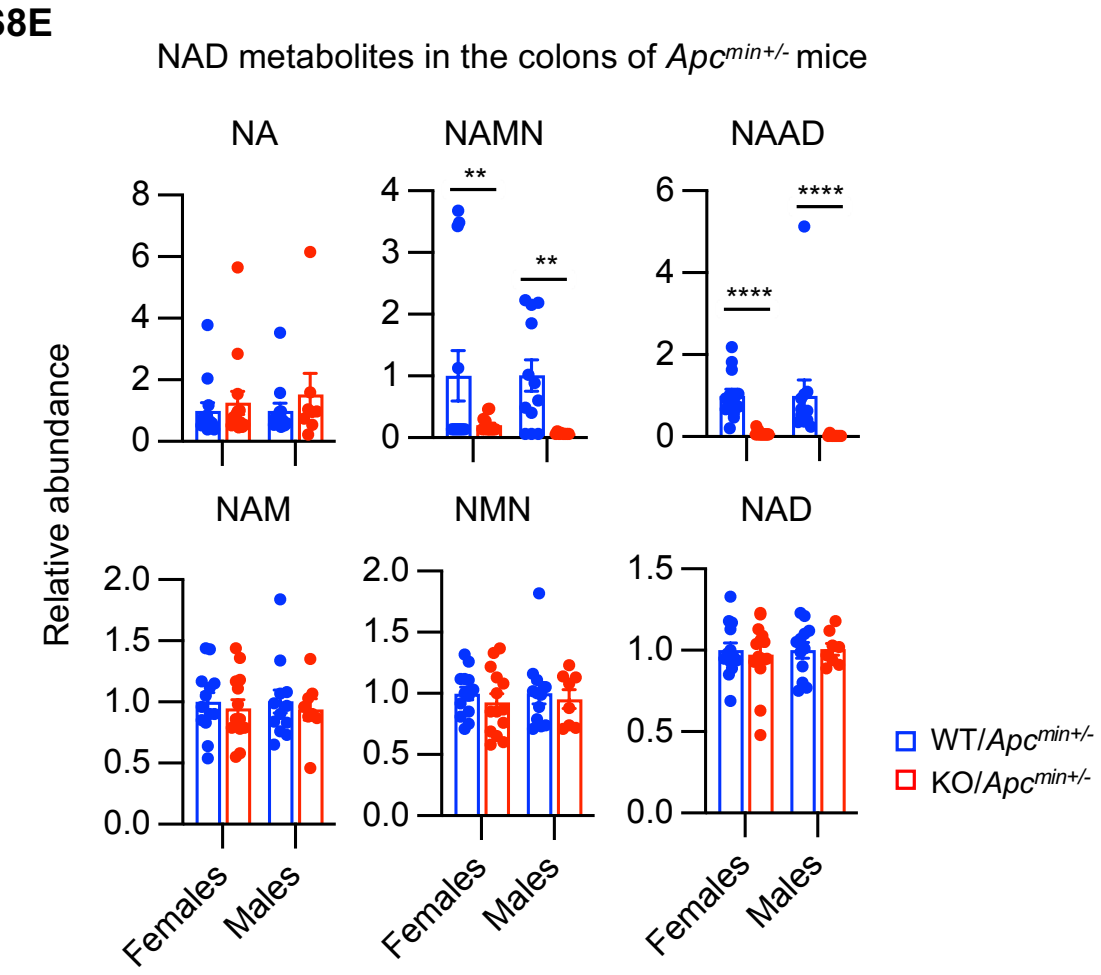

To test the possibility that "without the support of the NAPRT-NAD arm, APC-deleted cells may not survive", as the reviewer suggested, we measured NAD metabolites in the colons of WT and NAPRT KO mice on the *$Apc^{min+/-}$* background at the end of experiment (Figure S8E). As expected, NAPRT KO mice still displayed impaired deamidated NAD biosynthesis, as indicated by the very low NAMN and NAAD levels (Figure S8E, NAMN and NAAD). However, we did not find any differences in the NAD levels between WT and NAPRT KO mice at this stage (Figure S8E, NAD). It is possible that NAD levels were mildly affected by NAPRT KO in some cells, but this difference was too mild to be detectable in bulk colon samples. Therefore, NAPRT-mediated deamidated NAD synthesis is not essential for the survival and function of APC-deleted cells in vivo, and its deficiency is likely compensated by the amidated, maybe even the *de novo*, biosynthesis in *$Apc^{min+/-}$* mice.

7. Figure 2D is difficult to understand, and the data interpretation lacks clarity. The authors claim that STF treatment sensitizes cells to MMS-induced cell death by comparing WT MMS+STF to WT MMS alone. However, when discussing the inactivation of deamidated synthesis in KO cells, the authors compare KO MMS to WT MMS instead, which seems inconsistent. According to Figure 2C, KO cells appear to have more DNA damage; therefore, in Figure 2D, MMS treatment alone would be expected to result in fewer colonies in KO cells compared to WT, but

this outcome is not shown. Furthermore, if the comparison in KO cells were done similarly to WT cells—that is, MMS+STF vs. MMS alone—a similar observation (fewer colonies with STF treatment) would likely emerge. The precise methodology of this experiment, particularly the comparisons drawn between groups, needs more explanation.

We apologize for the "lack of clarity" in describing the methodology of this experiment (now in Figure 2E). In this study, we used STF treatment in WT cells to inactivate the amidated pathway (WT STF), and NAPRT KO to inactivate the deamidated pathway, and STF treatment in NAPRT KO cells (KO STF) to inactivate both amidated and deamidated pathways. Thus, the comparison between WT MMS+STF vs WT MMS alone assessed the effect of amidated pathway inactivation on cell survival upon MMS treatment. The comparison between KO MMS vs WT MMS evaluated the impact of deamidated pathway inactivation on cell survival upon MMS treatment. The comparison between KO MMS+STF and KO MMS alone, as suggested by the reviewer, is to compare the impacts of inactivating both pathways vs only the deamidated pathway on cell survival. In short, WT STF is equivalent to NAMPT KO. We hope this clears up any confusion.

2F NAD and ATP in NAPRT or NAMPT KO CRC119 cells

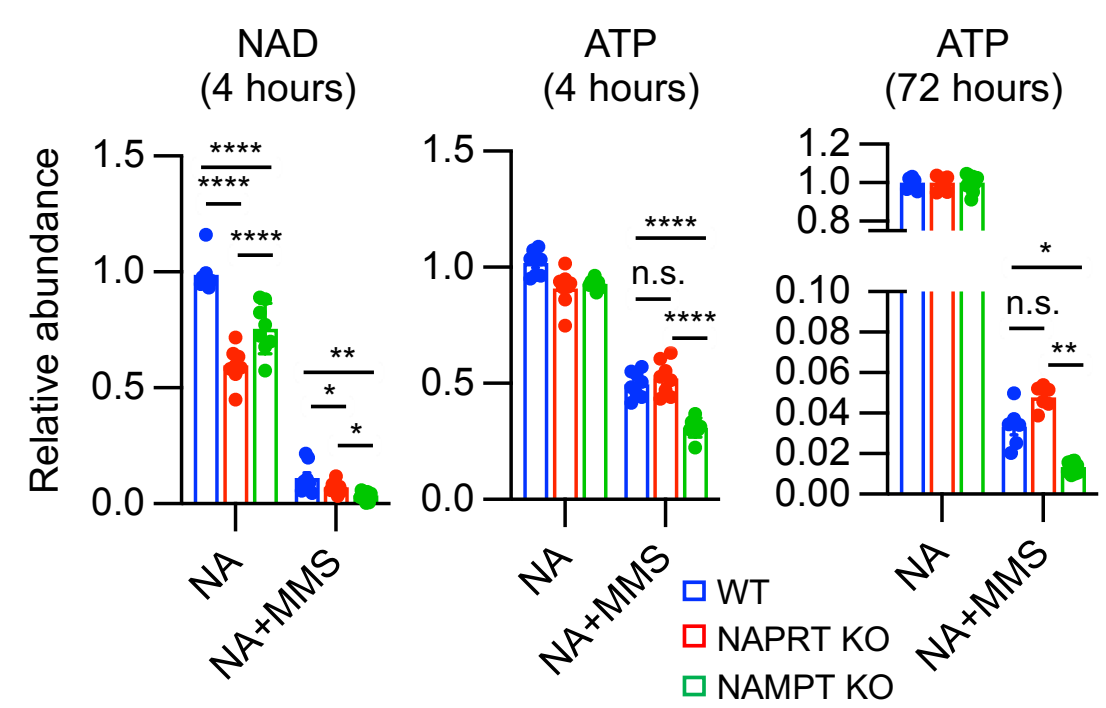

To further make our data interpretation more straightforward, we generated NAMPT KO CRC119 cell using the crispr/cas9 gene editing technology (Figure S2G). We then cultured WT, NAPRT KO, and NAMPT KO cells in full medium containing 20 μM NA and 8 μM NAM, and treated them with or without MMS for 4 hours or 72 hours (Figure 2F). Despite a significant NAD reduction (Figure 2F, NA; NAD (4 hours)), either the amidated or the deamidated pathways were sufficient to maintain cellular ATP levels in this full medium (NA; ATP (4 hours)). Treatment with MMS for 4 hours depleted cellular NAD levels by 90% in all cells and markedly reduced cellular ATP levels. Yet compared to WT cells, NAPRT KO cells displayed a comparable cellular ATP level whereas NAMPT KO cells had significantly reduced cellular ATP (NA+MMS; ATP (4 hours)). Similar results were observed after cells were treated with MMS for 72 hours (NA+MMS; ATP (72 hours)). These observations are consistent with our colony formation assay in Figure 2E, suggesting that NAPRT KO CRC119 cells have a comparable survival ability as the WT cells in response to the genotoxic stress.

Regarding the "inconsistency" between the DNA damage data in Figure 2C and the colony formation data in Figure 2D, the "not shown" of "fewer colonies of KO cells", as expected by the reviewer, is the exact reason why we concluded that despite the accumulation of more severe DNA damage, NAPRT KO gut epithelial cells are able to survive the genotoxic stress similarly to WT cells. The elevated DNA damage in these surviving cells could potentially increase mutagenesis and tumorigenesis. We rephrased the original conclusion as: "*However, despite the accumulation of more severe DNA damage compared to WT cells, a comparable fraction of NAPRT-deficient gut epithelial cells survives the stress. The elevated DNA damage in these surviving NAPRT KO cells could potentially increase tumorigenesis*." We hope it now makes

more sense to the reviewer.

Minor points

1. Some data described in the text is not shown in the figures (e.g. Figure 1 is missing to show cell morphology and proliferation rates).

Thanks for pointing this out. We added data on the cell morphology and proliferation rates into new Figure S1E and S1F.

2. Some panels of the figures are not even mentioned in the text (e.g. Figure 2A). All data shown in the figures must be discussed.

We did not discuss NA and NAM data because both metabolites were present at high levels in the culture medium. Although the cells were washed prior to harvesting, residual amounts from the medium could have interfered with their measurement. ADPR levels were intended to assess the activity of PARPs. However, ADPR is not a direct product of PARP but rather an indirect product of protein PARylation and MARylation, followed by PARG-mediated hydrolysis. For these reasons, we decided to remove these three panels from the revised manuscript.

3. Figure legends contain explanations already present in the results section. They should be more descriptive and summarized.

Thanks for pointing this out. Our initial goal was to make each figure self-explanatory, eliminating the need to refer to the main text and Methods section. In the revision, we tried our best to simplify the figure legends. However, as the experimental parameters, such as doses, times, etc., vary from figure to figure, it is impossible to give the details in the Methods section for each figure. Additionally, NIH mandates us to provide experimental details to enable reproducible research. As a result, the revised figure legends still contain some experimental details.

4. Full uncut immunoblots should not be added in the supplementary figures (Fig. S1D, Fig. S2B).

We included all uncut immunoblots into a separate file and provide it as an independent supplementary document in the revision.

5. In lines 141-142, the authors claim that ‘‘ NAPRT deficiency impairs cellular metabolism and increases the sensitivity of gut epithelial cells to NAMPT inhibition induced NAD depletion and subsequence cell death’’. However, the authors should provide an experiment that shows that cell death occurs such as Brdu incorporation or stainings for apoptotic markers.

To address this question, we performed new propidium iodide (PI) exclusion flow cytometry assay in WT and NAPRT KO cells treated with or without 20 μM NA and 100 nM STF for 42

hours. We chose to use propidium iodide exclusion because cell death following NAD depletion is usually necrosis rather than apoptosis. Our analysis revealed that in the absence of NA, 42-hour treatment of STF induces massive cell death in both WT and NAPRT KO cells. This death could be rescued by NA in WT but not in NAPRT KO cells (new Figure 1G), supporting our conclusion that "NAPRT deficiency increases the sensitivity of gut epithelial cells to NAMPT inhibition-induced cell death".

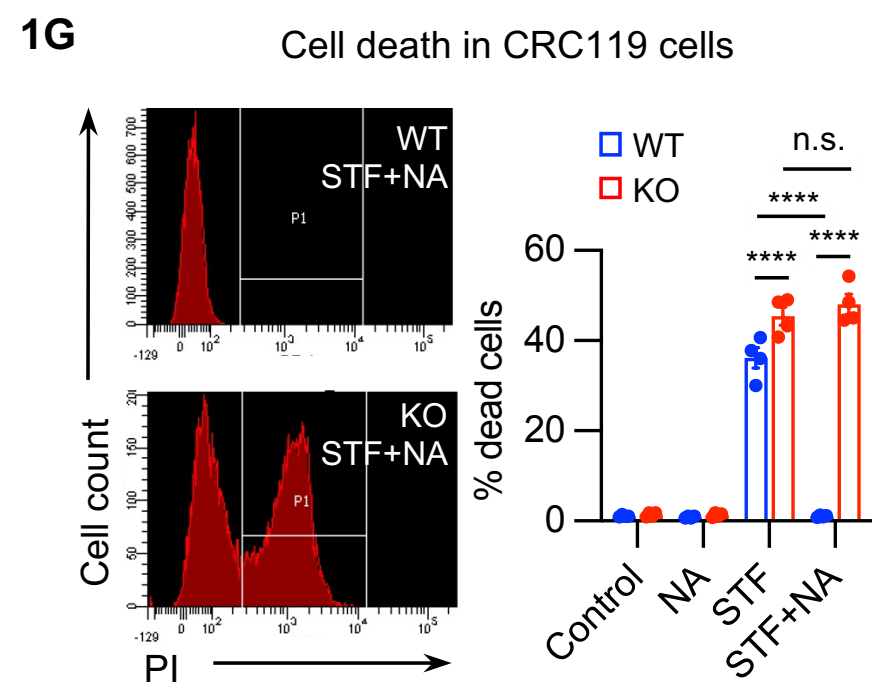

6. The authors should consider changing the title of the manuscript since it does not align with the findings shown.

We are sorry to know that the reviewer thought that the title of our manuscript was not supported by our findings. We hope that our revision addressed this concern.

7. Figure 3D, quantify the number of organoids

The images in Figure 3D are representative only to show the differences in organoid morphology and size. The numbers of organoids did not change significantly by the treatments as dead organoids were still present in Matrigel. That's the reason why we used Cell Titer Glo (CTG) assay to quantify the live organoids. The results of this quantification are shown in Figure 3E.

8. The authors explain the observed phenotypes due to the accumulation of DNA damage due to NAD+ depletion. Therefore, DNA damage measures are key to support their conclusions. In this regard, authors rely on yH2AX staining quantified by intensity/cell. The use of additional DNA markers for DNA damage, beyond yH2AX, should be also included to further corroborate their results.

To address this question, we consulted several experts in the DNA repair field and conducted additional assays, including alkaline comet assay, long-range PCR of the β-globin gene, and TUNEL staining. However, due to the large clonal variability and limitations in assay sensitivity, these approaches did not produce reliable results.

On the other hand, as noted in our response to Major point #1, we analyzed metabolites related to dNTP biosynthesis using a metabolomic dataset from AOM-treated mouse colons. We found that several deoxyribonucleosides, including 2'-deoxycytidine, 2'-deoxyuridine, and thymidine, were markedly increased upon AOM-induced DNA damage. In both WT and NAPRT KO colons, these nucleosides were elevated following AOM treatment, peaking at 12 hours post-injection when DNA damage was at its highest (Figure 4C), suggesting that DNA damaged-induced DNA turnover and repair may be the sources of these nucleosides [17]. Notably, 2'-

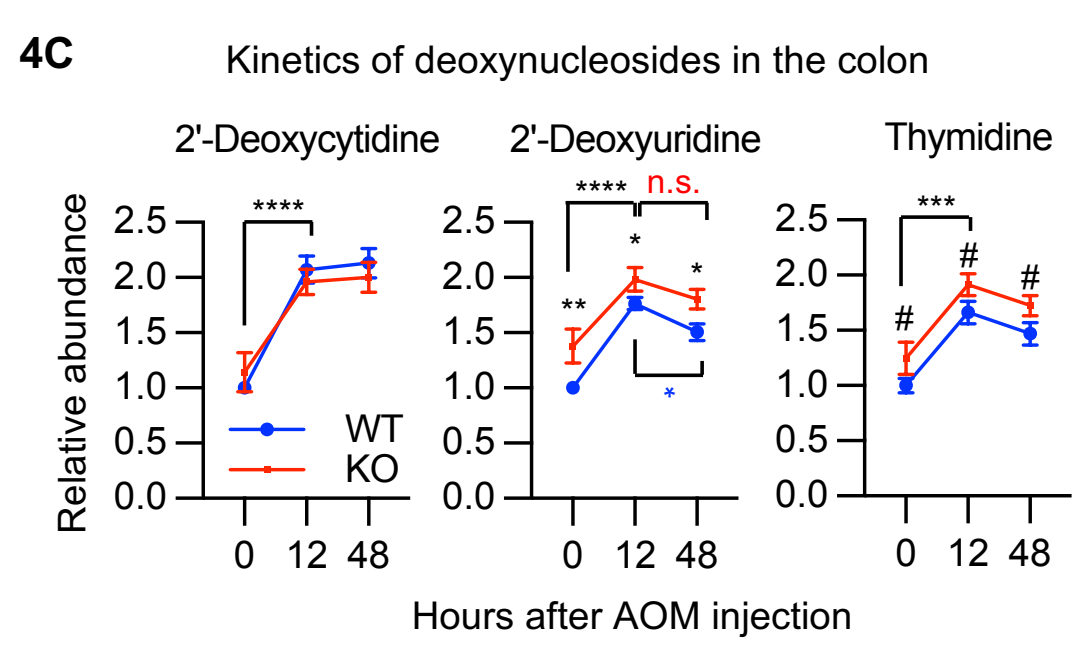

deoxyuridine levels were significantly higher in NAPRT KO colons than in WT at all time points and did not show substantial recovery at 48 hours (Figure 4C, 2'-deoxyuridine), suggesting increased and sustained DNA damage in the absence of NAPRT.

In addition, measure of DNA damage in their NARPT KO aged mice is also requested.

Again, we tried to quantify DNA damage in the colons of aged WT and NAPRT KO mice using different methods, including IF staining of γH2AX, immunoblotting of γH2AX, and TUNEL staining. However, the staining signals from all methods were too weak to be reliably quantified.

9. The authors should show the activation of DNA repair pathways in gut epithelial cells upon DNA damage.

We have data showing that PARylation and MARylation were induced after MMS treatment in CRC119 cells (Figure 2B, S2B, and S2C), indicating the activation of the PARP activity following MMS induced DNA damage. Our results further revealed that the activation of the PARP was transient and peaked around 1 hour after the treatment, and deletion of NAPRT attenuated the activation of PARP (Figure 2B and S2B).

10. Microbiome or diet are the source of NA is their study? The relevance of the microbiome could be explored via antibiotics treatment to get insights into its role.

This is a great suggestion. As stated in our manuscript as well as in our early responses, this question was the topic of our previous study by Shats et al. in Cell Metabolism [1]. Here is the abstract of that study "*Nicotinamide adenine dinucleotide (NAD), a cofactor for hundreds of metabolic reactions in all cell types, plays an essential role in metabolism, DNA repair, and aging. However, how NAD metabolism is impacted by the environment remains unclear. Here, we report an unexpected trans-kingdom cooperation between bacteria and mammalian cells wherein bacteria contribute to host NAD biosynthesis. Bacteria confer resistance to inhibitors of NAMPT, the rate limiting enzyme in the amidated NAD salvage pathway, in cancer cells and xenograft tumors. Mechanistically, a microbial nicotinamidase (PncA) that converts nicotinamide to nicotinic acid, a precursor in the alternative deamidated NAD salvage pathway, is necessary and sufficient for this protective effect. Using stable isotope tracing and microbiota-depleted mice, we demonstrate that this bacteria-mediated deamidation contributes substantially to the NAD-boosting effect of oral nicotinamide and nicotinamide riboside supplementation in several tissues. Collectively, our findings reveal an important role of bacteria-enabled deamidated pathway in host NAD metabolism.*" This finding was subsequently confirmed and extended by Chellappa et al. [2]. It is now well recognized in the NAD metabolism field that oral NAD boosting supplements, such as NAM, NR, and NMN, are all prodrugs of NA due to the activity of gut microbiota.

We strongly encourage the reviewer to read the above two papers. We believe they can address the major concerns/questions the reviewer had for our current study.

Reviewer #3 (Remarks to the Author):

The manuscript by Xiaoyue and colleagues "NAPRT-mediated deamidated NAD biosynthesis enhances tissue resiliency and suppresses tumorigenesis," aims to investigate the tumour suppressing roles of the deamidated arm of NAD biosynthesis. To achieve that, they constructed NAPRT gene knock-out models and assess their effects using both in vitro (CRC119 cell lines and colonic organoids) and in vivo (mouse) systems. Here they found that removal of NAPRT gene altered metabolism such as glycolysis, increased DNA damage, impaired PARP activity, heightened sensitivity to inflammatory stress, and susceptibility to tumorigenesis in mouse models. Furthermore, reduced NAPRT expression correlates with poor prognosis in stage 1, not stage 3 and 4, CRC patients.

The manuscript is well-written, and the analyses are thorough and comprehensive. In my view, the findings presented in this manuscript are valuable and provide some insights into the mechanisms underlying colorectal cancer (CRC) development. Additionally, these insight may also extend to understanding precancerous conditions, such as polyps, offering a foundation for further research in this area.

However, I have a few comments that I hope the authors will address to strengthen the manuscript further:

1. The authors focus their study on gut, but occasionally deviate and discuss NAPRT expression in other organs without clear purpose or sufficient discussion, which may confuse the reader. For example, it is not clear why the author show NAD metabolites in liver instead of colon in figure 3A?

We originally presented NAD metabolites in the liver instead of colon in Figure 3A because our previous[1] and current studies showed that the liver is the organ where the deamidated pathway contributes the most to total tissue NAD biosynthesis (approximately 90%) following NAM supplementation. In the colon, this contribution is around 66%. We agree with the reviewer that focusing on the gut will enhance clarity for readers in this study. We therefore replaced the Figure 3A with colon data and moved the liver data into Figure S3E.

2. For NAPRT/NAPRT expression in humans (Fig. 1A or Fig. 6I), it would be helpful to stratify the data based on previously published transcriptomic subtypes, such as CMS (https://doi.org/10.1038/nm.3967), IMF (https://doi.org/10.1038/s41588-022-01100-4), or MSI/MSS, to provide a more detailed understanding.

Figure 1A is about the expression of *Nampt* and *Naprt* in normal mouse small intestine and colon. We stratified the data in Figure 6I based on the reviewer suggestion using CMS [26] and the new results are shown in new Figure 8C and 8D.

As shown in Figure 8C, all four CRC subtypes had more tumors with an elevated expression of *NAMPT* than those with low expression of this gene. Notably, CMS1, the subtype characterized by increased immune infiltration and strong activation of immune evasion pathways [26], had the highest proportion of *NAMPT*-high tumors. On the other hand, CMS4, the subtype that shows upregulation of epithelial mesenchymal transition (EMT) and activation of transforming growth factor β (TGF β) [26], had the highest proportion of *NAPRT*-low tumors (Figure 8C, *NAPRT*).

8C

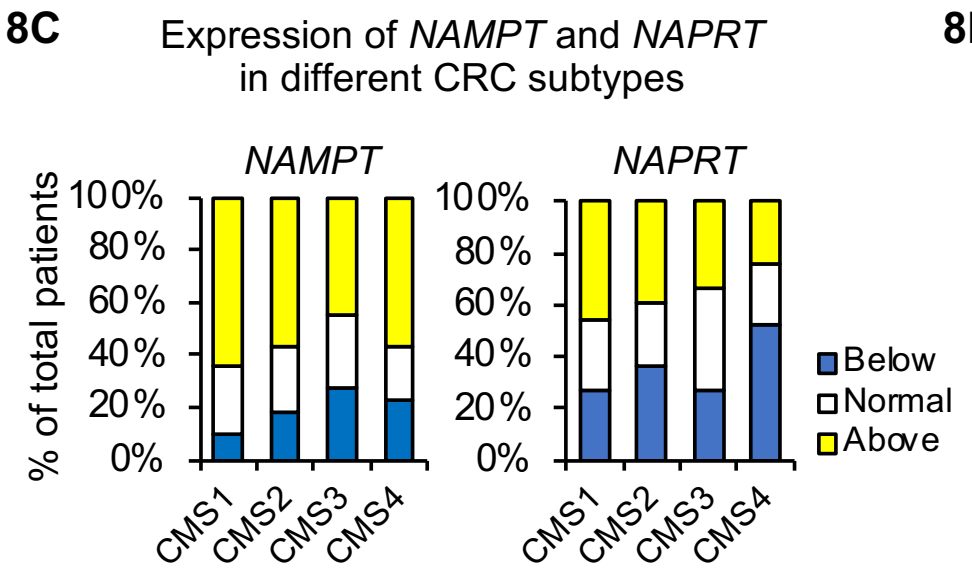

8D

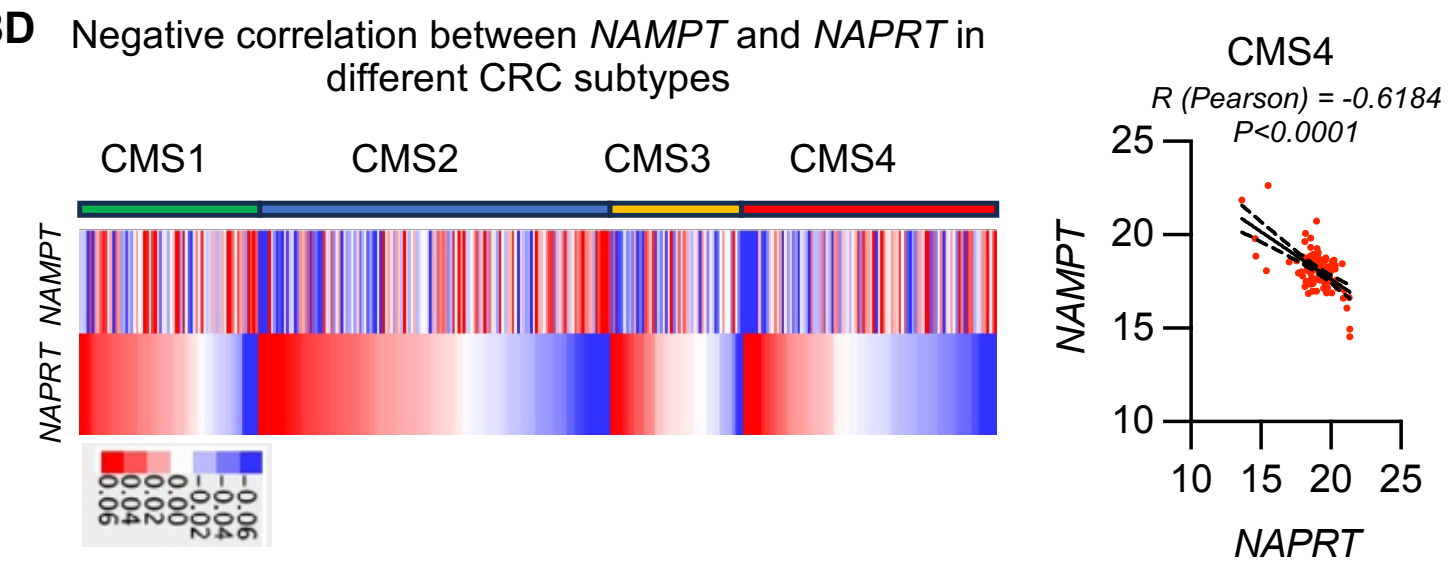

These findings are consistent with our observations that NAMPT is enriched in immune cells whereas NAPRT is predominantly expressed in epithelial cells (Figure 1A, 1B, S2B, S2C, 6C, and S7C). Intriguingly, the expression levels of *NAMPT* and *NAPRT* were negatively correlated in CRC, especially in the CMS4 subtype that displays worse overall and relapse-free survival compared to other subtypes [26] (Figure 8D), highlighting the subtype-specific roles of these two NAD biosynthesis pathways in intestinal tumorigenesis.

3. It would also be interesting to investigate whether there is any dysregulation of NAPRT in the polyps. The authors could consider utilizing previously published polyp datasets, such as those from Chen et al. (Cell, Volume 184, Issue 26, 6262 - 6280.e26) or Becker et al. (Nat Genet 54, 985–995 (2022)), to explore this further.

S8G

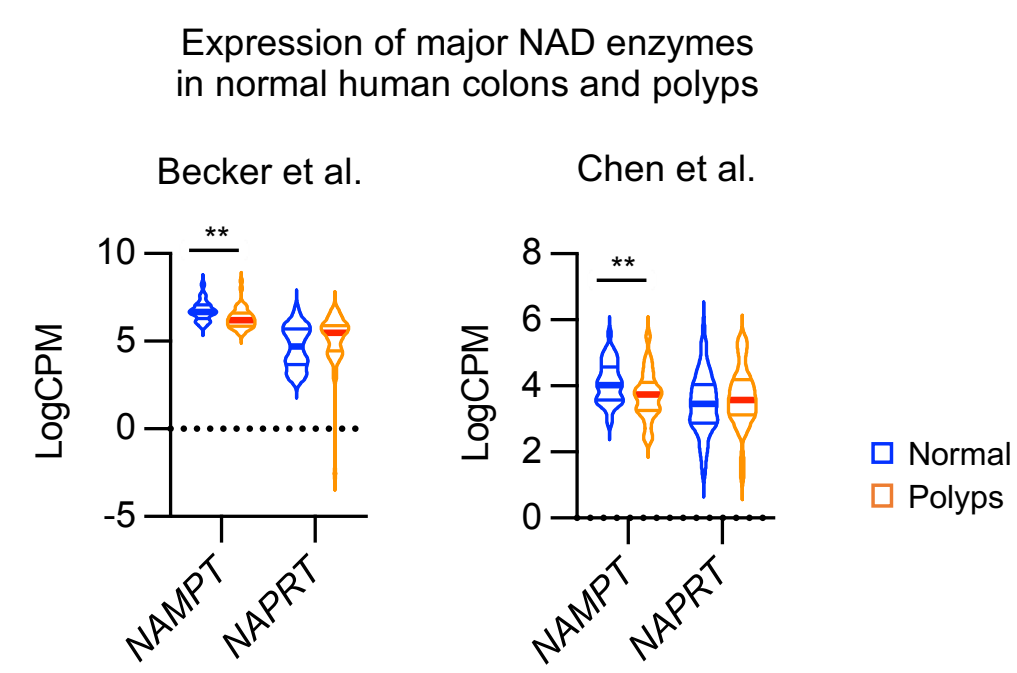

Thanks for the suggestion. We analyzed the expression of *NAMPT* and *NAPRT* in scRNA-seq datasets from both studies by combining counts from all cell types and the results are shown in Figure S8G. Interestingly, compared to normal human colon tissues, colorectal polyps showed significantly reduced expression of *NAMPT* but normal levels of *NAPRT* in both datasets. This observation supports our conclusion that deamidated and amidated NAD biosynthesis have distinct roles in regulation of intestinal tissue homeostasis.

4. The mechanism behind the increase in NA following NAM feeding in the mouse model is unclear. The authors suggest that gut bacteria may convert dietary NAM to NA, but a similar increase in NA was also observed in other organs (Fig. 3C). Further explanation are needed.

The reason why we did not provide a detailed description of the mechanism behind the increase in NA following NAM feeding is because this was the topic of our previous study by Shats et al. in Cell Metabolism [1]. In that study, using stable isotope tracing and microbiota-depleted mice, we demonstrated that orally administrated NAM (as well as NR) is deamidated to NA by the pncA activity of gut microbiota. This NA is then absorbed from the gut and enters the systemic circulation, boosting NAD biosynthesis in other tissues through the deamidated pathway. This finding was subsequently confirmed and extended by Chellappa et al. [2].

Please note that the massive NA accumulation in different tissues was only observed in NAPRT KO mice because without NAPRT, NA cannot be assimilated into NAD and is therefore

accumulated in all tissues. In WT mice, on the other hand, circulating NA is rapidly metabolized by the tissues to build NAD through the deamidated pathway, resulting in very low levels of NA in tissues.

5. It is unclear why NA was used as the substrate in the in vitro experiments, while NAM was used in the in vivo studies. The authors may consider using NAM as the substrate in the in vitro system as well, which could also help address my previous question (point 4).

We are sorry that we did not explain this clearly in the paper. Regular cell culture media already contain NAM but not NA. Since mammalian cells lack an enzyme that can convert NAM to NA, supplementation with NA is needed to enable the deamidated pathway so that its contribution to overall NAD biosynthesis can be assessed. In contrast, as discussed in our response to #4, we and others have demonstrated that following oral administration of amidated NAD boosting precursors such as NAM or NR, gut microbiota converts them to NA in vivo [1,2]. Therefore, oral supplementation with either NAM or NA activates the deamidated pathway in vivo. Since currently such amidated precursors are more popular than NA in the field of NAD supplementation, we used NAM supplementation in some of our in vivo experiments. On the other hand, in our DNA damage experiment on Figure 4, we directly used NA to activate the deamidated pathway.

We modified the text as follows to better explain the rationale of using NA or NAM in our experiments: (1) On page 7, we explained the reason for supplementation of NA to the regular culture medium "*NAPRT KO CRC119 cells displayed normal morphology and proliferation rates when cultured in a regular growth medium containing 8 μM NAM but no NA (Figure S1E and S1F). Supplementation of 100 μM NA, a concentration comparable to the serum NA range after oral supplementation with NAD boosting precursors [21], markedly induced deamidated NAD synthesis and significantly increased total NAD abundance in WT but not in NAPRT KO CRC119 cells (Figure 1C and 1D).*" (2) On page 11, we stated that "*As previously elucidated, the induction of the deamidated NAD precursors NAMN and NAAD in WT mice following supplementation with NAM is a result of gut microbiota-mediated deamidation of NAM to NA [20, 21].*"

6. Figure 4A, why NA level was decreased in KO mice after AOM treatment?

Good question. We do not have a clear answer but there are at least two possibilities. It is likely that AOM and/or its metabolites may be toxic to the microbiota, leading to decreased pncA-mediated deamidation of NAM to NA. It is also possible that AOM-induced epithelial damage reduced the absorption of NA into gut epithelium.

7. Figure 4C:
a. Why was the group highlighted in the black box below are not discussed? This group appears to include genes that are highly expressed in both WT and KO at 12 hours but downregulated in WT at 48 hours.

The reason why we did analyze this cluster of genes (less than one hundred) is because their difference between KO and WT at the time 0 was not strong, although as the reviewer pointed out here, this group includes genes that are highly expressed in both WT and KO at 12 hours but downregulated in WT at 48 hours. Pathway enrichment analysis revealed that they are enriched in genes involved in maintenance of cellular structure, protein quality control, and metabolism. We included this information into Figure 4F, cluster 4.

4F

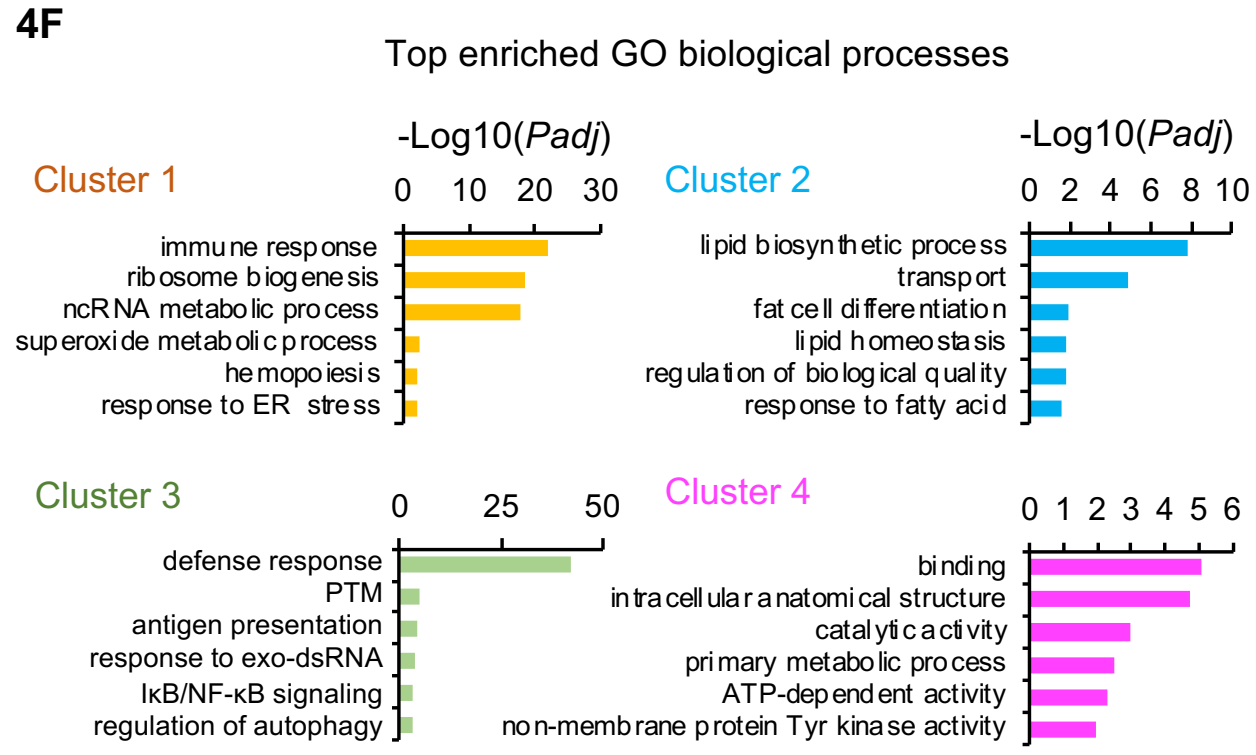

b. It would also be interesting to compare genes within the group across different time points (e.g., WT0 vs. WT12 vs. WT48) and then examine which pathways are altered in the KO

Per the suggestion, we analyzed AOM-induced DEGs between different time points (0 vs 12 vs 48) in WT and NAPRT KO mice respectively, then clustered them using pheatmap and cutree. The heatmap of the z-scores of these AOM-induced DEGs is shown in Figure S5B and the top enriched GO pathways in different clusters are shown in Figure S5C. In WT colons, AOM injection suppressed the expression of genes associated with nutrient metabolism and membrane trafficking (Figure S5B and S5C, clusters 1 and 2) while inducing those involved in immune response, ROS production, cell migration, DNA and RNA metabolism, and stress response (Figure S5B and S5C, clusters 3-6). These transcriptomic changes were more pronounced in NAPRT KO colons compared to WT (Figure S5B and S5C).

8. Figure 5H: The author write in line 330-333 that NAMPT, NAPRT and NADSYN1 are differentially altered in IBD. However, figure 5H clearly shown only NAMPT are differentially altered.

We apologize for the confusing sentence. Because the expression of *NAPRT* and *NADSYN1* remained unchanged, while the expression of *NAMPT* was induced in IBD, what we intended to say is that "the expression of *NAPRT* and *NADSYN1* and that of *NAMPT* were differentially altered in IBD". In other words, the comparison is between the lack of changes in the expression of the two deamidated enzymes in IBD versus induction of *NAMPT*.

To avoid the confusion, we removed this sentence entirely.

This results actually contradict their conclusion that "NAPRT deficiency is associate with increased sensitivity to IBD". Better explanation and support are needed.

We agree with the reviewer that we did not find decrease of NAPRT in human IBD samples and thus we changed the title of this Results section to "NAPRT deficiency is associated with increased sensitivity to DSS-induced colitis"

a. The author may also be interested in checking the NAPRT expression in colon instead of intestine (https://doi.org/10.1038/s41586-019-0992-y).

Thanks again for the suggestion. We analyzed the scRNA-seq dataset from the suggested reference. However, due to low quality of this dataset, we were not able to detect the expression of NAPRT in any cell types.

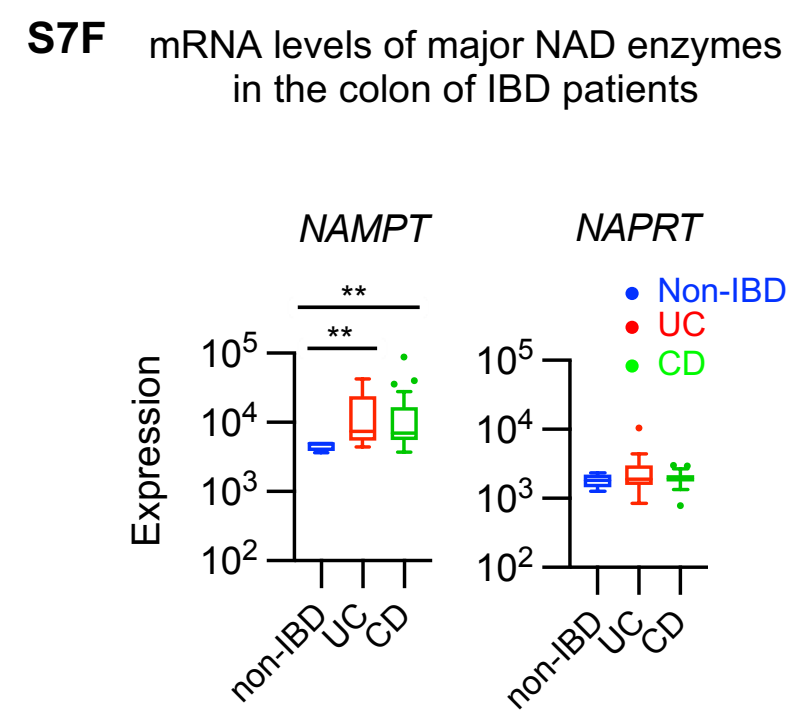

To address the reviewer's question, we reanalyzed the dataset we used in the original manuscript and sorted out the data from the colon. The new results are now shown in new Figure S7F. Although there are only 5 non-IBD colon samples, the results are consistent with those from all samples-- the expression of *NAMPT* is induced in both UC and CD colons, while the expression of *NAPRT* is not altered.

9. Figure 6I: The authors state in lines 387-388 that "TCGA datasets showed that the mRNA levels of NAPRT are reduced in a significant proportion of tumors," which is inaccurate. Figure 6I shows that, in COAD, only 40% of patients show a reduction in NAPRT, while another 40% actually show an increase. This discrepancy is even more pronounced in the LUAD and LUSC datasets, where a larger proportion of patients exhibit increased NAPRT expression. This contradicts the authors' claim that reduced deamidated NAD biosynthesis is associated with tumorigenesis in both mice and humans (lines 394-395). A better explanation and additional support for this discrepancy are needed.

As the reviewer correctly pointed out, only a fraction of tumors exhibited decreased NAPRT expression. This is precisely why we used the phrase "a significant proportion of tumors" rather than "a larger proportion" or "most tumors." It is indeed true that a notable subset of tumors shows elevated NAPRT expression compared to normal tissues. As discussed in the manuscript, this observation has been reported previously and, together with data showing reduced xenograft growth following NAPRT knockdown [5, 6], supports a tumor-promoting role for NAPRT in some cancers.

The novelty of our study lies in focusing on a different subset of patients, specifically, the ~40% of COAD cases in which tumors show lower NAPRT expression than corresponding normal tissues. Our findings that NAPRT knockout increases tumor incidence in mice are consistent with reduced survival in this patient subgroup. We do not see a contradiction in these results. Instead, they support the idea that NAPRT may have tumor-promoting or tumor-suppressing effects depending on cancer stage or molecular context.

We are in fact grateful to the reviewer for the suggestion of performing a subgroup analysis in colorectal cancer. This analysis revealed subtype-specific patterns of NAMPT and NAPRT expression, including a general inverse correlation between these two NAD biosynthetic enzymes (see point #2, Figure 8C and 8D).

We agree with the reviewer that the statement in lines 394-395 is confusing. We therefore deleted this statement in the revision.

10. The figure legends could be improved. Ex: it is unclear what is M+2, M+3, and M+4 in figure 3A and what is 0.5,1,1.5, and 2 in figure 5A means.

We apologize for the unclear figure legends. In Figure 3A, M+2, M+3, and M+4 are heavy isotope-labeled metabolites. For example, M+2 represents the metabolite that is two mass units heavier than the regular metabolite (M+0). In Figure 5A, 0.5, 1, 1.5, and 2 are scores of the rectal bleeding evaluated based on this reference [23], a higher score means a more severe bleeding. We included more details about the bleeding scores in the Methods section.

References:

1. Shats, I. *et al.* Bacteria Boost Mammalian Host NAD Metabolism by Engaging the Deamidated Biosynthesis Pathway. *Cell Metab* **31**, 564-579 e567 (2020).
2. Chellappa, K. *et al.* NAD precursors cycle between host tissues and the gut microbiome. *Cell Metab* **34**, 1947-1959 e1945 (2022).
3. Longarini, E.J. *et al.* Modular antibodies reveal DNA damage-induced mono-ADP-ribosylation as a second wave of PARP1 signaling. *Mol Cell* **83**, 1743-1760 e1711 (2023).
4. Liu, L. *et al.* Quantitative Analysis of NAD Synthesis-Breakdown Fluxes. *Cell Metabolism* **27**, 1067-1080.e1065 (2018).
5. Chowdhry, S. *et al.* NAD metabolic dependency in cancer is shaped by gene amplification and enhancer remodelling. *Nature* **569**, 570-575 (2019).
6. Piacente, F. *et al.* Nicotinic Acid Phosphoribosyltransferase Regulates Cancer Cell Metabolism, Susceptibility to NAMPT Inhibitors, and DNA Repair. *Cancer Res* **77**, 3857-3869 (2017).
7. Yu, S.W. *et al.* Mediation of poly(ADP-ribose) polymerase-1-dependent cell death by apoptosis-inducing factor. *Science* **297**, 259-263 (2002).
8. Zong, W.X., Ditsworth, D., Bauer, D.E., Wang, Z.Q. & Thompson, C.B. Alkylating DNA damage stimulates a regulated form of necrotic cell death. *Genes Dev* **18**, 1272-1282 (2004).
9. Bai, P. *et al.* PARP-1 inhibition increases mitochondrial metabolism through SIRT1 activation. *Cell Metab* **13**, 461-468 (2011).
10. Rank, L. *et al.* Analyzing structure-function relationships of artificial and cancer-associated PARP1 variants by reconstituting TALEN-generated HeLa PARP1 knock-out cells. *Nucleic Acids Res* **44**, 10386-10405 (2016).
11. Cohen, M.S. Interplay between compartmentalized NAD(+) synthesis and consumption: a focus on the PARP family. *Genes Dev* **34**, 254-262 (2020).
12. Ruszkiewicz, J.A., Burkle, A. & Mangerich, A. Fueling genome maintenance: On the versatile roles of NAD(+) in preserving DNA integrity. *J Biol Chem* **298**, 102037 (2022).

13. Tummala, K.S. *et al.* Inhibition of de novo NAD(+) synthesis by oncogenic URI causes liver tumorigenesis through DNA damage. *Cancer Cell* **26**, 826-839 (2014).
14. Gomes, A.L. *et al.* Metabolic Inflammation-Associated IL-17A Causes Non-alcoholic Steatohepatitis and Hepatocellular Carcinoma. *Cancer Cell* **30**, 161-175 (2016).
15. Cantó, C., Menzies, K.J. & Auwerx, J. NAD+ Metabolism and the Control of Energy Homeostasis: A Balancing Act between Mitochondria and the Nucleus. *Cell Metabolism* **22**, 31-53 (2015).
16. Alves-Fernandes, D.K. & Jasiulionis, M.G. The Role of SIRT1 on DNA Damage Response and Epigenetic Alterations in Cancer. *Int J Mol Sci* **20** (2019).
17. Hakem, R. DNA-damage repair; the good, the bad, and the ugly. *EMBO J* **27**, 589-605 (2008).
18. Kim, L.J. *et al.* Host-microbiome interactions in nicotinamide mononucleotide (NMN) deamidation. *FEBS Lett* **597**, 2196-2220 (2023).
19. Rechsteiner, M., Hillyard, D. & Olivera, B.M. Turnover at nicotinamide adenine dinucleotide in cultures of human cells. *J Cell Physiol* **88**, 207-217 (1976).
20. Dietrich, L.S. & Muniz, O. Inhibition of nicotinamide phosphoribosyltransferase by pyridine nucleotides. *Biochemistry* **11**, 1691-1695 (1972).
21. Peek, C.B. *et al.* Circadian clock NAD+ cycle drives mitochondrial oxidative metabolism in mice. *Science* **342**, 1243417 (2013).
22. Dall, M. *et al.* Mitochondrial function in liver cells is resistant to perturbations in NAD(+) salvage capacity. *J Biol Chem* **294**, 13304-13326 (2019).
23. Chassaing, B., Aitken, J.D., Malleshappa, M. & Vijay-Kumar, M. Dextran sulfate sodium (DSS)-induced colitis in mice. *Curr Protoc Immunol* **104**, 15 25 11-15 25 14 (2014).
24. Starr, P. Oral Nicotinamide Prevents Common Skin Cancers in High-Risk Patients, Reduces Costs. *Am Health Drug Benefits* **8**, 13-14 (2015).
25. Lee, R. *et al.* Synthetic Essentiality of Tryptophan 2,3-Dioxygenase 2 in APC-Mutated Colorectal Cancer. *Cancer Discov* **12**, 1702-1717 (2022).
26. Guinney, J. *et al.* The consensus molecular subtypes of colorectal cancer. *Nat Med* **21**, 1350-1356 (2015).

REVIEWER COMMENTS

Reviewer #1 (Remarks to the Author):

This manuscript is a revised version of a paper I previously reviewed. In this revision, several issues have been addressed and improved. However, I believe there remain critical concerns, particularly regarding aspects of systemic nicotinic acid (NA) metabolism, that require further revision.

Major points

1. In my previous review, I pointed out that there is a Nat Commun paper (Nomura M, PMID: 38092728) demonstrating that NA is converted to NAR in a NAPRT-dependent manner. During this revision period, an additional study reported NA-to-NAR conversion in mice (Song WS, Cell Metab '25, PMID: 40315855). Thus, two independent groups have now shown that NA can be converted to NAR. Nevertheless, the present manuscript does not mention or consider this conversion pathway at all. I believe this omission constitutes a significant limitation.

To answer the reviewer's question about whether NA or NAR is the main circulating deamidated NAD precursor beyond the intestinal epithelium during the first revision, we provided evidence that NA is the main source for deamidated NAD biosynthesis in various tested mouse tissues. However, we did not realize that the reviewer would also like to see our measurement on the conversion of NA to NAR. We apologize for the oversight.

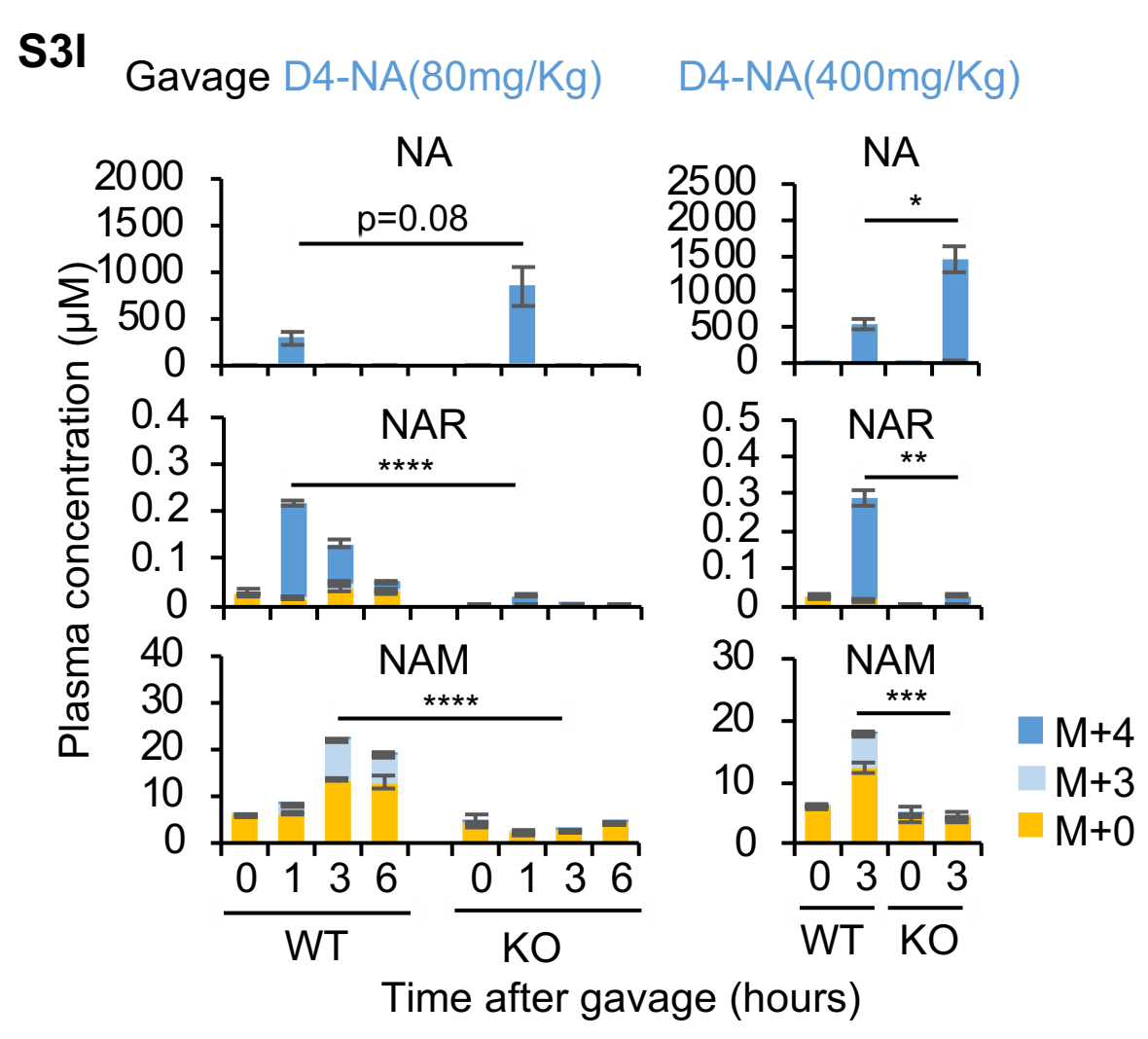

To address this concern as well as Point #3, we orally gavaged D4-labeled NA into WT and NAPRT KO mice, then followed the kinetics of NA, NAR, and NAM in systemic plasma. As shown in the new Figure S3I, the baseline concentration of NA (M+0) in plasma of mice under the regular chow diet, which was reported to be around 1 µM by Chellappa et al. [1], was below the detection limit of our platform (1-2 µM). One hour after oral gavage of D4-NA at 80 mg/Kg, plasma NA levels rose to 278±73 µM in WT and 824±217 µM in KO mice, indicating reduced NA utilization in NAPRT KO mice. NA levels dropped below the detection limit by three hours after gavage (Figure S3I, NA).

Supporting the reviewer's note that NA can be converted into NAR in an NAPRT-dependent manner [2], we observed a significant increase in plasma NAR levels (from 0.028± 0.007 µM to 0.202± 0.007 µM) in WT mice one hour post-gavage, and this increase was almost completely abolished in KO mice (Figure S3I, NAR). Additionally, D4-NA gavage elevated plasma levels of both M+0 and M+3 NAM, peaking between 1- 3 hours in WT but not in KO mice (Figure S3I,

NAM), suggesting increased NAD consumption following NA-induced NAD boosting in WT mice.

Because the peak NAR concentration we observed in WT mice (~0.2 µM) is much lower than levels (1-2 µM) reported by recent studies [2, 3], we repeated the experiment using 400 mg/Kg D4-NA as in Song et al. [3]. Even then, plasma NAR levels remained below 0.3 µM three hours after gavage, in contrast to 540 µM of NA (Figure S3I, right panels).

2. The authors state that the NA concentration used in the in vitro experiments (100 uM) is comparable to the peak serum concentration following NAD booster administration, as reported in the previous study (Chellappa K, Cell Metab '22, PMID: 36476934). While this is factually correct, the study also reported that steady-state levels of NA (in the absence of NR administration) are very low. Omitting this point could lead readers to overestimate physiological NA concentrations.

The reviewer is correct that the baseline concentration of NA in plasma of mice under the regular chow diet without supplementation was reported to be around 1 µM by Chellappa et al. [1]. We agree with the reviewer that even the reduced concentration of 20 µM NA, which we used in the first revision to address this criticism, is still significantly higher than 1 µM. In response to this point, we used 1 µM NA in the new NAPRT add-back MMS experiment requested in point 5.

2E

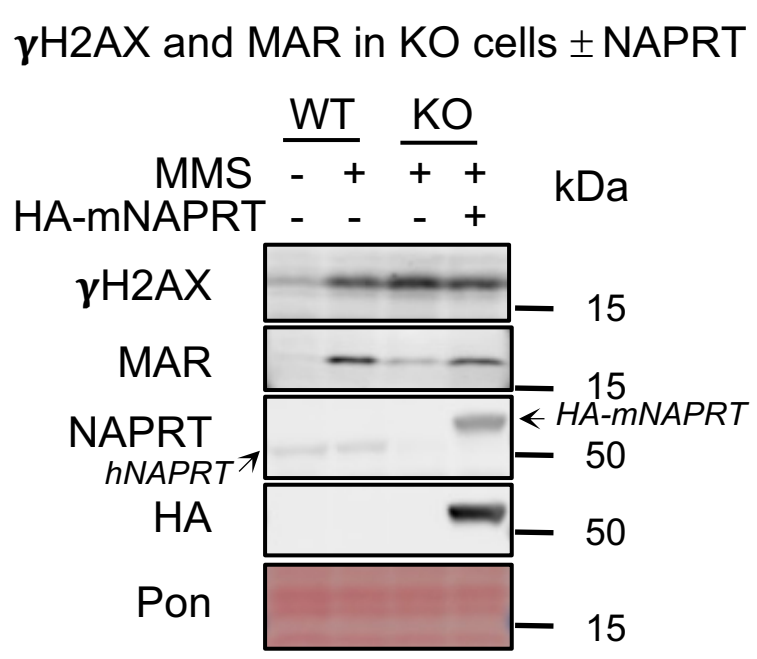

As shown in new Figure 2E, the higher DNA damage in NAPRT KO cells was also observed with this physiologically relevant NA concentration. Stable re-expression of HA-mNAPRT rescued this defect, confirming the genuine role of NAPRT in DNA repair. Our results further suggest that NAPRT-mediated NAD synthesis may be important for maintenance of homeostatic DNA repair.

3. To allow for a more accurate and reliable interpretation of the metabolic data presented in this study, I strongly recommend that the authors include, at a minimum, plasma or serum (not whole blood) concentrations of NAD-related metabolites in WT and Naprt-KO mice, both at both at baseline (prior to NA administration) and at multiple time points following NA administration. Blood sampling should preferably be performed from the systemic circulation, rather than the portal vein, to better reflect whole-body metabolism. In this context, blood collection from the right ventricle is considered the most appropriate.

We performed the experiment requested by the reviewer and the new data are included in new Figure S3I (please see our response to Point #1).

Minor points.

4. In Figures 1 and 2, the in vitro experiments were conducted using only one colorectal cancer

cell line, CRC119. The authors should be more cautious in generalizing the results to normal "gut epithelial cells" as a whole.

In addition to CRC119 cancer line data, the conclusion about the increased sensitivity of NAPRT KO to DNA damage is based on increased gH2Ax staining of normal gut epithelial cells before and after in-vivo AOM treatment of mice (Figure 4D). However, as requested by the reviewer, since our mechanistic studies were performed in CRC119 cells and presented on a separate figure than the in vivo data, we changed "NAPRT-deficient gut epithelial cells" to "NAPRT-deficient cells" when describing the results on Figure 2.

In addition, more information about CRC119 should be provided in the Methods section; at present, even the species is not indicated.

We did mention in the main text (page 7) that CRC119 is a human colon cancer cell line. We provided a reference to the paper describing the derivation of this cell line [4] and added this information also in the method section in this revision.

5. In Figure 2D, the results of the add-back experiment are not clear. Such experiments should be performed using a stable expression system, rather than transient transfection.

Per the reviewer's request, we stably expressed HA-mNAPRT in KO cells and performed MMS experiment using a more physiological 1 μM NA concentration in response to Point #2. The new data are shown in new Figure 2E (please see our response to Point #2).

Reviewer #2 (Remarks to the Author):

At the outset, I was indeed very positive about this manuscript, under the condition that my initial comments were thoroughly addressed. While the paper remains a very well-written and insightful contribution, providing important advances on the role of the Preiss–Handler pathway in NAD biosynthesis, particularly the function of NAPRT in DNA repair and tumorigenesis, it is concerning that some of my key points appear to have been overlooked.

Several other key issues outlined below remain unaddressed. I strongly recommend that these be carefully revised and clarified before the manuscript can be considered suitable for publication. If adequately addressed, this work could indeed represent a valuable contribution to the field.

Point 1

In particular, as highlighted in my Major Concern, the pioneering study by Tummala et al. (2014) on the role of NAD^+ in tumorigenesis, along with the more recent and controversial work by Lee et al. (2022), should be not only cited—which the authors have done—but also properly discussed and integrated into the conceptual framework of the manuscript. These citations currently lack critical engagement.

I respectfully disagree with the response provided to Comment 1. The authors argue that their study is mechanistically distinct from the work of Tummala et al. and Gomes et al., both of

which demonstrated that NAD^+ depletion leads to DNA damage, likely through PARP inactivation or reduced dNTP synthesis. While the authors emphasize that their study centers on NAPRT and its role in DNA repair, they also conclude that NAPRT knockout cells, which have compromised NAD^+ levels, exhibit defective DNA repair and accumulate DNA damage upon MMS treatment.

In my view, the underlying message of these studies remains fundamentally aligned. Therefore, a proper and critical discussion of these prior findings is necessary. Without it, the current manuscript falls short in demonstrating sufficient novelty.

Mechanistic insights into how NAPRT deficiency leads to DNA damage remain insufficiently resolved. Although the authors now demonstrate reduced PARylation and MARylation in NAPRT KO cells, this evidence essentially confirms the role of PARP in their phenotype, thereby aligning their study more closely with previously published findings. Similarly, while SIRT1's role remains unclear in their system, and dNTP synthesis appears largely unaffected. As established in seminal studies such as Tummala et al. (2014) and Gomes et al. (2016), NAD^+ depletion can lead to DNA damage through several converging pathways, including PARP inactivation, Sirtuin inhibition, and reduced dNTP synthesis, all of which are potentially relevant in this context. These studies should be properly cited and integrated into the manuscript's conceptual framework.

Therefore, I respectfully maintain that the manuscript would benefit from a more rigorous and integrative discussion acknowledging how their findings intersect with existing knowledge and litteratire. The notion that NAPRT-derived NAD^+ is essential for DNA repair in gut epithelial cells is interesting; however, the message conveyed converges with previously published observations about NAD^+ depletion and genome instability. This convergence must be transparently acknowledged and discussed to better contextualize the contribution of this work and to address concerns around novelty.

It is unclear why the authors have chosen to omit citation and discussion of previous pioneering studies that have significantly shaped the understanding of NAD^+ metabolism and its role in DNA damage and tumorigenesis.

We apologize that we did not provide a rigorous and integrative discussion to acknowledge previous pioneering studies on NAD, DNA damage, and tumorigenesis in our first revision. In this revision, we added the following paragraph in the Discussion section:

"*The importance of NAD in supporting DNA repair and maintaining genome stability has been well-established in the literature (reviewed in [5, 6]). Consistently, pathological conditions that lead to NAD depletion, such as aging, high-fat diet feeding, and oncogenic gene expression, have been reported to increase DNA damage and promote tumorigenesis through various NAD-dependent mechanisms, including PARP inactivation, sirtuin inhibition, and reduced dNTP synthesis. For instance, a seminal study by Tummala et al. showed that in hepatocytes, suppression of the de novo NAD biosynthesis pathway by expression of an oncogene unconventional prefoldin RPB5 interactor (URI) leads to reduced cellular NAD levels and increased DNA damage at early stages of tumorigenesis possibly by inhibition of PARP [7].*

Restoration of hepatic NAD pool with NR supplementation prevents DNA damage and tumor formation [7]. In a following-up study, Gomes et al. reported that URI-induced inhibition of de novo NAD biosynthesis and subsequent DNA damage also trigger hepatic T cell infiltration and increase IL-17A production, which further promotes non-alcoholic steatohepatitis and hepatocellular carcinoma [8]. These defects could also be abolished by dietary supplementation of NR [8]. In the present study, by utilizing a genetic NAPRT KO model that enabled us to investigate the role of the Preiss-Handler arm of the deamidated NAD biosynthesis during tumor initiation, we demonstrated that in the gut epithelium, deficiency of this pathway impairs PARP activity and compromises DNA repair, thereby increasing susceptibility to inflammation and tumorigenesis. To our knowledge, this is the first study to identify endogenous NAPRT as a tumor suppressor rather than a tumor promoter."

We hope this addition addressed the reviewer's concern.

Point 2

The authors fairly replied to this point: Orally administered NR and NMN are largely converted by gut microbiota into nicotinic acid (NA), which then enters the Preiss-Handler pathway requiring NAPRT for NAD^+ synthesis. In NAPRT knockout (KO) models, this pathway is disrupted, preventing NAD^+ restoration from NR/NMN, as seen in both in vitro (e.g., CRC119 cells) and in vivo studies. Despite this, NR and NMN remain widely used because, in organisms with functional NAPRT and normal gut microbiota, they effectively boost NAD^+ levels and provide physiological benefits. Thus, the efficacy of NR/NMN depends on the presence of NAPRT and highlights the critical role of the Preiss-Handler pathway in their metabolism.

Yet, dietary levels of tryptophan may be sufficient to compensate for NAPRT deficiency in many tissues. This has not been tested. A rescue experiment can still be important.

We are sorry that we are not sure what type of "rescue" experiment the reviewer is requesting here.

If "a rescue experiment" refers to tryptophan (Trp) supplementation, we are concerned that such an experiment may not yield meaningful outcomes. The conversion of Trp to NAD is only one of several Trp metabolic fates that requires eight metabolic reactions, and the ratio of conversion is low (e.g. ~ 60:1 in humans) [9, 10]. This conversion is expected to be particularly limited in the gut, where QPRT expression is minimal (Figure 1A). Indeed, in a recent report published in *Cell Metabolism,* Song et al. showed that Trp only boosts NAD levels in the liver and kidney [3]. Since our study is focused on the gut, a rescue experiment with Trp supplementation is not feasible.

Alternatively, if the reviewer's intention is to ask us to test whether "dietary Trp compensates for NAPRT deficiency in many tissues", then we could potentially place NAPRT KO mice on a Trp-deficient diet so that any possible compensations from *de novo* NAD synthesis will be unmasked. However, the NAMPT-mediated amidated NAD salvage could very likely compensate loss of both NAPRT deficiency and inhibition of *de novo* pathway, masking any effects from Trp restriction. Moreover, Trp is an essential amino acid with broad roles beyond

NAD synthesis, including protein biosynthesis and generation of multiple bioactive metabolites [11]. It also has extensive interactions with gut microbiota and Trp-deficient diet feeding has been shown to induce intestinal inflammation and many other adverse effects [12]. These NAD-independent effects would significantly complicate interpretation of any observed phenotypes.

Given these factors, we are unsure how Trp rescue and/or restriction experiments would yield interpretable or meaningful results.

Point 3

This is justified by the authors stating, "We agree that conditional NAPRT KO in different cell types would be more informative. However, as the reviewer noted here, such studies are beyond the scope of the current revision."

This is inaccurate; the reviewer never claimed it was beyond the scope but explicitly requested this experiment. Nevertheless, the xenograft studies presented do not fully support the paper's conclusions, and conditional KO mouse models would be important to address this gap.

We apologize that we are not able to generate a conditional KO mouse model within a reasonable time-frame to meet this request.

In terms of the xenograft results, we respectfully disagree with the reviewer's interpretation. As previously noted (in the first revision), using NAPRT KO mouse model, we found that impaired deamidated NAD biosynthesis in NAPRT deficient cells leads to diminished PARP activity and compromised DNA repair in response to carcinogen-induced DNA damage. This defect in DNA repair increases the rate of oncogenic mutations, thereby promoting cancer initiation. Established cancer cell lines used in the xenograft experiment, on the other hand, are not suitable to study oncogenic mutation-associated cancer initiation because they have already accumulated all the mutations needed for malignant growth. Further inhibition of DNA repair by deletion of NAPRT in these cell lines is not expected to further enhance their growth rate. It can instead decrease the growth rate of xenograft tumors due to excessive DNA damage and reduced metabolic fitness. This outcome was supported by results from our xenograft experiment as well as by previous published findings [13].

We would like to again emphasize that our study is the first to demonstrate that NAPRT can function as a tumor suppressor rather than a tumor promoter. This novel finding was made possible by our use of a newly developed genetic NAPRT KO model, which enabled us to investigate the effects of NAPRT deficiency during tumor initiation, in contrast to prior studies using xenograft models based on established cancer cell lines. In fact, our observations that NAPRT deficiency increased tumor initiation in mice but reduced tumor growth in established cancer cell lines reveal an intriguing stage-specific role of NAD in cancer: suppressing tumor initiation at early stages while supporting tumor growth and progression at later stages. We believe this finding offers valuable guidance for the development of future NAD-based strategies in cancer prevention and treatment.

Other important points

I regret to note that γH2AX, the standard marker for DNA damage, has not been successfully demonstrated in this study, despite its widespread and well-established use in numerous publications. It is unclear why evidence of DNA damage is not provided or visible here.

We are perplexed by the comment here because we believe that our data in Figures 2B, 2C, 2D, new 2E, 4D, S2E, and S2F clearly showed increased γH2AX in NAPRT KO cells and mice following genotoxic stress. Particularly, our new NAPRT add-back experiment (Figure 2E) further validates the authentic role of NAPRT in DNA repair at physiological levels of NA (1 μM) as demonstrated by higher γH2AX in KO cells and rescue of this defect by NAPRT add-back.

Reviewer #3 (Remarks to the Author):

The authors have addressed all my concern

References

1. Chellappa, K. *et al.* NAD precursors cycle between host tissues and the gut microbiome. *Cell Metab* **34**, 1947–1959 e1945 (2022).
2. Nomura, M. *et al.* Niacin restriction with NAMPT-inhibition is synthetic lethal to neuroendocrine carcinoma. *Nat Commun* **14**, 8095 (2023).
3. Song, W.S. *et al.* Nicotinic acid riboside maintains NAD(+) homeostasis and ameliorates aging-associated NAD(+) decline. *Cell Metab* **37**, 1616–1618 (2025).
4. Tung, K.L. *et al.* Integrated chromatin and transcriptomic profiling of patient-derived colon cancer organoids identifies personalized drug targets to overcome oxaliplatin resistance. *Genes Dis* **8**, 203–214 (2021).
5. Cohen, M.S. Interplay between compartmentalized NAD(+) synthesis and consumption: a focus on the PARP family. *Genes Dev* **34**, 254–262 (2020).
6. Ruszkiewicz, J.A., Burkle, A. & Mangerich, A. Fueling genome maintenance: On the versatile roles of NAD(+) in preserving DNA integrity. *J Biol Chem* **298**, 102037 (2022).
7. Tummala, K.S. *et al.* Inhibition of de novo NAD(+) synthesis by oncogenic URI causes liver tumorigenesis through DNA damage. *Cancer Cell* **26**, 826–839 (2014).
8. Gomes, A.L. *et al.* Metabolic Inflammation-Associated IL-17A Causes Non-alcoholic Steatohepatitis and Hepatocellular Carcinoma. *Cancer Cell* **30**, 161–175 (2016).
9. Fukuwatari, T. & Shibata, K. Nutritional aspect of tryptophan metabolism. *Int J Tryptophan Res* **6**, 3–8 (2013).
10. Connell, N.J., Houtkooper, R.H. & Schrauwen, P. NAD(+) metabolism as a target for metabolic health: have we found the silver bullet? *Diabetologia* **62**, 888–899 (2019).
11. Xue, C. *et al.* Tryptophan metabolism in health and disease. *Cell Metab* **35**, 1304–1326 (2023).

12. Yusufu, I. *et al.* A Tryptophan-Deficient Diet Induces Gut Microbiota Dysbiosis and Increases Systemic Inflammation in Aged Mice. *Int J Mol Sci* **22** (2021).
13. Piacente, F. *et al.* Nicotinic Acid Phosphoribosyltransferase Regulates Cancer Cell Metabolism, Susceptibility to NAMPT Inhibitors, and DNA Repair. *Cancer Res* **77**, 3857–3869 (2017).

REVIEWER COMMENTS

Reviewer #1 (Remarks to the Author)

I appreciate that in this revision the authors have addressed the concerns I previously raised. However, there remains one important issue that should be resolved before publication. I apologize for not noticing it earlier, but I believe it is critical for the validity of the study.

This concerns the mutant mouse line referred to as "Naprt-KO" in the manuscript. Typically, genome-editing–based gene disruption involves a frameshift mutation, which leads to nonsense-mediated mRNA decay and thus a marked reduction in transcript levels. However, as the authors noted, the allele in this mouse carries an in-frame deletion of seven amino acid residues (Fig. S3A), which would not normally cause a severe decrease in mRNA levels as reported in Fig. S3B.

Although several functional studies in the paper suggest a loss of Naprt activity, it remains essential to confirm that Naprt protein is indeed absent in this mutant line. This could be demonstrated, for example, by Western blotting of tissue lysates. Such verification would clarify whether the allele truly represents a null mutation.

I am aware that the deletion may have disrupted an exon splicing enhancer (ESE), potentially leading to cryptic splicing; however, this possibility further underscores the need to verify the actual loss of Naprt protein.

We are sorry that our data in Figure S3B confused the reviewer. We have noted in the figure legend that this qPCR analysis was done using **WT specific primers,** which were designed to specifically amplify the full length *Naprt* mRNA containing the 21-bp fragment that is present in the WT but not in the mutant allele. The aim of this verification qPCR is to confirm the deletion of the 21-bp in the Naprt KO cells and tissues. The sequences of these WT specific primers have already been included in Table S7 (mNAPRT genotyping-F and mNAPRT genotyping-R). We have another set of qPCR primers of mNAPRT, which are specific for Exon 1 of *Naprt* gene, for regular qPCR analysis. Their sequences are also included in Table S7 (mNAPRT-F and mNAPRT-R).

To present our figures and results more clearly to the reader, we have made the following modifications in this revision:

(1) We labeled the positions of the aforementioned four primers in Figure S3A (new Supplementary Figure 3a) in green.

(2) We conducted additional qPCR analysis in primary cells and tissues from WT and NAPRT KO mice using both primer sets to show that while the genotyping (GT) primer set failed to amplify the mutant *Naprt* mRNA in cells/tissues from NAPRT KO mice, the regular primer set revealed comparable levels of *Naprt* mRNA between WT and KO mice (new Supplementary Figure 3b).

(3) We performed immunoblotting analysis of NAPRT in the liver and colon per the reviewer's request (new Supplementary Figure 3c). Our results showed that in both liver and colon, the deletion of the 7 amino acids in NAPRT KO mice resulted in a slight down shift of NAPRT protein bands. Moreover, this deletion was coupled with significant protein degradation, particularly in the liver.

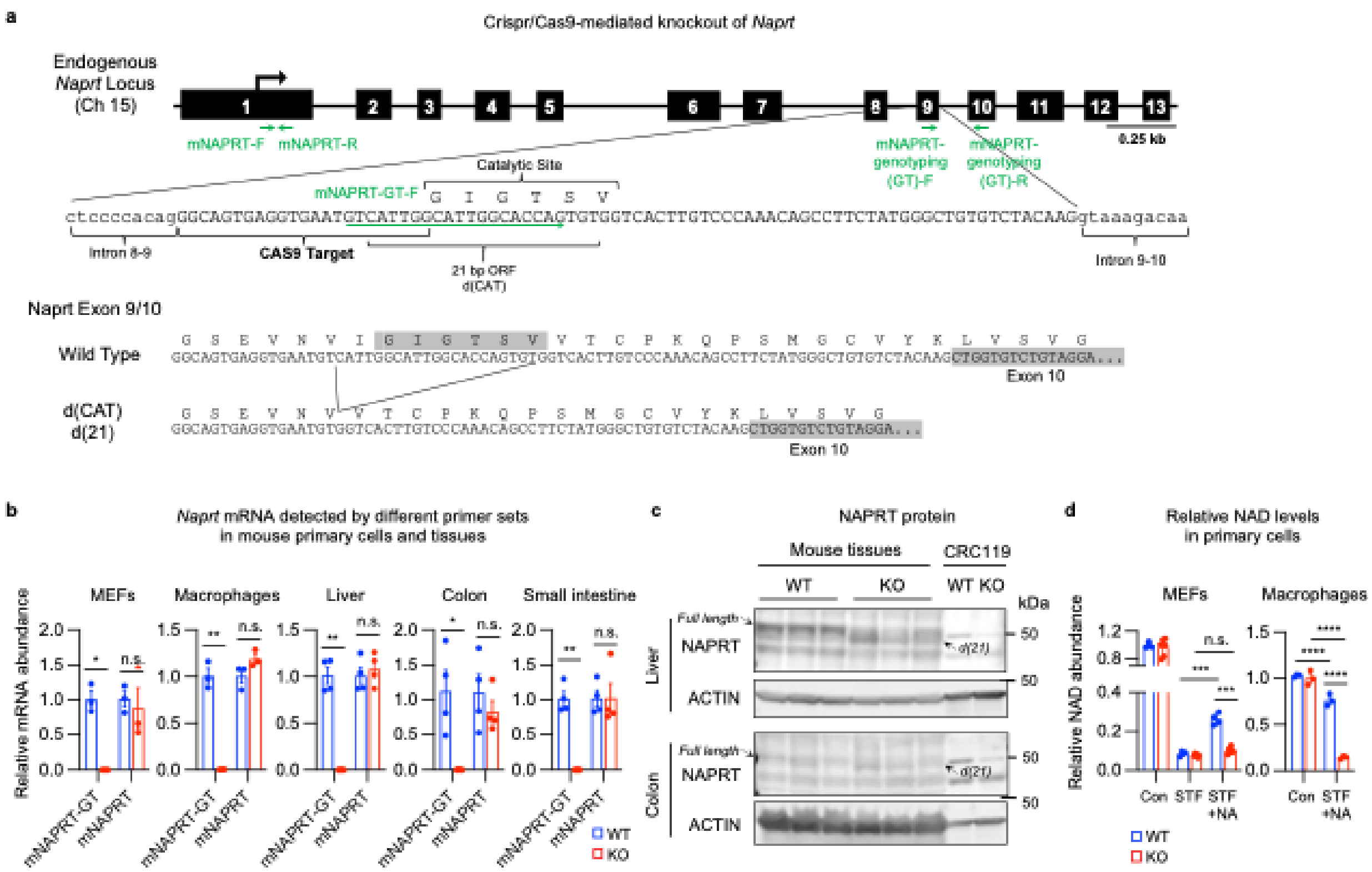

Reviewer #2 (Remarks to the Author)

Although I was initially very critical of certain experiments and aspects of the discussion, the authors have addressed all my concerns. I am satisfied with their responses and the revisions provided.

One minor point is that the authors should discuss the limitation of the study arising from the use of xenograft models, rather than the use of conditional NAPRT knockout in different cell types.

With this minor suggestion, I recommend that this paper be accepted for publication.

We are glad to know that we have addressed all concerns of the reviewer.

For the minor point of the reviewer, we modified the Discussion section to include the following language:

The first paragraph on Page 28: "*In fact, our observations that NAPRT deficiency increased tumor initiation in mice together with previous reports on the anti-tumor growth effects of NAPRT inhibition in established cancer cell lines and xenografts reveal an intriguing stage-specific role of NAD in cancer: suppressing tumor initiation at early stages while supporting tumor growth and progression at later stages. These findings could offer valuable guidance for the development of future NAD-based strategies in cancer prevention and treatment."*

At the end of the first paragraph on Page 29: "*Future studies employing tissue-specific NAPRT knockout models are needed to further elucidate the role of NAPRT in different cell types and at different stages along the carcinogenesis process*."

Hope these are sufficient to address this minor point.
