# Supplementary material for: NAPRT-mediated deamidated NAD biosynthesis enhances colon tissue resiliency and suppresses tumorigenesis
Source: Nat Commun. 2026 Feb 10;17:8651. doi: 10.1038/s41467-026-68998-w (PMC13490428; doi:10.1038/s41467-026-68998-w)

Supplemental Figures

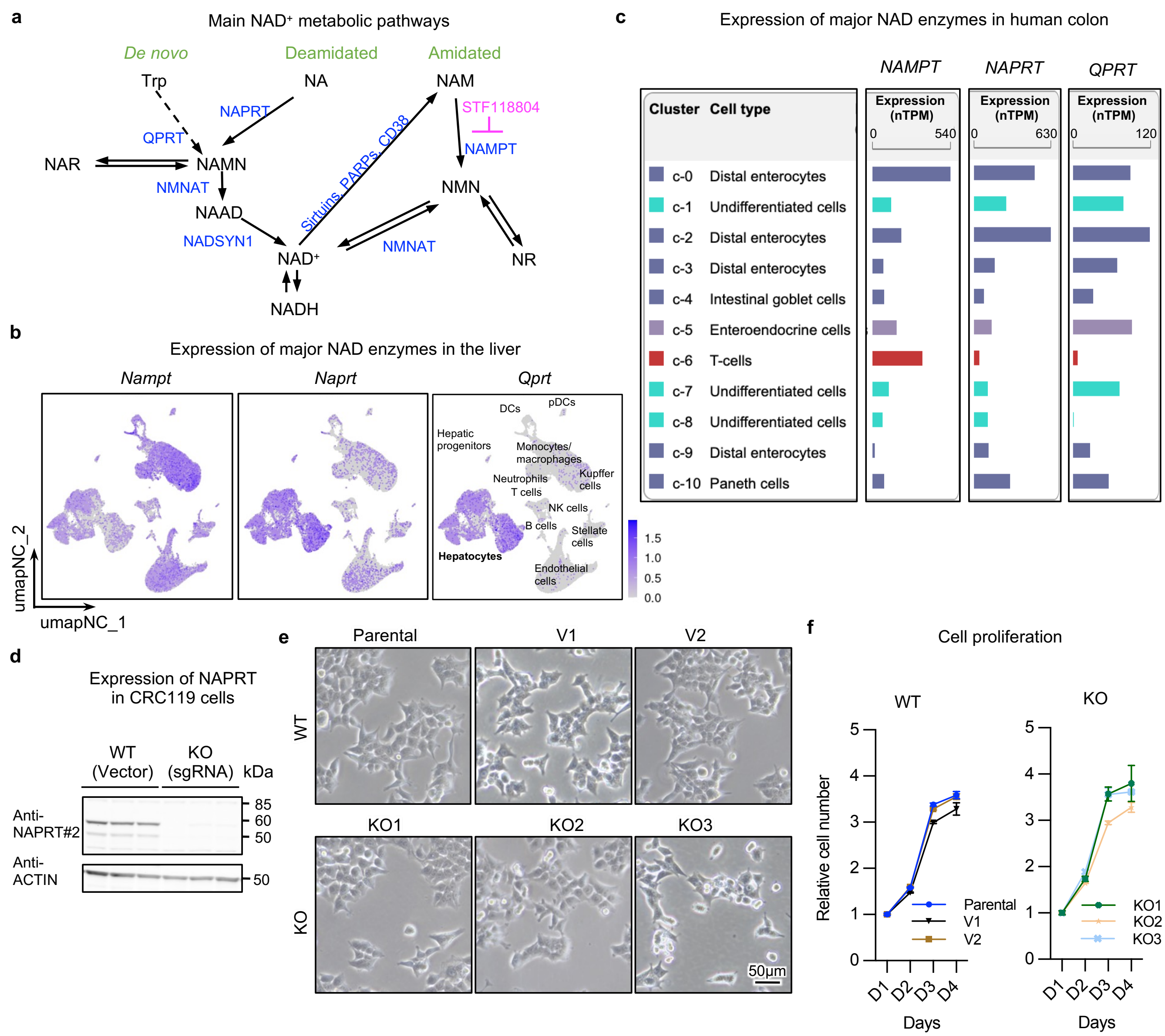

Supplementary Figure 1. NAPRT is enriched in gut epithelial cells
a, Main NAD biosynthesis pathways. Trp, tryptophan; NAR, nicotinic acid riboside; NAMN, nicotinic acid mononucleotide; NAAD, nicotinic acid adenine dinucleotide; NA, nicotinic acid; NAM, nicotinamide; NMN, nicotinamide mononucleotide; NR, nicotinamide riboside; QPRT, quinolinate phosphoribosyltransferase; NAPRT, nicotinic acid phosphoribosyltransferase; NMNAT, nicotinamide mononucleotide adenylyltransferase; NADSYN1, NAD synthetase; NAMPT, nicotinamide phosphoribosyltransferase; PARPs, poly (ADP-ribose) polymerases. **b**, Expression of key enzymes in major NAD biosynthesis pathways in the liver. Feature plots for the expression of *Nampt*, *Naprt*, and *Qprt* in all cells in mouse liver (scRNA-seq dataset was from [1], n=82,168 total single cells). **c**, Expression levels of *NAMPT*, *NAPRT*, and *QPRT* in human colon. Data were obtained from scRNA-seq data from The Human Protein Atlas (www.proteinatlas.org). **d**, Protein levels of NAPRT in WT (Vector control) and NAPRT KO CRC119 cells analyzed by immunoblotting. WT and NAPRT KO CRC119 cells were generated as described in Methods. Three independent clones per genotype are shown. **e-f**, Morphology and proliferation of WT and NAPRT KO CRC119 cells. Source data are provided as a Source Data file.

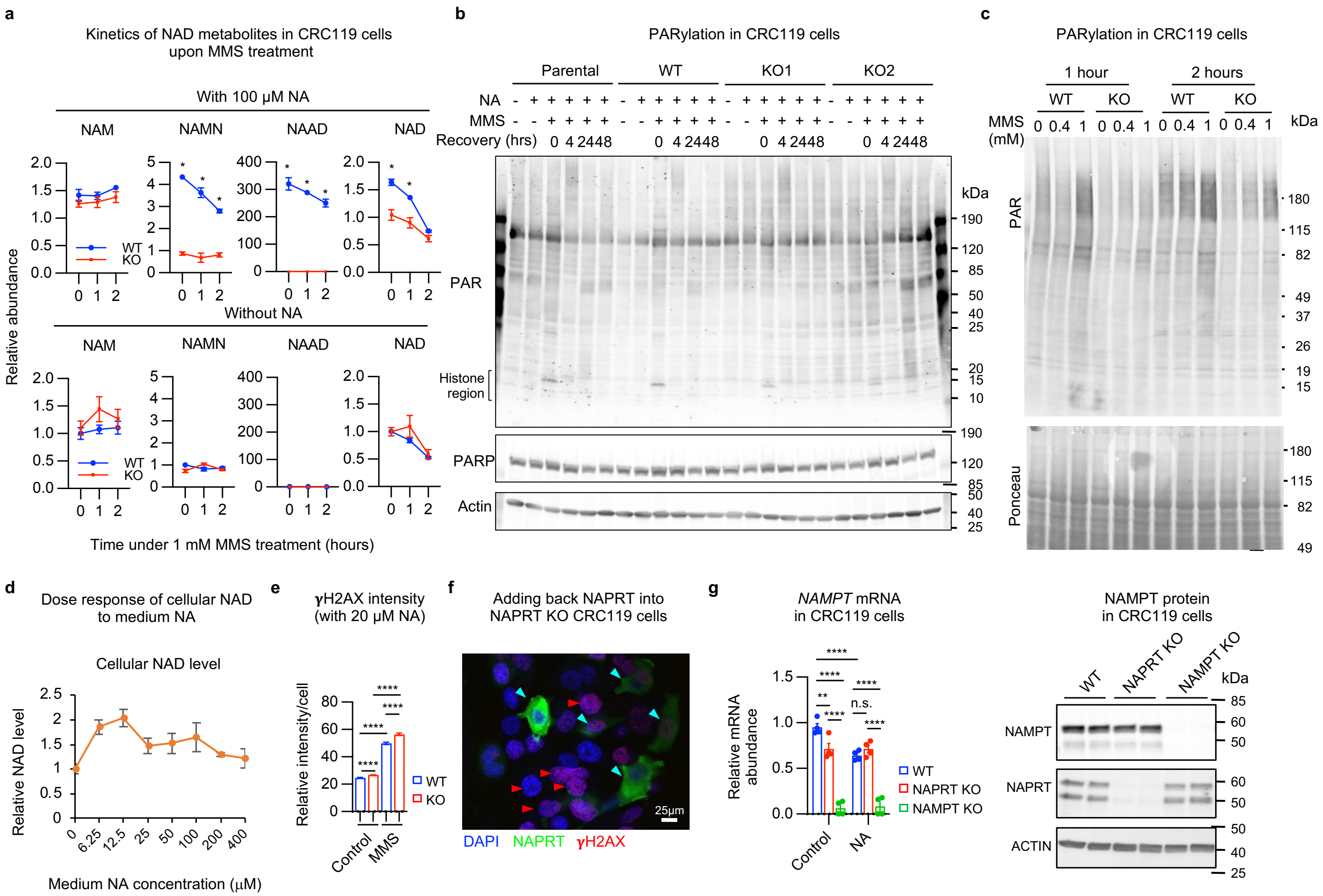

Supplementary Figure 2. NAPRT deficiency sensitizes cultured cells to chemically induced DNA damage

a, DNA damage-induced changes of NAD metabolites in CRC119 cells. WT and NAPRT KO CRC119 cells were cultured in regular RPMI1640 medium with or without 100 μM NA for 48 hours. Cells were then treated with 1 mM MMS for 1 or 2 hours, and intracellular NAD-related metabolites were measured by LC-MS (n=4 biological replicates/group, results were normalized to total protein levels; values are expressed as mean ±SEM; two-tailed Mann-Whitney test). **b-c**, NAPRT KO CRC119 cells have reduced protein PARylation after DNA damage. **b**, WT and NAPRT KO CRC119 cells were cultured with or without 100 μM NA for 48 hours. They were then treated with or without 1 mM MMS for 1 hour, followed by recovery in NA-containing medium without MMS for 0, 4, 24, and 48 hours. Total cell lysates were analyzed using an anti-PAR reagent (Sigma-Aldrich, cat# MABE1016) and anti-PARP1 antibodies. **c**, WT and NAPRT KO CRC119 cells were cultured in growth medium containing 100 μM NA for 48 hours, then treated with indicated concentrations of MMS for 1 or 2 hours. Total cell lysates were analyzed using an anti-PAR antibody (10H). **d**, Dose response of cellular NAD to medium NA levels. CRC119 cells were cultured with indicated concentration of NA for 22 hours in regular RPMI medium. Cellular total NAD levels were measured by the NAD-GLO assay kit (n=2 biological replicates/group, values are expressed as mean ±SD). **e**, NAPRT KO CRC119 cells have increased DNA damage. WT and NAPRT KO CRC119 cells were cultured in growth medium with 20 μM NA for 48 hours, then treated with or without 1 mM MMS for 1 hour. The immunofluorescence staining intensity of γH2AX from at least 2,000 cells/group was quantified (values are expressed as mean ±SEM; two-way ANOVA). **f**, Adding back NAPRT in NAPRT KO CRC119 cells reduces their sensitivity to DNA damage. Cells were cultured in medium containing 20 μM NA, treated with MMS for 1 hour, then co-stained for NAPRT and γH2AX. Red arrowheads, NAPRT negative (Neg) cells; Cyan arrowheads, NAPRT positive (Pos) cells. **g**, mRNA and protein levels of NAMPT in WT (Vector control), NAPRT KO, and NAMPT KO CRC119 cells (n=2 biological replicates x 2 independent clones, values are expressed as mean ±SEM, two-way ANOVA), and protein levels were analyzed by immunoblotting with indicated antibodies. Source data are provided as a Source Data file.

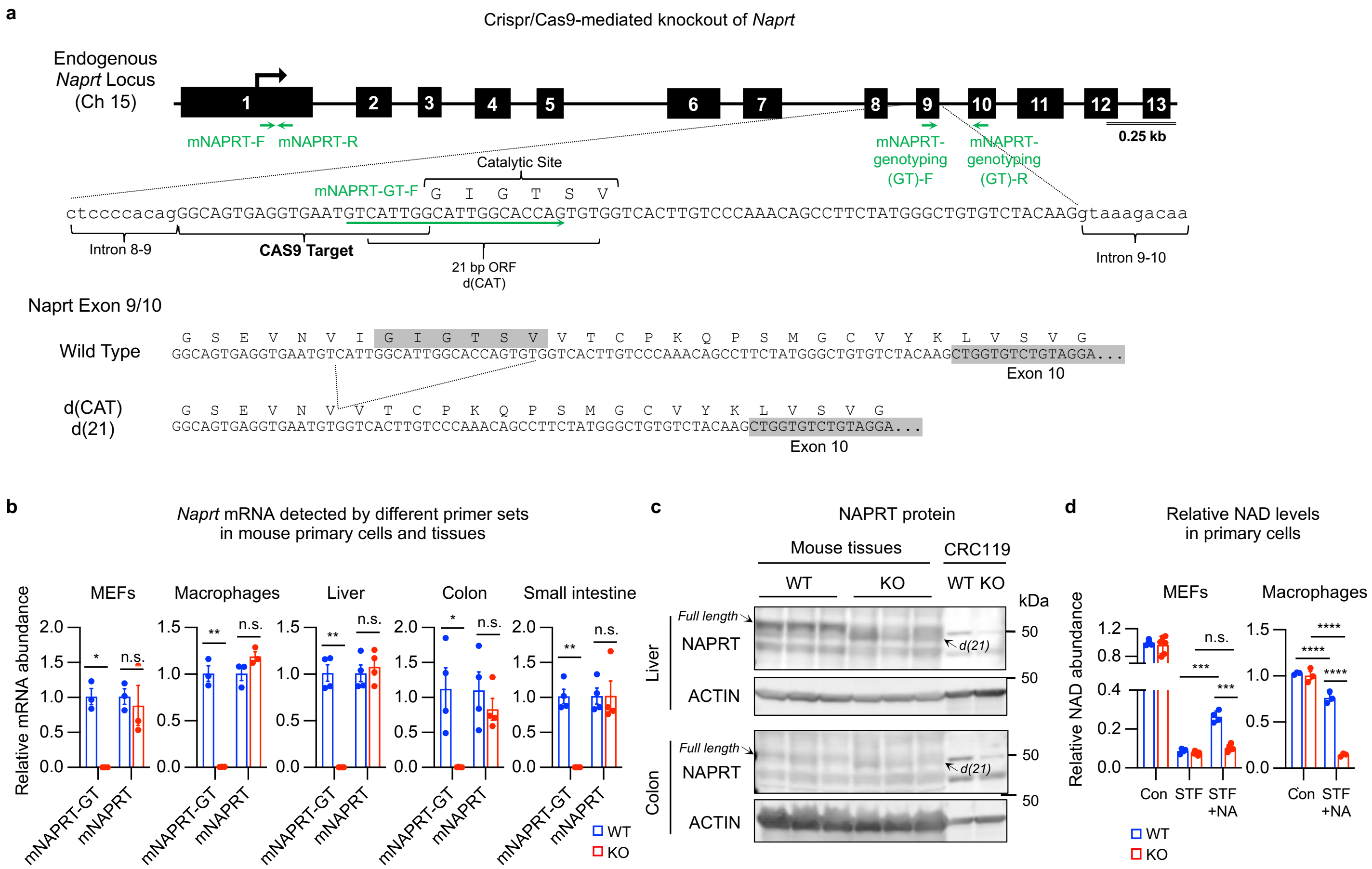

Supplementary Figure 3. Generation of NAPRT KO mice

a, DNA sequence of a mutant NAPRT line d(21) with a 21-bp fragment (7 a.a.) deletion in the catalytic domain of *Naprt* gene. The positions of two qCPR primer sets are shown in green: mNAPRT, for regular qPCR quantification of *Naprt* mRNA; and mNAPRT-genotyping (GF), for verification of the deletion of the targeted 21-bp ORF in the KO allele (WT specific). **b**, qPCR analysis of *Naprt* mRNA abundance in primary mouse cells and tissues using two qPCR primer sets (n=3 pairs of mice for MEFs and macrophages and 4 pairs for other tissues, values are expressed as mean ±SEM, two-tailed unpaired Student's t-test). **c**, Immunoblotting analysis of NAPRT protein in the liver and colon of WT and NAPRT KO mice. Anti-NAPRT#2 was used. **d**, NAPRT KO primary mouse cells are sensitive to STF-induced NAD depletion in the presence of NA. Primary MEFs and primary bone marrow differentiated macrophages were cultured with or without 10 μM NA or 100 nM STF118804 (STF) for 48 hours (MEF, n=4 WT and 6 KO independent embryos; Macrophages, n=3 mice/genotype; values are expressed as mean ±SEM; two-way ANOVA). Source data are provided as a Source Data file.

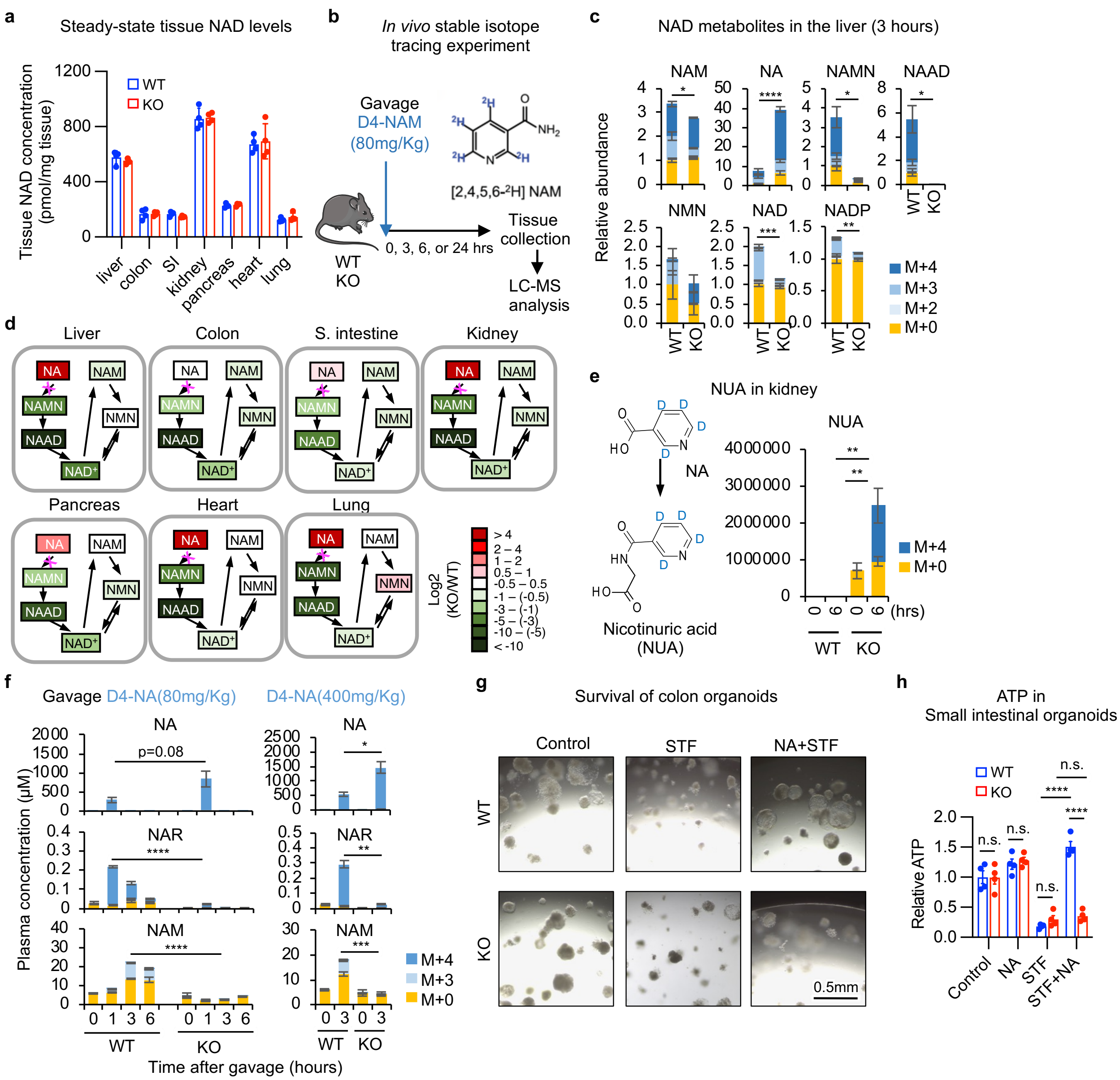

Supplementary Figure 4. Deletion of NAPRT in mice impairs NAD metabolism

a, Basal tissue NAD levels (n=4 mice/group, values are expressed as mean ±SEM). **b**, Schematic of the in vivo stable isotope tracing experiment. Mouse cartoon image was drawn by using pictures from Servier Medical Art (https://smart.servier.com/wp-content/uploads/2016/10/Animals.pptx). Servier Medical Art by Servier is licensed under a Creative Commons Attribution 3.0 Unported License (https://creativecommons.org/licenses/by/3.0/). **c**, NAD metabolites in the liver. WT and KO mice were treated as in (**b**) (n=4 mice/genotype, values expressed as mean ±SEM; two-tailed Mann-Whitney test). **d**, The log ratios of the relative abundance of labeled metabolites in KO vs WT tissues are represented by a color scale (n=4 mice/genotype). **e**, NAPRT KO mice have increased catabolism of NA in the kidney. WT and KO mice were treated as in (**b**) and levels of NA catabolite nicotinuric acid (NUA) in the kidney were measured by LC-MS (n=4, 4, 4, and 5 mice, respectively; raw MS signals of NUA were normalized with tissue weight and expressed as mean±SEM, two-tailed unpaired Student's t-test). **f**, NAPRT deficiency abolishes NAR synthesis after NA gavage. Circulating NAD metabolites from the systemic plasma were measured by LC-MS (n=3-5 mice/group, values are expressed as mean ±SEM; two-tailed unpaired Student's t-test). **g**, Representative brigh field images of WT and KO mouse colon organoids cultured in medium with or without 100 μM NA or 100 nM STF for 48 hours. **h**, Cellular ATP levels of WT and KO small intestinal organoids cultured in medium with or without 100 μM NA or 100 nM STF for 72 hours (n=4 biological replicates/group, values are expressed as mean ±SEM; two-way ANOVA). Source data are provided as a Source Data file.

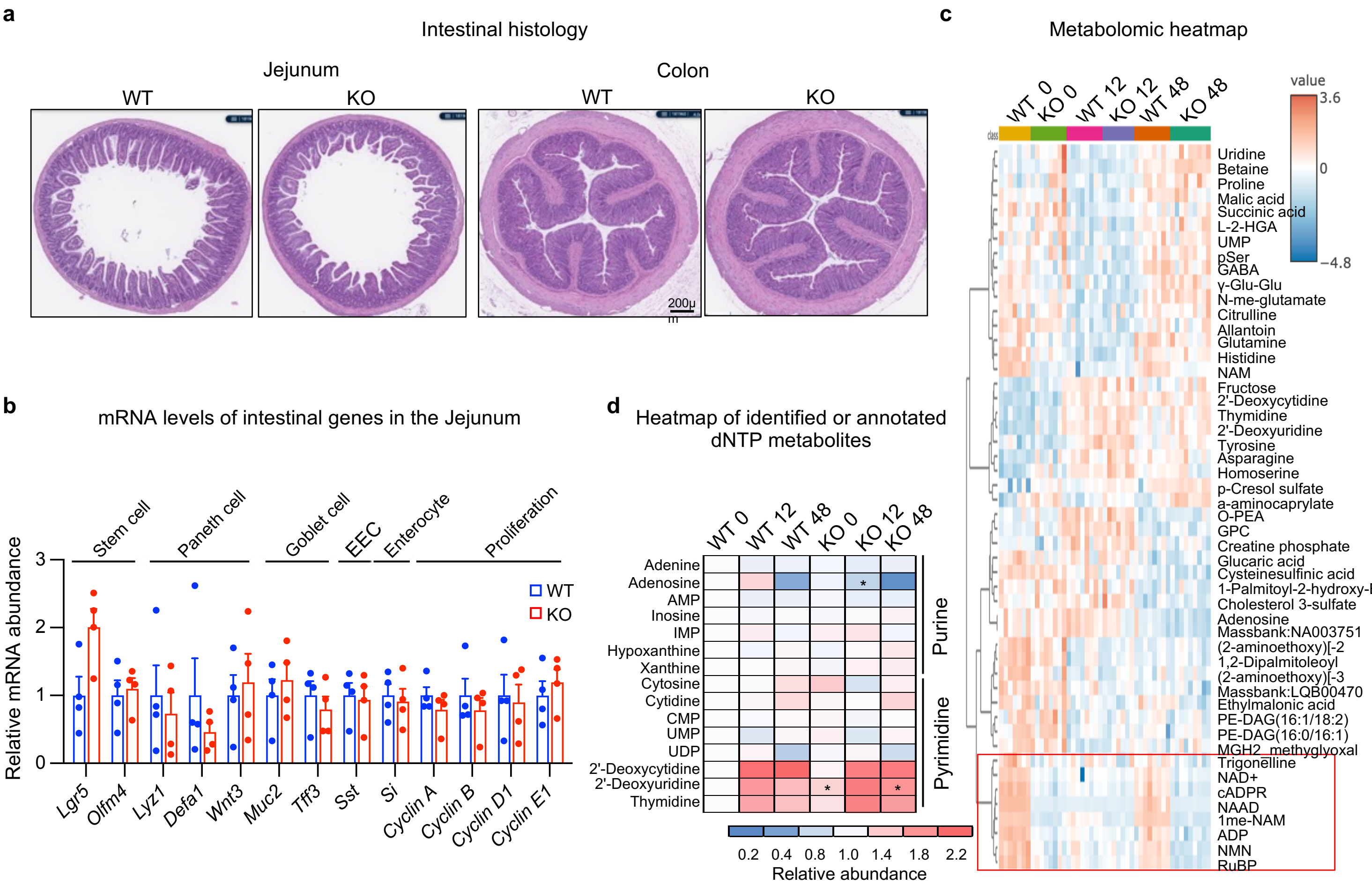

Supplementary Figure 5. NAPRT deficiency sensitizes the intestine to chemically induced DNA damage
a, NAPRT KO mice have normal intestinal histology. **b**, NAPRT KO mice have normal expression of intestinal marker genes in the jejunum (n=4 mice/genotype, values are expressed as mean ±SEM). **c**, The heatmap of the top 50 differential identified metabolites in the colon of WT and NAPRT KO mice after injection of AOM for 0, 12, or 48 hours. Colon tissues from indicated mice were analyzed for their metabolomic changes using LC-MS as described in Methods (n=7, 8, 8, 8, 8, 9 mice, respectively). The red box indicates NAD-related metabolites. **d**, Heatmap of identified or annotated metabolites related to dNTPs (n=7, 8, 8, 8, 8, 9 mice, respectively, values are normalized to WT 0; comparisons were between KO vs WT at the specific time point, two-tailed unpaired Student's t-test). Source data are provided as a Source Data file.

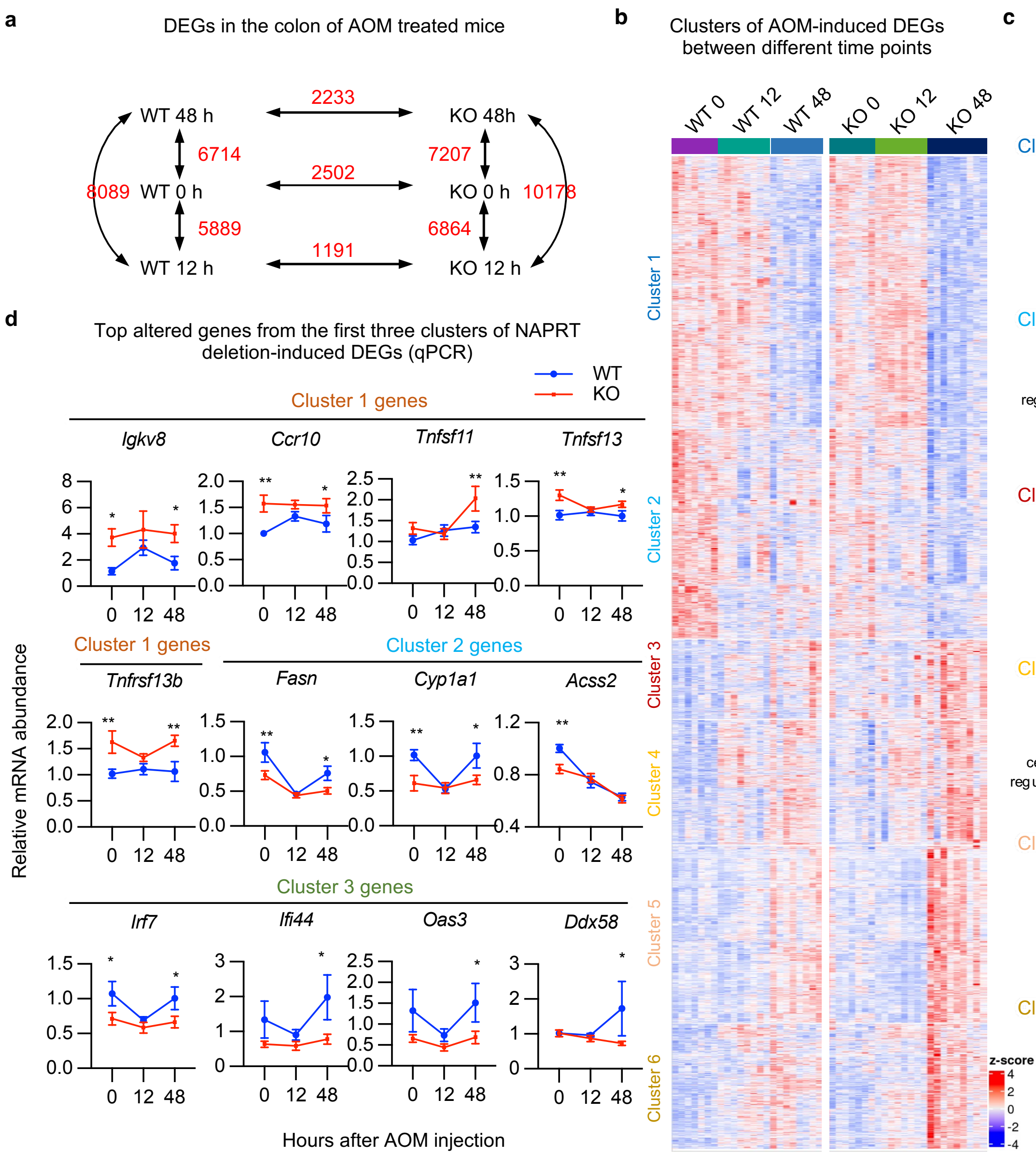

Supplementary Figure 6. Transcriptomic responses of WT and NAPRT KO mice to AOM treatment

a, Numbers of differentially expressed genes (DEGs) between different treatment groups in WT and NAPRT KO mice during AOM treatment. Whole colon tissues from WT and NAPRT KO mice at time 0, 12, and 48 hours after AOM injection were analyzed by RNA-seq (n=7-9 mice/group, DEGs were identified by DESeq2 with p<0.05). **b**-**c**, AOM-induced DEGs between different time points (0 vs 12 vs 48) in WT and NAPRT KO mice respectively were clustered based on their expression patterns using pheatmap and cutree packages. **b**, Heatmap of the z-scores of AOM-induced DEGs. **c**, Top enriched GO pathways in different clusters in (**b**). **d**, Top altered genes from the first three NAPRT deletion-induced DEG clusters (in Figure 4**e**) were analyzed by qPCR (n=7, 8, 8, 8, 8, 9 mice, respectively, two-way ANOVA). Source data are provided as a Source Data file.

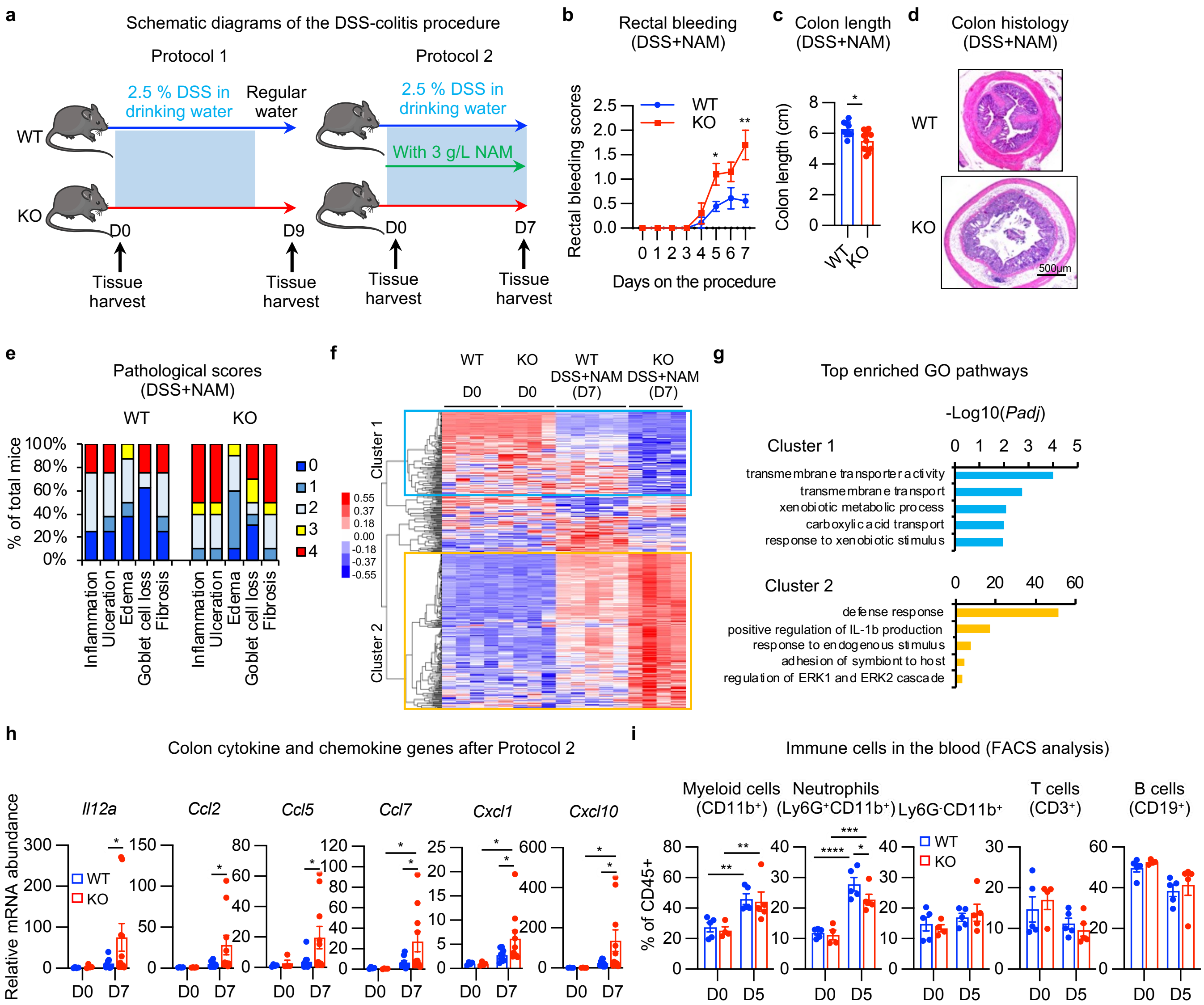

Supplementary Figure 7. NAPRT KO mice are hypersensitive to DSS-induced colitis

a, Schematic diagrams of two DSS-colitis protocols used in this study. Protocol 1, mice were treated with 2.5% DSS in drinking water for 7 days, then recovered with regular water for 2 days. Protocol 2, mice were treated with 2.5% DSS together with 3 g/L NAM in drinking water for 7 days. Mouse cartoon image was drawn by using pictures from Servier Medical Art (https://smart.servier.com/wp-content/uploads/2016/10/Animals.pptx). Servier Medical Art by Servier is licensed under a Creative Commons Attribution 3.0 Unported License (https://creativecommons.org/licenses/by/3.0/). **b**, NAPRT KO mice have increased rectal bleeding during DSS treatment (Protocol 2; n=9 WT and 10 KO mice, D7; values are expressed as mean ±SEM; two-tailed Mann-Whitney test). **c**, NAPRT KO mice have more severe colon shortening after Protocol 2 (n=9 WT and 10 KO mice, D7; values are expressed as mean ±SEM; two-tailed Mann-Whitney test). **d**, Representative H&E colonic images of WT and NAPRT KO mice after Protocol 2. **e**, Pathological scores of colonic tissues after Protocol 2 evaluated by a board-certified veterinary pathologist (n=8 WT and 10 KO mice, D7). **f-g,** NAPRT KO mice have increased induction of genes involved in inflammation and immune function (Cluster 2) and increased suppression of genes involved in various transport functions (Cluster 1) in the colon after Protocol 2. **f**, Heatmap. **g**, Top enriched GO pathways in cluster 1 and cluster 2. **h**, NAPRT KO mice have increased induction of cytokines and chemokines in the colon after Protocol 2 (D0, n=4 WT and n=4 KO; D7, n=9 WT and n=10 KO mice; values are normalized to the untreated WT controls and expressed as mean ±SEM; two-way ANOVA). **i**, Blood immune cell populations analyzed by flow cytometry in WT and NAPRT KO mice after 5-day treatment of DSS (D0, n=4 WT and 5 KO; D5, n=5 WT and 5 KO mice; values are expressed as mean ±SEM; two-way ANOVA). Source data are provided as a Source Data file.

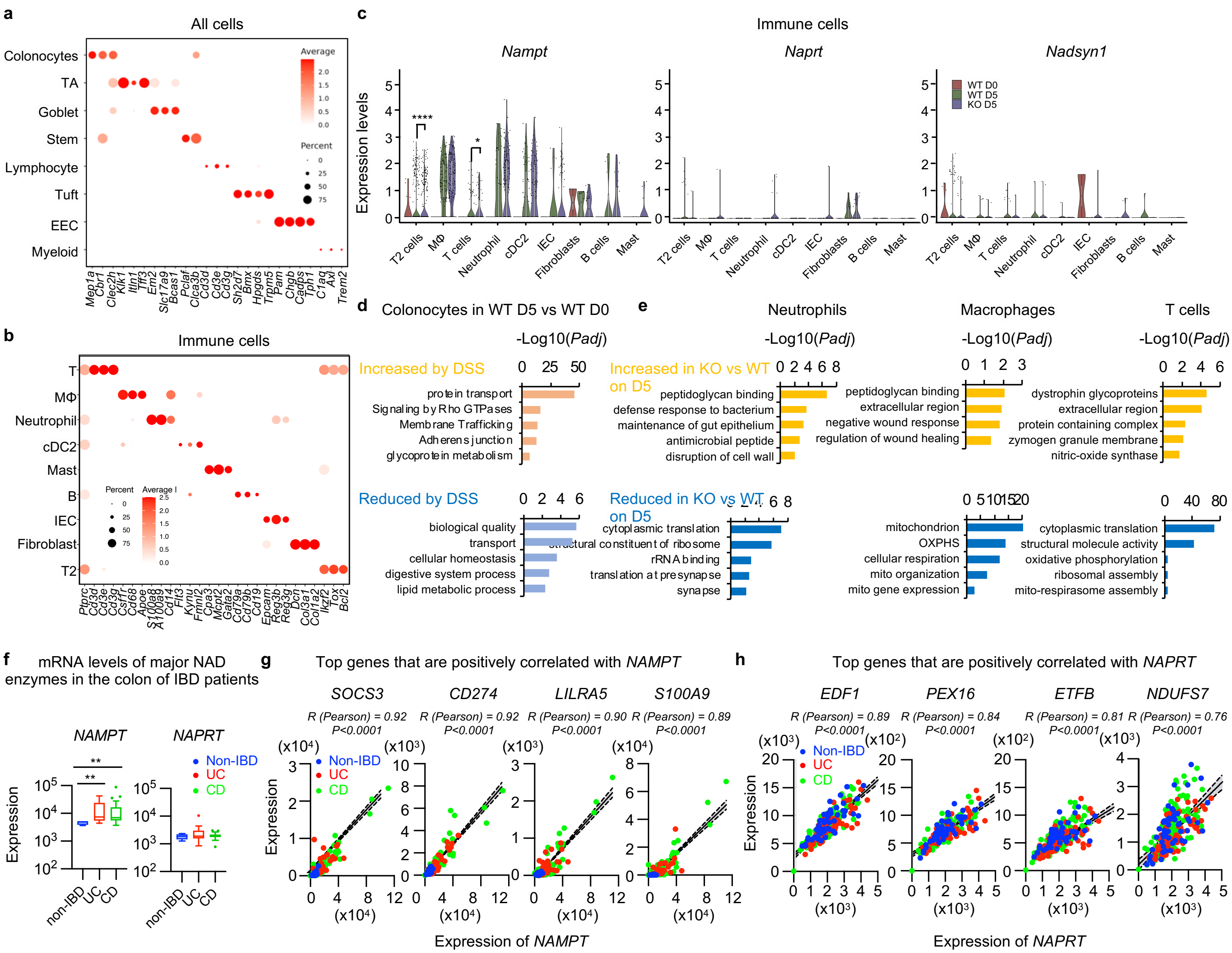

Supplementary Figure 8. NAPRT deficiency is associated with increased anti-microbial defense response and reduced maintenance of tissue integrity in colitis.

a, Dot plot showing the expression of marker genes in different function groups of total live cells isolated from the colons of WT and NAPRT KO mice treated with or without DSS as determined by scRNA-seq (Figure 6A). **b**, Dot plot showing the expression of marker genes in different immune cell sub-groups of total live immunme cells (lymphocytes and myeloid cells in **a**, Figure 6**b**). **c**, *Nampt*, but not *Naprt* or *Nadsyn1*, is significantly induced by DSS treatment in two T cell clusters in the colons (Wilcoxon test). **d**, Top enriched biological processes in genes increased or reduced by DSS in WT colonecytes. **e**, Top enriched biological processes in genes altered by NAPRT deletion in different immune cells at D5 after DSS treatment. **f**, The expression of *NAMPT* but not *NAPRT* or *NADSYN* is significantly elevated in the colon of IBD patients. A subset of colonic samples from the RNA-seq dataset from [2] were analyzed (Kuskal-Wallis test) **g**, The top genes positively correlated with *NAMPT* are induced in IBD patients and involved in proinflammatory response (n=50 non-IBD, 73 UC, and 127 CD patients, from [3]). **h**, The top genes positively correlated with *NAPRT* are important for cell proliferation and metabolism regardless of the patient IBD status (n=50 non-IBD, 73 UC, and 127 CD patients, from [3]). Source data are provided as a Source Data file.

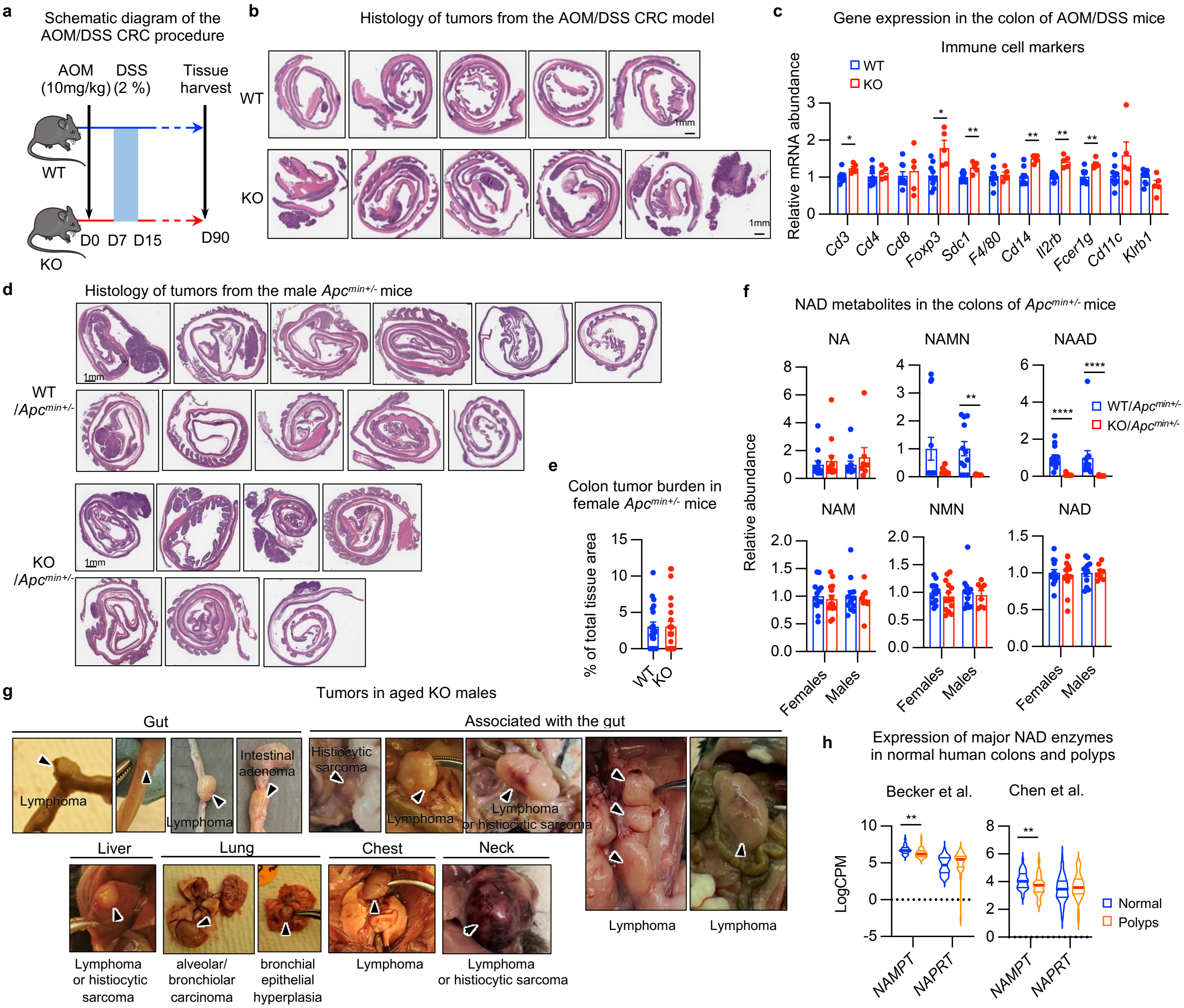

Supplementary Figure 9. NAPRT deficiency increases susceptibility to tumorigenesis

a, Schematic diagram of the AOM/DSS CRC procedure. Mouse cartoon images were drawn by using pictures from Servier Medical Art (https://smart.servier.com/wp-content/uploads/2016/10/Animals.pptx). Servier Medical Art by Servier is licensed under a Creative Commons Attribution 3.0 Unported License (https://creativecommons.org/licenses/by/3.0/). **b**, H&E colonic images of 5 pairs of WT and NAPRT KO female mice at the end of the AOM/DSS CRC procedure used for tumor stage evaluation. **c**, NAPRT KO mice have increased expression of immune cell markers at the end of the AOM/DSS CRC procedure (n=9 WT and 5 KO mice, values are expressed as mean ±SEM; multiple Mann-Whitney tests). **d**, H&E colonic images of WT and NAPRT KO male mice on the *Apc*$^{min+/-}$ background at the age of 16 weeks. **e**, Colon tumor burden in WT and NAPRT KO female mice in the *Apc*$^{min+/-}$ background analyzed at the age of 16 weeks (n=20 WT, 24 KO, values are expressed as mean ±SEM). **f**, NAD metabolites in the colones of WT/*Apc*$^{min+/-}$ and NAPRT KO/*Apc*$^{min+/-}$ mice at the age of 16 weeks (n=13 WT and 14 KO females and 12 WT and 8 KO males; values are expressed as mean ±SEM; two-tailed unpaired Mann-Whitney tests). **g**, Spontaneous tumors observed in various tissues of aged (22-24 months) NAPRT KO male mice. Tumor types were determined by a board-certified pathologist based on H&E slides. **h**, The expression of *NAMPT* and *NAPRT* in polyps vs normal human colon tissues. scRNA-seq and RNA-seq datasets from Becker et al. [4] and Chen et al. [5] were analyzed as described in Methods (n=24 normal and 43 polyps in Becker et al.; n=68 normal and 48 polyps in Chen et al.; two-tailed unpaired Mann-Whitney tests). Source data are provided as a Source Data file.

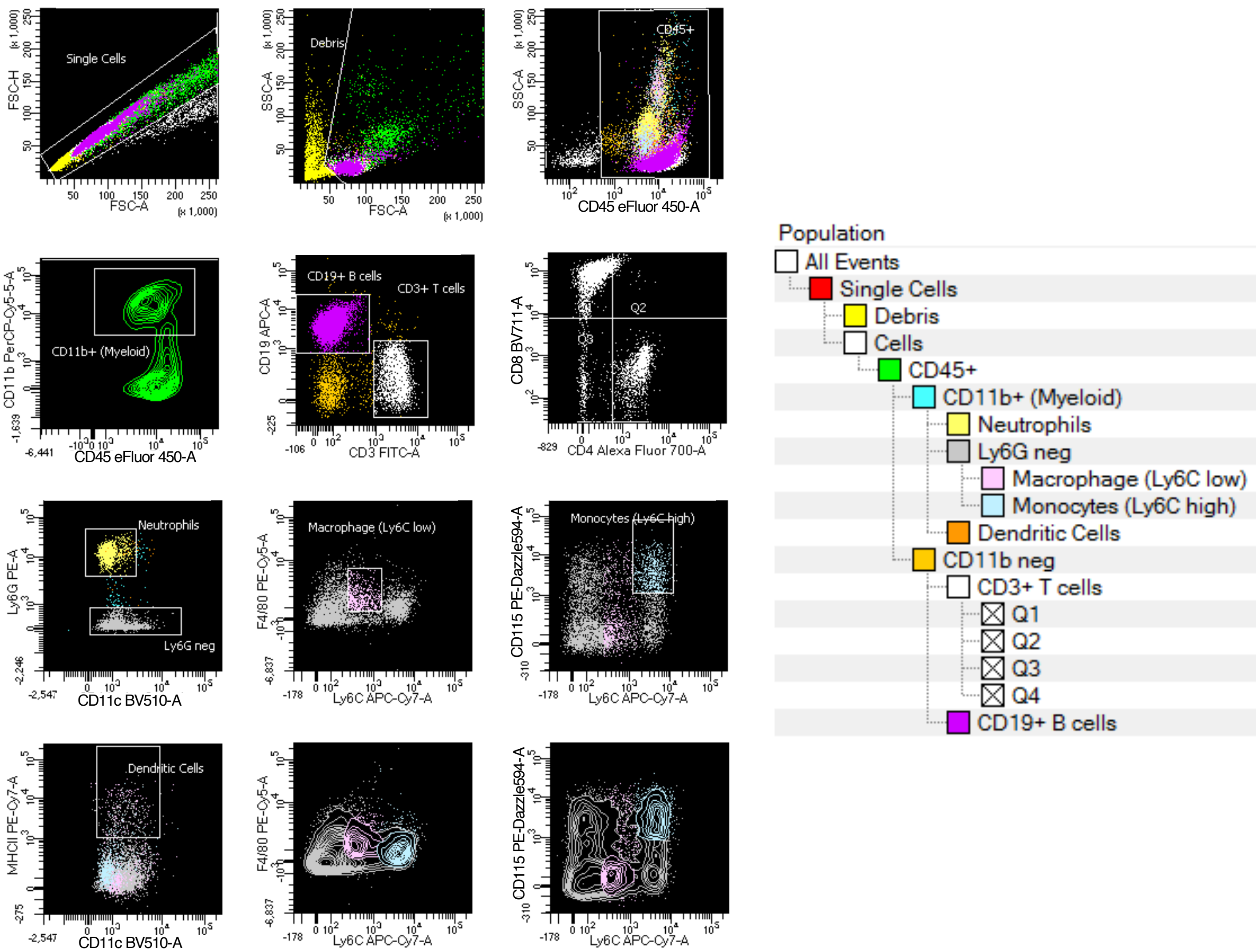

Supplementary Figure 10. FACS gating strategy in this study

References:

1. Su, Q. *et al.* Single-cell RNA transcriptome landscape of hepatocytes and non-parenchymal cells in healthy and NAFLD mouse liver. *iScience* **24**, 103233 (2021).
2. Lloyd-Price, J. *et al.* Multi-omics of the gut microbial ecosystem in inflammatory bowel diseases. *Nature* **569**, 655-662 (2019).
3. Lloyd-Price, J. *et al.* Multi-omics of the gut microbial ecosystem in inflammatory bowel diseases. *Nature* **569**, 655-662 (2019).
4. Becker, W.R. *et al.* Single-cell analyses define a continuum of cell state and composition changes in the malignant transformation of polyps to colorectal cancer. *Nat Genet* **54**, 985-995 (2022).
5. Chen, B. *et al.* Differential pre-malignant programs and microenvironment chart distinct paths to malignancy in human colorectal polyps. *Cell* **184**, 6262-6280 e6226 (2021).

Uncropped immunoblots in Supplementary Figures

Supplementary Figure 1d

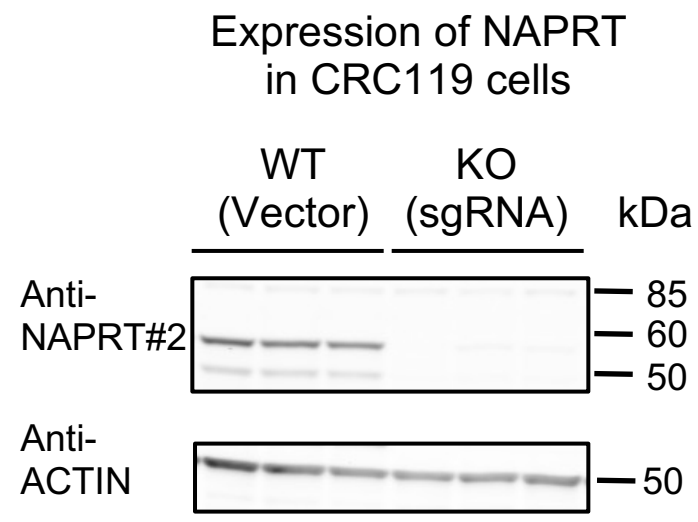

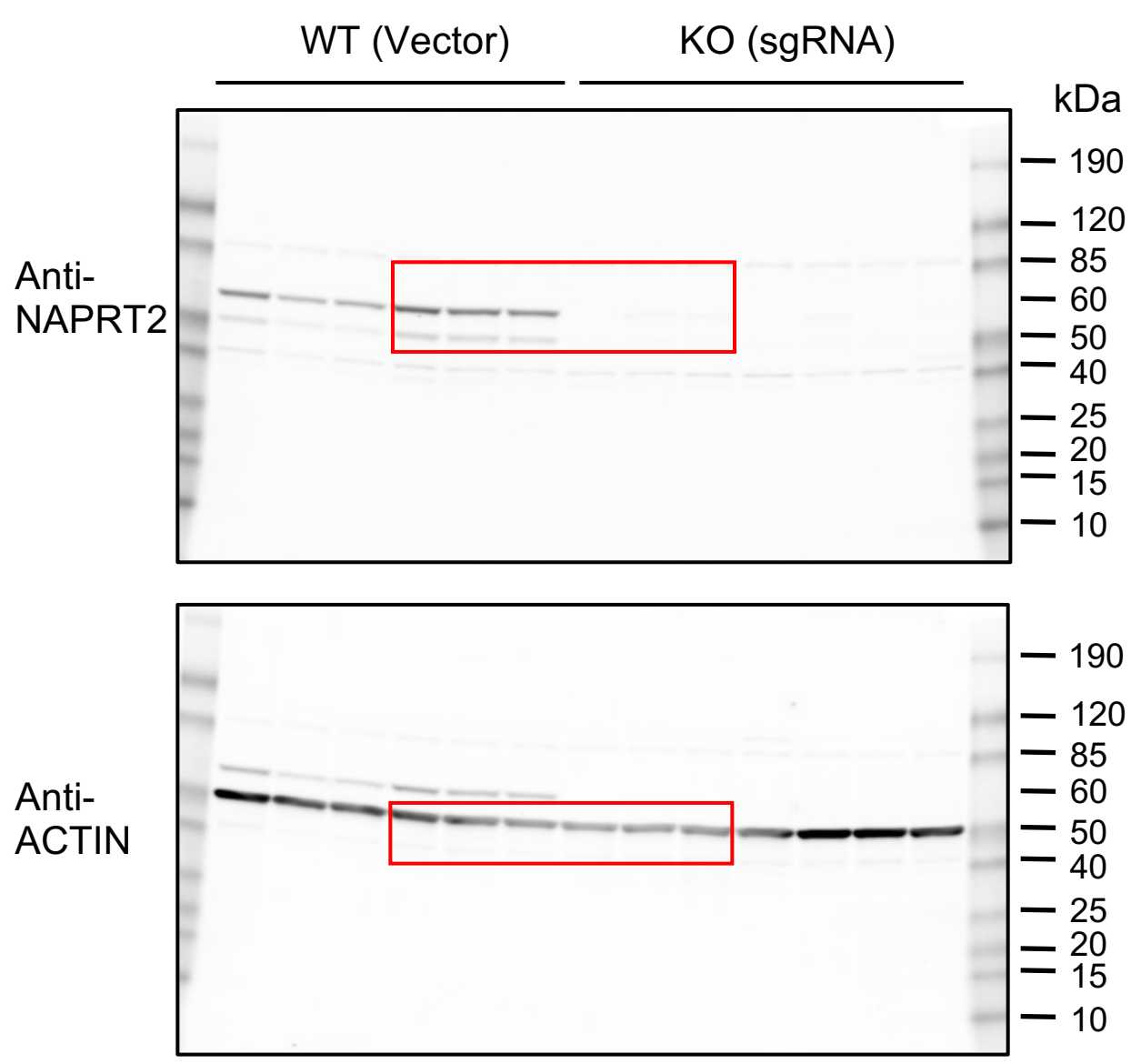

Supplementary Figure 2g

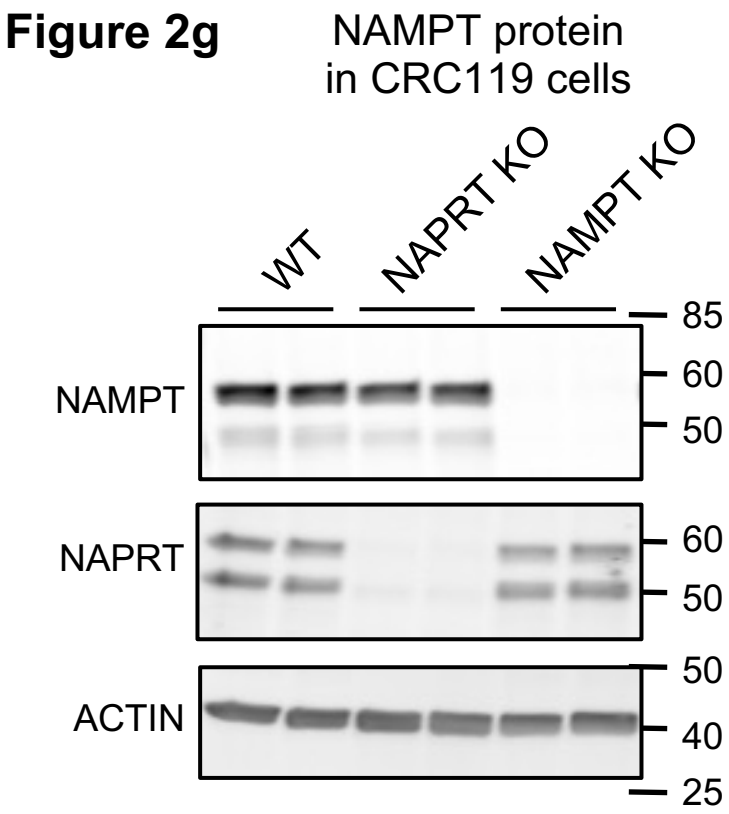

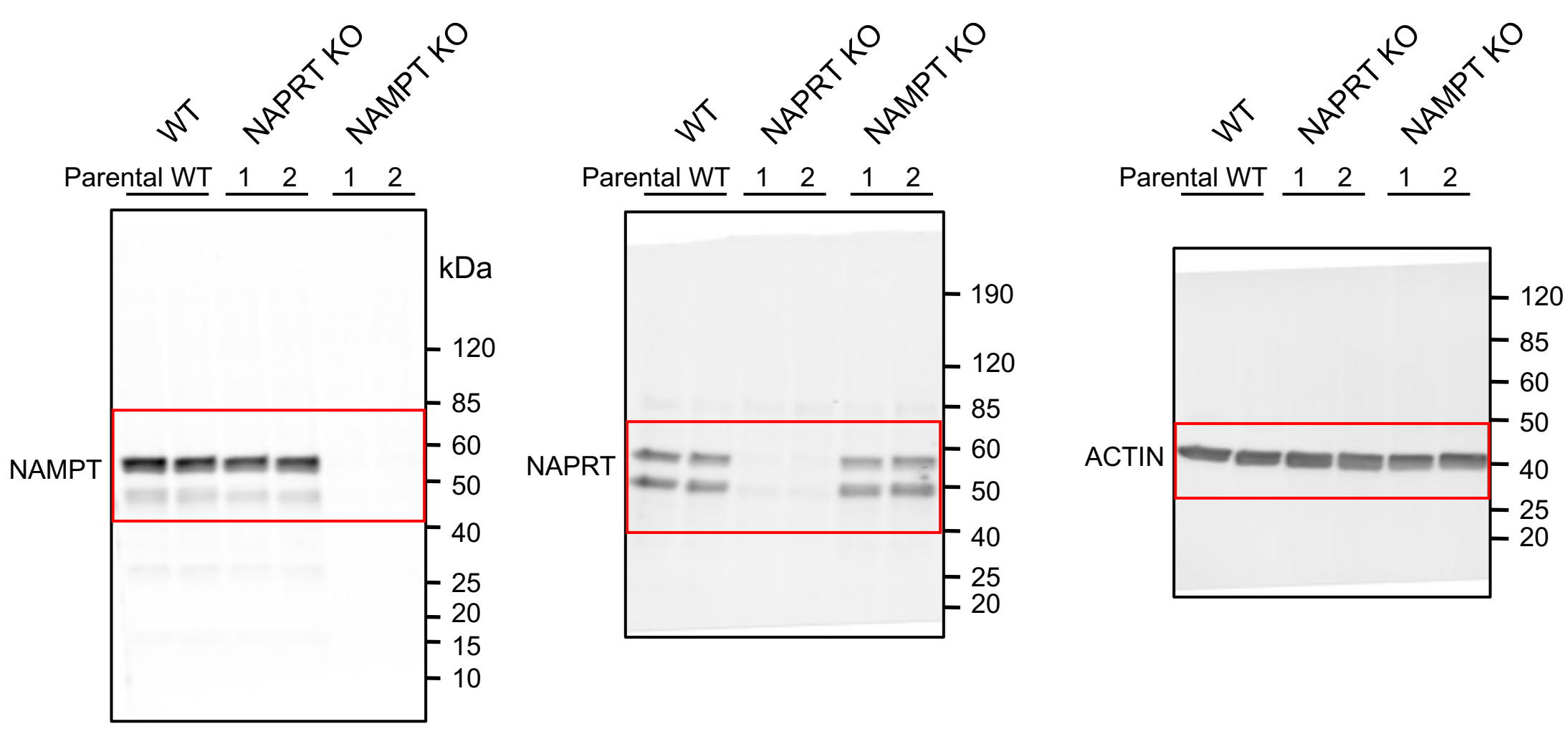

Supplementary Figure 3c

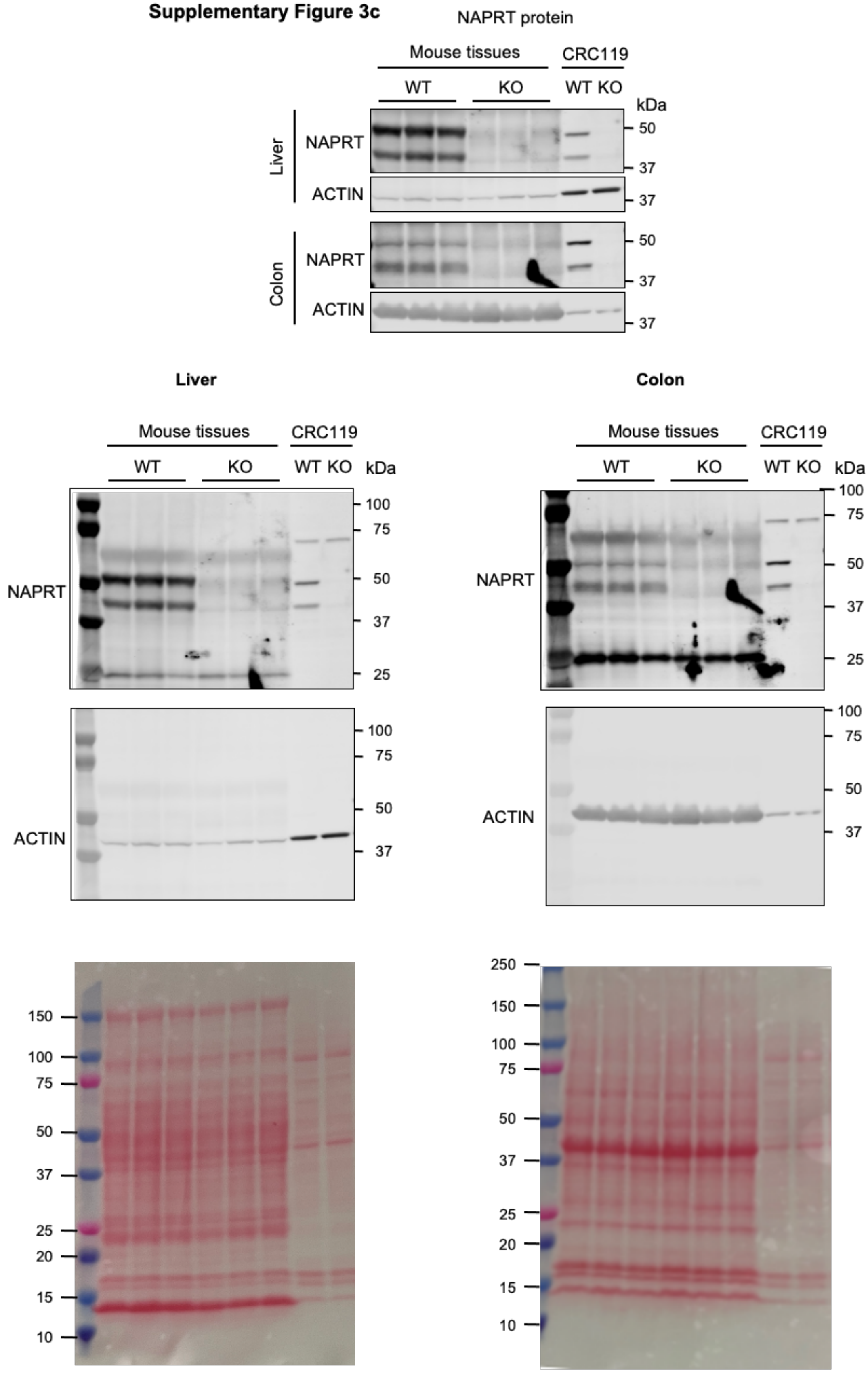

Supplement: Supplementary file 3 [file 41467_2026_68998_MOESM1_ESM.pdf]
