# Supplementary material for: NAPRT-mediated deamidated NAD biosynthesis enhances colon tissue resiliency and suppresses tumorigenesis
Source: Nat Commun. 2026 Feb 10;17:8651. doi: 10.1038/s41467-026-68998-w (PMC13490428; doi:10.1038/s41467-026-68998-w)
Supplement: Supplementary file 4 [file 41467_2026_68998_MOESM2_ESM.pdf]

Description of Additional Supplementary Files

Supplementary Data 1. Parameters and results of named metabolites in the metabolomics analysis of the colon from AOM treated mice

Supplementary Data 2. DEGs from RNA-seq analysis of the colon from untreated KO vs WT mice

Supplementary Data 3. Four clusters of AOM altered genes in the colon of KO vs WT mice

Supplementary Data 4. scRNA-seq data of DSS-treated mice

Supplementary Data 5. Genes correlated with NAMPT or NAPRT in GSE111889

Supplementary Data 6. Expression of *NAMPT* and *NAPRT* in human tumors vs normal tissues

Supplementary Data 7. Oligonucleotide and primer sequences

Supplementary Data 8. Representative LC-MS spectra of NAD metabolites from mouse tissues

Supplementary Data 9. NUA parameters and representative spectra

Supplementary Data 10. Plasma NA, NAM, and NAR quantification and representative spectral peaks

Supplementary Data 11. Metabolomics workflow and parameters

Supplementary Data 12: Antibodies and other resources
